# Technology Roadmap of Micro/Nanorobots

**DOI:** 10.1021/acsnano.5c03911

**Published:** 2025-06-27

**Authors:** Xiaohui Ju, Chuanrui Chen, Cagatay M. Oral, Semih Sevim, Ramin Golestanian, Mengmeng Sun, Negin Bouzari, Xiankun Lin, Mario Urso, Jong Seok Nam, Yujang Cho, Xia Peng, Fabian C. Landers, Shihao Yang, Azin Adibi, Nahid Taz, Raphael Wittkowski, Daniel Ahmed, Wei Wang, Veronika Magdanz, Mariana Medina-Sánchez, Maria Guix, Naimat Bari, Bahareh Behkam, Raymond Kapral, Yaxin Huang, Jinyao Tang, Ben Wang, Konstantin Morozov, Alexander Leshansky, Sarmad Ahmad Abbasi, Hongsoo Choi, Subhadip Ghosh, Bárbara Borges Fernandes, Giuseppe Battaglia, Peer Fischer, Ambarish Ghosh, Beatriz Jurado Sánchez, Alberto Escarpa, Quentin Martinet, Jérémie Palacci, Eric Lauga, Jeffrey Moran, Miguel A. Ramos-Docampo, Brigitte Städler, Ramón Santiago Herrera Restrepo, Gilad Yossifon, James D. Nicholas, Jordi Ignés-Mullol, Josep Puigmartí-Luis, Yutong Liu, Lauren D. Zarzar, C. Wyatt Shields, Longqiu Li, Shanshan Li, Xing Ma, David H. Gracias, Orlin Velev, Samuel Sánchez, Maria Jose Esplandiu, Juliane Simmchen, Antonio Lobosco, Sarthak Misra, Zhiguang Wu, Jinxing Li, Alexander Kuhn, Amir Nourhani, Tijana Maric, Ze Xiong, Amirreza Aghakhani, Yongfeng Mei, Yingfeng Tu, Fei Peng, Eric Diller, Mahmut Selman Sakar, Ayusman Sen, Junhui Law, Yu Sun, Abdon Pena-Francesch, Katherine Villa, Huaizhi Li, Donglei Emma Fan, Kang Liang, Tony Jun Huang, Xiang-Zhong Chen, Songsong Tang, Xueji Zhang, Jizhai Cui, Hong Wang, Wei Gao, Vineeth Kumar Bandari, Oliver G. Schmidt, Xianghua Wu, Jianguo Guan, Metin Sitti, Bradley J. Nelson, Salvador Pané, Li Zhang, Hamed Shahsavan, Qiang He, Il-Doo Kim, Joseph Wang, Martin Pumera

**Affiliations:** 1 Central European Institute of Technology, 48274 Brno University of Technology, Purkyňova 123, Brno 61200, Czech Republic; 2 The Aiiso Yufeng Li Family Department of Chemical and Nano Engineering, 8784 University of California San Diego, La Jolla, California 92093, United States; 3 Multi-Scale Robotics Lab, Institute of Robotics and Intelligent Systems, 27219 ETH Zurich, Tannenstrasse 3, Zurich 8092, Switzerland; 4 Max Planck Institute for Dynamics and Self-Organization (MPI-DS), 37077 Göttingen, Germany; 5 Department of Mechanical and Automation Engineering, 26451 The Chinese University of Hong Kong, Shatin, Hong Kong, 999077, China; 6 Department of Chemical Engineering, Institute for Polymer Research, Center for Bioengineering and Biotechnology, Waterloo Institute for Nanotechnology, 8430 University of Waterloo, 200 University Ave. W, Waterloo, ON N2L 3G1, Canada; 7 School of Medicine and Health, 47822 Harbin Institute of Technology, Harbin 150080, China; 8 Dipartimento di Fisica e Astronomia “Ettore Majorana”, Università degli Studi di Catania, via S. Sofia 64, Catania 95123, Italy; 9 Department of Materials Science and Engineering, 34968 Korea Advanced Institute of Science and Technology (KAIST), 291 Daehak-ro, Yuseong-gu, Daejeon 34141, Republic of Korea; 10 Department of Mechanical and Automation Engineering, The Chinese University of Hong Kong, Shatin, Hong Kong 999077, China; 11 Department of Chemical Engineering, Faculty of Engineering, University of Waterloo, Waterloo, ON N2L3G1, Canada; 12 Acoustic Robotics Systems Lab, Institute of Robotics and Intelligent Systems, ETH Zurich, Rüschlikon, CH 8803, Switzerland; 13 DWI - Leibniz Institute for Interactive Materials, Department of Physics, RWTH Aachen University, Forckenbeckstr. 50, 52074 Aachen, Germany; 14 School of Materials Science and Engineering, Harbin Institute of Technology (Shenzhen), Shenzhen, Guangdong 518055, China; 15 Systems Design and Biomedical Engineering, Centre for Bioengineering and Biotechnology, Waterloo Institute for Nanotechnology, 8430 University of Waterloo, 200 University Ave. W, Waterloo, ON N2L 3G1, Canada; 16 CIC nanoGUNE BRTA, Tolosa Hiribidea 76, E-20018 Donostia − San Sebastian, Spain; 17 Department of Materials Science and Physical Chemistry, Institute of Theoretical and Computational Chemistry, 16724 University of Barcelona, 08028 Barcelona, Spain; 18 Mechanical Engineering Department, 1757 Virginia Tech, Blacksburg, Virginia 24061, United States; 19 Mechanical Engineering Department, School of Biomedical Engineering and Sciences, Biological Systems Engineering Department, Institute For Critical Technology and Applied Science, Virginia Tech, Blacksburg, Virginia 24061, United States; 20 Chemical Physics Theory Group, Department of Chemistry, 7938 University of Toronto, Toronto, Ontario M5S 3H6, Canada; 21 Department of Chemistry, The University of Hong Kong, Hong Kong 999077, China;; 22 State Key Laboratory of Synthetic Chemistry, The University of Hong Kong, Hong Kong 999077, China; 23 College of Chemistry and Environmental Engineering, 47890 Shenzhen University, Shenzhen 518060, China; 24 Department of Chemical Engineering, Technion − Israel Institute of Technology, Haifa 32000, Israel; 25 Department of Robotics and Mechatronics Engineering, Daegu Gyeongbuk Institute of Science and Technology (DGIST), Daegu 42988, South Korea; 26 DGIST-ETH Microrobotics Research Center, DGIST, Daegu 42988, South Korea; 27 Institute for Bioengineering of Catalunya (IBEC), The Barcelona Institute of Science and Technology, Barcelona 08028, Spain; 28 Max Planck Institute for Medical Research, Universität ​Heidelberg, Heidelberg 69120, Germany; 28a Institute for Molecular Systems Engineering and Advanced Materials, Universität Heidelberg, Heidelberg 69120, Germany; 29 Department of Physics, Indian Institute of Science, Bangalore 560012, India; 30 Department of Analytical Chemistry, Physical Chemistry, and Chemical Engineering, Universidad de Alcala, Alcala de Henares E-28802 Madrid, Spain; 31 Institute of Science and Technology Austria (ISTA), Klosterneuburg 3400, Austria; 32 Department of Applied Mathematics and Theoretical Physics, Centre for Mathematical Sciences, 2152 University of Cambridge, Wilberforce Road, Cambridge CB3 0WA, United Kingdom; 33 Department of Mechanical Engineering, 3298 George Mason University, Manassas, Virginia 20110, United States; 34 Interdisciplinary Nanoscience Center (iNANO), Aarhus University, Gustav Wieds Vej 14, Aarhus 8000, Denmark; 35 Department of Materials Science and Physical Chemistry, University of Barcelona, 08028 Barcelona, Spain; 36 School of Mechanical Engineering and Department of Biomedical Engineering, University of Tel-Aviv, Tel-Aviv 69978, Israel; 37 Departament de Ciència de Materials i Química Física, Institut de Química Teòrica i Computacional, Universitat de Barcelona, Barcelona 08028, Spain; 38 Departament de Ciència de Materials i Química Física, Institute of Nanoscience and Nanotechnology (IN2UB), Universitat de Barcelona, Barcelona 08028, Spain; 39 Catalan Institution for Research and Advanced Studies (ICREA), Passeig de Lluís Companys, 23, Barcelona, 08010, Spain; 40 Department of Chemistry, 8082 The Pennsylvania State University, University Park, Pennsylvania 16802 United States; 41 Department of Materials Science and Engineering, The Pennsylvania State University, University Park, Pennsylvania, 16802, United States; 42 Department of Chemical and Biological Engineering, University of Colorado Boulder, Boulder, Colorado 80303, United States; 43 Key Laboratory of Microsystems and Microstructures Manufacturing (Ministry of Education), Harbin 15001, China; 44 Sauvage Laboratory for Smart Materials, School of Integrated Circuits, 529484 Harbin Institute of Technology (Shenzhen), Shenzhen 518055, China; 45 Department of Chemical and Biomolecular Engineering, 1466 Johns Hopkins University, Baltimore, Maryland 21218, United States; 46 Department of Chemical and Biomolecular Engineering, 6798 North Carolina State University, Raleigh, North Carolina 27695−7905, United States; 47 Institute for Bioengineering of Catalonia (IBEC), The Barcelona Institute for Science and Technology (BIST), Baldiri i Reixac 10-12, Barcelona 08028, Spain; 48 Catalan Institute of Nanoscience and Nanotechnology (ICN2), CSIC and BIST, Campus UAB, E-08193 Bellaterra, Barcelona, Spain; 49 Pure and Applied Chemistry, University of Strathclyde, Cathedral Street, Glasgow, G1 1BX, United Kingdom; 50 Surgical Robotics Laboratory, Department of Biomechanical Engineering, University of Twente, 7522 NB, Enschede, The Netherlands; 51 Surgical Robotics Laboratory, Department of Biomaterials and Biomedical Technology, University of Groningen and University Medical Center, Groningen 9713, AV, Groningen, The Netherlands; 52 State Key Laboratory of Robotics and System, 47822 Harbin Institute of Technology, Harbin 150080, China; 53 Department of Biomedical Engineering and Institute for Quantitative Health Science and Engineering, 3078 Michigan State University, 775 Woodlot Dr., East Lansing Michigan, 48824, United States; 54 University Bordeaux, CNRS, Bordeaux INP, ISM UMR 5255, 33607 Pessac, France; 55 Department of Mechanical Engineering, Department of Biology, Biomimicry Research and Innovation Center (BRIC), 1076 University of Akron, Akron, Ohio 44325, United States; 56 The Danish National Research Foundation and Villum Foundation’s Center for Intelligent Drug Delivery and Sensing Using Microcontainers and Nanomechanics (IDUN), Department of Health Technology, 5205 Technical University of Denmark, Ørsted Plads, Kgs, Lyngby 2800, Denmark; 57 Wireless and Smart Bioelectronics Lab, School of Biomedical Engineering, 387433 ShanghaiTech University, Shanghai 201210, China; 58 Institute for Biomaterials and Biomolecular Systems, 9149 University of Stuttgart, 70569 Stuttgart, Germany; 59 Department of Materials Science and International Institute of Intelligent Nanorobots and Nanosystems, State Key Laboratory of Surface Physics, 12478 Fudan University, Shanghai 200438, China; 60 NMPA Key Laboratory for Research and Evaluation of Drug Metabolism and Guangdong Provincial Key Laboratory of New Drug Screening, School of Pharmaceutical Sciences, 70570 Southern Medical University, Guangzhou 510515, China; 61 School of Materials Science and Engineering, 26469 Sun Yat-Sen University, Guangzhou 510275, China; 62 Department of Mechanical and Industrial Engineering, 7938 University of Toronto, 5 King’s College Road, Toronto, Ontario M5S 3G8, Canada; 63 Institute of Mechanical Engineering, 27218 Ecole Polytechnique Fédérale de Lausanne, CH-1015 Lausanne, Switzerland; 64 Departments of Chemistry, Chemical Engineering, and Materials Science and Engineering, 8082 The Pennsylvania State University, University Park, Pennsylvania 16802, United States; 65 Robotics Institute, University of Toronto, Toronto, ON M5S 3G8, Canada; 66 Institute of Biomaterials and Biomedical Engineering, University of Toronto, Toronto, ON M5S 1A1, Canada; 67 Department of Materials Science and Engineering, University of Michigan, Ann Arbor, Michigan 48109, United States; 68 Institute of Chemical Research of Catalonia (ICIQ), The Barcelona Institute of Science and Technology (BIST), Av. Països Catalans, 16, Tarragona E-43007 Spain; 69 Materials Science and Engineering Program, Texas Materials Institute, 12330 University of Texas at Austin, Austin Texas 78712, United States; 70 Walker Department of Mechanical Engineering, University of Texas at Austin, Austin, Texas 78712, United States; 71 School of Chemical Engineering, The University of New South Wales, Sydney, NSW 2052 Australia; 72 Department of Mechanical Engineering and Materials Science, 3065 Duke University, Durham, North Carolina 27709, United States; 73 International Institute of Intelligent Nanorobots and Nanosystems, Fudan University, Shanghai 200438, China; 74 Andrew and Peggy Cherng Department of Medical Engineering, Division of Engineering and Applied Science, 6469 California Institute of Technology, Pasadena, California 91125, United States; 75 Guangdong Key Laboratory of Biomedical Measurements and Ultrasound Imaging, School of Biomedical Engineering, Health Science Center, Shenzhen University, Shenzhen 518060, Guangdong, China; 77 School of Chemical Engineering and Technology, China University of Mining and Technology, Xuzhou, Jiangsu 221116, China; 78 Research Center for Materials, Architectures and Integration of Nanomembranes (MAIN), Chemnitz University of Technology, 09126 Chemnitz, Germany; 79 International Institute for Intelligent Nanorobots and Nanosystems (IIINN), Fudan University, Shanghai 200438, China; 80 State Key Laboratory of Advanced Technology for Materials Synthesis and Processing, International School of Materials Science and Engineering, 12565 Wuhan University of Technology, Wuhan 430070, China; 81 School of Medicine and College of Engineering, Koç University, 34450 Istanbul, Turkey; 82 Chow Yuk Ho Technology Centre for Innovative Medicine, The Chinese University of Hong Kong, Shatin, Hong Kong 999077, China; 83 Department of Chemical and Biomolecular Engineering, Yonsei University, 50 Yonsei-ro, Seodaemun-gu, Seoul 03722, Korea; 84 Rudolf Peierls Centre for Theoretical Physics, 6396 University of Oxford, Oxford OX1 3PU, United Kingdom; 85 Physical Intelligence Department, Max Planck Institute for Intelligent Systems, Heisenbergstr. 3, Stuttgart 70569, Germany; 86 Key Laboratory of Science and Engineering for the Multi-modal Prevention and Control of Major Chronic Diseases, Ministry of Industry and Information Technology, HIT Zhengzhou Research Institute, Zhengzhou 450000, China; 87 Key Laboratory of Microsystems and Microstructures Manufacturing (Ministry of Education), Harbin Institute of Technology, 150080 Harbin, China; 88 CNR-IMM, via S. Sofia 64, Catania 95123, Italy; 89 Ikerbasque, Basque Foundation for Science, Plaza Euskadi 5, 48009 Bilbao, Spain; 90 Chair of Micro- NanoSystems, Center for Molecular Bioengineering (B CUBE), Dresden 01307, Germany; 91 HKU-CAS Joint Laboratory on New Materials and Department of Chemistry, Hong Kong 999077, China; 92 Materials Innovation Institute for Life Sciences and Energy (MILES), The University of Hong Kong, Hong Kong 999077, China; 93 Center for Nanomedicine, Institute for Basic Science (IBS), Seoul 560012, Republic of Korea; 94 Department of Nano Biomedical Engineering (NanoBME), Advanced Science Institute, Yonsei University, Seoul 03722, Republic of Korea; 95 Centre for Nano Science and Engineering, Indian Institute of Science, Bangalore 560012, India; 96 Chemical Research Institute “Andres M. Del Río”, Universidad de Alcala, Alcala de Henares, E-28802 Madrid, Spain; 97 Materials Research Institute, The Pennsylvania State University, University Park, Pennsylvania 16802, United States; 98 Catalan Institute for Research and Advanced Studies (ICREA) Passeig Lluis Companys 23, Barcelona 08010, Spain; 99 State Key Laboratory of Advanced Medical Materials and Devices, ShanghaiTech University, Shanghai 201210, China; 100 Shanghai Clinical Research and Trial Center, Shanghai 201210 China; 101 Yiwu Research Institute of Fudan University, Yiwu 322000, Zhejiang, China; 102 Shanghai Frontiers Science Research Base of Intelligent Optoelectronics and Perception, Institute of Optoelectronics, Fudan University, Shanghai 200438, China; 103 Department of Electrical and Computer Engineering, University of Toronto, Toronto, ON M5S 1A1, Canada; 104 Department of Computer Science, University of Toronto, Toronto, ON M5S 1A1, Canada; 105 Chandra Department of Electric and Computer Engineering, University of Texas at Austin, Austin, Texas 78712, United States; 106 Australian Centre for NanoMedicine, The University of New South Wales, Sydney, NSW 2052 Australia; 107 Graduate School of Biomedical Engineering, The University of New South Wales, Sydney, NSW 2052 Australia; 108 Multi-Scale Medical Robotics Center, Hong Kong Science Park, Shatin NT, Hong Kong 999077, China; 109 T Stone Robotics Institute, The Chinese University of Hong Kong, Shatin, Hong Kong 999077, China; 110 Department of Surgery, 26451 The Chinese University of Hong Kong, Shatin, Hong Kong 999077, China; 111 Department of Biomedical Engineering, National University of Singapore, Singapore 117583, Singapore; 112 Institute of Robotics and Intelligent Systems, Dalian University of Technology, Dalian 116024, China; 113 State Key Laboratory of Photovoltaic Science and Technology, Fudan University, Shanghai 200438, China; 114 Department of Condensed Matter Physics, University of Barcelona, Martí i Franquès, 1-11, Barcelona, 08028, Spain

**Keywords:** micro/nanorobots, smart materials, propulsion, functionality, intelligence, collective behavior, nanotechnology, technological translation

## Abstract

Inspired by Richard Feynman’s 1959 lecture and the 1966 film *Fantastic Voyage*, the field of micro/nanorobots has evolved from science fiction to reality, with significant advancements in biomedical and environmental applications. Despite the rapid progress, the deployment of functional micro/nanorobots remains limited. This review of the technology roadmap identifies key challenges hindering their widespread use, focusing on propulsion mechanisms, fundamental theoretical aspects, collective behavior, material design, and embodied intelligence. We explore the current state of micro/nanorobot technology, with an emphasis on applications in biomedicine, environmental remediation, analytical sensing, and other industrial technological aspects. Additionally, we analyze issues related to scaling up production, commercialization, and regulatory frameworks that are crucial for transitioning from research to practical applications. We also emphasize the need for interdisciplinary collaboration to address both technical and nontechnical challenges, such as sustainability, ethics, and business considerations. Finally, we propose a roadmap for future research to accelerate the development of micro/nanorobots, positioning them as essential tools for addressing grand challenges and enhancing the quality of life.

## Graphical abstract

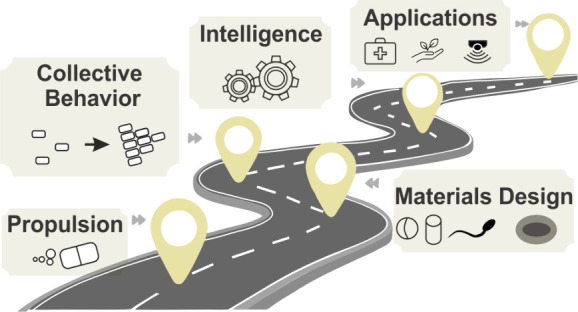

## 1 Introduction

### 1.1 History of Micro/Nanorobots: From Science Fiction to Reality

Richard Feynman’s 1959 lecture, “There’s Plenty of Room at the Bottom” and the 1966 science fiction movie *Fantastic Voyage*, captured the world’s imagination and inspired the development of today’s nanotechnology and micro/nanorobotics field, driving the design of functional micro/nanoscale machines that perform complex tasks.^1^ Nearly six decades later, this fictional vision is coming closer to reality, with synthetic micro/nanoscale machines being increasingly used in various applications, including the originally envisioned targeted drug delivery to previously inaccessible areas of the body.^2,3,4,5,6^ Currently, the development of such machines and the movement of micro/nanoscale objects are among the most exciting challenges facing nanotechnology.

Micro/nanoscale robots are classified based on their actuation mechanisms as chemically propelled robots or externally powered robots.^7,8,9^ While the chemically powered robots convert locally supplied fuels to force and movement, externally powered robots utilize magnetic, ultrasound, electrical, or optical fields to drive their motion.^10,11,12^ Initial efforts in the field of microscale robots were focused on advancing the propulsion, navigation, and cargo-towing capabilities of chemically or magnetically powered microrobots in different environments. Subsequent efforts over the past decade have led to new impressive technological advances and powerful capabilities, toward multifunctionality, collective behavior, intelligence, programmable navigation, hybrid control, biocompatibility, sensing, new fuels, transient behavior, and innovative manufacturing approaches that enable advanced biomedical and environmental applications of microrobots.^13,14,15^ In particular, functionalizing microscale robots with reactive and responsive materials and components allows them to perform specific tasks for biomedical or environmental applications. The field of micro/nanorobots has grown rapidly over the past two decades (Figure 1), leading to a new understanding of the propulsion and collective behaviors of microrobots, demonstrating new capabilities, and offering exciting new opportunities.

**Figure 1:**
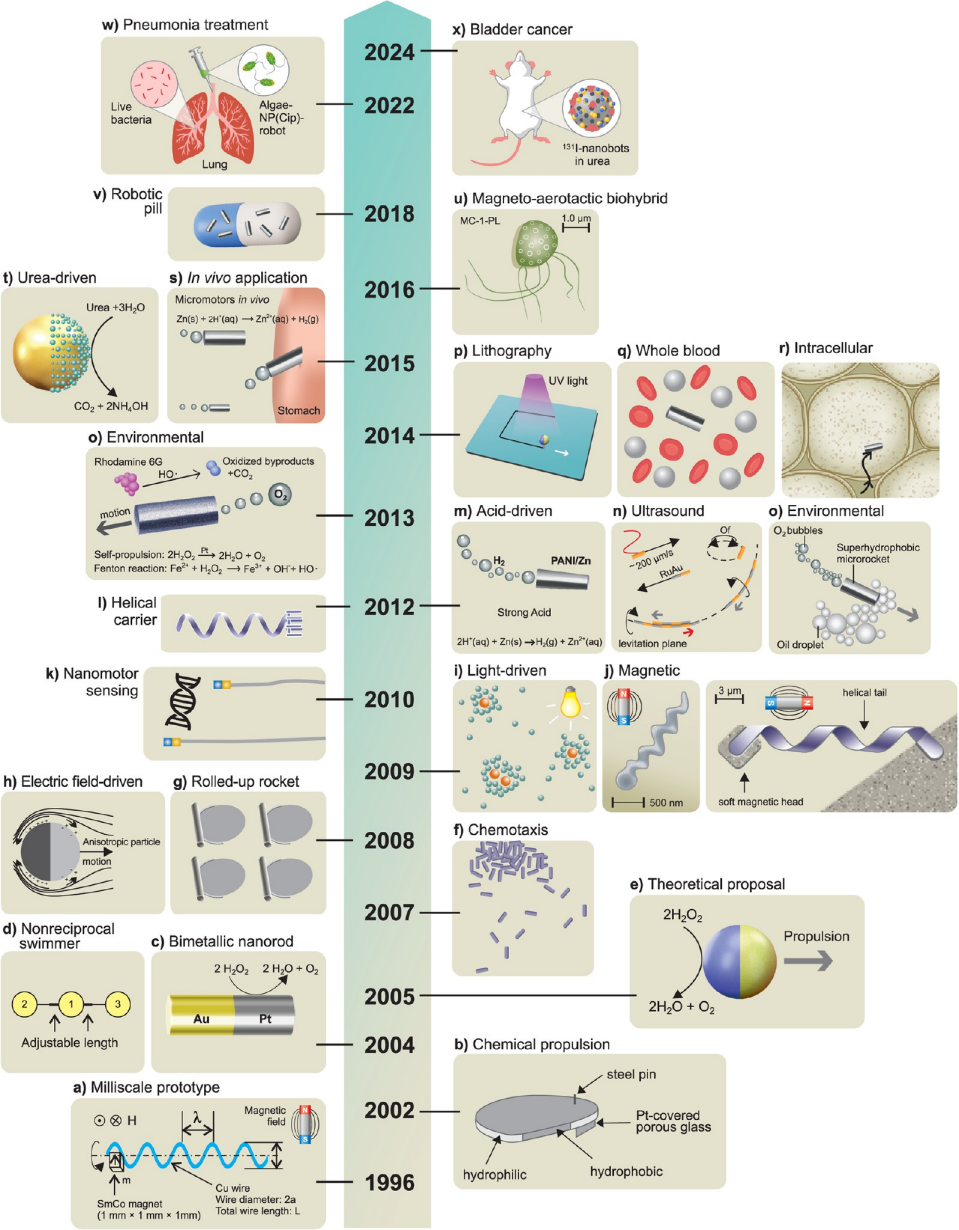
**Historical evolution of micro/nanorobotics. a)** Magnetic milliscale helical swimmer as a prototype. Reproduced from ref 47, Copyright 1996 IEEE. **b)** Chemically propelled self-assembled Pt motor. Reproduced from ref 25, Copyright 2002 WILEY-VCH. **c)** Self-propelled bimetallic nanorod *via* self-electrophoresis. Reproduced from ref 26, Copyright 2004 American Chemical Society. **d**) Theoretical proposal for a microswimmer containing three spheres linked by two rigid rods with changeable lengths. Reproduced from ref 23, Copyright 2004 American Physical Society. **e)** Self-propulsion of Janus spherical microrobots. Reproduced from refs 22, 176 , Copyright 2007 American Physical Society. **f)** Chemotaxis of nanomotors, Reproduced from ref 112, Copyright 2007 American Physical Society. **g)** Catalytic microrocket fabricated *via* roll-up technology. Reproduced from ref 28, Copyright 2008 WILEY-VCH. **h)** Electric-driven Janus microrobot. Reproduced from ref 76, Copyright 2008 American Physical Society. **i)** Light-driven semiconducting AgCl micromotor, Reproduced from ref 66, Copyright 2009 WILEY-VCH. **j)** Magnetic field-driven microrobots. Reproduced from refs 50, 51 , Copyright 2009 American Chemical Society. **k)** Nanomotor-based DNA sensing. Reproduced from ref 139, Copyright 2010 Springer Nature. **l)** Helical carrier operated by a magnetic field. Reproduced from ref 109, Copyright 2012 WILEY-VCH. **m)** Microrocket using gastric acid as fuel. Reproduced from ref 32, Copyright 2012 American Chemical Society. **n)** Ultrasound-propelled nanowires. Reproduced from ref 59, Copyright 2012 American Chemical Society. **o)** Micro/nanorobots for environmental applications. Reproduced from refs 147, 148 , Copyright 2012 and 2013 American Chemical Society. **p)** Nanomotor lithography. Reproduced from ref 103, Copyright 2014 Springer Nature. **q)** Helical nanomotor operates in whole blood. Reproduced from ref 52, Copyright 2014 American Chemical Society. **r)** Ultrasound nanomotor propelling inside living cells. Reproduced from ref 62, Copyright 2014 WILEY-VCH. **s)** First *in vivo* application of microrobots, using Zn-based microrockets propelling in mice stomach. Reproduced with permission under a Creative Commons CC-BY License from ref 126, Copyright 2015 American Chemical Society. **t)** Urea-powered enzymatic nanomotor. Reproduced with permission under a Creative Commons CC-BY License from ref 35, Copyright 2015 American Chemical Society. **u)** Magneto–aerotactic biohybrid micromotor for cancer treatment. Reproduced from ref 128, Copyright 2016 Springer Nature. **v)** Microrobotic pills containing drug-carrying Mg micromotors. Reproduced from ref 133, Copyright 2018 American Chemical Society. w) Biohybrid algae robots functionalized with ciprofloxacin loaded nanoparticles for acute pneumonia treatment. Reproduced from ref 131, Copyright 2022 Springer Nature. **x)** Urease-powered nanobots for radionuclide bladder cancer therapy. Reproduced with permission under a Creative Commons CC-BY License from ref 37, Copyright 2024 Springer Nature.

### 1.2 Early Efforts: Toward Nanoscale Locomotion and Navigation

Major advances in manufacturing and nanotechnology have facilitated the fabrication of microscale devices capable of propulsion at small scales.^13,16^ Prior to 2000, there were a few serious discussions of the use of nanorobots and nanotechnology especially within the human body.^17,18,19^ However, only within the last two decades have researchers developed strategies to practically fabricate and operate micro/nanorobots for such applications. Meanwhile, a variety of theoretical studies have provided crucial insights into the underlying mechanisms of self-propulsion at the nanoscale (see Section 3 for more details).^20,21,22,23^ In 2004, Golestanian *et al*. proposed a one-dimensional swimmer, composed of three interconnected spheres with adjustable rod lengths and driven by periodic nonreciprocal motion, demonstrating the possibility to swim at a low Reynolds number.^23^ Such studies laid the foundation for understanding low Reynolds number hydrodynamics and the principles of cell motility toward optimizing the design of artificial microscale swimmers and developing efficient locomotion strategies. As a result, two main approaches, based on external energy fields and harvesting energy from the environment, have been explored to power microscale robots.^24^

#### 1.2.1 Chemically Propelled Microrobots

This type of microrobots relies on creating spatially asymmetric chemical reactions. The first demonstration of self-propulsion of large centimeter-size objects in the presence of H_2_O_2_ fuel was demonstrated in 2002 by Whitesides and co-workers in connection to the spontaneous movement of asymmetric placement of a catalytic Pt strip on a millimeter-sized polydimethylsiloxane (PDMS) structure.^25^ Pioneering efforts by Sen and Mallouk at The Pennsylvania State University and by Ozin’s group in Toronto have led to the introduction of chemically powered nanoscale robots based on 2-μm long bi-segment (Pt-Au, Ni-Au) nanowires that display autonomous propulsion in aqueous solutions in the presence of H_2_O_2_ fuel.^22,26,27^ Such movement is based on the catalytic decomposition of H_2_O_2_ to O_2_ and H_2_O that leads to a self-electrophoresis mechanism. Following these pioneering studies from 2004 and 2005, numerous groups have contributed to the development of chemically powered microrobots.^22^

Bubble-propelled chemically powered tubular microengines (“microrockets”) were introduced in 2008 by Mei and Schmidt to address the limitation of catalytic nanowire robots in low-ionic strength environments.^28^ These open-tube conical microengines have an inner catalytic layer (commonly Pt) and an outer metal or metal oxide inert surface. The motion of these microrockets is due to the catalytic reaction of the fuel (commonly H_2_O_2_) on their inner surface, which induces the formation and expulsion of O_2_ bubbles through their wider opening, and the generation of a powerful thrust.^29^ These early microrockets were fabricated using an advanced rolled-up lithographic fabrication route.^30^ Wang’s group described in 2011 a template membrane-based electrodeposition synthesis of highly efficient and smaller (8-μm long) hollow polymer/Pt bilayer conical microtube engines.^31^ The same group introduced acid-powered microrockets in 2012, based on an inner Zn layer, for operation in the stomach gastric fluid where the acid–Zn reaction leads to H_2_ bubble thrust.^32^ Biocatalytic catalase layers were shown by Sánchez *et al*. in 2010 to be an attractive alternative to inner catalytic metal layers for propelling open-tube microengines in H_2_O_2_ solutions.^33^

Another common route for creating spatially asymmetric catalytic microrobots involving Janus microspheres was introduced in 2007 by Jones and Golestanian.^22^ These two-faced spherical Janus microrobots rely on a Pt catalytic cap (on an inert polymeric microsphere, commonly polystyrene) for the catalytic decomposition of H_2_O_2_ fuel that leads to efficient movement *via* a self-generated phoretic mechanism. Pumera’s group described the use of Pt-free catalysts, based on Ag and MnO_2_ microsphere surfaces, for efficient chemical propulsion of Janus microsphere robots.^34^ The increasing demand of the biomedical community has facilitated the propulsion fuel transforming from toxic substrate (*e.g.*, H_2_O_2_) to bioavailable fluid (*e.g.*, urea, glucose). Sánchez’s group described Janus microsphere robots, powered by the biocatalytic decomposition of urea, based on coating one-half of the particle with the enzyme urease.^35^ The biocatalytic engines have been integrated with various building entities (*i.e.*, platelet,^36^ SiO_2_,^37^ liposomes,^38^ vesicles,^39^ polymers,^40^ *etc*.) to harvest propulsive power from the local biological environment. Such a design eliminates the need for external fuel or power sources, enabling long-lasting movement inside the body.

#### 1.2.2 Magnetic Propulsion

Magnetic swimmers rely on the use of magnetic actuation to replicate the movement of natural microorganisms.^41^ Wireless magnetic actuation allows locomotion in an untethered manner while keeping the local environment intact.^42,43,44,45^ Such swimmers have been widely explored for diverse biomedical applications.^46^ The first magnetically powered helical structure was a millimeter-sized prototype presented in 1996 by Honda’s group.^47^ In 2007, Nelson’s group demonstrated the formation and magnetic propulsion of nanoscale helical structures.^48,49^ Such helical swimmers consisted of a soft magnetic “head” and a helical “tail”, mimicking bacterial flagella (*e.g.*, *Escherichia coli*). A low-strength rotating magnetic field was used to apply continuous torque to this artificial bacterial flagellum (ABF), enabling the swimmer to rotate and generate a corkscrew directional motion along its central axis. Such movement depends strongly on several factors such as the frequency, strength, and direction of the magnetic field, as well as the helix geometry, the properties of the coated magnetic layer, and the fluid viscosity. These helical swimmers were fabricated by a self-scrolling technique.^50^ In 2009, Ghosh and Fischer reported the fabrication of ultra-small helical swimmers with a diameter of 200–300 nm and a length of 1–2 μm using glancing angle deposition (GLAD) techniques.^51^ These helical swimmers were able to push microbeads with a diameter of 5 μm. Helical magnetic propellor robots have been shown to move in human blood^52^ and through the viscous vitreous humor of the eye.^53,54^

#### 1.2.3 Ultrasound-Powered Microrobots

Ultrasound is unique in its noninvasiveness, high biocompatibility, and deep penetration into biological tissues, making it ideal for the programmable manipulation of microrobots in the field of medicine.^55^ Unlike other methods, ultrasound manipulation does not require specific shapes or materials^56^ for microrobots and can be directly applied to actuate nanomaterials, colloids, living cells, and even entire organisms.^57^ In 2012, Mallouk, Wang, and their co-workers illustrated the use of ultrasonic acoustic waves to propel Au nanowires in biologically relevant environments, demonstrating that these robots can achieve fast axial directional motion (∼200 μm s^–1^) as well as in-plane rotation.^58,59^ The ability of MHz frequency acoustic waves to propel, align, rotate, and assemble metallic nanowires in aqueous media was illustrated. Ahmed *et al*. examined the influence of nanowire materials and shape on the acoustic movement of nanowire robots.^60^ A propulsion mechanism proposed for these acoustic nanowire robots suggests that asymmetric steady streaming is used to generate a finite propulsion speed along their symmetry axis and perpendicular to the oscillation direction.^61^ In 2012, researchers developed an innovative propulsion method utilizing ultrasound to vaporize a perfluorocarbon emulsion contained within a hollow micromachine, enabling exceptionally rapid, bullet-like motion.^58^ Later, the ultrasound-driven propulsion of such Au nanowires within cells was demonstrated by Mallouk *et al*.^62^ Similar Au nanowires, modified with small interfering RNA (siRNA), were used to perform intracellular gene delivery and gene silencing.^63^

#### 1.2.4 Light-Powered Microrobots

Light is an abundant, powerful, and versatile energy source that offers considerable promise for actuating and controlling wirelessly synthetic microrobots with high spatial and temporal resolutions.^64^ The speed of light-driven microrobots can be controlled by adjusting the light intensity and wavelength.^65^ The photoinduced activation of photocatalytic microrobots is mediated by the generation of electron-hole pairs, which migrate to the robot surface to participate in chemical reactions.^8^ Early light-driven microrobots were developed using TiO_2_ and AgCl particles, and then substantially extended to a wider range of semiconductor materials, including Fe_2_O_3_, Si, and BiVO_4_.^66–70^ A diverse array of wide bandgap semiconducting materials with high photocatalytic efficiency has enabled the design of a variety of smart microrobots exhibiting different behaviors, including the precise control over motion speed, direction, and responsive collective behaviors.^71,72,73^ Efficient light-induced self-electrophoresis propulsion of TiO_2_/Au Janus microspheres was demonstrated in the presence of pure water using low-intensity UV light.^74^

#### 1.2.5 Electrical-Driven Microrobots

In 2006, Velev’s group demonstrated how miniature semiconductor diode “particles” suspended in water propel themselves electro-osmotically.^75^ Such externally powered propulsion involves harvesting electric energy from external AC fields (applied *via* remotely positioned electrodes) and then converting it to mechanical propulsion.^76,77^ Calvo-Marzal *et al*. reported on the electrical-driven propulsion of semiconductor diode nanowires induced by an external AC electric field.^77^ In 2010, Kuhn *et al*. utilized electric field-induced polarization to trigger spatially separated oxidation and reduction reactions on a microrobot surface, resulting in directional motion, either based on its controlled dissolution at one extremity and regeneration at the opposite end,^78^ or due to asymmetric gas bubble formation.^79^ AC electrical fields have been used also for the efficient propulsion of metallo–dielectric Janus microspheres, as well as for simultaneous cargo loading, transport and release using a single external electric field.^80^ It is probably useful to note that the mechanism is known as “induced-charge electrophoresis” (ICEP). At higher electric frequencies, a different self-propulsion mechanism, termed self-dielectrophoresis, was discovered by Boymelgreen *et al*.^81,82^ Furthermore, DC electric fields can drive polymer microspheres into directional motion in insulating oils, a system known as “Quincke rollers”.^83^

#### 1.2.6 Hybrid Microrobots

Hybrid micro/nanoscale robots powered by multiple distinct energy sources achieve performance levels unattainable with a single propulsion method.^84,85^ Creating such hybrid microrobots requires careful attention to the different requirements of the individual propulsion within a single nanoscale device. Such a dual-propulsion mode of hybrid nanorobots increases the versatility, broadens the scope of operation of microscale robots, and improves their adaptability in changing environments. In 2011, Gao *et al*. introduced the first hybrid microrobot powered by chemical and magnetic sources for operation under changing environments.^86^ Such hybrid fuel-driven and fuel-free movement relied on a flexible hybrid Pt-Au-Ag_flex_-Ni nanowire robot. In 2015, Li *et al*. designed a fuel-free magneto-acoustic hybrid nanorobot, combining a magnetic helical structure and a concave nanorod end, which can be powered by either a magnetic or ultrasound field.^87^ An alternative concept, proposed by Kuhn *et al.*, combines chemical fuel with magnetic fields, using the intrinsically present Lorentz force to control the trajectory of Janus microrobots without the need for any ferromagnetic components in the robot’s design.^88^

Biological-based micromotors are nature’s micro/nanoactuators. Soong *et al*. created one of the earliest hybrid organic-inorganic nanodevices in 2000.^89^ They fabricated nanorods, integrated with a bacterial F_1_-ATPase rotary motor *via* Ni-capped post using differential attachment chemistry. Biomotors within intact cells or whole-cell actuators were introduced a few years later and the field of *biohybrid microrobotics* was born. Biohybrid microrobots are created by integrating whole cells with synthetic micro/nanofabricated components. Whole-cell actuators have distinct advantages. They can metabolize simple fuel (*e.g.*, glucose), self-replicate inexpensively, and self-regenerate. They are also equipped with highly versatile and sensitive sensory systems that can be harnessed to regulate the biomotors’ motion through innate or synthetic signaling networks. Thus, the biotic component of biohybrid microrobots is exploited for actuation, sensing, and control. Seminal works in this area demonstrated the cardiomyocyte-powered movement of a self-assembled muscle-MEMS system^90^ and controlled load transport using bacteria,^91,92,93^ algae,^94^ and sperm.^95^

### 1.3 Navigation and Cargo Transport

The navigation of microrobots with high spatial and temporal precision is critical for the diverse operations of such microscale machines.^96^ Major efforts have thus been devoted to advancing the motion control of microrobots toward targeted destinations and achieving fully autonomous operation of such microscale vehicles. Various internal (chemotaxis and chemokinesis) and external stimuli (such as magnetic fields, light, ultrasound, and heat) have thus been shown to be useful for controlling the speed and directing the movement of artificial nanorobots. For example, thermal modulations were utilized for regulating the moving speed of Pt/Au nanorobots by controlling the rate of the surface catalytic reaction.^97^ Schmidt *et al*. demonstrated the ability to thermally control the motion behavior of a catalytic microrocket by changing the shape of the thermal-responsive tubular layer to allow repeated on/off cycles at different temperatures.^98^ Guan’s group demonstrates biomimetic chemotaxis in synthetic micromotors, where ZnO-based Janus micromotors not only autonomously move using biocompatible CO_2_ fuel but also actively self-reorient to follow CO_2_ gradients.^99^

#### 1.3.1 Motion Control

Embedding a magnetic segment or layer in nanowire and microrocket robots, respectively, is extremely useful for the magnetic guidance of microrobots.^100,101^ Such guidance of catalytic nanowire robots was demonstrated first by Kline *et al*. who incorporated a ferromagnetic (Ni) segment that can be magnetized by an external magnetic field.^100^ Magnetic steering of cargo-towing catalytic nanowire robots has led to the demonstration of their navigation in complex microchannel networks along with directional cargo transport through such microfluidic networks.^101^ Autonomous collision-free navigation of microscale robots in complex maze-like microstructures has been achieved by combining magnetic guidance along with an artificial intelligence (AI) planner.^102^ Magnetically powered microrobots can also be guided by adjusting the orientation of the applied homogeneous magnetic field. Such guidance has facilitated novel applications such as nanorobot-enabled lithography.^103^ Self-navigation has been demonstrated by taking advantage of tactic movements toward favorable regions or away from harmful areas.^104,105^ Such self-targeting capability along gradients of various fields (toward favorable regions and away from harmful areas) allows microrobots to self-navigate and move adaptively by responding to their surrounding’s gradients, thus mimicking the tactic movement of living organisms.^106,107^ Controlled direction manipulation using biohybrid microrobots has been achieved by directly signaling the biomotor and the sensory response system for autonomous control of the biomotor output, or guiding the microrobot’s movement using an externally applied driving force. Behkam and Sitti devised a chemical switching technique that directly addresses *Serratia marcescens* flagellar motors, enabling on/off motion control of bacteria-propelled microparticles.^92^ Whitesides’ group harnessed phototaxis response in the photosynthetic algae *Chlamydomonas reinhardtii* to steer microparticle-carrying algae using light as an input.^94^ Martel and co-workers demonstrated magnetically controlled manipulation of microparticles enabled by the magnetotactic *Magnetospirillum gryphiswaldense*.^93^

#### 1.3.2 Cargo Towing

The use of microrobots for cargo transport plays an important role in diverse applications, ranging from the delivery of therapeutic payloads to the capture and isolation of macromolecules or cells. Microrobots have been shown to enable the loading and transport of cargo by various mechanisms. Kagan *et al*. demonstrated in 2010 the ability of chemically powered nanowire robots to transport common drug carriers, such as drug-loaded liposomes and poly-d,l-lactic-*co*-glycolic acid particles, over predefined routes toward a predetermined destination.^108^ In 2012, parallel efforts at ETH Zurich demonstrated the use of magnetically powered helical micromachines, consisting of a helical body and a microholder, to capture and transport selected cargo payloads confined in the 3D microholder, consisting of six rigid, finger-like protrusions.^109^ Similarly, Ghosh and Fischer demonstrated the ability of helical micropropellers to carry and push different payloads^51^ while Gao *et al*. illustrated the ability of flexible magnetic Ni–Ag nanoswimmers to transport drug-loaded microparticles to HeLa cells in biological media.^110^

### 1.4 Imparting Collective Behavior

Many important applications of microrobots require cooperation between multiple microrobots, analogous to the organization of live microorganisms.^111,112^ Such interactions have inspired substantial research activity toward the self-organization of synthetic microscale robots. The efforts have thus been devoted to microrobot swarms that function collectively to accomplish challenging tasks that would be impossible using a single microrobot.^105^ The creation of such microrobot swarms commonly relies on the use of external stimuli (*e.g.*, light^67,113^ and magnetic field^114^) to create a gradient that promotes such interactions and assembly of microrobots. For example, microrobots powered by chemical gradients can respond to each other when their self-generated gradients overlap.

The pioneering work of Ibele *et al*. demonstrated the use of UV light to induce the swarming of AgCl and silica particles.^66^ Solovev *et al*. reported that self-propelled tubular microjets can be assembled into complex structures through signals originating from chemical reactions.^115^ Similarly, the collective self-organization of large assemblies of autonomous Mg-based microrobots can be triggered by chemical gradients.^116^ Kagan *et al*. demonstrated the schooling behavior of Au particles triggered by the addition of hydrazine,^117^ while Xu *et al*. described the use of ultrasound for inducing the assembly of chemically powered microrobots.^118^ Gao *et al*. demonstrated the use of hydrophobic interactions for the self-assembly of Janus microrobots with hydrophobic hemispheres.^119^

In biohybrid microrobots, collective behavior is achieved by broadcasting signals that elicit a response from the entire swarm, otherwise known as “centralized control”, or through agent–agent communication, otherwise known as “decentralized control”. Martel’s group showed the assembly of a miniature version of the Pyramid of Giza by centralized control of a swarm of thousands of magnetotactic bacteria in 2010.^120^ Behkam’ group demonstrated centralized chemotactic control of bacterial microrobots in 2011.^121^ They also demonstrated hybrid control of bacterial microrobots by implementing a hybrid centralized and decentralized control strategy, wherein centralized control was achieved by chemotaxis and decentralized control was achieved using population density-regulated response.^122^

### 1.5 Biomedical Applications of Micro/Nanorobots

The powerful capabilities of modern microscale robots offer considerable promise for diverse biomedical applications, including targeted drug delivery, sensing, or microsurgery, and should thus have a major impact on the treatment and prevention of diseases.^123,124,125^ Wang’s group described the first *in vivo* demonstration of synthetic microrobots in live animals.^126^ This study demonstrated the efficient propulsion of synthetic PEDOT/Zn tubular microrobots in the gastric acid of mouse stomachs without needing additional fuel. Esteban-Fernández de Ávila *et al*. reported the first *in vivo* therapeutic application of microrobots for active drug delivery for treating gastric bacterial infection in a mouse model.^127^ Martel’s group demonstrated the use of bacteria containing magnetic iron oxide nanocrystals to target active cancer cells deep inside tumors guided by magnetic field toward the tumor.^128^ Ullrich *et al*. demonstrated the locomotion of magnetic tubular microrobots inside the living rabbit eye toward intraocular microsurgery,^53,129^ while Fischer’s group demonstrated the long-term movement of a swarm of magnetic micropropellers through the gel-like vitreous body of a porcine eye.^54^ Recent efforts have demonstrated the successful biomedical application of microrobots in body organs. This includes work from Sánchez’s group on urea-powered biocatalytic Janus microsphere robots for treating bladder cancer^37,130^ and research from Wang’s group on algae-based biohybrid microrobots for eliminating pneumonia-causing bacteria in the lungs.^131^ Both studies illustrate a significantly higher accumulation of microrobots in the disease sites compared with the control groups. Ahmed’s group demonstrated the ability of acoustic microrobots to navigate in a living mouse brain toward drug delivery applications in the complex brain vasculature.^132^

#### 1.5.1 Robotic Pills

Six decades after Feynman predicted that one day we would be able to swallow the surgeon, in 2018, researchers at UCSD demonstrated the feasibility of using common pharmaceutical pill formulations to carry and administer drug-loaded Janus microsphere robots.^133^ The cargo-loaded microrobots, released from the dissolved pill in gastric fluid, maintained the attractive movement and transport capabilities of *in vitro* microrobots to offer strong retention of their payloads onto the stomach lining (compared to orally administrated passive microparticles and free cargo microrobots). The movement of the released microrobots is not influenced by their encapsulation within the pill or by the corresponding inactive excipient materials. Mg-based Janus microrobots embedded within pill formulations have led to a built-in stirring capability toward enhanced drug absorption rate and bioavailability.^134^ Such efforts are expected to facilitate the practical biomedical applications of microrobot technology.^135,136^

#### 1.5.2 Into the Cell

Reaching and operating in living cells represents the ultimate goal of nanoscale machines.^137^ Pioneering work from Mallouk’s group demonstrated the effective internalization of acoustically propelled Au nanowires into HeLa cells after 24 h incubation without affecting cell viability.^62^ An active intracellular propulsion of the internalized robots under an acoustic field, involving axial propulsion and spinning, was observed. These acoustically propelled Au nanowire robots have been subsequently used by Wang’s group for rapid intracellular miRNA sensing and enhanced siRNA delivery into cells.^63,138^

#### 1.5.3 Robot-Based Biosensing

The motion of microscale robots opens up unique opportunities for diverse biosensing applications.^2^ Wu *et al*. demonstrated the first example of synthetic nanorobots as a bioanalytical tool.^139^ This motion-based DNA hybridization sensing relied on the use of Ag nanoparticle tags for inducing nanorobot acceleration. The higher the concentration of the target nucleic acid, the greater the number of Ag nanoparticles captured and, hence, the higher the speed of the nanorobot.^140^ Subsequent efforts by Wang’s group demonstrated the utility of receptor-functionalized microrobots for efficient capture, transport, and isolation of target biological targets, such as circulating tumor cells.^141^ Micromotors functionalized with lectin, antibody, oligonucleotide, or aptamers bioreceptors have thus been shown to be extremely attractive as self-propelled micro-transporters for bacteria, cancer cells, nucleic acid, or proteins, respectively.^142^ This approach enabled the rapid isolation of biological targets directly from raw biological samples, eliminating the need for preparatory or washing steps and facilitating “on-the-fly” detection of various bioanalytes.^143^ Behkam’s group demonstrated the autonomous transport of nanocargo-carrying bacteria into orthotopic triple-negative breast cancer tumors in mice and achieved up to 100-fold enhancement in the penetration and distribution of the nanocargo compared to the passively diffusing nanocargo.^144^

### 1.6 Environmental Applications of Micro/Nanorobots

The continuous movement of microscale machines adds a new dimension to decontamination processes for environmental remediation, which leads to higher efficiency and shorter cleanup times.^145^ Functionalizing self-propelled microscale robots with advanced reactive materials has thus provided new opportunities for efficient motion-based “on-the-fly” remediation processes.^146^ In 2012, Guix *et al*. demonstrated the first example of microrobots removing oil pollutants based on efficient “on-the-fly” oil adsorption onto self-propelled tubular microengines functionalized with superhydrophobic *n*-alkanethiols chains.^147^ In 2013, Sánchez and Schmidt demonstrated the ability of tubular microrockets containing a reactive material (Fe) to degrade rapidly organic pollutants in water *via* the Fenton reaction in the presence of H_2_O_2_ fuel.^148^ Such Fenton oxidation generates the hydroxyl radical active intermediates that degrade organic contaminants. The efficient fluid mixing induced by bubble-propelled microrobots is extremely useful for accelerating the oxidative neutralization processes of organophosphate nerve agents.^149^ Pumera’s group initially demonstrated the degradation of wet wipes using Bi_2_WO_6_ spherical microrobots.^150^ This was followed by the report of “on-the-fly” removal of micro/nanoplastics from water using self-propelled light-powered photocatalytic and magnetic MXene-derived multi-layered microrobots.^151^ Following these pioneering studies, numerous groups have contributed to the development of new active microscale cleaners performing “on-the-fly” remediation activities, toward efficient decontamination of different types of pollutants.^145^

### 1.7 Terminology and Summary

After witnessing tremendous progress in the field over the past two decades, we feel the urgent need to provide a ***comprehensive review*** to navigate the intriguing aspects of micro/nanorobots by unraveling their historical developments, current status, and future challenges. The paper is organized into nine key sections, each exploring a distinct facet of this burgeoning field. Beginning with a historical overview, the introduction sets the stage for subsequent deep dives into propulsion mechanisms, theoretical foundations, collective behavior, intelligent functionalities, materials design, various applications, and technical scale-up. Each section is carefully organized to provide a holistic understanding of micro/nanorobots, covering their locomotion mechanisms, design principles, building materials, distinct capabilities, as well as diverse applications in biomedical, environmental, and engineering fields. The review concludes with a forward-looking perspective on the burning issues and grand challenges, emphasizing the need for creating autonomous motion and robotic operations, enhanced control and collective behavior, improved biocompatibility, multifunctionality, and translation from the laboratory into real-world applications.

#### 1.7.1 Terminology

Although we will primarily refer to “micro/nanorobots” in this review, many terms have been used to describe synthetic micro/nanoscale objects that transduce ambient energy into mechanical work. These include colloidal motors,^152^ micromotors,^153^ nanomotors,^26^ self-propelled particles,^154^ microbots,^155^ nanobots,^156^ active colloids,^157^ artificial microswimmers,^158^ micro- and nanorobots,^158^ and others.^159^ This variety of terms reflects the interdisciplinary nature of this field, which welcomes researchers from chemistry, biology, physics, engineering, and beyond. Of course, researchers from different technical backgrounds often use different terms to refer to the same objects. Unfortunately, this variety can create confusion, especially for newcomers. For example, a paper that exclusively uses the term “microrobots” may be difficult to find for someone who enters the term “active colloid” into a search engine. Below, we delineate the subtle but real differences among various terms that are often used in the literature:

***Micro/nanomotor*** is attractive as a generic term as the word “motor” connotes the conversion of one form of energy (such as electrical energy) into mechanical energy. Because this description applies to nearly all objects termed “micro/nanorobots”, this term applies to a wide range of scenarios, and we advocate for its broad usage.

***Micro/nanoswimmer***: In everyday usage, the verb “to swim”, generally implies locomotion through a liquid of a wide variety of creatures and cells (*e.g.*, bacteria, spermatozoa, fish, tadpoles, whales, or humans), typically by deforming themselves through a cyclic series of body motions. However, this description does not necessarily always apply to our field: many micro/nanorobots demonstrate locomotion despite having no moving parts. To minimize confusion, we thus recommend the use of “micro/nanoswimmer” to specifically describe systems that generate their motion through mechanical deformation. In the natural world, this includes most biological microswimmers (*e.g.*, swimming bacteria^160^). In the engineered world, micro/nanoswimmers include the three-link design introduced by Purcell in his seminal 1977 work^161^ and analyzed extensively by various groups,^162,163,164,165,166,167^ the three-sphere swimmer proposed and analyzed by Najafi and Golestanian^23^ and realized experimentally by Grosjean *et al.*,^168^ or the “Pushmepullyou” design proposed by Avron *et al*.^169^ It is worth noting that in Newtonian fluids, micro/nanoswimmers must satisfy the well-known scallop theorem, also due to Purcell (violations of the scallop theorem have been reported in non-Newtonian fluids as reviewed by ref 170).

***Active colloid*** is another common term for synthetic colloids that can transduce energy from their surroundings into motion. By “colloid”, we mean a solid particle or liquid droplet, generally on the order of 100 μm or smaller in size, suspended in a fluid. As defined, this term encompasses both colloidal particles that generate their own motion (*e.g.*, *via* chemical reactions) and those that require an external field to move. Examples of the latter include colloids whose motion is driven by an AC electric field,^171^ a static magnetic field,^172^ or an oscillatory^173^ or rotating magnetic field.^51,174,175^

***Self-propelled particles*** can be considered a subset of active colloids, but the term “self-propelled” implies that the particle can generate its motion without the need for an externally applied field. Under this definition, magnetically propelled helical particles^51,174,175^ would be considered active colloids but not self-propelled particles, as these objects cannot execute non-Brownian motion without an applied field.

***Micro/nanorobots*** encompass a broad variety of devices and functions that will be explored in detail in this comprehensive review. Here, we advocate for the adoption of uniform and consistent terminology to refer to different types of micro/nanorobotic devices and their cousins within the micro/nanorobotics community. To minimize confusion and foster the growth of this emerging field, we encourage the community to engage in discussions aimed at defining these terms more precisely and to apply them uniformly across research and publications. The adoption of more consistent terminology by the micro/nanorobotics community will not happen overnight. Defining this terminology will require a concerted effort and may necessitate the formation of “standards”, similar to those established by organizations such as ASTM (formerly the American Society for Testing and Materials) or ASHRAE (the American Society of Heating, Refrigerating and Air-Conditioning Engineers), to determine the accepted definitions for different types of micro/nanoscale active matter systems.

## 2 Propulsion

As an integral part of their design, micro/nanorobots should have component(s) to convert energy source(s) into locomotion. However, demonstrating locomotion is not a straightforward task considering the challenging environments in which micro/nanorobots operate, such as the human body or contaminated water. Furthermore, the size range of micro/nanorobots further complicates their locomotion capabilities. Unlike the common depiction in science fiction movies, scaling down a macroscopic robot into its microscale counterpart is not a reliable strategy in real-world conditions. In such a direct miniaturization, the resulting robots would not work because the physics of swimming at the macroscale is fundamentally different than that of the microscale. In this section, we initially cover the physical principles of swimming at small scales (Figure 2a and Figure 2b). Then, we introduce locomotion mechanisms in three different categories, *i.e.*, chemical (Figure 2c), physical (Figure 2d), and biohybrid (Figure 2e) approaches. It is important to note that this Review does not follow a universal nomenclature for equations. Instead, parameters and nomenclature are defined as each equation is introduced.

**Figure 2:**
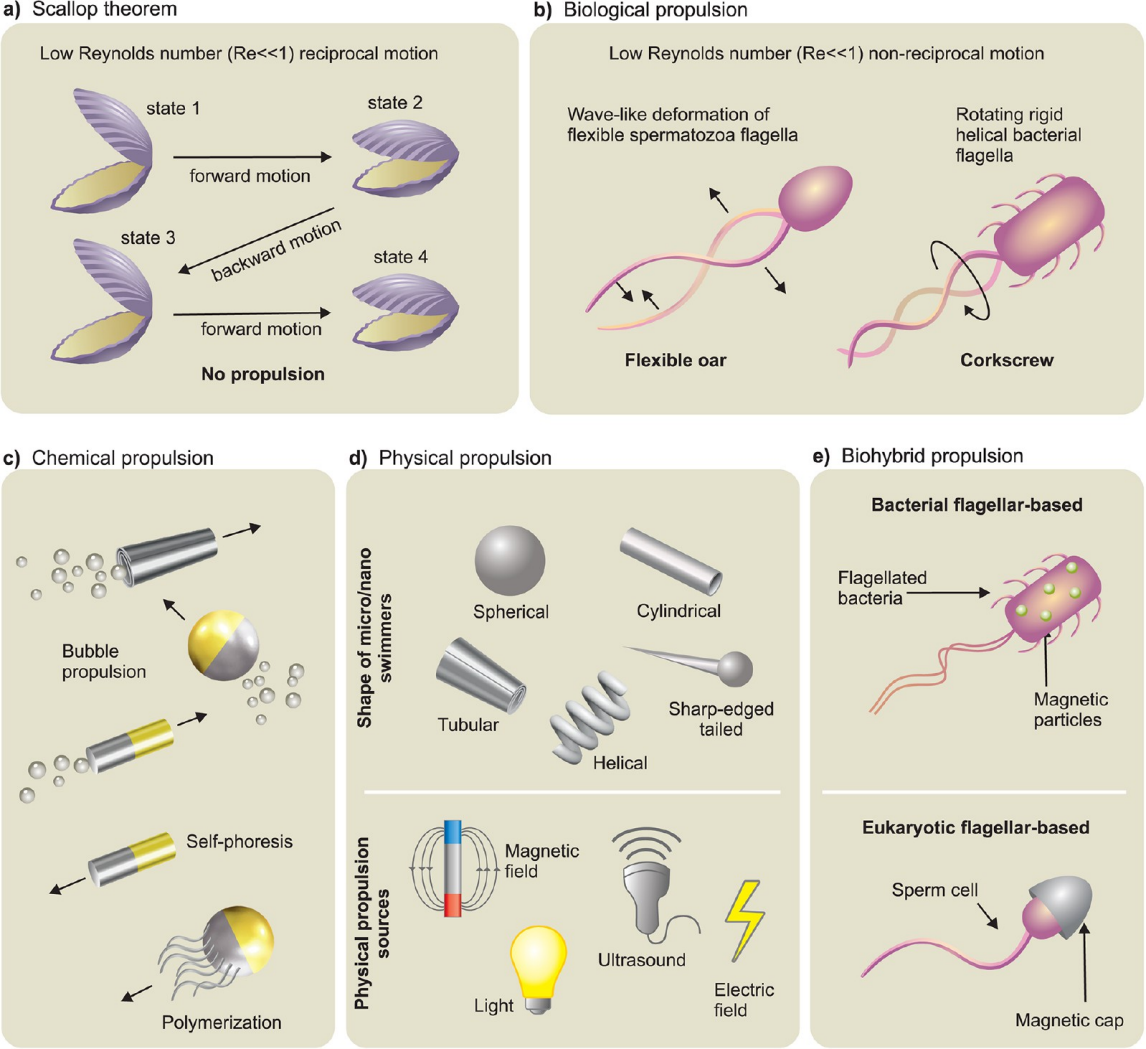
**Theory of locomotion at low Reynolds numbers and micro/nanorobot propulsion mechanisms. a)** Schematic drawing of Purcell’s “Scallop Theorem” with reciprocal motion at low Reynolds number regime. Adapted from ref 161, Copyright 1977 AIP Publishing. **b)** Two main biological propulsion mechanisms based on nonreciprocal motion at low Reynolds number regime, *i.e.*, beating a flexible oar (in eukaryotic flagellar-based propulsion) and rotating a chiral flagellum (in bacterial flagellar-based propulsion). Adapted from ref 161, Copyright 1977 AIP Publishing. **c)** Schematic overview of various chemical propulsion mechanisms utilized for different kinds of micro/nanomotors. **d)** Schematic overview of various kinds of micro/nanorobots propelled *via* external physical power sources, *i.e.*, magnetic field, acoustic field, light, and electric field. **e)** Schematic overview of biohybrid propulsion mechanisms based on prokaryotic (bacterial) and eukaryotic flagella.

### 2.1 Theory of Locomotion at Low Reynolds Number

#### 2.1.1 Equations of Motion for Fluid Flow

The incompressible flow of a simple fluid, including its response to a body moving within the fluid, can be described by the classical Navier–Stokes equations:^177^

2.1 $$\rho \left(\frac{\partial u}{\partial t}+\left(u\cdot \nabla \right)u\right)=-\nabla p+\mu \nabla^{2}u+f_{\mathrm{ext}}$$

2.2 ∇·u=0

where *ρ* is mass density, **u** is velocity field of the fluid, *t* is time, *p* is dynamic pressure, *μ* is dynamic viscosity of the fluid, and **f**_ext_ represents external forces (per unit volume), *e.g.*, gravitational forces acting on the fluid. This equation applies to Newtonian fluids, for which the viscosity is constant for all shear rates. Equation 2.1 is the statement of momentum conservation for the fluid; the terms on the left capture the acceleration (or deceleration) of the fluid elements while on the right, the term *∇p* corresponds to pressure gradients and *μ∇* ^2^ **u** represents forces due to viscous friction. Equation 2.2 is the condition of mass conservation for incompressible flows, including all those where the mass density remains constant. To gain intuition in the case of a small-scale swimmer moving in a Newtonian fluid, it is useful to re-cast Equation 2.1 in a nondimensional form. With a characteristic velocity *U* and a characteristic length *L*, and anticipating a fluid flow dominated by the action of viscosity, it is standard to define the dimensionless variables (where tildes are used to imply dimensionless quantities):

2.3 $$ũ=\frac{u}{U},\nabla ̃=L\nabla ,\frac{\partial }{\partial \mathrm{t̃}}=\frac{L}{U}\frac{\partial }{\partial t},\mathrm{p̃}=\frac{L}{\mu U}p,{\mathrm{f̃}}_{\mathrm{ext}}=\frac{L^{2}}{\mu U}f_{\mathrm{ext}}$$

The Reynolds number is a dimensionless number that compares the typical magnitude of inertial force density *f*_*i*_ = (*ρU* ^2^)/*L* to viscous forces density *f*_*v*_ = (μ*U*)/*L* ^2^, and thus is given by:

2.4 Re=fifv=(ρU2)/L(μU)/L2=ρULμ

Using eq 2.3 and eq 2.4, we can re-write the Navier–Stokes equation in a dimensionless form as follows:

2.5 $$Re\left(\frac{\partial ũ}{\partial \mathrm{t̃}}+\left(ũ\cdot \nabla ̃\right)ũ\right)=-\nabla ̃\mathrm{p̃}+{\nabla ̃}^{2}ũ+{\mathrm{f̃}}_{\mathrm{ext}}$$

Micro/nanoscale swimmers have characteristics that almost always put them in the low Reynolds number regime: length scales of tens of micrometers or less and velocities in the order of tens of micrometers per second. These values indicate Reynolds numbers around 10^–4^ in water (or typically even lower).^177^ This implies that we can neglect the left-hand side of eq 2.5, leading to the incompressible (dimensionless) Stokes equation:

2.6 $$0=-\nabla ̃\mathrm{p̃}+{\nabla ̃}^{2}ũ+{\mathrm{f̃}}_{\mathrm{ext}}$$

2.7 ∇·ũ=0

In the low Reynolds number regime, eq 2.6 implies that viscous drag forces dominate inertial forces at small scales and the equations of motion become linear and time-reversible.^178^

#### 2.1.2 Boundary Conditions

The equations of motion (eq 2.6 and eq 2.7) need to be accompanied by appropriate boundary conditions. Two types of boundary conditions are applicable for the locomotion of small-scale swimmers. The first one is the “kinematics” type where the deformation of the body is imposed and the resulting motion (*i.e.*, swimming linear and angular velocities) is solved for using the fact that the micro/nanoscale swimmers are, in general, force- and torque-free. This is often the case for theoretical modeling, which allows us to obtain analytical predictions on model systems.^179^

For the second case, typically harder to solve, the shape of the swimmer is not known but must be solved as a part of the swimming problem itself. In this case, there is a two-way coupling between kinematics (swimmer shape) and dynamics (distribution of forces and torques). This is exemplified by the swimming of spermatozoa, where the shape of the flagella is a balance between internal molecular forcing, the mechanical response of the flagella, and the external fluid flow.^180^

#### 2.1.3 Reciprocal *vs*. Nonreciprocal Motion

The disappearance of time as an explicit parameter from the Stokes equation has one important consequence for the ability of small-scale swimmers to generate propulsion: motion of their bodies and appendages that are reversible in time (so-called “reciprocal motion”) cannot generate propulsion. This is famously captured by E. M. Purcell’s “Scallop Theorem”, which states that symmetric back-and-forth motion (exemplified by the motion of scallop shells opening and closing) cannot produce propulsion on average (Figure 2a).^161^ To achieve a net translation in low Reynolds number regimes, small-scale swimmers must employ motion sequences that are not symmetric in time.

#### 2.1.4 Biological Propulsion

Biological or synthetic swimmers that self-propel using body movement must follow nonreciprocal kinematics. Because swimmers with a single degree-of-freedom are necessarily reciprocal, in his famous talk entitled “Life at Low Reynolds Numbers”, E. M. Purcell introduced the minimal nonreciprocal swimmer, consisting of three rigid links connected by two hinges, which can swim provided the hinges oscillate with a finite phase difference.^161^

More broadly, the biological world offers many examples of nonreciprocal swimming strategies (Figure 2b). One is the corkscrew mechanism of bacterial flagella where rigid helical flagellar filaments are rotated by specialized motors embedded in the cell wall. Another example is the wave-like deformation of flexible spermatozoa flagella. Less studied examples include neutrophils that exploit friction against surfaces to propel themselves, rolling along the endothelium lining the blood vasculature. In most cases, these biological examples have led to the design of bio-inspired swimmers in laboratory conditions, such as artificial bacterial flagella^16^ and artificial spermatozoa.^173^

#### 2.1.5 Locomotion *vs*. Diffusion

At small scales relevant to biological or artificial swimmers, the stochastic Brownian motion, due to the continuous collisions with atoms or molecules within the fluid, can have significant effects. The classical Einstein relation connects the diffusion coefficient *D* of a suspended particle of hydrodynamic mobility *ζ* (for simplicity, assumed to be scalar) and the mean thermal energy *k*_B_ *T* in the fluid at absolute temperature *T via*:

2.8 $$D=\zeta k_{B}T$$

where *k*_B_ is Boltzmann’s constant. In Stokes flow, the mobility *ζ* is the inverse of the drag coefficient *c_d_*. Thus, for a spherical particle of radius *a* and in a Newtonian fluid of dynamic viscosity *μ*, we have the following equation:

2.9 $$D=\frac{k_{B}T}{6\pi a\mu }$$

This diffusion constant *D* in turn controls its average mean squared displacement (in three dimensions) across multiple paths within a time interval *τ* as:

2.10 $$\left\langle r^{2}\left(\tau \right)\right\rangle =6D\tau$$

Similarly, random rotation results from Brownian torques. While for large objects, Brownian forces and torques are usually negligible, they can become important on small scales. For example, Brownian reorientation is known to affect the swimming of the smallest bacteria.^181^ Similarly, the propulsion performance of nanoscale artificial swimmers is severely affected in water due to the impact of Brownian motion.

#### 2.1.6 Locomotion in Complex Fluids

The aforementioned discussion applies only to Newtonian fluids, for which the relationship between stresses and rate of deformation is linear. On the other hand, the Scallop Theorem no longer holds in non-Newtonian fluids whose viscosity varies with shear rate. The two classical examples are shear-thinning fluids (viscosity decreases with deformation) and shear-thickening fluids (viscosity increases with deformation). The nonlinear properties of these fluids enable artificial swimmers to move more effectively using reciprocal motion. This was demonstrated experimentally using a single-hinge magnetic microscallop unable to propel in Newtonian fluids but showing propulsion in non-Newtonian fluids under asymmetric opening/closing motion.^182^ Complex fluids can also display viscoelastic behavior, some elastic properties in addition to their normal viscous response, which can in turn greatly influence the kinematics and dynamics of micro/nanoscale swimmers in a manner that depends critically on the relevant length scales in the fluid.^183^

### 2.2 Chemical Propulsion Mechanisms

Chemically propelled micro/nanomotors locomote as a consequence of chemical processes, such as a catalytic reaction or diffusion of a substance to the surrounding fluid (Figure 2c and Figure 3). Catalytic reactions can be induced just by contact of a material (catalyst) with reagent(s) or by activating the catalyst with an external stimulus (*e.g.*, light or electric field) to boost the chemical reaction. As we will see in the upcoming sections, there are several mechanisms proposed for the motion of catalytically propelled micro/nanomotors, which depend on several factors, *e.g.*, size, shape, and composition. Other strategies exploit Marangoni effects, galvanic displacement reactions, ion-exchange processes, bipolar electrochemistry, or polymerization.

**Figure 3:**
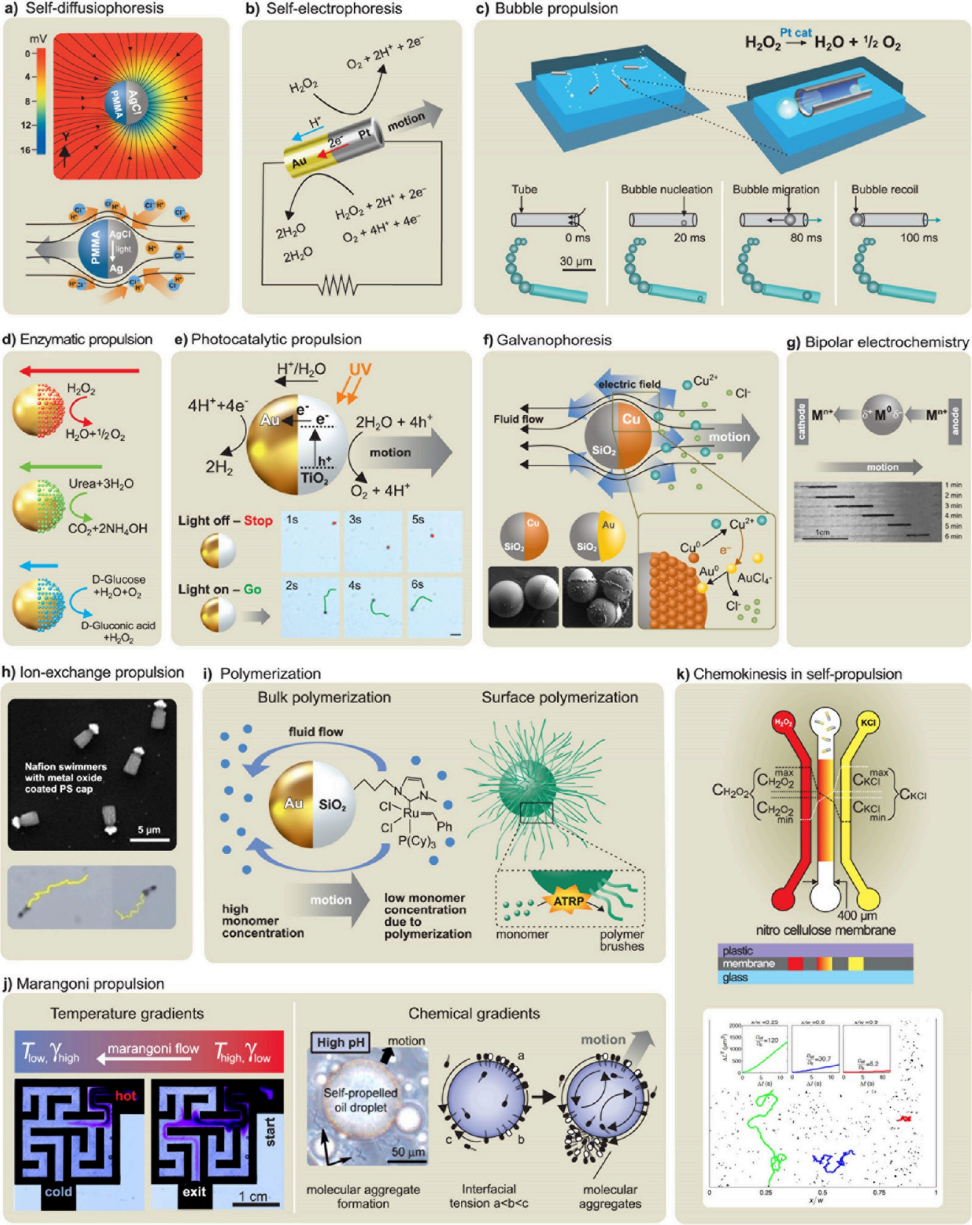
**Chemical propulsion mechanisms. a)** Self-diffusiophoresis observed in AgCl-PMMA Janus microspheres due to the photodecomposition of AgCl on the surface. Reproduced from ref 226, Copyright 2018 American Chemical Society. **b)** Self-electrophoresis of segmented Au/Pt wires with an internal electron flow from the Pt segment to the Au segment and migration of protons in the surrounding area. Reproduced from ref 26, Copyright 2004 American Chemical Society and ref 358, Copyright 2020 ELSEVIER. **c)** Bubble-propelled tubular nanojets by the generation and release of bubbles. Reproduced from ref 10, Copyright 2015 WILEY-VCH and ref 197, Copyright 2010 WILEY-VCH. **d)** Selection of enzyme-powered hollow mesoporous Janus nanomotors. Reproduced with permission under a Creative Commons CC-BY License from ref 35, Copyright 2015 American Chemical Society. **e)** Photocatalytic propulsion of TiO_2_-Au Janus micromotors powered by UV light in water, demonstrating cyclic on/off UV light activation. Reproduced from ref 74, Copyright 2015 American Chemical Society. **f)** Galvanophoresis of Cu-SiO_2_ Janus micromotors illustrating the galvanic exchange from Cu to Au caps. Reproduced from ref 292, Copyright 2021 American Chemical Society. **g)** Bipolar self-regeneration principle and propulsion of Zn micromotors in a glass tube filled with ZnSO_4_ solution under the influence of an external electrical field. Reproduced from ref 78, Copyright 2010 American Chemical Society. **h)** Nafion micromotors and their propulsion with ion-exchange mechanism. Reproduced with permission under a Creative Commons CC-BY License from ref 307, Copyright 2024 Royal Society of Chemistry. **i)** Polymerization-based propulsion due to the bulk polymerization of polymer on the SiO_2_ side of a Janus motor (left panel) and the surface polymerization of hydroxyethylmethacrylate-based polymer brushes on nanomotors (right panel). Reproduced from ref 191, Copyright 2011 WILEY-VCH and ref 310, Copyright 2021 Royal Society of Chemistry. **j)** Temperature-induced Marangoni propulsion of dye particles in a maze filled with a hot solution of a fatty acid to find the shortest path (left panel) and the directional Marangoni propulsion of the oil droplet due to the chemical gradients caused by the hydrolysis of ester-containing cationic surfactant (right panel). Reproduced from ref 328, Copyright 2015 Royal Society of Chemistry and ref 334, Copyright 2011 American Chemical Society. **k)** Chemokinesis-driven accumulation of self-propelled Pt/Au nanorods in low-mobility regions due to the fuel gradients, showing the traces of rods’ motion in high-speed, medium-speed, and low-speed (green, blue, and red, respectively) regions. Reproduced with permission under a Creative Commons CC-BY License from ref 354, Copyright 2021 Springer Nature.

Some of the pioneering micro/nanomotors contained Pt or Ni^27^ metals for the catalytic decomposition of H_2_O_2_ in one segment of the rod-based structures. Depending on the metal, different mechanisms were observed, *i.e.*, self-electrophoresis and bubble propulsion.^140,184,185^ Since the first spherical micromotors were reported in 2005, Pt has been the most widely used catalyst in the field thanks to its high catalytic performance.^22^ Afterwards, motors from tens of nanometers to a few micrometers were reported by utilizing different combinations.^186,187,188,189,190,191,192,193,194,195^ For instance, tubular micro/nanojets powered by the decomposition of H_2_O_2_ fuel generate a thrust of O_2_ bubbles asymmetrically released from the interior of the tubes. Although this type of motor presents high-speed values that can be utilized for drilling^196^ and towing^197^, the use of H_2_O_2_ as a fuel limits its application in biomedical environments. Nevertheless, the vigorous bubble release and high speeds can enable fluid mixing, leading noteworthy prospects toward proof-of-concept water remediation applications.^146,147,148,198,199^

While noble metals have been extensively used as efficient catalysts, the toxicity of the H_2_O_2_ fuel required can limit their utilization, especially in biomedical applications. Alternatively, enzymes have emerged as a biocompatible alternative with high versatility in enzyme-substrate configurations. Since the first examples of bienzymatic reactions used to power large fibers^200^ at the air-liquid interfaces to the propulsion of multi-walled carbon nanotubes,^201^ the field has grown in the pursuit of more biocompatible combinations of fuel-substrate using enzymatic reactions. Until now, catalase and urease have been the most commonly used enzymes, constituting a majority of the enzymatically powered micro/nanorobots reported.^202^ For example, catalase was used to replace Pt inside the walls of tubular microjet engines, enabling a reduction in the concentration of H_2_O_2_ fuel.^33^ Other enzymes, like collagenase,^203^ acetylcholinesterase,^153^ glucose oxidase,^35^ combinations of glucose oxidase and catalase,^204^ trypsin,^205^ and others, have been used as well as combinations of enzymes and inorganic catalysts. A comprehensive review on the different types of enzymes and the types of motors, materials, shapes, and sizes has been recently reported.^202^ Beyond inorganic materials, MOFs,^206,207^ coacervates,^208^ DNA nanotubes,^208^ and liposomes,^209^ among others, can also be used as a chassis for the motion of enzyme motors.^208,210^ Tactic phenomena were also described for urease and catalase motors^211^ and, later on, following the Hoffemeister series for urease–liposome-based nanomotors.^38^

#### 2.2.1 Self-Phoresis

In a unified framework, self-phoresis defines the locomotion of a particle driven by a self-generated driving field gradient. The driving field *Ψ* can be the concentration of species (self-diffusiophoresis), electric potential (self-electrophoresis), or temperature (self-thermophoresis). In these scenarios, the surface activity of the particle leads to the generation of a normal surface flux of the field, given by:

2.11 J=−Dn̂·∇Ψ

where *D* is the diffusivity of the field and *n̂* is the unit vector normal to the particle surface. In the limit of the thin interaction layer, the gradient of the field over the particle surface leads to the formation of a tangential slip velocity in the vicinity of the particle surface, given by:

2.12 $$v_{\mathrm{slip}}=\mu_{\mathrm{ph}}\left(I-\mathrm{n̂}\mathrm{n̂}\right)\cdot \nabla \Psi$$

where μ_ph_ is the phoretic mobility and *I* is the identity matrix. Lammert *et al*.^212^ provided a general expression for translational and rotational velocities of a self-phoretic micromotor in terms of slip velocity:

2.13 $$\left(\begin{array}{l} U\\ \Omega \end{array}\right)=-\int_{S}K\left(x_{S}\right)\cdot v_{\mathrm{slip}}dS$$

with the integral tensorial phoresis kernel given by:

2.14 $$K\left(x_{S}\right)=R^{-1}\left(\begin{array}{l} E^{†}\left(x_{S}\right)\\ G^{†}\left(x_{S}\right) \end{array}\right)$$

where *R* is the resistance matrix. The position-dependent tensors E and G , defined over the particle surface, stem from the auxiliary problem of a rigid body (*r.b*.) translation (**U**_*r.b*._) and rotation (**Ω**_*r.b*._) for the same particle, and relate the surface traction **f**(**x**_S_) exerted by particle on the fluid to the velocity of rigid body motion:

2.15 $$f\left(x_{S}\right)=E\left(x_{S}\right)\cdot U_{r.b.}+G\left(x_{S}\right)\cdot \Omega_{r.b.}$$

The kernel-based surface integration of slip velocity is a standard approach to solve phoretic velocity. However, from a design perspective, we may be interested in making a direct connection between the velocity and distribution of activity. For example, in designing electrocatalytic bimetallic micro/nanomotors and investigating the effect of geometry, we can measure the electrochemical flux from microelectrodes and are interested in its relation to the micro/nanomotor’s motion. To directly link the experimental flux measurements and distribution of the surface activity to the micromotor dynamics, Lammert *et al*.^212^ took advantage of the linearity of the governing equation to obtain a relationship between flux distribution and velocity by surface integration of the flux *J*(**x**_S_) weighted by an integration kernel for particles with uniform phoretic mobility:

2.16 $$\left(\begin{array}{l} U\\ \Omega \end{array}\right)=-\frac{\mu_{\mathrm{ph}}}{D}\int_{S}K\left(x_{S}\right)\cdot \mathrm{n̂}\mathrm{n̂}J\left(x_{S}\right)dS$$

Nourhani and Lammert^213^ had earlier obtained an expression for the phoresis kernel of a spheroid moving along its symmetry axis. The kernel depends only on particle geometry, not flux distribution, and provides some insights and design principles. For example, in rod-like geometries, the kernel value around the equator was nearly zero, making the flux contribution to motion negligible. Thus, expensive materials could be used near the poles and cheaper metal in the middle while maintaining velocity. Also, for a slender-body particle with uniform phoretic mobility, Schnitzer and Yariv obtained an approximate expression for osmotic self-propulsion in terms of the weighted integral of the surface flux.^214^

The slip velocity formalism and phoresis kernel formalism are complementary, each providing a different perspective on particle motion. Phoresis kernels illustrate how geometry defines the local contribution of the field or surface flux to particle motion, making them particularly useful for designing an individual particle with specific dynamics. The slip velocity formalism has been instrumental in studying the flow field around particles and collective dynamics. Thus, both slip velocity and phoretic kernel formalisms can be used simultaneously to address different aspects of the problem under study; one can design a particle with specific dynamics using the phoresis kernel formalism and then calculate the corresponding slip velocity to apply established frameworks for studying the flow field around the particle and collective behavior.

##### 2.2.1.1 Self-Diffusiophoresis

Diffusiophoresis refers to the transport of a particle in a solute gradient. As a recognized effect since the mid-20^th^ century, the underlying physics of diffusiophoresis is well-established both theoretically and experimentally.^215,216,217,218^ Traditionally, diffusiophoresis is discussed in the context of an externally applied solute gradient. More recently, it was found that the solute gradients generated by a particle itself can lead to self-propulsion, an effect appropriately termed “self-diffusiophoresis”.^219,220^ Just like conventional diffusiophoresis, self-diffusiophoresis can arise from the concentration gradient of ions (“electrolyte” or “ionic” self-diffusiophoresis) or neutral molecules (“nonelectrolyte” or “neutral” self-diffusiophoresis). Because ions are commonly involved in the chemical reactions that power a micro/nanomotor, we focus on ionic self-diffusiophoresis in this section.

Let us consider a typical scenario of a Janus microsphere half-coated with a metal cap. The metal cap can be chemically active, releasing a pair of ions in an aqueous fuel solution and/or under external stimuli, such as a SiO_2_-Ag microsphere in H_2_O_2_.^221,222,223^ In this case, Ag dissolves in H_2_O_2_ and releases Ag^+^ and OH^–^ (or OOH^–^) besides the Ag-catalyzed decomposition of H_2_O_2_. Alternatively, the microsphere itself can be chemically active while the inert cap partially blocks the ionic flux, such as a CaCO_3_ microsphere half-coated with an inert layer.^224^ In this case, CaCO_3_ dissolves in H_2_O and releases Ca^2+^ and CO_3_^2–^ ions, the latter further reacts with water to generate HCO_3_^–^ and OH^–^.^225^ Other examples of such Janus microspheres of asymmetric ionic release include SiO_2_-Au in a mixture of N_2_H_4_ and H_2_O_2_ ^109^ and SiO_2_-AgCl in water and under UV light.^226^

The photodecomposition of AgCl in the structure of SiO_2_-AgCl Janus microspheres serves as an example of how this surface reaction and the resulting ionic gradient leads to directional motion *via* ionic self-diffusiophoresis (Figure 3a) (a more detailed description is given in ref 226). In this case, AgCl is believed to photo-decompose into Ag^+^, H^+^, and Cl^–^. The latter two ions diffuse from the coated surface of a Janus microsphere into the surrounding aqueous solution. Because H^+^ diffuses significantly faster than Cl^–^ (9.311 × 10^–9^ m^2^/s *vs*. 2.032 × 10^–9^ m^2^/s), an electric field pointing toward the AgCl cap emerges to speed up Cl^–^ and slows down H^+^.^227^ This electric field then pushes the ions in the diffuse layer on the surface of the microsphere and generates an electro-osmotic flow along its surface. Because the particle is negatively charged, the resulting electro-osmotic slip flow moves from the AgCl to the SiO_2_ hemisphere and the particle moves in the opposite way with the AgCl cap leading.

This process is only the electrophoretic component of ionic diffusiophoresis (or self-diffusiophoresis) and requires the ions to diffuse at different rates. On the other hand, the ions also interact with the colloidal surface and any difference in the interaction potential between the different ions can lead to the directional transport of colloids (and self-propulsion for self-generated ionic gradient). This is known as the chemiphoretic component of ionic diffusiophoresis (or self-diffusiophoresis). Combined, the speed of a micro/nanomotor powered by both the electrophoretic and the chemiphoretic components of ionic diffusiophoresis is given by (at a small Zeta potential limit):^228^

2.17 $$\mu =\frac{\varepsilon }{4\pi \eta }{\left(\frac{k_{B}T}{Ze}\right)}^{2}\left[\beta \mathrm{\zeta ̅}\left(1-3\lambda \right)+\frac{1}{8}{\mathrm{\zeta ̅}}^{2}\left(1-\frac{21}{2}\lambda \right)\right]$$

where *ε* and *η* are the electrical permittivity and viscosity of the solution, respectively, *k*_B_ *T* is thermal energy, *Z* is charge valence, and *e* is the elementary charge. In addition, *ζ̅* = *Zeζ*/*k*_B_ *T* where ζ is the Zeta potential of the motor (in practice, it is often the average Zeta potential of a Janus particle). The difference in the diffusivity between the cation and the anion is represented by:

$$\beta =[\left(D_{+}-D_{-}\right)/\left(D_{+}+D_{-})\right]$$ 2.18

Finally, *λ* = (*κa*)^−1^, where *κ* is the Debye length and *a* is the particle radius. Note that the chemiphoretic contribution of ionic self-diffusiophoresis is assumed to be small, and therefore, often ignored. However, one can see that this is only applicable for a pair of ions of a large difference in diffusivity (*i.e.*, large *β*), and the electrophoretic component vanishes for *β* = 0. For example, for SiO_2_-AgCl (3 μm, −40 mV) moving in an electrolyte (1 μM), the relative contribution from the electrophoretic to the chemiphoretic component is roughly 1.2 according to eq 2.17, with each oppositely moving the particle. Other choices of parameters can easily produce a scenario where chemiphoretic forces dominate. Such situations, and the resulting reversal of a chemical micromotor, were discussed previously.^228^

Although the reactive cap is consumed for the example of AgCl micromotors, there are also other ways to generate ions. Plenty of examples are found in biology, where some enzymes efficiently convert substrate molecules into ionic products. Urease is an example of such enzymes commonly used for constructing catalytic micro/nanomotors that operate *via* ionic self-diffusiophoresis.^35,229,230^ Urease catalyzes the conversion of urea into NH_3_ and CO_2_, which then reacts with water to produce NH_4_^+^, HCO_3_^–^, OH^–^, and a small amount of CO_3_^2–^. Interested readers are directed to excellent review articles such as refs 11, 231, 232, 233 and other sections of this review for more references on micro/nanomotors powered by enzymes.

Finally, we briefly comment on neutral self-diffusiophoresis. As a straightforward idea, a Janus microsphere can move directionally if its surface interacts sufficiently strongly with a neutral molecule in its concentration gradient, arising from surface reactions. Over the years, many micro/nanomotors have been proposed to move *via* this mechanism: microspheres half-coated with Pt moving in H_2_O_2_,^22,234^ SiO_2_-Au Janus micromotors functionalized with the Grubbs catalyst moving in norbornene,^191^ liposomes moving in β-cyclodextrin,^235^ motors coated with catalase^35^ or glucose-oxidase^236^ in H_2_O_2_ and glucose, *etc*. However, it remains controversial if these motors are truly powered by neutral self-diffusiophoresis. This controversy is primarily rooted in the lack of two critical pieces of information: 1) if any ionic species are involved in the reactions and 2) the microscopic details and accurate measurements of how the neutral molecules interact with the colloidal particles. For an expanded discussion on this controversy about neutral self-diffusiophoresis, see a recent perspective article.^237^

##### 2.2.1.2 Self-Electrophoresis

Self-electrophoresis, also known as “auto-electrophoresis”, is a mechanism by which micro/nanomotors propel themselves *via* the generation of electric fields. The motors typically possess a charged surface, which can interact with the electrolytic medium in which they swim, forming a layer of counterions on the surface, known as the electrical double layer (EDL). The exchange of ions between this layer and the particle, together with the established gradient, allows the propulsion of the micro/nanomotors, typically driven by electrochemical reactions occurring at the surface.^219,238^

The decomposition of H_2_O_2_ has been routinely used to drive bimetallic Pt/Au micromotors, with generated protons diffusing through the medium and electrons being conducted through the rod from Pt to Au.^26,239,240,241^ At the Au end of the rod, a separate H_2_O_2_ decomposition reaction occurs, consuming protons and electrons to generate water from the fuel. In this situation, the rod acts like a circuit, with the flux of current generated across its surface leading to an inhomogeneous distribution of ions that generates an electric field, driving the motion of the micromotor with the Pt moving forward (Figure 3b).^242^ Indeed, when rods are fabricated from other combinations of metals, including Au, Ni, Pd, Pt, Rh, and Ru, the metal acting as the anode will typically be at the front of the motile micro/nanomotors.^184^

Alternatively, self-electrophoretically driven Janus micromotors containing semiconductor–metal junctions (*e.g.*, TiO_2_ with Pt, Au, Ag, Fe, or Cu) have been reported, where electrons generated on the TiO_2_ side of the motor migrate to the metal side while protons are similarly generated at the TiO_2_ surface and then consumed at the metal surface *via* reduction reactions. This leads to the propulsion of the motors with the TiO_2_ end moving forwards.^74,243,244,245^ In these cases, because the generation of anions and cations at the motor surface is typically achieved using photoelectrochemical reactions, on/off control over the propulsion behavior may be achieved using light as an external trigger, which may enhance the suitability of these motors for targeting applications.

Importantly, in contrast to motors propelled *via* diffusiophoresis, self-electrophoretic motors may not cause a net increase in ions over time, as one end of the motor generates the ions and the other consumes them, consequently acting as an ion source and an ion sink. Since motors driven by either type of propulsion respond negatively to increasing bulk ionic strength, this means that self-electrophoretically propelled micro/nanomotors can maintain almost constant speed over time regardless of population density, whilst the speed of diffusiophoretic-driven micro/nanomotors decreases as population density increases, potentially representing a significant advantage of electrophoretic-driven micro/nanomotors.^246^ In addition to the background ionic strength, the speed of electrophoretically driven micro/nanomotors is influenced by the concentration of the fuel in the solution, with a linear relationship at low to moderate concentrations and more complex behavior observed at higher concentrations due to the formation of bubbles causing variability in the motion.^219^ The “leveling off” of speed at high concentrations is also believed to arise from the saturation of reaction sites on the catalytic surface, which eventually becomes the limiting factor.^247^ For photocatalytically induced propulsion, increasing the light intensity can increase the speed of the motion by increasing the production of the ions^245,248,74^ while the geometry can also be modified to vary the rate of motion, with both the shape and the distribution of materials forming the device playing key roles.^213,240^

Indeed, by modifying the design of such micro/nanomotors beyond simple spheres or rods, more advanced control over their propulsion may be achieved. For example, Dai *et al*. demonstrated how a Janus TiO_2_/Si nanotree structure, with TiO_2_ nanowires acting as a photoanode and Si nanowires as a photocathode, could propel *via* self-electrophoresis only when illuminated with 365 nm UV light.^107^ Alternatively, by choosing a TiO_2_-Fe semiconductor metal combination for their spherical micromotor, Wang *et al*. were able to use external magnetic fields to control the direction of the self-electrophoretically propelled micromotor.^249^ Meanwhile, by coating Si nanowires with polyelectrolytes, the tolerance of the motor to high ion concentrations could be improved, increasing the feasibility for use in bodily fluids, such as blood.^250^

##### 2.2.1.3 Self-Thermophoresis

Thermophoresis describes the directed motion of ions, molecules, and colloidal particles in response to a temperature gradient. The thermophoretic velocity, **v**_*T*_ = −*D_T_* ∇*T*, is proportional to the temperature gradient ∇*T*, with *D_T_* being the thermophoretic mobility. When a temperature difference is established, solutes immersed in a fluid experience forces that drive them from hotter to colder regions or vice versa, depending on their interactions with the solvent. In colloidal systems, this movement is primarily explained by the temperature gradient inducing a fluid slip velocity at the particle’s surface. This slip velocity results from the different interactions between solute and solvent molecules at the interface, creating asymmetric interfacial forces that generate a net force, propelling the particle. The magnitude and direction of thermophoretic transport are determined by the interactions between the colloidal particle and the solvent.^251^ Self-thermophoresis refers to the special case in which the colloidal particle autonomously generates its own temperature gradient, leading to self-propulsion in the liquid. This can typically occur when a colloidal particle, such as a Janus particle, is partially coated with a material that absorbs light^252^ or undergoes an exothermic chemical reaction,^253^ producing a localized temperature difference around the particle. The induced thermal asymmetry leads to a slip velocity at the particle surface, which propels it through the liquid without the need for an externally applied temperature gradient. Self-thermophoresis is an important mechanism in the study of artificial micro/nanorobots, as it enables controlled motion at microscopic scales, with applications in targeted drug delivery, microscale transport, and synthetic active matter.

Many theoretical and experimental studies have explored micro/nanorobots or active colloids driven by self-thermophoretic forces. From the theoretical point of view, Kapral and collaborators have derived Langevin equations for the dynamics of metal-based Janus motors under radiation fields using fluctuating hydrodynamics and radiative heat transfer theory.^254^ Ripoll’s group has actively worked on self-thermophoretic Janus-based motors, using simulations and theoretical analysis to study self-propelled Janus particles and nano/microdimers in solutions, with the latter exhibiting thermally induced puller/pusher characteristics.^255–258^ Other studies have focused on the effect of fluid-colloid interactions on the mobility of thermophoretic microswimmers through numerical simulations^259^ or have developed an analytical framework to determine the self-induced thermophoretic velocity of laser-heated Janus metamaterial microparticles, deriving explicit expressions for thermophoretic hydrodynamics, while providing practical estimates for self-propulsion based on key physical parameters.^260^

From an experimental perspective, numerous studies have explored self-thermophoresis using light to induce thermal gradients. Sano and colleagues conducted experimental studies on Au-based Janus particles, demonstrating active motion via self-thermophoresis in a defocused laser beam. They were the first to measure both the temperature distribution and the thermal slip flow field around a micrometer-sized Janus particle.^261^ He’s group has been highly active in studying self-thermophoretic motors under near-infrared (NIR) light, investigating various designs, including Janus mesoporous silica/Au motors, Janus microcapsules with a gold-coated surface on one side, polymeric tubular motors functionalized with Au nanoshells, and needle-like liquid Ga nanoswimmers, among others.^252,262–267^ Similarly, asymmetric porous and hollow carbon-based nanoparticles have been developed as fuel-free nanomotors, propelled by NIR-light-driven self-thermophoresis.^268^ Additionally, magnetically induced thermophoretic locomotion has been demonstrated in permalloy-capped Janus motors, enabling precise control over their motion.^269^

Beyond pure thermophoretic motion, researchers have engineered nanomaterials that integrate self-thermophoresis with other propulsion mechanisms to enhance locomotion. Examples include C/Mn-based nanomotors that combine H_2_O_2_-driven self-diffusiophoresis with NIR-induced self-thermophoresis;^270^ hybrid Janus enzyme-modified silica/carbon@Pt nanomotors powered by H_2_O_2_-induced oxygen gradients, NIR-driven self-thermophoresis, and enzyme-driven self-diffusiophoresis;^271^ and light-driven ZnO/Au nanomotors that couple self-electrophoresis with self-thermophoresis.^272^

#### 2.2.2 Bubble Propulsion

Since the invention of self-propelled micro/nanomotors, bubble propulsion has been a research focus with several advantages, such as higher speeds and enhanced mass transfer. In this way, bubble-propelled micromotors indicate promising characteristics, especially for environmental remediation. A strategy of Pt/H_2_O_2_ combination to provide mechanical power opens the gate of bubble-propelled nanomotors, where the catalyzed H_2_O_2_ decomposition and momentum change *via* O_2_ bubbles lead to the autonomous locomotion of catalytic nanomotors.^194^

Among the catalytic motors, self-propelled tubular microrobots pioneered by Mei *et al*.^28^ can be particularly attractive for practical biomedical applications. Pumera’s group also presented a rapid fabrication method for nanojets, using a template-directed electrochemical deposition method where the bubble-ejecting nanojets were grown within Al_2_O_3_ templates.^273^ In addition to many metals, the combination of polyaniline, polypyrrole, or poly­(3,4-ethylenedioxythiophene) with Pt can be used for the development of catalytic microjets.^155^ To better explain the motion mechanism of nanojets, researchers divided their motion into three stages (Figure 3c). In the first stage, the fuel solution wets the catalytic material containing energetically favorable nucleation points, where O_2_ accumulates and expands as bubbles. In the second stage, bubbles migrate toward one opening of the tube—normally the larger opening—and finally, the bubbles are released, thereby inducing another motion step.^10,197^

In addition, research efforts to replace toxic H_2_O_2_ fuel have provided different alternatives. For example, Zn, Al, or Mg can react with acids or water to produce hydrogen (H_2_) bubbles, leading to active locomotion. Enzymatic reactions also bring new insights into the possibility of using nontoxic fuels to obtain the locomotion of micro/nanomotors. Therefore, the integration of mesoporous SiO_2_ and enzymes has led to another type of enzyme-powered nanomotors. For example, Ma’s group fabricated self-propelled Janus nanomotors based on hollow mesoporous SiO_2_ nanoparticles, which are powered by biocatalytic reactions of three different enzymes: catalase, urease, and glucose oxidase (Figure 3d). Overall, the enzyme-powered nanomotors presented above bring more inspiration toward the development of bubble-propelled motors.^35^

#### 2.2.3 Photocatalytic Propulsion

Photocatalytic propulsion is a process that involves the use of light to propel micro/nanomotors, converting both chemical and light energy into motion.^8,64^ This process usually incorporates: 1) light absorption, 2) electron-hole separation, 3) redox reactions, and 4) product generation, generating propulsion through self-electrophoresis, self-diffusiophoresis, or bubble propulsion.^9^ A key metric of photocatalytic micro/nanorobots is operation wavelength, which is determined by the photon energy required to excite an electron from the valence band to the conduction band. Based on the bandgap (E_gap_) of applied semiconductor materials, photocatalytic micro/nanorobots can be designed to be activated in ultraviolet, visible, or near-infrared regions.^274^

To construct photocatalytic micro/nanomotors, either a mono-component or multi-component scheme can be adopted.^65^ Mono-component micro/nanomotors are usually based on simple semiconducting particles, such as AgCl, TiO_2_, Fe_2_O_3_, C_3_N_4_, and BiVO_4_, which manifest advantages in facile and mass production.^66–69,275^ However, this type of micro/nanomotors is often limited by the high recombination rates of electron-hole pairs as well as insufficient overall efficiency. To address this challenge, multi-component schemes are proposed based on metal-semiconductor or semiconductor–semiconductor junctions. Upon contact with the metal, the difference between the electron affinity of the semiconductor and the work function of the metal results in a Schottky barrier, facilitating electron-hole separation and photocatalytic efficiency. For instance, a variety of Janus micro/nanomotors have been designed with such principles, including but not limited to TiO_2_/Au, TiO_2_/Pt, Cu_2_O/Au, BiOI/Au, ZnO/Pt, C_3_N_4_/Pt, and Si/Au.^74,245,251,276–279^ In general, their higher photocatalytic efficiency results in enhanced performance in low light intensity and even pure water environments (Figure 3e). Moreover, the fabrication of hybrid photocatalytic/magnetic micro/nanorobots has demonstrated enhanced motion speeds due to improved electron-hole separation. These enhancements are attributed to the synergistic effects of magnetic spinning combined with photocatalytic motion,^280^ as well as charge transfer mechanisms induced by the external magnetic field.^281^

A lower electron-hole recombination rate can also be accomplished by constructing semiconductor–semiconductor p-n junctions.^70,282–286^ Such photocatalytic micro/nanomotors could be regarded as a device with cascaded photovoltaic and electrochemical cells.^287^ The performance of a photovoltaic cell is described by the following equation:

2.19 $$j=j_{L}-j_{s}\left[e^{\frac{qV}{nk_{B}T}}-1\right]$$

where *j* is the output current density, *j*_s_ is reverse saturation current density, *q* is electronic charge (1.602 × 10^–19^ C), *n* is the ideality factor, *k*_B_ is Boltzmann’s constant (1.38 × 10^–23^ J K^–1^), *V* is the solar cell open circuit voltage, *j*_L_ is photogenerated current density, and *T* is temperature. The photovoltage *V* can be further divided into three potentials:

2.20 $$V=h_{c}+h_{a}+h_{\mathrm{solution}}$$

where *h*_solution_ is the solution potential drop, *h*_c_ and *h*_a_ are the overpotential at the cathode and anode, respectively. The overpotentials can be described by an electrochemical kinetics equation containing both electrochemistry polarization and concentration polarization:

2.21 $$h_{c}=\frac{RT}{\alpha F}\ln\left(\frac{j_{c}}{2j_{O}^{0}}+\sqrt{{\left(\frac{j_{c}}{2j_{O}^{0}}\right)}^{2}+1}\right)+\frac{RT}{\alpha F}\ln\left(\frac{j_{\mathrm{dc}}}{j_{\mathrm{dc}}-j_{c}}\right)$$

2.22 $$h_{a}=\frac{RT}{\beta F}\ln\left(\frac{j_{a}}{2j_{R}^{0}}+\sqrt{{\left(\frac{j_{a}}{2j_{R}^{0}}\right)}^{2}+1}\right)+\frac{RT}{\beta F}\ln\left(\frac{j_{\mathrm{da}}}{j_{\mathrm{da}}-j_{a}}\right)$$

where *R* is molar gas constant, *T* is absolute temperature, *F* is Faraday constant, *j*_c_ is cathode current density, *j*_a_ is anode current density, *j*_O_^0^ is cathode exchange current density, *j*_R_^0^ is the anode exchange current density, *j*_dc_ is the cathode diffusion-limited current density, *j*_da_ is the anode diffusion-limited current density, and *a* and *b* are cathodic and anodic transfer coefficients, respectively. Equations 2.19–2.22 provide an outline to analyze the photovoltaic and electrochemical processes involved in heterojunction-based photocatalytic micro/nanomotors. The enhanced propulsion of micro/nanomotors could be achieved by optimizing photovoltaic efficiency as well as the kinetic constant and diffusion coefficient of redox chemicals.^287^

#### 2.2.4 Galvanophoresis

Nobel Laureate Peter Mitchell was fascinated by the microscale mechanisms involved in the energy system of microorganisms, which leads to all the processes that define life, including motility.^288^ Extending the principle of his chemiosmotic theory of electron gradients across membranes to microorganisms, he postulated an ion-gradient-driven swimming mechanism for bacteria.^289^ Despite several attempts, experimental evidence for this swimming mechanism in microorganisms has never been found. Later, this mechanism was elaborated at a more fundamental level in the theory of phoresis^218,290^ and Golestanian proposed a model for the self-propulsion of spheres.^176^ However, these models mostly relate to the motion of colloids in very diluted electrolyte gradients, resulting in thin Debye layers.^291^ To improve the realistic description of colloids releasing ions, a thicker Debye layer and a strong coupling between the ion flux/solute transport and the charge balance were introduced into the model by DeCorato and co-workers.^291^ This results in a completely different motion mechanism from the standard phoretic models, which depends entirely on the surface flux of the ions instead of the solute–surface interactions. It considers specific properties, such as different diffusion coefficients of the ionic species. These lead to qualitatively different behavior, which has been demonstrated using an experimental system based on enzyme-grafted colloids. A full understanding of the mechanism of nonequilibrium movement at high electrolyte concentrations has also been hampered by the fact that enzyme-driven systems are dominated by experimental imperfections and biological variability.

A better model system to study this novel swimming mechanism can be galvanophoresis, a motion strategy based on electromotive forces. In electrochemistry, electromotive forces refer to the maximum potential difference between two electrodes of an electrochemical cell, or the tendency of an element to gain or lose electrons. If we define a Janus particle as an electrochemical cell consisting of a less noble anode deposited as a hemisphere on the inert particle, then the tendency of the (dissolved) noble metal ions to be reduced to the pure metal on the micromotor leads to the dissolution of the cap and release of the corresponding ions. The surface flux of these ions creates currents that can lead to active propulsion. For the example of a Cu-based Janus particle in an Au-based HAuCl_4_ solution (Figure 3f), we can formulate the following half-cell reactions, where positive potential indicates a spontaneous reaction:

$$\begin{array}{l} 3\mathrm{Cu}\to 3{\mathrm{Cu}}^{2+}+6e^{-}\\ 2H^{+}+2{{\mathrm{AuCl}}_{4}}^{-}+6e^{-}\to 2\mathrm{Au}+8{\mathrm{Cl}}^{-}+2H^{+} \end{array}$$

The overall reaction is as follows:

$$3\mathrm{Cu}+2{\mathrm{HAuCl}}_{4}\to 2\mathrm{Au}+3{\mathrm{CuCl}}_{2}+2\mathrm{HCl}$$

While electrochemistry requires the use of the Nernst equation to calculate the electromotive forces and the Gibbs free energy (Δ*G*) for the actual process by calculating the difference of the standard potentials, it is not yet clear if and how this value relates to the driving force of the process:

2.23 $$\Delta G_{\mathrm{free}}=-\mathrm{zF}\left(E_{\mathrm{cathode}}-E_{\mathrm{anode}}\right)=-\mathrm{zFE}$$

where *z* is the number of electrons exchanged, *F* is the Faraday constant, and *E* is the potential difference of the reaction. We find a dependence on concentration using the Nernst equation; however, it is also overlaid by the absolute potential, reflecting the tendencies of different metal combinations to undergo reduction and oxidation reactions. An additional influence is created by the different behavior of individual ions; using the example from above, positively charged Cu ions accumulate near the negatively charged colloid surface while the negatively charged Cl ions diffuse away. Taking into account the different diffusivity of Cu and Cl ions,^292^ we find a resulting diffusion potential that creates an electric field, resulting in a fluid flow that leads to the locomotion of particles. Whether a self-electrophoretic mechanism^293^ with self-diffusiophoretic contributions^292^ or a strongly coupled ion flux/solute transport model best describes this type of propulsion is currently under investigation.^292a^

#### 2.2.5 Bipolar Electrochemistry

Bipolar electrochemistry deals with (semi)­conducting objects on which oxidation and reduction reactions occur simultaneously at spatially distinct locations. The so-called “bipolar” electrode does not need to be connected to a power supply because the driving force of the overall reaction is provided by the polarization of the object with respect to the solution potential. In turn, the solution potential is governed by an electric field imposed by two feeder electrodes.^294^ As the conducting object is by definition equipotential, the potential gradient in the surrounding solution will lead to a gradually changing potential difference between the solution and the object along its main axis, oriented parallel with respect to the electric field lines. If this polarization is strong enough, then one extremity of the object will be able to drive oxidation processes, whereas the opposite extremity will be the site of reduction reactions. The resulting inhomogeneous reactivity intrinsically constitutes a straightforward strategy for breaking the symmetry in electrochemical systems. Consequently, bipolar electrochemistry can be considered as an attractive mechanism to induce controlled propulsion of micro/nanorobots. This concept was reported in the 2010s, and horizontal motion based on two different mechanisms was described around this time.

The first case corresponds to a bipolar metal electrode made out of a Zn dendrite that is positioned inside a capillary filled with an aqueous ZnSO_4_ solution (Figure 3g).^78^ The application of only a modest electric field is sufficient to overcome the small overpotentials needed to promote the anodic dissolution of Zn together with Zn deposition on the cathodic pole. Finally, the dendrite translates continuously toward the feeder anode. From a conceptual point of view, one can argue whether the observed translocation can be considered a real motion, as the Zn dendrite continuously changes its shape and length during its movement. In fact, the phenomenon might be regarded as the propagation of a chemical wave, driven by an electric field. In principle, such a strategy can be potentially applied to a variety of non-noble metal particles; later, an analog concept was used for the propulsion of a sacrificial Cu bipolar electrode in folded fluid channels.^295^

The second proposed mechanism involves the site-selective production of gas bubbles. The evolution of either H_2_ or O_2_ can be used for this purpose but, practically, H_2_ bubbles are more efficient due to the stoichiometric advantage of this reaction.^79^ The locomotion of C beads of several sizes has been achieved by applying an increasing driving force when downsizing the characteristic dimensions of the object. Vertical motion was also reported by placing a conducting bead inside a capillary filled with an aqueous solution.^296^ The idea is to select the polarity of the feeder electrodes to generate a cathodic reaction site at the bottom of the bead and, therefore, to produce H_2_ gas below the object. The bubbles can readily accumulate and lift the bead in the direction of the external cathode, which is positioned at the top of the capillary. Initially, the anodic reaction converted sacrificial chemical species, but one can select advantageously another specific oxidation reaction to obtain an additional level of functionality. This was demonstrated with anodic electrogenerated chemiluminescence that can be recorded *in situ* while the bead is moving inside the capillary.^297,298^ Following a similar strategy, a second generation of bipolar motors has been designed, featuring a set of conducting arms combined with a small reservoir enabling the collection and controlled release of the electrogenerated bubbles.^299^ This leads to an object that can be successfully actuated in 2D space. Additionally, in this work, the integration of an LED was proposed to allow wireless electronic light emission during their motion. In a more general context of triggering motion at the macro- and microscale, bipolar electrochemistry can also be employed to address objects made of conducting polymers, leading to site-selective swelling or shrinking, which can consequently lead to controlled locomotion.^300,301^

#### 2.2.6 Ion-exchange Propulsion

Noncatalytic nanomaterials, such as polymeric ionomers, can have the capability to store ions within the polymeric network and exchange them at the interface as a potential mechanism to propel micromotors. An example of such polymeric ionomers is Nafion, a polymer with high mechanical, thermal, and chemical stability, made of a hydrophobic tetrafluoroethylene backbone with hydrophilic sulfonic acid pendant groups.^302^ This structure creates a porous network in which water penetrates, facilitating ion transport and ion exchange. Other examples of ion exchange polymeric materials with similar capabilities are sulfonated polystyrene microparticles and sulfonated polystyrene-divinylbenzene copolymer microstructures.^303,304^

During the ion-exchange process, a chemical gradient is established, generating a local electric field. This field arises due to the unequal diffusion coefficients of the exchanged ions. For the case of Nafion, this electric field has been quantified and has been demonstrated to point perpendicularly toward the Nafion structure when the polymer is initially loaded with protons.^302^ This is also applicable to other polymeric structures with ion-exchange capabilities.

The chemical gradients generate an ion current governed by the Nernst–Planck equation:

2.24 $$j_{i}=-D_{i}\nabla n_{i}+\frac{en_{i}z_{i}}{k_{B}T}D_{i}E+n_{i}v$$

where the terms on the right-hand side represent the diffusion (due to concentration gradient), migration (due to an electric field **E**), and convection (due to a fluid velocity **v**) contributions, respectively. *D_i_* and *n_i_* stand for the diffusion coefficient and concentration of ion *i*, *z_i_* is the ion valence, e is the elementary charge, *k*_B_ is Boltzmann’s constant, and *T* is temperature. Because the ion exchange does not generate any net electric current **J**_*e*_, the imposition of the condition **J**_*e*_ = ∑_*i*_ *ez_i_* **j**_*i*_ = 0 generates an electric field:

2.25 $$E=\frac{k_{B}T}{e}\frac{\sum_{i}z_{i}D_{i}\nabla n_{i}}{\sum_{i}z_{i}^{2}D_{i}n_{i}}$$

Interestingly, the electric field, apart from being inversely proportional to the ion concentration, is also directly proportional to the gradient of ion concentration. Therefore, ion-exchange micromotors can work at high salt concentrations because the screening effect of the electric field at higher salt concentrations is compensated by the fact that the salt itself is the fuel generating the electric field and driving motion.^305^ This is in contrast to electrophoretic or ionic diffusiophoretic micromotors discussed above, in which the addition of salts drastically screens the electric field and annihilates the interfacial fluid flow, halting the locomotion of micromotors.

By patterning or nanostructuring the surface of the polymeric material, one can generate tangential components of the electric field, which can trigger interfacial fluid flows. These systems act as pumps (the counterparts of motors) sharing the same operational principles.^306^ By engineering such pump platforms and the surrounding Zeta potentials, one can manipulate fluid flows from radial to unidirectional, opening new possibilities in the area of wireless micro/nanofluidic networks.^305^ All this knowledge and its combination with simulations are crucial for the design of ion-exchange-based micromotors.

One requirement to design efficient micromotors is to introduce compositional or shape asymmetry to the polymeric structure to ensure asymmetric chemical gradients and activate tangential fluid flows and phoretic mechanisms. Numerical simulations have shown that a nanorod made entirely of an ion-exchange polymer only generates symmetric recirculating fluid flows at its surface, which cannot produce a net propelling velocity when suspended in a fluid. However, when capped with another passive material (*i.e.*, not capable of ion exchange), the symmetry of the system breaks down, inducing a net tangential fluid flow that allows the motion of the structure. These results have led to the design of Nafion micromotors capable of self-propelling autonomously in a bulk fluid (Figure 3h).^307^ Indeed, Nafion micromotors with collective behavior signatures have been developed, offering high versatility to control their motion direction and strength depending on the capped structure and without requiring the proximity or presence of walls to move.^307^ Under the same context, interesting studies have been pursued using ion-exchange spherical microparticles based on sulfonated polystyrene–divinylbenzene block polymers.^304^ In this case, motion is triggered when these particles form complexes with surrounding passive particles, creating asymmetric modular complexes without being attached to each other. None of the constituents exhibit active swimming by themselves. In this case, the modular motion was explained by the electric field generated by the ion-exchange resin, which induces an electro-osmotic solvent flow on the charged substrate or walls of the cell containing the particle dispersion. Recently, another study addressed the use of sulfonated polystyrene beads as ion exchangers.^303^ An individual symmetric sulfonated polystyrene microbead is mechanically passive as it cannot self-propel without breaking the symmetry. However, as a chemically active ion exchanger, it generates osmotic flow around itself along the substrate, leading to mechanical interactions with other sulfonated polystyrene microbeads in a crowded environment and creating mechanical motion at the ensemble level.

#### 2.2.7 Polymerization/Depolymerization

Polymerization and depolymerization processes can facilitate locomotion across a wide range of biological systems, from single-celled organisms to complex multicellular structures. The dynamic and reversible nature of the polymerization process enables these organisms to respond rapidly to environmental stimuli and perform various forms of locomotion. For instance, in mammalian cells, actin polymerization is crucial for cell motility. *Listeria monocytogenes* is a pathogenic bacterium that can hijack this mechanism to exhibit actin-based motility within and between host mammalian cells. Based on this idea, Cameron *et al*. used polystyrene beads coated with an actin-polymerizing protein ActA to induce motion.^308^ As other examples from nature, the rapid movements of a carnivorous plant (*Venus flytrap*) and a sensitive plant (*Mimosa pudica*) involve changes facilitated by the polymerization and depolymerization of components in the cell wall.

Inspired by natural organisms, the concept of (de)­polymerization has been suggested as a mechanism to obtain the motion of micro/nanomotors. On the other hand, despite the ubiquity of polymers as a building block of micro/nanomotors, (de)­polymerization has been significantly less explored compared to other motion mechanisms.^309^ The first synthetic polymerization-powered micromotors were reported by Sen’s group in 2011 using the ring-opening metathesis polymerization of Norbornene.^191^ These micromotors were based on Au-SiO_2_ Janus particles, chemically modified with the Grubbs’ catalyst on the Si side (Figure 3i left panel). The authors attributed the enhanced diffusion of micromotors to the asymmetrically positioned Grubbs’ catalyst that consumed numerous monomer molecules while forming only a limited number of polymer chains. Therefore, these micromotors were able to establish the required monomer gradient for fluid low from the side with lower substrate concentration (Si side) to the side with higher substrate concentration (Au side). Approximately 10 years later, Städler’s group followed up on this idea, using SiO_2_-based micromotors that were surface-functionalized with an atom-transfer radical polymerization initiator for surface polymerization of hydroxyethylmethacrylate into polymer brushes (Figure 3i right panel).^310^ In addition to enhanced diffusion during the polymerization process, a high-density population of micromotors exhibited wave-like motion as an indicator of the presence of swarming behavior.

Complementary efforts explored the use of depolymerization to induce locomotion. This concept was initially reported for micropumps where the first effort considered films made from tert-butyldimethylsilyl end-capped poly­(phthalaldehyde), which depolymerizes in response to fluoride.^311^ The released monomers accumulated near the film, which results in increased osmotic pressure, leading to a directed bulk water flow. This idea was only recently translated to induce the locomotion of micromotors. Specifically, SiO_2_ particles were coated with a self-immolative polymer that could be triggered to disintegrate in a domino-like fashion when exposed to albumin.^312^ It could be argued that the mobility of motors driven by the disintegration of self-immolative polymers is limited due to the fast diffusion times of the monomers that resulted in only short-lived monomer gradients in the proximity of the motors. Therefore, Städler’s group explored the locomotion of motors when entire polymer chains detached from the surface of the motors. In particular, the pH-triggered disintegration of polymer multilayers deposited on the surface of the motors^313^ and the pH-triggered detachment of PEG chains from the motors^313^ resulted in enhanced locomotion with speed of 3–4 μm s^–1^ in both cases. Taken together, these few efforts do not sufficiently elucidate the relation between polymer degradation kinetics and resulting motor speeds. This implies the requirement of further investigations to evaluate if the disintegration of synthetic polymers is a competitive concept to induce locomotion.

In nature, the “burnt bridges” mechanism involves a molecule, like an enzyme, moving along and degrading a substrate, preventing backward movement. This ensures unidirectional motion, as seen in nucleases degrading DNA or collagenases breaking down collagen. It is therefore an interesting question to evaluate the motion characteristics of motors equipped with these depolymerizing enzymes. In contrast to the previous examples, the motor’s environment is depolymerized, acting as fuel for the motors. In this context, Städler’s group developed motors that can locomote in collagen-based environments by decorating SiO_2_ particles with collagenase, which degrades collagen or gelatin into small fragments upon calcium activation. The motion mechanism, a burnt-bridge Brownian ratchet, relied on collagen degradation to propel the motors. Evaluating these collagenase-based motors in photo-crosslinked gelatin-based hydrogels of varying densities revealed that the motors moved in long, straight trajectories within the Ca^2+^ gradient.^203^ The trajectory length decreased as gelatin concentration increased, with top speeds of ∼30 μm s^–1^ in 0.1 wt% photo-crosslinked gelatin. To imitate the extracellular matrix more closely, collagen fiber networks were explored as the environment for motors made of 500 nm polystyrene particles decorated with collagenase.^314^ In this case, speeds up to ∼30 μm s^–1^ in low-density fiber networks were observed, confirming effective motion in these fibrous environments. Finally, the collagenase-based motors were evaluated in the extracellular matrix using 3D cell spheroids of SAOS-2 cells.^315^ Collagenase motors incubated with these spheroids and exposed to Ca^2+^ showed a three-fold increase in motor penetration compared to control groups. Additionally, motors decorated with magnetic nanoparticles delivered heat *via* magnetic hyperthermia, reducing cell viability to ∼60–70% compared to > 90% in controls. In summary, collagenase-powered motors are effective in navigating viscous, fibrous media and complex biological environments like the extracellular matrix in 3D cell constructs, making them relevant for biomedical applications. Overall, the use of (de)­polymerization as a tool to induce locomotion is successfully illustrated by nature and scientists have started to imitate this concept, obtaining motors with a broad range of speeds.

#### 2.2.8 Marangoni Propulsion

Marangoni flow occurs due to a gradient in surface or interfacial tension between two phases. Surface or interfacial tension gradients can arise from various sources, including gradients in temperature or gradients in chemical compositions along the surface. The area with higher interfacial tension has a larger pulling force than the low interfacial tension area, leading to a corresponding tangential stress to be balanced by the viscous stress, resulting in a flow. The stress and flow are denoted as Marangoni stress and Marangoni flow, respectively, and can be used to induce the motion of macroscale and microscale motors.^316,317,318^ In daily life, the Marangoni effect can be easily observed at the macroscopic scale, such as in the “tears of wine” phenomenon^319^ and “the camphor boat demonstration”.^320^ At millimeter and submillimeter length scales, the Marangoni effect enables the manipulation of objects along a fluid interface^321,322^ and spontaneous motility of artificial colloidal motors.^323,324,325,326^

The characteristics of motion (*e.g.*, speed, directionality) driven by the Marangoni effect change with the size and shape of the motor and the surrounding environment. The speed of Marangoni flow (*u*) is related to the change in interfacial tension (Δ*γ*) and viscosity (*μ*):

2.26 $$u\approx \frac{\Delta \gamma }{\mu }$$

The Marangoni number *M_a_* is a dimensionless quantity that is often used to compare the rate of Marangoni flow versus the rate of diffusion:

2.27 $$M_{a}=\frac{uL}{D}=\frac{\Delta \gamma L}{\mu D}$$

where the relevant length scale of the motor (*i.e.*, object to be moved) is *L* and the diffusion rate is *D*. For small *M_a_*, diffusion dominates, and Marangoni flow becomes trivial. On the other hand, for large *M_a_*, Marangoni flow occurs and can be used to drive the motion.

Because the surface tension of a fluid changes with temperature (surface tension typically decreases with increasing temperature), objects can also be induced to move directionally using a thermally induced Marangoni effect.^327^ For example, Lovass *et al*. demonstrated maze-solving by particles with the help of thermal gradients (Figure 3j left panel).^328^ In this study, a centimeter-scale maze was filled with a hot solution, and then a temperature gradient was created by placing a cold metal sphere at the maze exit. Dye particles added to the maze entrance were driven by Marangoni flow to the maze exit and followed the shortest continuous path because it had the steepest thermal gradient. Besides applying a direct temperature gradient, other stimuli, such as light, can be integrated to generate thermal gradients (*i.e.*, a photothermal effect), that in turn leads to the Marangoni effect.^329,330^ For instance, Rybalko *et al*. directed oil droplet motion by heating part of the droplet *via* a laser beam, where an interfacial tension difference led to a convective flow.^329^

Additionally, gradients of chemicals at an interface can be used to induce interfacial tension gradients and generate Marangoni flows. There are many ways that chemical gradients can arise, such as localized reactions, interfacial transport across phases, evaporation from a mixture, or external application. In general, surfactant molecules are used in some manners while exploiting Marangoni propulsion because surfactants are surface-active and effective at changing surface tension. Chemical processes occurring at or near the interface of the motor that results in an interfacial tension difference are of particular interest as this can lead to spontaneous self-generated locomotion.

One prominent example of chemically driven Marangoni propulsion is in the case of self-propelled or “swimming” droplets.^323,324,325,326^ Such droplets can be either oil-in-water or water-in-oil. Self-propulsion of an isotropic droplet that lacks geometric asymmetry can still occur under certain conditions where nonlinear solute transport dynamics contribute to spontaneous symmetry breaking.^331^ Usually, Marangoni flows are created *via* two mechanisms: chemical reactions or interfacial transport (like solubilization). In the case of chemical reactions, at least one reagent is normally dispersed inside the droplet to induce reactions at or near the interface (these reactions often involve degrading or creating surfactant molecules). Marangoni flow is thus generated according to the chemical gradient-induced interfacial tension gradient (typically, the side of the drop with the more surfactant molecules has the lowest interfacial tension) (Figure 3j right panel). Advective transport of reactants helps sustain the gradient across the surface outside the motor. A series of reactions have been designed to create active, swimming droplets, including hydrolysis reactions,^332^ redox reactions,^333^ pH-sensitive reactions,^334^ and others.^335,336^ For example, Thutupalli *et al*. fabricated water-in-oil active droplets by dispersing bromine water in a continuous squalane oil phase containing monoolein as a surfactant.^333^ Droplets were induced to move by the spontaneous bromination of the surfactant at the drop interface, which weakened the surfactant activity. Micellar solubilization is another common mechanism to create the Marangoni flows needed to generate active droplets.^337,338^ During solubilization, the droplet contents are transferred into surfactant micelles (self-assembled aggregates of surfactants) to create gradients in the continuous fluid phase. It is not completely understood how solubilization (either the products of it or the process itself)^337^ alters the interfacial tension, although one mechanism may be a solubilization-induced lowering of the critical micelle concentration.

#### 2.2.9 Manipulation with Chemical Propulsion

Many motile cells and single-celled microorganisms demonstrate chemotaxis, which refers to directed motion up or down a gradient in chemical concentration. Chemotaxis can allow bacteria to seek nutrients and avoid predators,^339^ facilitates immune cells’ pursuit of those same bacteria,^340^ and enables spermatozoa to find and fertilize an ovum, among others.^341^

Inspired in part by this utility, several researchers have aimed to realize chemotaxis in artificial active matter systems (Figure 3k). This is not trivial because artificial micro/nanomotors lack sensing, signaling, and motion redirection capabilities that motile cells use to perform chemotaxis. Nevertheless, experimental evidence for chemotaxis has been presented in a few studies, for example with active droplets^342^ and enzymatic micromotors, which are popular model systems for artificial chemotaxis^35,38,204,343^ as reviewed in ref 344. It should be noted that the physics underlying enzyme-driven locomotion in the first place, let alone its ability to execute true chemotaxis, remains the subject of ongoing research and debate.^345,346^

A related phenomenon to chemotaxis is chemokinesis, which refers to a dependence of translational speed (regardless of direction) on the local concentration of a chemical immediately surrounding the swimmer.^347^ Like chemotaxis, chemokinesis is observed in nature and can lead to nonuniform accumulation of cells over time.^347,348,349,350^ As summarized by Wilkinson,^351^ chemotaxis implies a vector response to a chemical field (as it implies a redirection of motion) and chemokinesis implies a scalar response to a chemical field (as it is concerned only with speed and is agnostic regarding direction). Chemokinesis can be positive or negative, depending on whether the propulsive speed is positively or negatively correlated with an increase in chemical concentration. If speed increases or decreases with increasing concentration, then the chemokinetic response is said to be positive or negative, respectively.

Chemokinesis is common in artificial active particle systems, such as those powered by catalytic decomposition of H_2_O_2_ or enzymatic motors that use the enzyme’s substrate as a fuel (though the speed typically saturates at high substrate concentration). For example, self-propelled Pt/Au rods (first demonstrated in 2004)^26^ exhibit a positive chemokinetic response to H_2_O_2_ (speed increases with H_2_O_2_ concentration) and a negative chemokinetic response to electrolytes such as sodium nitrate or lithium nitrate (speed decreases with increasing electrolyte concentration), as first demonstrated in 2006.^352^ A notable exception is any salt containing silver (Ag), which tend to accelerate the rods’ motion as a positive chemokinetic response.^140^

However, chemokinesis alone does not imply chemotaxis. Popescu *et al*. analyzed Janus particles that self-propel due to an asymmetry in surface chemistry and/or properties.^353^ Using theoretical arguments, they found that (to first order), a phoretic Janus particle’s tendency to rotate in a chemical gradient (proportional to its chemotactic response) depends on the mismatch in phoretic mobility between its halves. In contrast, its overall translational speed (its chemokinetic response) depends on the average mobility of the two halves. Physically, a particle that exhibits pure chemokinesis should tend to dwell in low-mobility regions for long periods because in high-mobility regions, they will by definition move more quickly and thus quickly escape these regions.

To test this idea experimentally, Moran *et al*. considered an ensemble of Pt/Au self-propelled nanorods in steady-state antiparallel concentration gradients of H_2_O_2_ and potassium chloride (KCl), to which the rods exhibit a positive and negative chemokinetic response, respectively (Figure 3k).^354^ Theory, Brownian dynamics simulations, and experiments demonstrated that over time, the rods accumulated in the low-speed regions (high KCl, low H_2_O_2_) of the motility gradient. This result is the opposite of what one would expect from positive chemotaxis (which had been previously claimed to occur for various chemokinetic micromotors, including the Pt/Au nanorods). The steeper the gradient in motility, the more asymmetric the accumulation. This work demonstrated that chemokinesis alone, *i.e.*, the mere fact that speed depends on local concentration does not lead to chemotactic behavior.

It is important not to mistake chemokinesis-driven accumulation for negative chemotaxis. Crucially, the theoretical and computational models of Moran *et al*.^354^ did not assume any “intentionality” on the part of the Pt/Au rods, meaning they did not assume that the rods would autonomously reorient themselves toward lower H_2_O_2_ concentration (which would be required for negative chemotaxis). The agreement between theory and experiment demonstrates that accumulation can be achieved in a motility gradient, even when no directional preference is assumed.

Although realizing chemotaxis in an artificial microscale active matter system is not trivial, chemokinesis-driven accumulation could have practical applications. If an active colloidal system is designed to slow down in regions where accumulation is desired, then one can still realize the same asymmetric swimmer accumulation that one would expect from chemotaxis. In this vein, Archer *et al*. demonstrated swellable hydrogel-based H_2_O_2_-powered microrobots that exhibit a pH-dependent speed.^355^ The microrobots are fabricated from poly­(2-vinyl pyridine) (PVP), a hydrogel that swells in response to pH decreases. As pH decreases, the PVP is protonated, leading to mutual electrostatic repulsion and swelling. At the same time, a model cargo (Rhodamine dye) was also released, implying that these particles can simultaneously slow down their motion and release their cargo preferentially in the most acidic regions of a region containing a nonuniform pH distribution. This strategy shows significant therapeutic potential; for example, in many biomedical scenarios, the most acidic regions often correspond to the areas where the therapeutic payload is most needed. Examples include the core of a tumor^356^ or the interior of a bacterial biofilm.^357^

## 2.3 Physical Propulsion Mechanisms

Apart from chemical propulsion mechanisms, micro/nanorobots can be actuated by externally applied fields. In this section, we cover propulsion mechanisms based on magnetic fields, ultrasound, light, and electric fields.

### 2.3.1 Magnetic Fields

#### 2.3.1.1 Types of Magnetic Materials

Depending on their response to applied magnetic fields, materials can be categorized as ferromagnetic, paramagnetic, diamagnetic, antiferromagnetic, and ferrimagnetic.^359,360^ These materials have been utilized in varying extents as components in small-scale robots, with ferromagnetic materials being the most employed. In the following paragraphs, a brief overview of each type is provided. For expanded explanations on these types of materials, the reader is referred to previous reviews and references.^361,362^

Ferromagnetic materials are composed of atoms or ions with a net magnetic moment due to the spins of unpaired electrons. These magnetic moments tend to align parallel to each other within small regions, called “domains”, resulting in a net spontaneous magnetization. If the magnetic material has not been previously subject to a magnetic field, then a ferromagnetic material typically consists of magnetic domains, where the collective sum of magnetic moments across all domains amounts to zero. Because of the presence of domains, ferromagnetic materials display hysteresis behavior, which implies that they retain a certain degree of magnetization even after the removal of the external magnetic field. The magnetic field strength required to reduce the magnetization of a ferromagnetic material to zero after it has been magnetized to saturation is known as “coercivity”. The values of coercivity and remanent magnetization are indeed used to further classify ferromagnetic materials into hard- and soft-ferromagnets. Hard-ferromagnets typically display high coercivity and high remanent magnetization. They require a significant external magnetic field to demagnetize and retain a strong magnetic field even after the external field is removed. This feature allows the ability to program hard-magnetic bodies to align in specific directions.^363,364^ Ferromagnets are characterized by high magnetic susceptibility, typically on the order of 10^3^–10^6^. Conversely, soft-ferromagnets have low coercivity and lower remanent magnetization. Magnetic susceptibility *χ* is defined as the ratio of the magnetization **M** of the material to the applied magnetic field intensity **H**:

2.28 $$\chi =\frac{M}{H}$$

This dimensionless number indicates how easily a material can be magnetized. Soft-magnets usually display higher magnetic susceptibility than hard-magnets.^361^ This property is key as soft magnets become more strongly magnetized in response to lower magnetic field strengths than hard magnets. Examples of ferromagnets are Co, Ni, Fe, and their alloys.^365^ One can find many examples of magnetic microrobots manufactured using these materials in the form of full-bodied metallic structures, polymer composites, or thin films integrated with a nonmagnetic chassis.^366,367,368,369^

Ferrimagnets are magnetic materials where atoms possess net magnetic moments, but a fraction of these moments is oriented oppositely to the remainder (this type of arrangement is known as “antiferromagnetism”). However, one population of atoms typically exhibits a higher overall magnetic moment magnitude compared to the other. Consequently, ferrimagnetic materials are structured into domains with a net magnetic moment. These materials essentially behave as ferromagnets and they can exhibit either soft or hard magnetic behavior. Among ferrimagnets, we find iron oxides (*e.g.*, magnetite and maghemite), ferrites (*e.g.*, zinc ferrite, cobalt ferrite), and some garnets (*e.g.*, yttrium iron garnet).^370,371^ These materials (usually in powder form) have been used in the structure of micro/nanorobots either combined with polymers as composites, attached to the surface of nonmagnetic bodies or as reconfigurable swarms.^365,372,373^ In a few examples, oxides can also be integrated as thin films.^373,374^

In paramagnetic materials, atoms possess individual magnetic moments; however, these are randomly distributed throughout the material’s structure, resulting in a net magnetization of zero. Although they can be magnetized when exposed to magnetic fields, their susceptibility is much lower compared to ferro- and ferrimagnets, typically on the order of 10^–5^–10^–3^. Paramagnetic materials do not exhibit magnetic hysteresis behavior and do not retain magnetization once the magnetic field is removed. Examples of paramagnetic materials include Al, Ti, W, and several oxides and sulfides of transition metals. A class of paramagnetic compounds includes those that exhibit superparamagnetic behavior. This phenomenon emerges when ferrimagnetic or ferromagnetic materials are processed into nanoparticles. At very small sizes, these nanoparticles display a single magnetic domain and their magnetic moment can flip. As a result, superparamagnetic nanomaterials display neither net magnetization nor remanence in the absence of a magnetic field. While these characteristics are typical of paramagnetic compounds, susceptibilities and saturation magnetizations of superparamagnets are similar or even higher than those of ferro- and ferrimagnetic bulk counterparts. Superparamagnetic nanoparticles have found application in various small-scale swimmer architectures, primarily as components in polymer composites.^375,376^

Diamagnetic materials are those in which atoms do not possess a net magnetization. However, when exposed to magnetic fields, they become magnetized in the opposite direction to the applied field.^377^ Consequently, their susceptibilities are negative, causing them to be repelled by magnetic fields. The susceptibility is typically on the order of ∼10^–9^ to ∼10^–5^. Examples of diamagnetic materials can be water, pyrolytic carbon, graphite, nylon, and some metals (Bi, Cu, Ag). Examples from the literature have investigated the possibility of using diamagnetic structures as levitating magnets for micro/nanorobotic applications or their manipulation in fluids. For instance, Llorente and co-workers have shown that diamagnetic pyrolytic graphite microflakes can be effectively transported in diamagnetic solutions in 3D.^378^

Antiferromagnetic materials are characterized by atoms with intrinsic magnetic moments that align in an antiparallel arrangement, effectively canceling each other out. This results in a net magnetic moment of zero in the absence of an external magnetic field.

Antiferromagnets behave as paramagnets when subjected to a magnetic field. Synthetic antiferromagnets can also be obtained by arranging alternating layers of ferromagnetic materials with nonmagnetic layers. This technique produces structures with high susceptibility and saturation magnetization while minimizing or suppressing remanent magnetization in the absence of a magnetic field. As an example, Wang *et al*. realized magnetically responsive nanoscavengers for water remediation by building artificial antiferromagnetic multilayered disk-shaped nanoparticles.^379^

#### 2.3.1.2 Magnetic Manipulation Principles

Magnetic fields exert torque *t* and force *f* on magnetic objects and, depending on their magnetic properties and geometry, different locomotion mechanisms can be attained. When a magnetic object with a magnetic moment **m** interacts with a magnetic field **B**, it tends to reorient itself to minimize the magnetic potential energy (*E* = −**m**·**B**). This reorientation can manifest as displacement or rotation of the object as it seeks to align its magnetic moment with the direction of the magnetic field. When the object experiences a translation, there is a magnetic force given by:

2.29 f=−∇E=(m·∇)B

This equation tells us that magnetic forces can only arise if a magnetic field gradient exists. If the magnetic object rotates by a certain angle **θ**, then there will be a torque **t** that causes the magnetic moment **m** to align with the field:

2.30 $$t=\frac{-\partial E}{\partial \theta }=m\times B$$

This equation implies that once the magnetic moment of the object is aligned with the magnetic field, the magnetic torque vanishes. Two primary sources contribute to the magnetic field **B**. The first source is the **H** field, generated by the circulation of electrical currents within coils. The second source is the magnetization **M**, which arises from magnetic materials (*e.g.*, hard magnets). Hence, in a vacuum media:

2.31 $$B=\mu_{0}\left(H+M\right)$$

where *μ_0_* is the magnetic permeability of the vacuum. Taking into account eq 2.28, one can express the **B** field as:

2.32 $$B=\mu_{0}\left(H+\chi H\right)=\mu_{0}\left(1+\chi \right)H$$

By defining the relative permeability μ = μ_0_ (1 + *χ*), we can express eq 2.32 as follows:

2.33 B=μH

Note that the susceptibility of paramagnets and diamagnets is very small (|*χ*| ≪ 1); hence, in these cases:

2.34 $$B=\mu_{0}H$$

When manipulating magnetic objects, it is essential to consider how the material will align with respect to the applied magnetic field.^380^ In other words, to control robots using magnetic fields, it is crucial to understand the magnetic anisotropy of a given magnetic architecture. The easy axis of magnetization, which is dictated by the anisotropy, will align parallel to the direction of the applied magnetic field.^5^ This easy axis is primarily influenced by the shape anisotropy of the material, though, in some cases, it can also be determined by the material’s crystal anisotropy.^381^

The shape anisotropy is influenced by the geometrical features of the material as the generated demagnetizing fields, once a magnetic field is applied, differ from the direction within a magnetic object.^46^ The demagnetizing fields are a result of magnetic charges that distribute at the surfaces and interfaces of magnetic objects. For example, in a rod-like structure, the demagnetizing fields are weaker along the long axis compared to the traverse directions as the surface charge density is lower along the length.^5^ Crystalline anisotropy arises from the preferential orientations of the crystal lattice, where certain directions or planes within the crystal structure favor magnetic coupling and alignments. This results in varying magnetic properties depending on the orientation of the magnetic field relative to the crystal axes or planes.^382^ For more detailed information on the magnetic methods used in robotics, the reader is referred to ref 381.

#### 2.3.1.3 Manipulation with Magnetic Field Gradients

The direct manipulation of magnetic objects or microparticles through magnetic field gradients allows for precise control of their motion, enabling targeted movement in specific directions. This method has been used for targeted delivery of chemotherapy to cancer sites using a passive magnet placed on tumors. Magnetic gradient pulling can levitate objects in the air at the millimeter and larger size scales,^383^ but stable levitation requires feedback and gets much more difficult at small scales due to the fast dynamics of microscale objects.

Field generation systems for the creation of large magnetic field gradients benefit from coils that are very near to the workspace.^384^ In the extreme, magnetic tweezers^385^ use electromagnets, sharpened to a millimeter-scale tip, inserted directly into the workspace to generate extremely high gradients of Teslas per meter (Figure 4a). Systems that can create gradients throughout the human body can generate tens to hundreds of milli-Tesla per meter with a sophisticated level of control (Figure 4b).^386^ Magnetic systems using large permanent magnets can also generate magnetic fields, which are modulated by moving or rotating the magnets for sophisticated control.

**Figure 4:**
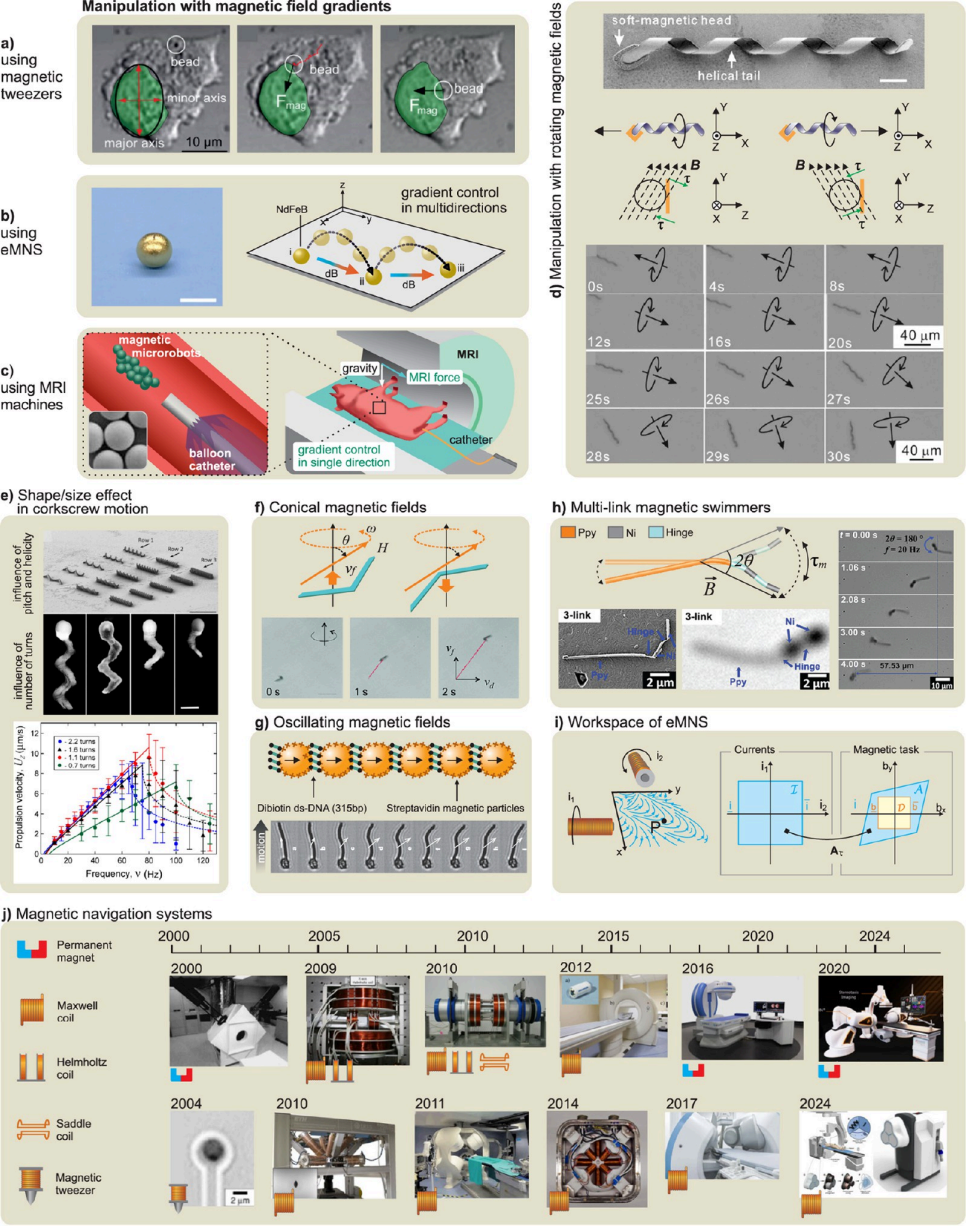
**Physical propulsion mechanisms based on magnetic fields. a–c)** Manipulation with magnetic field gradients: **a)** Magnetic bead navigated using a magnetic tweezer inside a cell to perform mechanical characterization. Reproduced from ref 385, Copyright 2019 American Association for the Advancement of Science. **b)** Au-coated NdFeB spherical magnets externally controlled *via* magnetic field gradients generated by an electromagnetic navigation system (eMNS). The scale bar is 2 mm. Reproduced from ref 386, Copyright 2024 WILEY-VCH. **c)** *In vivo* microrobot navigation using gradients of MRI to steer the aggregate into the target vessels where the hepatic flow was partially reduced by inflating a balloon catheter. Reproduced from ref 388, Copyright 2019 American Association for the Advancement of Science. **d–h)** Manipulation with (d,e) rotating, (f) conical, and (g,h) oscillating magnetic fields. **d)** Untethered artificial bacterial flagella (ABF) swimming controlled by rotating magnetic fields. Optical images indicate forward, backward, and turning motion of an ABF steered by magnetic fields. Reproduced from ref 389, Copyright 2009 AIP Publishing. **e)** Effects of shape and size in corkscrew motion: ABFs having different shapes, *i.e.*, helical actuators (Row 1), single twist-type actuators (Row 2), and double twist-type actuators (Row 3). Scale bars are 50 μm. Reproduced from ref 393, Copyright 2014 WILEY-VCH. Helical nanopropellers having different lengths, *i.e.*, 2.2, 1.6, 1.1, and 0.7 full helical turns (left to right, scale bars are 500 nm), showing different relations between their propulsion speed *vs*. rotation frequency of the external magnetic field. Reproduced from ref 400, Copyright 2015 American Chemical Society. **f)** Right- and left-handed 2D swimmers in a precessing field (field strength H, angular velocity ω, precession angle θ) presented with their swimming directions and the propulsion under the effect of conical magnetic fields. Reproduced from ref 405, Copyright 2018 WILEY-VCH. **g)** Artificially segmented swimmer fabricated from magnetic particles that are attached with double-stranded DNA *via* specific biotin–streptavidin interaction and its motion under oscillating magnetic fields (filament length = 24 μm). Reproduced from ref 173, Copyright 2005 Springer Nature. **h)** Multi-link magnetic swimmer comprising an elastic eukaryote-like polypyrrole tail and rigid magnetic Ni links connected by flexible polymer bilayer hinges and its propulsion under the influence of oscillating magnetic fields (2θ = 180° and *f* = 20 Hz). Reproduced from ref 416, Copyright 2015 American Chemical Society. **i)** Illustration of two-coil planar eMNS and its workspace that is defined as the set of positions (*e.g.*, at point P) in space where a desired set of tasks is feasible given a set of admissible currents. Reproduced from ref 432, Copyright 2023 IEEE. **j)** Magnetic navigation systems: Magnetic stereotaxis system (MSS), 2000 − Reproduced from ref 429, Copyright 2000 American Association of Neurological Surgeons; Magnetic tweezer, 2004 − Reproduced from ref 433, Copyright 2004 IEEE; Helmholtz/Maxwell setup, 2009 − Reproduced from ref 434, Copyright 2009 IOP Publishing; OctoMag, 2010 − Reproduced from ref 380, Copyright 2010 IEEE; Electromagnetic actuation (EMA) system, 2010 − Reproduced from ref 437, Copyright 2010 ELSEVIER; Catheter Guidance Control and Imaging (CGCI), 2011 − Reproduced with permission under a Creative Commons CC-BY License from ref 435; Magnetically guided capsule endoscopy (MGCE), 2012 − Reproduced from ref 436, Copyright 2012 IEEE; Minimag, 2014 − Reproduced from ref 438, Copyright 2014 Springer Nature; NaviCam, 2016 − Reproduced from ref 439, Copyright 2016 ELSEVIER; Aeon Phocus, 2017 − Reproduced from ref 440, Copyright 2017 IEEE; Genesis stereotaxis, 2020 − Reproduced from ref 441, Copyright 2020 Springer Nature; NAVION, 2024 − Reproduced from ref 386, Copyright 2024 WILEY-VCH.

MRI machines can generate field gradients in a controlled manner for microrobot motion over the entire human body, sequenced with imaging.^387^ However, MRI systems are limited in gradient magnitude without custom system hardware. Large gradients present around the outside of an MRI machine can be used for micro/nanorobot manipulation by moving the patient relative to the system (Figure 4c).^388^

#### 2.3.1.4 Manipulation with Rotating, Oscillating, and Conical Magnetic Fields

Traditional techniques of magnetic manipulation are generally based on applying gradient fields to generate a force for steering magnetic micro/nanorobots. An alternative innovative approach^51,389^ relies on actuation by a uniform in (*xy*) plane rotating magnetic field **B**_rot_ = *B*(***x̂*** cos *ωt* + ***ŷ*** sin *ωt*) that generates a magnetic torque resulting in micro/nanorobots turning about the field rotation z-axis. Given that the robot’s morphology admits nontrivial rotation-translation viscous coupling (*e.g.*, helix), the torque-driven actuation can result in net propulsion (Figure 4d). Although the field gradient can steer an isotropic (*i.e.*, spherical) particle, strong magnetic fields are required to generate sufficient field variation over the particle size, whereas torque-driven propulsion relies on weak uniform fields. Simple scaling arguments^390^ suggest that the ratio of the propulsion velocity *U* by the rotating field to the pulling velocity *V* by the field gradient reads:

2.35 $$\frac{U}{V}∼Ch\frac{B}{l\nabla B}$$

where *Ch* is the dimensionless chirality coefficient and *l* is the characteristic length of the object. *Ch* is ≲ 0.4 for helices, while the maximal values of the field gradient *∇B* in modern MRI devices is approximately 10^3^ Oe/cm. Therefore, for a micrometer-sized object, the ratio is *U*/*V*∼1 already for quite low magnitude of the rotating field (*B*∼1 Oe).

Torque-driven magnetic actuation provides a precise, fuel-free, and engine-free method for remotely steering micro/nanorobots, operating independently of boundary proximity or mutual interactions. Various sophisticated methods, such as “top-down” approaches,^389^ delamination of magnetic stripes,^391^ glancing angle deposition,^51^ direct laser writing,^109^ bio-templated synthesis,^392^ two-photon polymerizations of a curable magnetic polymer composite,^393^ spiraling microfluidic flow lithography^394^ and other techniques have been developed for fabrication of micrometer- and submicrometer-sized^174^ helical motors (Figures 4d and Figure 4e).

Theoretical research of torque-driven actuation by the rotating fields started about a decade ago. The angular dynamics of a slender helix with remanent magnetization driven by a rotating field **B**_rot_ in a Newtonian fluid was first considered in refs 390, 395 showing two synchronous (*i.e.*, in-sync with the field) rotation regimes dependent on the field frequency *ω*: (i) low-frequency tumbling, where the helix’s long axis rotates in the plane of the field; (ii) high-frequency wobbling, where the object’s long axis rotates about the field-rotation axis with a precession (or wobbling) angle *θ* < *π*/2 that diminishes with frequency as sin *θ*∼*ω* ^–1^. At the step-out frequency, the magnetic torque can no longer counterbalance the viscous friction and the synchronous rotation switches to asynchronous twirling, where the average angular velocity of the propeller diminishes fast with *ω*. The dimensionless propulsion speed along the field rotation *z*-axis in the wobbling regime has the form:

2.36 $$\frac{U_{z}}{\omega l}=Ch\left[1-{\left(\frac{\omega_{0}\cos\Phi }{\omega }\right)}^{2}\right]$$

where *Φ* is the angle between the magnetic moment **m** and the helical axis, *ω*_0_ = *mBF*_⊥_ is a characteristic (magneto–viscous) frequency defined with transverse rotational mobility *F*_⊥_ and *Ch* = *G*_∥_/(*lF*_∥_) is the chirality coefficient, defined with the longitudinal (*i.e.*, rotation about the helical axis^390^) coupling *G*_∥_ and rotational *F*_∥_ mobilities, respectively. The propulsion velocity *U_z_* increases quasi-linearly with *ω*, attaining its maximum at the step-out, where the wobbling angle *θ* reaches its minimal value. The preferable magnetization orientation is transverse to the helical axis (*i.e.*, *Φ* = *π*/2). Propulsion in the tumbling regime is typically negligible and beyond the step-out the propulsion speed drops rapidly with frequency.^390^

A similar approach was applied to analyze the dynamics of magnetizable (*i.e.*, superparamagnetic) slender microhelices.^396^ Such propellers possess no remanent magnetization and their instantaneously acquired magnetic moment by the driving field is determined by the magnetic susceptibility of the material, geometry of the propeller, and its orientation. In comparison to ferromagnetic micro/nanorobots, superparamagnetic propellers do not exhibit aggregation induced by mutual magnetic interactions in the absence of the field, and they can be fabricated using a variety of methods.^392,393,394,397,398^ The dynamics of the elongated magnetizable microhelix are similar to its magnetic counterpart; however, its magnetization is not known *a priori* and should be determined self-consistently. As a result, there is an extra constraint on the steerability parameter *γ* = *p*tan *Φ*, where *Φ* is the angle the magnetic easy-axis forms with the helical (long) axis, *p* = *F*_∥_/*F*_⊥_ is the ratio of rotational mobilities. The emergence of tumbling-to-wobbling transition and, hence, net propulsion, requires *γ* > 1. Assuming uniaxial magnetic anisotropy, it was predicted that slender microhelices with a small helix angle (*Θ* < Θ_*_) magnetize along their long axis (*i.e.*, *Φ* ≈ 0°) and are thus poor propellers. In contrast, tight microhelices (*Θ* > Θ_*_) magnetize transversely (*i.e.*, *Φ* ≈ 90°) and can efficiently propel, as confirmed in experiments.^398^ The critical helix angle Θ_*_ depends on the geometry; for a circular cross-section of the filament it is Θ_*_ ≈ 55°. In a later study,^399^ the *ad hoc* assumption of uniaxial magnetic anisotropy was relaxed, and the full magnetic susceptibility matrix was computed from first principles upon averaging over the dipole-dipole interactions among magnetic nanoparticles embedded in the polymer matrix (see ref 393). The resultant (generally biaxial) magnetic anisotropy was found to be controlled by the geometry, i.e., by the helix angle, filament cross-section shape, and orientation.

Another important question concerns the optimal geometry of magnetic microhelices. To display an efficient rotation–translation coupling with high propulsion speed, there should be enough helical turns. On the other hand, long multi-turn helices exhibit reduced step-out frequency due to high viscous friction. It can be shown that for a prescribed amount of the deposited magnetic material, the maximum propulsion speed (*i.e.*, at the step-out, in body-length per unit time) is attained roughly for a single-turn helical propeller (Figure 4e).^400^

Although the dynamics of helical micro/nanorobots are well understood, microfabrication involves rather sophisticated methods (see above). One way to circumvent complicated microfabrication relies on the fact that the shape of torque-driven propeller should not be helical or even chiral. The first demonstration of the geometrically achiral microrobot made of just three interconnected magnetic beads that can be steered efficiently by a rotating magnetic field was presented.^401,402^ Inspired by these findings, the theory of the dynamics of a magnetic object of arbitrary shape was developed in ref 403. In particular, the propulsion velocity for in-sync rotation has a compact form:

2.37 $$\frac{U_{z}}{\omega l}=\mathrm{\Omega ̂}\cdot \mathrm{Ch}\cdot \mathrm{\Omega ̂}$$

where **Ch** is a dimensionless chirality matrix given by the symmetric part of G ·( F *l*)^−1^, where G and F are the coupling and rotational viscous mobility tensors, respectively, and **Ω̂** = **Ω**/*ω* = ***ẑ*** is the unit angular velocity of the propeller. Notice that both the diagonal (owing to the object’s chirality) and off-diagonal (do not necessitate chirality) terms of **Ch** can contribute to propulsion. In the body frame, **Ch** is fixed and determined solely by its geometry, **Ω̂** can be expressed *via* the Euler angles as determined from the solution of the corresponding rotational problem.^403^ It was further suggested that the notion of chirality in torque-driven actuation should account not just for the geometry, but also for the orientation of the magnetic moment. It was predicted that specific (off-plane) magnetization, can render the geometrically achiral 2D object into a chiral one, resulting in unidirectional propulsion similar to magnetic microhelices. A combined theoretical and experimental study of two-dimensional (V- and arc-shaped) structures actuated by an in-plane rotating magnetic (or electric) field was conducted previously.^404^ The relationship between different in-sync gaits, determined by the orientation of the magnetic moment **m**, was established through symmetry analysis involving parity and charge conjugation. It was demonstrated that a magnetic propeller, magnetized along one of the principal rotation axes, exhibits no net propulsion. Magnetized in-plane 2D propellers can efficiently generate propulsion due to a spontaneous symmetry breaking; however, the dual stable rotational solutions corresponding to complementary (to *π*) precession angles *θ* and *π* − *θ*, yield propulsion with equal in magnitude but opposite in sign velocities. This finding indicates that a swarm of such 2D microrobots could not be steered in a controlled fashion as it would exhibit zero ensemble-average speed. It was also confirmed experimentally,^404^ in agreement with theoretical predictions, that the off-plane magnetized arc-shaped propeller can swim unidirectionally similarly to a helical propeller.

Although 2D ferromagnetic (or superparamagnetic) planar structures are of practical interest, due to the ease of microfabrication (*e.g.*, *via* standard photolithography^405^), they are prone to magnetize in their plane, rendering uniform off-plane magnetization at the microscale challenging. However, the controlled propulsion of 2D propellers is still feasible by using a conically rotating field (Figure 4f), **B**_con_ = *B*(***x̂*** cos *ωt* + ***ŷ*** sin *ωt* + *δ**ẑ***),^405^ whereas a static magnetic field of magnitude *δB* is superimposed on the in-plane rotating field **B**_rot_ along the field rotation *z*-axis. The constant field serves to align the magnetic moment along the *z*-axis, thereby determining and enabling the selection of a specific wobbling gait. As predicted theoretically^406^ and demonstrated experimentally,^407^ a highly symmetric flat V-shaped propeller, magnetized in its plane, can swim unidirectionally in a conically rotating field where it rotates in-sync with the field in a limited range of frequencies and propels with a constant (*i.e.*, frequency-independent) velocity proportional to *δ*.

An alternative approach to circumvent complex 3D microfabrication of helical micro/nanorobots is to fabricate 1D flexible magnetic nanowires that deform and acquire helicity when actuated by the rotating field due to an interplay of viscous and elastic forces.^408,409^ It was later demonstrated that the efficient propulsion of flexible magnetic nanowires is due to dynamically acquired asymmetry (*i.e.*, bent shape), while the chirality (helicity) only comes into play at higher frequencies.^410^ Another approach involves the spontaneous aggregation of magnetic nanoparticles into random 3D clusters that can also be steered by an in-plane rotating field.^411,412^ However, such random clusters appear to be significantly less efficient propellers on average (*i.e.*, speed-wise) in comparison with optimal structures (in terms of geometry and magnetization).^413^ Moreover, the quadratic form of eq 2.37 implies that the optimal propulsion speed is a purely geometric property that can be estimated from |*U_z_* |/*ωl* ≤ *λ*_max_, where *λ*_max_ is the largest (by the absolute value) eigenvalue of the chirality matrix **Ch**.

By symmetry, the synchronous actuation by an in-plane (and conically) rotating field yields net propulsion along the field rotation axis.^413^ Diverting (or bending) the trajectory of the micro/nanorobots would typically require rotation of the field’s axis by tuning the Helmholtz coil setup. Such temporal guidance would simultaneously affect the trajectories of all microrobots located at distinct positions. However, spatial control over the trajectories is also feasible by superimposing a static magnetic field that acts in the plane of the magnetic field. The resultant asymmetric in-plane rotating field, *e.g.*, **B**_asym_ = *B*(***x̂*** cos *ωt* + ***ŷ*** sin *ωt* + *δ**x̂***), yields a net drift in the *xy*-plane such that the propulsion direction diverts from the *z*-axis.^414^ These findings indicate that actuation by an asymmetric in-plane rotating field can potentially be exploited for “path planning” of the swarms or micro/nanorobots. Furthermore, superimposing a static magnetic field with a weak spatial gradient on **B**_rot_ can be exploited for focusing/localizing the swarms of micro/nanorobots.^414^

While the application of the rotating field on helical magnetic objects mimics propulsion powered by a rotating bacterial flagellum (*e.g.*, *E. coli*), the in-plane oscillating field can be applied to mimic an alternative biological gait of undulatory locomotion utilized by some eukaryotic cells (*e.g.*, sperm) and worms. The first demonstration of an artificial undulating microrobot was provided in ref 173, where a linear chain of magnetic microbeads linked by DNA and attached to a red blood cell was actuated by an oscillatory magnetic field (Figure 4g). A minimal design of the undulating magnetic microswimmers made of just two rigid links (with one of them magnetic) connected by a torsional spring was suggested in ref 415. In contrast to a self-propelled, force- and torque-free undulatory swimmer—such as the well-known Purcell three-link swimmer,^161^ which requires at least three links due to the constraints imposed by the famous Scallop Theorem—a minimal magnetic propeller can achieve swimming motion with only two links. The analogous nanowire multi-link analog was realized experimentally in ref 416 (Figure 4h). A highly efficient two-arm magnetic nanobot exhibiting a complex (“freestyle”) 3D undulatory gait in the planar oscillatory magnetic field was reported in ref 417.

#### 2.3.1.5 Magnetic Navigation Systems

The use of magnetic systems in micro/nanorobotics allows a high level of accuracy in terms of actuation and guidance while performing wireless navigation tasks. Such control has been of great interest when manipulation is taking place in confined spaces, such as the human body, where it is essential to exert refined control over such miniaturized surgical tools. In this context, the development of efficient magnetic control systems capable of generating diverse magnetic fields (*e.g.*, uniform rotating fields, global gradient fields, frequency-dependent fields) is crucial. It is also crucial to integrate these systems with imaging modalities to establish a comprehensive robotic approach.^418,419,420^

When referring to magnetic actuation, it is important to highlight that microrobots can be either actuated or guided by magnetic fields. Magnetic actuation can give rise to different propulsion mechanisms, such as rolling or helical swimming, but it is also extremely useful to guide microrobotic platforms that are already active, e.g., spermbots and bacterial microrobots.^419^ The implementation of such robots will be extensively covered in the following sections; however, it is important to remark that their actuation mode will be different depending on the tasks they perform, e.g., micromanipulation and cargo transport. This becomes especially relevant for *in vivo* applications, where a stochastic environment with dominant viscosity is found at the microscale range.^161^ These challenging conditions related to force and friction can be addressed by optimizing the design, composition, and actuation of magnetic robots, along with tailoring the electromagnetic field to suit the specific configuration of the microrobot, which can range from individual or collective microagents to capsule endoscopes or catheters.

When constructing magnetic systems to manipulate microrobots, one can find two main design approaches: (i) the combination of different electromagnets conveniently arranged to apply controlled magnetic fields and (ii) the controlled manipulation of permanent magnets.^381,421^ While the first approach can easily provide homogeneous magnetic fields with multiple degree-of-freedom (DoF) together with the possibility of decoupling one from the others, the second approach provides a large actuation workspace thanks to automated robotic arms. Depending on the system requirements, each of them can cover different DoFs, working space, magnetic field magnitude, and/or magnetic field gradients (Figure 4i and Figure 4j).

The design of an electromagnet is generally based on insulated copper wires wrapped around a ferromagnetic core. Although this system presents some constraints in terms of heating and requires an efficient cooling system to extend the working time, it is one of the most extensively used systems. An example of such a system is the Helmholtz coil, which has the ability to generate force-free torque on a magnetic object, generating a uniform and aligned magnetic field to the axial direction, but with a rather limited workspace. However, in such systems, the magnetic coupling (which generally utilizes Lorentz force) is extremely strong, leading to the efficient transfer of power. One can also find the Maxwell coil, which adequately separates different electromagnets to achieve a uniform magnetic gradient.^422^ Third, saddle coils present another interesting configuration whose geometry is constrained to only the surface of a cylinder, achieving a uniform magnetic field or a gradient field orthogonal to the axis of the cylinder.^423^ These configurations can be used individually or in combination to leverage various advantages. For instance, combining saddle coils with Helmholtz and Maxwell coils enables the application of fields and gradients, enhancing manipulation capabilities.^424^ Another interesting configuration is the magnetically nonorthogonal systems of electromagnets, where the electromagnets are programmed in couples, resulting in a more versatile actuation approach in terms of workspace size and shape. One well-known example is the OctoMag,^380^ where electromagnets are oriented from a central axis (a more compact version in the MiniMag configuration).^380^ Other more advanced configurations with more than eight electromagnets have also been reported before (*e.g.*, Omnimagnets),^425^ having the ability to be used both in modular and reconfigurable systems.

The proper manipulation of permanent magnets by translating them and/or rotating them on demand can allow the generation of strong fields. The exact location of such permanent magnets in space can be simulated beforehand to provide a defined magnetic field, controlled in a programmable spatiotemporal manner. Generally, such magnets are moved with robotic arms, *i.e.*, six-DoF robotic arm configuration,^426^ eight-DoF robotic arm configuration,^427^ or by constraining each magnet to rotate along a single axis.^428^ Both configurations can be translated to clinical applications, but they can be sometimes limited by the gravity effect regarding the downward locomotion and the space required for their application. The Niobe system^429^ demonstrated promising performance in terms of safety in conditions similar to clinics. The same manipulation method has been explored by not moving permanent magnets, but electromagnets, giving rise to systems such as the BigMag^430^ and the DeltaMag,^431^ having four and three air-core electromagnets, respectively. When comparing the two configurations—using either permanent magnets or electromagnets—feedback closed-loop systems are simpler to implement and require less computationally intensive simulations.

Some of the most recent advances in the development of electromagnetic setups allowed the development of magnetic surgical instruments that incorporate such magnetic components controlled by external magnetic field generators. Some of the operating magnetic navigation systems on the market at the clinical stage able to generate large magnetic fields over a large workspace are Levita® Magnetic Surgical System (Levita Magnetics, United States), Genesis® (Stereotaxis Inc., United States), Aeon Phocus (Aeon Scientific, Switzerland), and the Navion® (MagnebotiX AG, Switzerland). In particular, Navion® has been tested for diverse magnetic structures (*e.g.*, spherical permanent magnets, microparticle swarms, catheters) and represents a portable configuration of great interest for healthcare facilities.^386^

### 2.3.2 Ultrasound

Ultrasound, renowned for its noninvasive nature and versatility, plays a critical role in medical diagnostics and treatments. Key applications include sonography for diagnostics and extracorporeal shockwave therapy for treatment. Emerging yet crucial fields include sonogenetics,^442,443^ where ultrasound aids in controlling cellular functions, and sonochemistry, where it initiates or enhances chemical reactions.^444^ Additionally, the capability of ultrasound to enhance cell growth^445^ and to perform anesthesia^446^ has broadened its clinical utility. Contrast agents, such as microbubbles, enhance vasculature visibility in ultrasound-based imaging^447^ and have recently been adapted to treat brain diseases^448^ and gastrointestinal pathologies upon activation by ultrasound.

The development of micro/nanorobots has seen significant contributions from ultrasound technology thanks to its safety, deep tissue penetration capability, and ability to generate diverse forces without the need for optical transparency. Looking ahead, significant advancements in the safety and biocompatibility of ultrasound-propelled robots are expected such as enhanced AI integration for superior control and precise manipulation. The increased testing and translation of ultrasound-propelled microrobot technology into animal studies are also anticipated, reflecting a growing focus on translational research. To highlight developments in the field of sound-propelled micro/nanorobots, this section discusses the principles of their propulsion, examines various types of sound-propelled robots, and describes how ultrasound can trigger specific functionalities within these robots.

#### 2.3.2.1 Acoustic Manipulation Principles

When particles are surrounded by fluid and a sound wave is propagated through this fluid, it can exert forces and torques on the particles. While these forces and torques oscillate with the frequency of the sound wave, they can have a bias that remains when averaging them over a period of the sound wave. These net forces and torques can enable the actuation of the particles. Because the motion of small particles in viscous fluids is typically overdamped, the observed motion of the particles usually corresponds to the translational and angular velocities at which the forces and torques acting on the particles are compensated by the Stokes drag forces and torques that originate from the particles’ motion through the fluid. By tuning sound fields with respect to space and time, it is therefore possible to control the position, orientation, and motion of particles. This principle is utilized by acoustic tweezers,^449^ which are versatile tools for manipulating small objects, like cells.

The force and torque exerted by a sound wave on a particle are also called acoustic radiation force **F** and acoustic radiation torque **T** and can be calculated as

2.38 F=∮ΣdA

2.39 $$T=\oint \left(x-x_{S}\right)\times \left(\Sigma dA\right)$$

Here, the integrals ∮d**A** with outwards-oriented surface element d**A** run over the surface of the particle, **Σ** is the stress tensor from the compressible Navier–Stokes equations, **x** is the position of d**A**, and **x**_S_ is the particle’s center of mass. There are different possible origins for the acoustic radiation force and torque. First, the particle can reflect the sound wave, leading to a momentum transfer from the sound field to the particle. Second, the sound wave can be absorbed and thus cause a force or torque. When the sound wave is absorbed by the particle, this again leads to a momentum transfer to the particle. The sound wave can also get absorbed by the fluid, leading to a net fluid flow (*i.e.*, a fluid flow that remains when time-averaging over a period of the sound wave). Known as “acoustic streaming”, this flow can set the particle in motion. Acoustic streaming is a nonlinear effect that can occur in the bulk fluid and near boundaries. When acoustic streaming occurs in bulk fluid, it is also called “Eckart streaming”. In this case, the surrounding fluid moves and carries the particle with it. When the sound wave is instead absorbed near a boundary, the resulting “boundary-driven acoustic streaming” is also called “Rayleigh streaming”. Such streaming can occur near the boundary of a particle and the streaming can set the particle in motion. Third, an acoustic radiation force and torque can occur even without reflection or absorption of a sound wave when the particle is compressible. In this case, the acoustic radiation force and torque are also called “Bjerknes force and torque”. An example of particles with high compressibility is gas bubbles.

By controlling the spatiotemporal structure of the sound field, one can make the acoustic radiation force and torque space- and time-dependent. A widely used structure is a standing sound wave. It often occurs in resonators due to interference between forward and backward propagating reflected waves. For a standing sound wave, the acoustic radiation force has a space-dependence that makes spherical particles move to the pressure nodes or pressure antinodes of the sound field. Whether the particles move to the nodes or antinodes depends on the mass density and speed of sound of the material the particles are made of relative to those of the surrounding fluid. For compressible particles, it is also relevant whether their resonance frequency is smaller or larger than the frequency of the sound. Such standing sound waves are often used in the context of acoustic tweezers. However, for many applications, such as sonography, a traveling sound wave is more common. Phased-array transducers and other tools allow us to generate sound fields with more complicated structures.

The idea of sound-propelled micro/nanorobots involves the use of sound to supply energy to small-scale robots so that they can move or perform other actions. It is important to go beyond the simple passive motion of particles in a sound field as robots belong to the class of “active particles”.^450^ The motion of passive particles typically follows an external field. An example is a charged particle that is pulled by an electrostatic field along the field lines or a spherical particle in a standing ultrasound wave that moves toward a pressure node. In contrast, the direction of motion of an active particle depends on its current orientation. An example is a microorganism that swims parallel to its current orientation. When the orientation of this microorganism changes due to rotational Brownian motion, its direction of motion changes accordingly. While for an individual particle it is possible to mimic active motion with a passive particle by tracking its orientation in real-time and rotating the external field that is used to move the particle accordingly, this is not feasible for a swarm of particles. Here, passive particles and active particles behave differently. While all the passive particles would move in the same direction when they are pulled by some external field, the active particles would move individually in directions that correspond to their current orientations.

An option to realize active propulsion with sound is to expose a particle with an asymmetric shape to a sound wave. In this case, the sound will lead to boundary-driven acoustic streaming around the particle, which leads to a flow field that is reminiscent of the flow field that some microorganisms generate in their environment for propulsion. A simple example is a cone-shaped microparticle that is exposed to a traveling ultrasound wave. Because the particle shape is not spherical, the acoustic streaming and its interaction with the particle have a preferred direction. This results in a nonzero net acoustic radiation force acting on the particle and propelling it forward. As is typical for robots and other active particles, the particle’s direction of motion depends on its current orientation.^451,452^ For a traveling sound wave, one does not observe ideal active motion in the sense that the propulsion speed of the particle depends only on the particle’s current orientation and has, for all orientations, the same modulus and the same tilting angle relative to the particle’s orientation. Instead, there is also a partial dependence on the orientation of the particle relative to the direction of the wave vector. When necessary, this problem can be overcome using isotropic sound as such sound contains no directional information.^451,452^ Interestingly, it is not necessary to break the symmetry of the particle shape to enable directed propulsion. Instead, an internal symmetry-breaking of particle properties can be also sufficient.^453^ Besides translational motion, acoustically propelled robots can also show other types of activity, such as rotational motion^454^ or other functionalities triggered by sound (see further below).

The properties of acoustically propelled robots can be studied in experiments and by theory. While performing experiments is straightforward, theoretical methods allow us to obtain deeper insights into the studied system and can help explain experimental observations. With theoretical approaches, it is also possible to obtain results that are difficult or impossible to measure in experiments. An example is the investigation of the dependence of the acoustic propulsion of robots on fundamental parameters, such as the viscosity of the surrounding fluid. While in a theoretical approach it is relatively easy to change the shear or bulk viscosity of the fluid and to study how the acoustic propulsion changes, it is practically impossible in experiments to change one of these viscosities without changing other parameters of the system. For example, changing the shear viscosity would be possible by choosing a different fluid, but this would also change the bulk viscosity, mass density, or speed of sound of the fluid. On the other hand, it is not reasonable to rely only on theoretical methods as experiments are important to confirm theoretical predictions.

Theoretical approaches can be distinguished as analytical or numerical. Analytical approaches aim at obtaining results in the form of mathematical expressions based on a derivation. For studying acoustically propelled robots, one typically starts from the compressible Navier–Stokes equations and linearizes them by a perturbative expansion.^455^ Motivated by the fact that the wavelength of the sound is typically much larger than the robots, one does not describe the sound propagation in detail but just assumes a bulk fluid around the particle that moves periodically forward and backward relative to the particle. One also focuses on specific particle shapes that are easier to address analytically. An example is a dumbbell shape that consists of two spheres that are so far from each other that their hydrodynamic interaction is negligible and that are connected by a solid rod that is so thin that its effect on the flow field in the surrounding liquid can be ignored. After some further steps, one can obtain an expression that describes the propulsion velocity of the considered particle. An advantage of such an analytical approach is that one obtains mathematical expressions that explicitly show how the propulsion or other features of the robots depend on the parameters of the system. Another advantage is that these expressions allow for deeper insights into the underlying physical mechanisms. A disadvantage of such analytical approaches is that they usually involve strong simplifying assumptions such as the fact that the incompressible instead of the compressible Navier–Stokes equations are used (although sound propagation with a finite speed of sound is covered only by the compressible ones), the linearization of the Navier–Stokes equations, and the restriction to special particle shapes. Hence, the analytical approaches are usually not applicable to realistic situations.

Numerical approaches, on the other hand, aim at obtaining results by a computer simulation, *i.e.*, by numerically solving fundamental equations that describe the behavior of the studied system. The most straightforward approach is numerically solving the compressible Navier–Stokes equations. In this simulation, one generates a sound wave by prescribing a pressure/velocity oscillation at one end of the system. The wave will then propagate through the system, interact with the particle, and leave the system again (if not absorbed in the system). This will allow us to see, *e.g.*, the generation of the flow field around the particle and the resulting forces and torques (obtained by eq 2.38 and eq 2.39) that are exerted on the particle. Standard methods for solving the Navier–Stokes equations numerically are the finite element method (FEM) and the finite volume method (FVM). An example of a corresponding software package using the FEM is COMSOL. Examples of software packages using the FVM are OpenFOAM, which is open source, and AcoDyn (Accelerating research and development using software solutions, https://acodyn.com), which is relatively new and optimized for acoustofluidic simulations, *i.e.*, for simulations of the interaction of sound with fluids. An advantage of this numerical approach is its wide applicability as it does not require strong approximations of the system considered. A disadvantage is that these simulations do not reveal directly the dependence of the system’s features on the system parameters. Instead, one must run the simulations for several parameter values and then guess the underlying functional dependence of the features of interest on the varied parameters. For example, this has been done to reveal the effect of the shape and size^452,456,457,458^ or orientation^451,452^ of the particles, the viscosity of the surrounding fluid,^459^ and the intensity and frequency of the sound on the acoustic propulsion of microrobots. Another disadvantage of simulations can be high costs.^451^

A reason for the large complexity of the simulations is the large number of finite-volume cells needed. The cells must be small around the particle to cover the particle shape with a sufficiently high resolution, and the system considered is typically much larger than the particle. A typical example is a micrometer-sized particle in a millimeter-sized blood vessel. Another reason for the large complexity is the required number of steps in time. When dealing with ultrasound with a frequency of 1 MHz, the time-step size in the simulations must be chosen orders of magnitude below the period of 1 μs. To take the Courant–Friedrichs–Lewy condition into account, which is a stability condition for the simulations, it can be necessary to reduce the time-step size even further. On the other hand, the time intervals that are usually studied can be 100 times the period or even larger to allow for relaxation of the flow fields. Thus, the simulations typically involve several millions of cells for the spatial discretization and several millions of time-steps. To speed up the simulations, one could, similar to the analytical approach, replace the Navier–Stokes equations with a linearized version that originates from a perturbative expansion. In this case, one must solve numerical equations at the lowest order that describe the oscillatory motion of the fluid in the sound field and equations of the next-higher order that describe the acoustic streaming. The flow described by the lowest-order equations may relax much faster than the solution of the full Navier–Stokes equations. The equations of the higher order must be solved for the same time interval as the full Navier–Stokes equations, but here much larger time-steps are possible as the time-step size for these equations is not prescribed by the frequency of the sound wave but by the velocity of the acoustic streaming, which is often a few micrometers per second.

#### 2.3.2.2 Standing Wave-Based Propulsion

Mallouck and Wang’s research groups have pioneered some of the earliest ultrasound-based micro/nanorobots. Typically, these rod-shaped robots are composed of bimetallic materials like Au-Ru and Au-Ni-Au, and feature asymmetric geometries with concave and convex ends. They have a length in the range of 1–5 μm and cross-sections of 100–250 nm.^59,460^ Many of the early microrobots were tested in ultrasound resonance chambers (Figure 5a) and, therefore, can be classified as standing wave-based propulsion systems. Wang *et al*. found that these nanorobots could exhibit rapid propulsion along the pressure nodes in the standing wave fields of an acoustic chamber when stimulated acoustically at 3.7–4.1 MHz, matching the chamber’s resonant frequencies. Their remarkable speed, achieving up to 50–100 body lengths per second, can be attributed to the concave and convex shapes at their ends, which create asymmetric vortices for unidirectional propulsion. Additionally, the nanomotors’ dynamic response is enhanced due to the large acoustic impedance mismatch between the metallic structures of the motors and the surrounding environment, which also leads to strong scattering of the ultrasound. This is because the speed of sound and mass density in the metal are much larger than in water.

**Figure 5:**
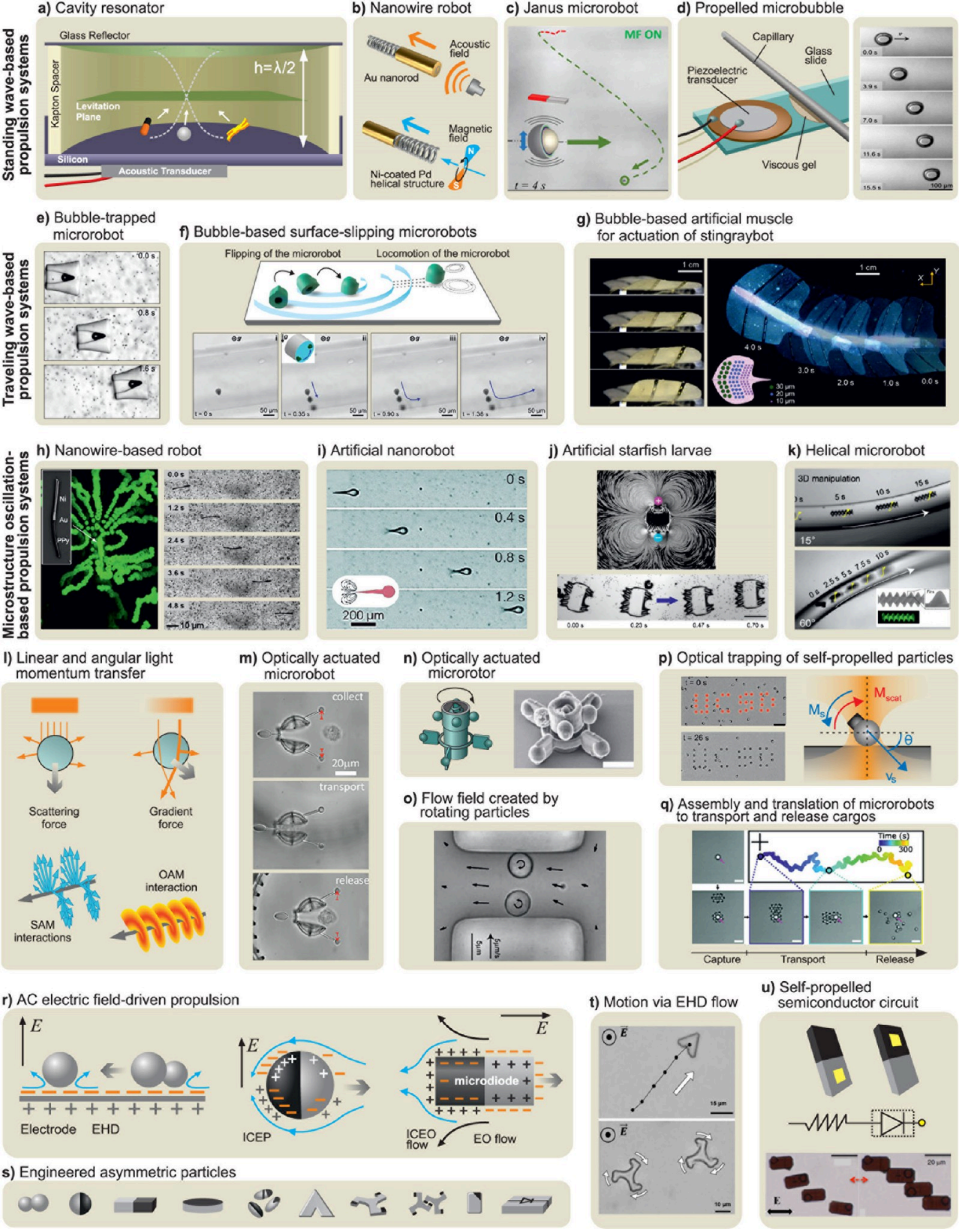
**Physical propulsion mechanisms generated by (a–k) ultrasound, (l–q) light, and (r–u) electric fields. a–d)** Standing wave-based propulsion systems: **a)** Cavity resonator that utilizes standing waves to levitate and control the propulsion of nanorobots. Reproduced from ref 462, Copyright 2023 American Chemical Society and ref 59, Copyright 2012 American Chemical Society. **b)** Magneto–acoustic hybrid nanorobot, showcasing dual propulsion modes using both acoustic and magnetic fields. Reproduced from ref 87, Copyright 2015 American Chemical Society. **c)** Nanorobot designed with an asymmetric material composition that results in varying densities to facilitate asymmetric oscillation and initiate propulsion upon ultrasound stimulation. Reproduced from ref 461, Copyright 2020 WILEY-VCH. **d)** Experimental setup featuring a discoidal microbubble positioned in a narrow slit between glass boundaries, exposed to ultrasound for propulsion. Reproduced with permission under a Creative Commons CC-BY License from ref 399, Copyright 2023 Springer Nature. **e–g)** Traveling wave-based propulsion: **e)** Translational locomotion of an acoustically propelled microrobot having a bubble trapped at its center. Reproduced with permission under a Creative Commons CC-BY License from ref 473, Copyright 2015 Springer Nature. **f)** Bubble-based surface-slipping microrobot effectively propels in both 2D and 3D artificial vessels. Reproduced with permission under a Creative Commons CC-BY License from ref 471, Copyright 2020 National Academy of Sciences. **g)** Microbubble-based artificial muscle for the actuation of a stingray-inspired stingraybot. Reproduced with permission under a Creative Commons CC-BY-NC-ND License from ref 476, Copyright 2024 bioRxiv. **h–k)** Microstructure oscillation-based propulsion: **h)** Nanowire-based robot featuring a metallic head and flexible tail propelling at a nearly constant velocity when subjected to ultrasound. Reproduced from ref 483, Copyright 2016 American Chemical Society. **i)** Directional motion of a bio-inspired artificial microrobot driven by large-amplitude oscillations of soft flagella *via* acoustic actuation. Reproduced from ref 486, Copyright 2017 Royal Society of Chemistry. **j)** Microrobot mimicking the ciliary bands of starfish larvae to facilitate ultrasound-based propulsion. Reproduced with permission under a Creative Commons CC-BY License from ref 487, Copyright 2021 Springer Nature. **k)** Sound-driven helical microrobot that features asymmetric double helix design interacting with the incident acoustic field to achieve propulsion within 3D vascular channels. Reproduced from ref 488, Copyright 2023 American Association for the Advancement of Science. **l–q)** Assembly and manipulation of microrobots with the transfer of light momentum: **l)** Linear and angular light momentum transfer to matter. Ray optics explanation of the scattering forces and gradient forces (top). Spin and orbital angular momentum interaction with matter (bottom). **m–o)** Passive structures controlled with light. **m)** Cell collection with an optically actuated microrobot. Reproduced from ref 511, Copyright 2024 WILEY-VCH. **n)** Microrotor assembled with optical tweezers. Scale bar is 8 μm. Reproduced with permission under a Creative Commons CC-BY License from ref 510, Copyright 2017 Springer Nature. **o)** Mapped flow field created by the rotating particles from spin angular momentum transfer. Reproduced from ref 512, Copyright 2006 Royal Society of Chemistry. **p,q)** Autonomous microrobots powered from within: **p)** Optical trapping principle of self-propelled active particles. Scale bar is 10 μm. Reproduced with permission under a Creative Commons CC-BY License from ref 514, Copyright 2021 Springer Nature. **q)** Assembly process of microrobots around a passive cargo (pink), autonomous translational motion, and release. Cross bar is 20 μm. Reproduced with permission under a Creative Commons CC-BY License from ref 515, Copyright 2022 WILEY-VCH. **r–u)** Principles and examples of AC electric field-driven propulsion effects in active systems: **r)** EHD flow around a stationary spherical dielectric particle; a metallo–dielectric spherical particle undergoing induced charge electrophoretic motion (ICEP); and a particle system including microcircuit to rectify the DC field from an external AC electric field. Reproduced from ref 516, Copyright 2022 ELSEVIER. **s)** Examples of engineered asymmetric structures used in different active systems. Reproduced from ref 516, Copyright 2022 ELSEVIER. **t)** Various trajectories of particles moving *via* EHD flows. Reproduced from ref 533, Copyright 2017 WILEY-VCH. **u)** Self-propelled semiconductor circuit powered by an external field. Reproduced with permission under a Creative Commons CC-BY License from ref 530, Copyright 2018 Springer Nature.

Further innovations have also led to the creation of a multi-actuation nanomotor, which combines a Ni-coated Pd helical appendage with an Au nanorod (Figure 5b).^87^ This design allows the nanorobot to be propelled acoustically by wave scattering from the Au segment while a rotating magnetic field drives the helical segment for additional magnetic propulsion and steering. In another study, a nanoscale motor was developed with an asymmetric material composition (Figure 5c). The microrobots, composed of spherical SiO_2_ particles, were half-coated in high-density materials such as Ti, Ni, or Pt while the other half remained uncoated. When these microrobots were exposed to standing wave ultrasound, the asymmetric coating caused unequal oscillations between the dense and less dense sides. This difference in oscillation led to acoustic microstreaming, ultimately producing translational motion.^461^

Recently, micro/nanorobots have been cleverly employed to showcase unique behaviors. For instance, McNeill *et al*. have demonstrated a dynamic method of self-organization using ultrasound for 3D synthetic bimorph nanospinners.^462^ Within a standing acoustic wavefield, the randomly oriented spinners assemble at the pressure node. Over time, these microspinners undergo phase separation, adopting one of two orientations (either facing up or down). Spinners oriented oppositely spin in opposite directions and are self-organized into positions both above and below the central pressure node of the acoustic chamber. In another study, Janiak *et al*. recently discovered that stimulating acoustic waves within a narrow slit between glass boundaries form discoidal-shaped microbubbles (Figure 5d). These microbubbles, in a viscous gel, are propelled by the superposition of shape and volume modes on their surfaces, exhibiting unique behaviors such as nucleation, self-assembly, microparticle transport, and deployment.^463^

Many nanorobots have also been applied in cellular studies, such as intracellular propulsion within HeLa cells,^62^ and used in developing strategies for intracellular gene silencing in cancer cells.^63^ Esteban-Fernández *et al*. demonstrated an ingenious method to treat and target human gastric adenocarcinoma cells.^464^ Caspase-3 was encapsulated within these nanorobots, which were then internalized by the tumor cells. Upon ultrasound stimulation, these robots induced motion within the cells, significantly enhancing apoptosis efficiency. Remarkably, this approach achieved up to 80% apoptosis of human gastric adenocarcinoma cells within only five minutes, dramatically outperforming other Caspase-3 delivery methods. This underscores the critical role that micro/nanorobots can play in the treatment of various diseases. While they have shown considerable value in various bio-applications, effectiveness largely depends on the boundaries of the ultrasound resonating chambers. As a result, controlling nanorobots in animal models poses challenges, particularly in establishing a predictable standing wave field.

#### 2.3.2.3 Traveling Wave-Based Propulsion

To date, the motion and propulsion of most ultrasound-based robots are constrained by the boundaries of their resonating acoustic chambers. Standing wave manipulation systems have shown efficacy in manipulating microparticles *in vitro*, such as in petri dishes and microfluidics, and even in small animal models like zebrafish embryos.^465^ However, their application in larger animals, which include models such as mice, pigs, and nonhuman primates with complex 3D vasculature networks, presents significant challenges. Specifically, manipulating objects in 3D using standing waves is particularly complex, as it requires the generation of wave patterns that can control movement along all three axes. To surmount this fundamental limitation, the development of microrobots propelled by traveling acoustic waves, *i.e.*, the capability to operate independently of the vasculature network’s boundary conditions, is essential.

Resonance-based microrobots, which can be externally driven using traveling acoustic waves, offer a promising alternative that interacts directly with the microrobots and operates independently of the acoustic chamber’s boundary conditions. The use of microbubbles in a liquid medium exposed to acoustic waves has been extensively studied in various ultrasound applications. This interaction between bubbles and acoustic fields has proven invaluable for fundamental research in fluid dynamics^466^ and physics,^467^ as well as for various applications in biotechnology^468^ and medicine.^469^ Gas-filled microbubbles in liquids typically exhibit nonlinear and resonant behavior when subjected to sound waves. The nonlinear response during bubble activation allows acoustic energy to be concentrated at high densities, contributing to a significant propulsive force, which ranges from micro- to nanonewtons. Meanwhile, the resonant behavior enables selective actuation of bubbles of different sizes by varying the frequency of the sound waves.

Microbubbles enclosed within soft hydrogel micro-containers (ranging 100–200 μm) can behave as actuators for microrobotic propulsion when stimulated with ultrasound (Figure 5e). These microrobots execute translational motion and generate significant propulsive speed, up to 6 mm s^–1^, which can be regulated externally by an electronic function generator. The propulsive force is attributed to asymmetric microbubble oscillation, creating localized microvortices in the surrounding liquid and radiation forces emitted by the microbubbles. Ahmed *et al*. demonstrated translational and rotational motions by strategically positioning the microbubbles at different locations within the micro-containers. They further demonstrated steerable microrobots by incorporating differently sized microbubbles within a container.^57^ In another study, Bertin *et al*. demonstrated significantly smaller acoustic bubble-based robots within a size range of 10–20 μm. These microstreaming-dominant microrobots were fabricated using the two-photon polymerization method and tested in a controlled traveling acoustic setup, which allowed for theoretical predictions of the net propulsive flow generated by bubble vibration.^470^

Various designs of microbubble-based robots have also been developed. Recently, Aghakhani *et al.* developed a bullet-shaped, 3D microrobot with a spherical air bubble in its core (Figure 5f). Stimulated by ultrasound, the bubble oscillates, aligning the microrobot’s axis through fluidic flow. A small fin was engineered onto its cylindrical surface to enable directional movement. The group also showcased the first bubble-based microrobots operating in 3D fluidic channels.^471^ In another study, Ren *et al*. designed and fabricated acoustically powered, bubble-based microrobots using 3D direct laser lithography. These microrobots could move autonomously in 3D through a combination of acoustic and magnetic fields and selectively transport and position individual synthetic colloids and cells.^472^

To advance the next generation of bubble-based microrobots, further testing in biological fluids is required. Although Ahmed *et al*.^473^ conducted preliminary studies in blood and shear-thinning gels, Aghakhani carried out a comprehensive investigation into the propulsion of acoustic microbubble microrobots through various biological fluids. This included *in vitro* navigation through both Newtonian and non-Newtonian fluids, such as mucus and other biologically relevant media.^474^ The design of bubble-based acoustic microrobots, coupled with their high-shear-rate propulsion mechanisms, could revolutionize the deployment of these devices in complex biofluids, opening new possibilities for minimally invasive targeted therapies.

To date, most microrobots use single or double microbubbles for propulsion. In a notable study, Qiu *et al*. developed arrays of acoustic resonant microcavities that generate significant acoustic forces, which they used to construct a millimeter-sized robot. This robot comprises four rigid surfaces, each embedded with a microbubble array of varying sizes. By alternating frequencies, they successfully achieved bidirectional rotational motion.^475^ In another study, Shi *et al*. introduced a new design paradigm for soft programmable artificial muscles, employing over 10,000 resonant microbubbles. Leveraging this technology, they engineered a bio-inspired ultrasound-controlled wireless stingraybot, featuring variable-sized microbubble arrays integrated into the fins of an artificial stingray (Figure 5g). High-resolution mold–replica techniques were used to create prototypes of these artificial muscles. The stingraybot’s fins feature microcavity arrays in three sizes along their length, producing a natural undulatory motion under sweeping ultrasound frequencies that propels it forward.^476^ Although these robots could generate substantial propulsive forces and potentially overcome significant barriers in applications, ensuring the long-term stability of the microbubbles still poses a major challenge.^477,478^

Bubble-based ultrasound microrobots have also been investigated *in vivo*. For example, Lo *et al*. developed a method to trap and manipulate microbubbles through acoustically generated vortices. By harnessing the unique properties of acoustic vortex fields, they engineered a stable potential well that facilitates precise control over the positioning and movement of microbubbles within a fluid medium.^479^ In a separate study, Lee *et al*. introduced bubble-based microrobots with asymmetric fins, enabling rapid rotational motion when exposed to ultrasound.^480^ This design allows the microrobots to anchor effectively to epithelial tissues and gradually release drugs, thereby ensuring sustained therapeutic effects.

Recently, Fonseca *et al*. demonstrated the self-assembly and steering of FDA-approved commercially available microbubbles with diameters ranging from 1 to 5 μm.^481^ When exposed to external ultrasound, these microbubbles oscillate and scatter the sound field. The interaction between the incident and scattered sound waves, along with the in-phase oscillation of the bubbles, prompts their assembly, driven by a phenomenon known as the “secondary Bjerknes force” in bubble acoustics. Manipulation of these microrobots was achieved by activating a piezoelectric transducer, directing the microrobot along the wave propagation, from regions of high to low pressure effectively following the pressure gradient. By incorporating multiple piezotransducers arranged in different configurations, precise steering of the microrobots was achieved. Fonseca *et al*. employed a similar strategy to navigate microbubble-based robots into the vasculature of a living mouse brain. By activating different sets of piezotransducers, the microrobots were able to navigate upstream, downstream, and across vascular streams under physiological conditions within mice.^132^ In a different study, Yang *et al*. developed a novel method using acoustically controlled tweezers to manipulate genetically modified bacteria equipped with gas vesicles. By employing a high-density ultrasound transducer array, this technique enables precise spatiotemporal control of bacterial clusters within the vasculature of a living mouse.^482^

Recent research has demonstrated the use of polymeric microstructure oscillation within microrobots for generating propulsion, thereby avoiding the requirement of bubbles in robot design. Ahmed *et al*. developed predominant traveling-acoustic wave-based nanorobots, which move through liquids *via* small-amplitude oscillations of a soft polypyrrole flagellum-like tail attached to a bimetallic head (Figure 5h).^483^ Ultrasound exposure triggers oscillations that produce a stable flow field, featuring counter-rotating vortices in the liquid, driving translational motion. Kaynak *et al*. have developed extremely soft and flexible sperm-like microrobots on a larger scale, utilizing UV photopolymerization (Figure 5i). These microrobots generate large-amplitude tail oscillations when acoustically stimulated, resulting in either linear or rotary movement, depending on their design. Typically, when ultrasound interacts with sharp edges,^484,485^ it creates microvortices. Building on this, Kaynak *et al*. further advanced their technology using two-photon polymerization to create 3D micromachines. These devices can induce bidirectional rotational motion, either clockwise or counterclockwise, and enable the trapping of microparticles when stimulated by ultrasound.^486^

In a recent study, Dillinger *et al*. demonstrated the first example of ultrasound-activated synthetic ciliary bands, designed to replicate the configurations observed on the surface of starfish larvae (Figure 5j).^487^ These artificial cilia utilize nonlinear acoustics to create fluid motion through small-amplitude oscillations. By arranging the planar ciliary bands to angle toward (+) or away from (−) each other, the microstructure produces bulk fluid flow resembling a source or sink. Combining nonlinear acoustics with this source/sink arrangement, a new propulsion mechanism for ultrasound-driven microrobots was established, bypassing the limitations of the Scallop Theorem. Furthermore, by aligning the + and – ciliary bands next to one another, an effective microparticle trap is created that emulates the feeding mechanism of starfish larvae. Deng *et al*. have introduced a sound-based helical microrobot inspired by spirochete bacteria, featuring sharp fins that spiral along its cylindrical shaft (Figure 5k). Its asymmetric double helix design interacts with the incident acoustic field, generating a propulsion torque that enables rotation around its long axis. Uniquely, the microrobot’s directionality can be altered by simply adjusting the acoustic frequency. These microrobots have been demonstrated to navigate up and down artificial 3D vascular channels.^488^

A significant limitation of these microrobots is their reliance on high-intensity ultrasound excitation. Zhang *et al*. tackled this challenge by designing composite microstructures featuring soft and hard hinges with Young’s moduli of 20 MPa and 200 MPa, respectively. The soft hinge was engineered using grated lines in a photomask for partial light transmission while the hard region was formed with a transparent region for full light passage. This configuration effectively concentrates acoustic energy at the soft hinge, enhancing oscillation and enabling rapid folding of the micromachine. Using this approach, Zhang *et al*. developed a crab-like motion, manipulating the lengths of the soft hinges and their folding through varying acoustic power levels, showcasing an innovative use of programmable micro-folding.^489^

Despite significant advancements in the field of ultrasound microrobotics, which have introduced unique and exciting capabilities, the experimental setups for most current research remain rudimentary. Both ultrasound microrobots and their experimental frameworks still demand substantial improvements. Looking ahead to the next decade, we expect the emergence of more refined actuation methods and experimental setups capable of testing and maneuvering microrobots with greater precision using traveling waves at safe acoustic pressure levels.

#### 2.3.2.4 Functionalities Triggered by Ultrasound

The use of ultrasound can trigger various functionalities in ultrasound-activated robots. These functionalities can be activated by employing selective frequencies; for example, single or multiple frequencies can be used for propulsion while a distinct frequency may be employed for other functionalities. In this section, we highlight some of these capabilities.

##### 2.3.2.4.1 Trapping of Particles and Drugs

Marine invertebrates in their larval stages, such as *Patiria miniata* (starfish), *Serpula columbiana* (polychaete worm), and *Actinotroch* (phoronid worm), are adorned with densely packed bands of motile cilia around their bodies.^490^ The beating of these ciliary bands creates microvortices in the surrounding water, serving dual purposes: propelling the larvae away from predators and facilitating their feeding mechanisms. Similarly, most ultrasound-activated microrobots scatter or re-radiate secondary sound fields. Adjacent to these microrobots, microparticles (*e.g.*, polystyrene beads, drug-loaded droplets, and biological cells) will primarily experience an acoustic radiation force (F_R_) and acoustic streaming-induced drag (F_AS_). The streaming-induced drag force scales with the particle radius, while the radiation force scales with the particle volume. By comparing their ratio (F_R_/F_AS_), we can predict particle trapping: if F_R_/F_AS_ < 1, streaming dominates, and particles remain untrapped; if F_R_/F_AS_ > 1, the radiation force dominates, and particles can get trapped. We can predict a particle of which size will be trapped by setting F_R_ = F_AS_.^472^ This ultrasound functionality has been exploited to trap microparticles near walls^491^ and in acoustofluidic systems for the selective trapping of microparticles near oscillating microstructures within an acoustic field. Although the utilization of microrobots for microparticle trapping is still new, this technique can be integrated into various external-field microrobots. Utilizing this mechanism, microparticles can be physically trapped, significantly broadening their potential in fields such as targeted drug delivery and micro-manipulation.

##### 2.3.2.4.2 Drug Delivery

When exposed to ultrasound, microbubbles, flagellum-like tails, or other appendages of micro/nanorobots oscillate, generating acoustic microstreaming. By modulating the ultrasound power supplied to the piezoelectric transducer, the velocities of the microstreaming can be finely tuned, facilitating the targeted transport of drug molecules.

Acoustic streaming can significantly enhance mass transport by creating localized fluid flows that actively drive drug molecules through the medium, thus overcoming the slow nature of passive diffusion. This method ensures faster and more effective drug delivery directly to target sites.^492,493,494^ Acoustic streaming could be used in clinical applications such as tumor treatment or localized infection management to improve drug penetration into dense tissues or across biological barriers. This capability is especially beneficial in chemotherapy, where enhanced drug delivery to tumors can dramatically improve therapeutic outcomes.

##### 2.3.2.4.3 Imaging

In addition to its applications in drug delivery, ultrasound can play a pivotal role in the imaging of microrobots. Ultrasound imaging facilitates the real-time monitoring of microrobots within the body, essential for potential medical operations. Many ultrasound-propelled microrobots that incorporate microbubbles are detectable using ultrasound brightness mode,^495^ enabling real-time tracking of the microrobots. Recently, Wang *et al*. introduced a sophisticated and innovative technique for the real-time tracking of robotic microswarms. They demonstrated that within a rotating magnetic field, magnetic microparticles naturally self-assemble into dynamic swarms, generating a three-dimensional rotating flow. This process allows for the precise monitoring of the microrobots using Doppler-mode ultrasound and paves the way for advanced applications in medical robotics.^496^

##### 2.3.2.4.4 Synergistic Actuation

The integration of multiple external fields has significantly advanced the functionalities of ultrasound-based microrobots. Drawing inspiration from the motility of spermatozoa and white blood cells near walls where drag is minimal, Ahmed *et al*. demonstrated the self-assembly of rotating clusters in an oscillatory magnetic field. These microswarms,^491,497,498^ represent a significant breakthrough in microrobot manipulation against blood flow. Similarly, Zhang *et al*. developed a strategy where microparticle swarms execute rolling motions along virtual walls in liquids, obviating the need for physical barriers, by harnessing both magnetic and acoustic fields.^499^ Despite these innovations, steering ultrasound-propelled microrobots remains challenging. Dillinger *et al*.^498^ showed that ciliated microrobots^500^ enable precise navigation *via* magnetic fields. Furthermore, Gao *et al*. demonstrated how ultrasound can guide microrobots powered by electric fields, chemicals, and light through acoustic streaming in complex biologically relevant environments.^74^ This approach not only expands the potential applications of microrobotics but also underscores the transformative impact of integrating diverse actuation methods.

Overall, acoustically actuated micro/nanorobots possess a biocompatible propulsion mechanism that can generate large propulsive forces, offering great potential *in vivo*. However, more research is still needed to turn their potential applications into reality. For example, the understanding of their behavior is still limited, they have not yet been optimized for maximum propulsion efficiency or optimal materials (*e.g.*, bioresorbable ones), and their navigation capabilities can be improved. Given the steep increase in research in this field over the last decade and the great significance of the envisioned applications, it is likely that these limitations will be eliminated in the near future.

#### 2.3.3 Light

In the field of micro/nanorobotics, light offers a versatile and potent tool owing to its high-resolution spatial (<1 μm) and temporal (<100 ms) control together with tunable properties, *e.g.*, wavelength, intensity, and polarization. Light control and manipulation of micro/nanorobots encompasses different physical phenomena as we describe in this section. Photosensitive molecules within polymer networks enable reversible bending and twisting upon illumination.^413,501,502^ Alternatively, photothermal effects—converting light into heat—can induce controlled expansion or contraction.^503,504^ In the aforementioned example, the relevant aspect of light is a scalar quantity through local intensity. In contrast, direct momentum transfer from light to matter generates forces and torques that can be used to manipulate microrobots. This section focuses on those aspects of assembly and manipulation of microrobots using the momentum of light.

A well-established technique to control forces with light is optical trapping, also known as “optical tweezers”, *i.e.*, tweezers made of light to manipulate matter without contact.^505,506,507,508^ Pioneered by Ashkin^509^ and recognized with the 2018 Nobel Prize in Physics, optical tweezers precisely manipulate objects by exploiting scattering and gradient optical forces. Scattering forces propel particles in the light propagation direction while gradient forces pull them toward the intensity maximum (Figure 5l). Depending on the ratio between the radius of the particle *R* and the wavelength λ, different models can describe the optical forces. For the two asymptotic cases, *R* ≫ λ and *R* ≪ λ, the optical forces are respectively described with ray optics and the electric dipole approximation. For particles with dimensions comparable to the wavelength, a complete wave optical modeling of the particle light interaction with Lorenz–Mie theory is necessary to compute the optical forces. To achieve sufficient light gradients and forces, optical tweezers typically employ high numerical aperture objectives (that focus the light strongly) or a counter-propagating beam to compensate for the scattering forces. Devices such as acousto–optic deflectors, galvo mirrors, or spatial light modulators then enable the spatial manipulation of the light and dynamic trapping.

In addition to linear momentum, light can carry angular momentum that exerts torques. Angular momentum can be separated into two terms, the spin angular momentum (SAM) associated with the circular polarization and the orbital angular momentum (OAM) that characterizes the rotation of the Poynting vector along the direction of propagation (Figure 5l). The SAM interaction is performed with a circularly polarized light obtained with a quarter-wave plate transferring the momentum to spin the particle. The OAM interaction is realized with a helicoidally wave front beam obtained with a spiral phase plate or a spatial light modulator to generate a revolution of the particle around the beam axis.

In a straightforward use of optical tweezers as “miniature fingers” at the microscale (Figure 5n), micro-fabricated constituents are grabbed and displaced into a structure, *e.g.*, a rotor.^510^ The rotor is then actuated using the forces of the optical tweezers to rotate the structure, akin to a miniature merry-go-round (Figure 5n). A similar approach is proposed to devise micromachines that can collect, displace, and release single cells at a desired location (Figure 5m).^511^ Alternatively, the angular momentum of light is used to spin birefringent colloids and create microfluidic pumps (Figure 5o).^512,513^ On the other hand, such an approach can have limitations. The assembly is tedious: an external operator must grab and move each component individually and adequately exert forces on the structure to actuate the microrobot. Finally, the structures are inherently passive, they lack autonomy and must be continuously driven with dynamical optical traps to remain active.

In contrast, microrobots made with energy-consuming constituents, such as artificial microswimmers or biological equivalents, are powered from within. They are metamachines or machines made of machines that benefit from the control provided by optical tweezers in a way that opens novel opportunities. The assembly of complex structures from colloidal particles by optical tweezers has been limited by the slow diffusion of micrometer-sized particles, preventing particles from occupying the traps autonomously and requiring the tedious “grab and place” described above. This bottleneck is eliminated using active colloids, whose effective diffusivity is 10^3^–10^4^ higher than the thermal diffusivity. This makes it possible to occupy static traps over a large field of view (hundreds of micrometers) in a matter of minutes instead of days in equilibrium. In effect, tens of optical traps can be occupied in tens of seconds^514^ autonomously, unlocking the opportunities for complex assembly by optical tweezers (Figure 5p). In addition, the interplay of the optical forces with the self-propulsion of active colloids unlocks opportunities beyond the conventional optical trapping of passive particles. For example, the forces of optical traps orient active colloids, owing to the physical (and optical) asymmetry that underpins the self-propulsion of the colloid. For example, Janus particles, such as metal–polymer or Fe_2_O_3_–polymer composites (at neutral pH), have a “back” metal or Fe_2_O_3_ half, which is a strong light-scatterer. Self-propelled particles reorient and align in the direction of propagation of light when crossing an optical trap, remaining stable inside the trap. In the absence of propulsion, the scattering forces lead to the expulsion of the particle from the trap. The propulsion force of the active colloid counter-balances the scattering forces controlled by the laser intensity allowing the position particles into the template formed by the traps (Figure 5p). In close-packed arrangements, the interactions between the active colloids allow structures to remain after the removal of the traps, forming dynamic micromachines.^514,515^ The machines are autonomous and capable to load and displace cargo as directed by external light-gradients (Figure 5q). The cargo can be released and micromachines can be disassembled by removal of the blue light that controls the photocatalytic activity of the active components.^515^ In the case of rotating micromachines made of a central sphere surrounded by active particles, they form self-spinning rotors whose direction of rotation is initially random. The brief application of an optical vortex beam to transfer orbital angular momentum orients the cogwheels,^515^ allowing persistent rotation without the permanent application of an optical torque. The imposition of light with angular momentum therefore allows for control of the clockwise or anti-clockwise direction of the rotation. To conclude, forces and torques from light can be combined with active colloids to provide a versatile and unconventional means of assembly and control of micromachines.

#### 2.3.4 Electric Field

Electric fields, particularly alternating current (AC), are of significant interest with their ability to provide an efficient way of remotely powering active objects.^516^ These fields can be easily adjusted by manipulating the AC signal’s amplitude and frequency. Moreover, electrical field-based actuation overcomes some of the limitations of other power sources utilized for micro/nanorobot locomotion, *e.g.*, limited fuel supply or nonbiocompatibility. Electric fields can control not only the propulsion of active objects, but also their interactions with other objects, even exhibiting frequency-controlled collective motion.^517^ For example, electrically powered microrobots have been shown to enable unified and selective cargo loading, transport, and release.^80,518^ They also offer precise control over the interaction between active particles and targeted mammalian cells. Additionally, exotic interactions controlled by electric fields include localized electroporation and subsequent drug or gene injection, as well as *in situ* nuclear electrodeformation.^519,520^ These capabilities highlight the robustness and versatility of AC electric field as an external source of energy.

To understand the physical mechanisms leading to particle motility, it is important to distinguish the simpler phoretic effects of particle motility along the field, such as electrophoresis,^521,522^ and the more complex electrohydrodynamic effects leading to active propulsion. Active particles can harvest the energy provided by the global electric field and convert it into directional motion by locally induced ionic gradients coupled to fluid flows in the surrounding liquid.^516,523^ In the case of electric fields, time-variable AC fields are of particular interest because they can be facile means to remotely power the active particles while their effects can be tuned through the parameters of the AC signal.

There are different physical mechanisms to drive micro/nanorobots *via* AC electric fields (Figure 5r). Notably, varying the frequency of the applied electric field generates several distinct nonlinear (quadratic with the electric field) electrokinetic effects that can power locomotion in different ways depending also on the experimental setup. In a parallel-plate electrode experimental setup, the proximity of a particle to the bottom electrode substrate distorts EDL induced within the electrolyte at the electrode interface and its associated electric fields. This disruption of uniformity in the applied electric fields due to the particle’s presence results in a tangential field component that acts on the induced EDL, generating electrohydrodynamic (EHD) flow around the particles.^524^ When this flow’s symmetry is broken by particle properties, such as asymmetric geometries (asymmetric doublet)^525^ or asymmetric dielectric properties (as seen in Janus particles), the particles (Figure 5s) can demonstrate different types of trajectories as demonstrated in Figure 5t.^526^ EHD flow is generated in the lowest frequency regime compared to the other mechanisms. The characteristic frequency of EHD is *f*_EHD_ = *D*/(2*πλ*_0_ *H*), corresponding to the charge relaxation time of the EDL induced at the electrolyte–electrode interface, where *λ*_0_ is the Debye layer length, 2H is the distance between electrodes, and *D* is the ionic diffusivity of the electrolyte. A maximum in the EHD effect is expected around this frequency. At higher frequencies, the induced EDL does not fully establish while at lower frequencies the induced EDL screens the powered electrode, limiting the electric field’s ability to penetrate the microfluidic chamber. Due to the nonlinear nature of EHD, the propulsion speed is a quadratic function of the electric field strength *U* ∝ *E* ^2^.

As another mechanism, the polarization of a mobile polarizable particle in a uniform electric field is countered by the development of an induced EDL at the particle surface. The field acting on the diffuse charge within the EDL results in nonlinear (quadratic, *U* ∝ *E* ^2^) electroconvection, creating a quadrupolar hydrodynamic flow known as induced-charge electro-osmosis (ICEO).^527,528^ Breaking the symmetry of this flow at the particle surface causes the particle to move under induced-charge electrophoresis (ICEP).^76,529^ For spherical particles, this symmetry breaking is often achieved by coating one half with a metallic layer, forming a Janus structure. The ICEO flow diminishes at frequencies beyond the charge relaxation time of the induced EDL, also known as the RC time for charging the double layer. The characteristic frequency for ICEP is *f*_ICEP_ = *D*/(2*πλ*_0_ *a*), where *a* is the particle’s radius.

Furthermore, beyond a certain frequency, the metallo–dielectric Janus particles can reverse direction, moving with their metallic hemisphere forward. This effect, known as self-dielectrophoresis,^81,82^ is associated with a net electrostatic force generated in the high-frequency regime where the induced EDLs on both the polarizable hemisphere of the Janus particle and the powered electrode are partially screened. Additionally, nonuniform gradients caused by the particle’s proximity to a wall can induce translation under electrostatic self-dielectrophoresis (sDEP) when the symmetry is broken due to the asymmetric polarizability of the Janus particle’s two sides.

Other less common mechanisms for propelling microrobots under AC electric fields include the use of diode particles. These particles are made of semiconducting materials and structured to rectify the applied AC electric field by conduction in only one direction (Figure 5u).^530^ The resulting DC electric field component between the electrodes of each diode drives electro-osmotic ionic flow along the surface, leading to the particles’ self-propulsion. Additionally, hybrid propulsion modes combine electric fields with other propulsion mechanisms. For example, the electric field can enhance the speed of Janus particles driven by magnetic rolling^531,532^ or photocatalysis under UV light. This enhancement occurs because the electric field stabilizes the metallo–dielectric interface through electro-orientation.

### 2.4 Biohybrid Propulsion

#### 2.4.1 Bacterial Flagellar Based

In nature, bacteria use polymorphic, helical, propulsive organelles, known as flagella, to generate ∼pN-level propulsive force. Peritrichous bacteria, such as *E. coli* or *S. typhimurium*, have several flagellar filaments, each of which is actuated by its flagellar motor. The flagellar motors must rotate at 100s of Hz in the same direction and in synchrony (within ∼4 Hz of one another) to produce a flagellar bundle that generates ∼pN-level force for the propulsion of the bacterium forward at 10s of body lengths per second, leading to Reynolds numbers in the order of 10^–6^. In general, bacterial motility consists of two states, *i.e.*, run and tumble. To effectively swim at such low Reynolds numbers, rotation of the flagellar bundle produces a net viscous force-based propulsion for forward motion, *i.e.*, run. When one or multiple flagella switch their rotation direction, the propulsion ceases, and Brownian motion causes a random change in the direction, *i.e.*, tumble. Periods of run (∼0.9 s) are interspaced with periods of tumble (∼0.1 s), leading to the bacteria’s “random walk”.^534^

In the field of micro/nanorobotics, bacterial biohybrid microrobots, comprised of one bacterium conjugated with an ensemble of nanoparticles, demonstrate random walk while their motion characteristics depend on the attached nanoparticle size and quantity.^535,536^ In contrast, an ensemble of bacteria, constrained by attachment to larger microparticles, results in a significantly different collective random walk. In this case, the motion characteristics depend on microparticle shape, bacteria quantity, and attachment location.^121,537,538^

Self-propulsion of bacteria has been classically studied using resistive-force and slender-body theories.^539^ The significant size difference between the bacterial body (∼2 μm) and flagellar filament (∼20 nm) limits the feasibility of computation or theoretical models that include both physical structures. This limitation is even more pronounced for the investigation of bacterial swarms. To circumvent this challenge, data-driven agent-based models are developed to capture individual behavior and swarms of bacterial biohybrids.^540^ As bacterial biohybrids translate from the laboratory to natural environments, there has been a focus on investigating their behavior in complex environments, including non-Newtonian biological fluids,^183,541^ reductionist tissue models,^542^ and even traveling through microvasculature.^543^ We expect that insights gained from these fundamental investigations will guide the design of the future generation of bacterial biohybrid microrobots.

#### 2.4.2 Eukaryotic Flagellar Based

Eukaryotic cilia and flagella are flexible extensions that project from the cell membrane and their length scale ranges from a few micrometers (algae) to a few millimeters (sperm). Sperm cells are one of the most diverse cell types; therefore, a large variation can be found across species. Despite this diversity, all eukaryotic flagella display the same architecture, a central bundle of microtubules consisting of nine pairs of microtubules surrounding a central microtubule pair. This characteristic “9+2” arrangement is the core of the flexible cell appendages. The motion of the flagella is induced by the sliding of the microtubule pairs toward neighboring pairs by the force of dynein arms. Dynein arms are molecular motor proteins that act as linkers between the different microtubule pairs and attach and detach from the microtubule at different times by the conversion of the chemical energy (ATP) into mechanical work, leading to the bending of the flagellum in propagating waves. This series of bending and the interaction with the surrounding fluid leads to the forward propulsion of the cells. Note that the mechanism of the eukaryotic flagellar motion is fundamentally different from the prokaryotic propulsion, eukaryotic flagella generate bending all along the tail while the bacterial motor generates the torque at the base of a passive tail.

The propulsion of spermatozoa in an incompressible fluid is generally modeled using the Navier–Stokes equation, describing the fluid dynamics and considering viscosity effects and nearby boundaries.^544^ Employing the resistive force theory, Gray and Hancock calculated the velocity of sperm cells and their sinusoidal beating pattern.^545^ These calculations are adopted as the basis for modeling the propulsion of biohybrid, sperm-driven microrobots, taking into account the effect of the artificial components, *e.g.*, the microtubes adding additional load onto the cell and restricting flagella motion, and physiological conditions such as the presence of surfaces, other cells, and highly viscous body fluids.^546,547,548^

##### 2.4.2.1 Sperm Driven

Spermatozoa were first incorporated in microdevices as an efficient propulsion source in 2013.^95^ Thanks to the strong propulsion force of sperm, they can carry additional cargo (microobjects and drugs).^549^ One very convenient way to use the propulsion source of spermatozoa is to capture the cells by their head and let the tail move freely and push the microstructure forward. To capture sperm cells, microtubes that fit the sperm head have been demonstrated to work well for that purpose. The microtubes were fabricated by roll-up nanotechnology^28^ and were small enough so that the cells became trapped and did not escape from the tubes. Specifically, ferromagnetic microtubes offer a method for the capture and remote control of motile cells by external magnetic fields. By the use of strain engineering,^28^ 50-μm long rolled-up nanomembranes were tuned in size to fit single bovine sperm cells.^95^ When immersed in a suspension of bovine sperm cells, the motile cells randomly entered the rolled-up microtubes, became mechanically trapped and started pushing the microtubes forward. The rolled-up nanomembranes contained a nanometer-thin Fe layer, which enabled the magnetic remote directional control of the sperm-driven microrobots by magnetic fields of just a few milli-Tesla.^550^ In comparison to free sperm, the sperm-driven microtubes displayed a velocity reduced by around 80% due to the physical confinement of the cell, which restricts the flagella bending motion. To improve the performance of such hybrid microrobots, shorter rolled-up microtubes (20 μm) were fabricated and used for the coupling with the sperm cells.^551^ This maintained a higher velocity of the biohybrid robots, but the coupling success was lower due to sperm cells being able to escape through the short microtubes more frequently. Next, surface functionalization methods were applied to bring sperm-binding proteins onto the inner surface of the microtubes for enhanced sperm binding.^551^ The extracellular matrix protein fibronectin was coated inside microtubes by linker chemistry or by microcontact printing. This coating provided a higher coupling success rate between the sperm cells and microtubes. The limitation of using Fe and Ti as microtube materials is that the microtube could not change its shape, thus controlled release may not be possible in this approach.

Sperm cell release was achieved by incorporating a thermo-responsive polymer layer into the Ti/Fe microtubes. Thermo-responsive polymers, such as poly-N-isopropylacrylamide, can respond to small temperature changes by a collapse of their hydrogel network so that a large shape change can be achieved. In the case of poly-N-isopropylacrylamide, at temperatures below its lower critical solution temperature, it is hydrophilic and takes up a large amount of water. When the temperature is increased above the lower critical solution temperature, the hydrogel shrinks abruptly as it changes from a hydrophilic to a hydrophobic state. This process is highly reversible and the response temperature can be tuned by co-monomers in the polymer network.^552^ The shape change of the hydrogel layer was turned into an opening/closing mechanism of the microtubes by adding a passive Fe layer on top. This facilitated three key processes: first, the capture of cells within the rolled-up hydrogel tubes; second, magnetic remote control; and finally, the release of cells triggered by a slight temperature increase.^552^ An alternative method for fabricating tubular spermbot scaffolds is mask-less lithography, which uses digital mirrors to project a pattern onto a photoresist.^547,549^ By adjusting the focal distance, slightly conical tubes can be created to hold sperm. This technique allows efficient production of high-density microtubes with tunable length and diameter, ensuring high yield and reproducibility. This precision was crucial for subsequent studies on sperm-driven microrobots in oviduct-like conditions and their further biophysical modeling.^546,547^ These highly viscoelastic body fluids display a viscosity of several orders of magnitude higher than standard cell medium, in which spermatozoa are usually handled in an andrology lab. Hence, to evaluate the ability of sperm-driven microrobots to move through such complex bodily fluids were tested *in vitro*. Bovine oviduct fluid from the early luteal phase was characterized in terms of its rheology and a substitute medium containing 0.2% methyl cellulose was suggested to mimic these properties of the fluid *in vitro*. Even though a reduction of speed was observed, spermbots were still able to propel with velocities of about 50 μm s^–1^.^547^

#### 2.4.3 Mammalian Cell Based

The effective propulsion of mammalian cell-based biohybrids relies on proper cell arrangement (*i.e.*, alignment) onto a passive structure that provides mechanical stability. The design of the robot mainly depends on the cell source, typically finding 3D configurations while using skeletal muscle cells or 2D membranes in the case of cardiomyocytes. In all cases, the contractile behavior of the mammalian cells is exploited to obtain an efficient motion, taking advantage of the high power-to-weight ratio and efficiency of the muscle tissue, sufficient control in terms of repeatability and accuracy, optimal energy storage, robustness, and environmental safety.^553^ The propulsion of muscle-based biorobots is intrinsically linked to its design, being generally biomimetic but finding some very interesting examples in other alternative designs. Currently, most skeletal muscle cell-based biohybrid robots operate at the mesoscale. Examples include systems where cell-laden hydrogels are integrated with 3D-printed flexible, asymmetric designs that mimic inchworm-like crawling movements,^554^ or with compliant, spring-like skeletons, enabling both inertial swimming at interfaces and crawling along surfaces.^555^ In contrast, cardiomyocytes are generally cultivated in 2D environments using thin polymeric films where cells are rationally aligned. One of the key examples, where the typical stroke kinematics observed in jellyfish were successfully reproduced, is the medusoid, where they could replicate the velocity–time function of lobe motion compared to ephyrae.^556^ While using optogenetically modified cells, a well-controlled spatiotemporal contraction was achieved by applying light pulses, leading to a forward thrust *via* the undulatory motion of its fins.^557^ More recently, the synchronized beating and efficient actuation of cardiac cells have been harnessed through the incorporation of a geometrically insulated cardiac node (G-node). This innovation integrates mechano-electrical signaling in a biohybrid fish, enabling self-sustained body–caudal fin propulsion driven by spontaneous rhythmic contractions.^558^

The only examples of mammalian-based biohybrid microrobotic systems at the microscale are the Xenobots, where cardiomyocytes from *Xenopus laevis* embryos were combined with noncontractile tissue with a defined alignment and distribution without the need for a chassis to provide mechanical stability. This microrobot design was the first biohybrid platform to be elucidated using an evolutionary algorithm, where the most optimal were reproduced considering active and passive biological building block designs to obtain an efficient motion. The Xenobots successfully reproduced the motion simulated *in silico*, demonstrating an efficient displacement resulting from contractile cardiac muscle tissue that pushes against the surface of the dish.^559^ Two other Xenobots configurations have also been explored. One of these utilizes multi-ciliated cells that enable swimming by cilial beating and demonstrates self-repair after damage^560^ and self-replication.^561^ The use of neural networks to design biohybrids, prioritizing their further motion rather than relying only on biomimetic designs, establishes a cornerstone in the field. However, a more careful evaluation of the motion mechanisms and collective behavior should be considered in future work.

### 2.5 Controlling Micro/Nanorobot Speed

Effective speed control mechanisms are essential for micro/nanorobots to ensure precise manipulation in medical and environmental applications. In low Reynolds number fluidic environments, where viscous forces dominate over inertial forces, locomotion quickly reaches a steady state. This makes speed control somewhat simpler at these scales compared to the macroscale, where inertia plays a more significant role. In general, control of speed can be achieved in two different ways: either through external actuation fields or *via* intelligent material design.

Chemical gradients can lead to the acceleration or deceleration of self-powered microrobots. Catalytic and other types of chemical reactions can be adjusted to generate propulsion, where controlling the concentration of reactants on micromotor surfaces allows for speed modulation. For instance, H_2_O_2_-powered microrobots can adjust their speed by modifying the fuel concentration.^562^ Similarly, in enzyme-based micro/nanorobots, the kinetics of biocatalytic reactions modulate speed.^563^ By spatially coating enzymes such as urease or glucose oxidase at different concentrations on the surface, enzyme-triggered reactions can be controlled, thus modulating locomotion speed. Electric field-induced chemical propulsion also offers an additional degree of control, allowing asymmetric motion of polarized microrobots through the spatial arrangement of anode and cathode poles.^79^

In the case of electric field actuation, both DC and AC fields can generate micromotor locomotion. Quincke rollers, for example, are motile colloids that move in polar directions under a DC electric field and exhibit complex collective behaviors.^83^ By adjusting the amplitude of the electric field and investigating hydrodynamic interactions between individual microparticles, collective propulsion speed can be modulated. For AC field actuation, dielectrophoretic (DEP) forces, arising from nonuniform electric fields acting on polarized objects, can direct the motion of micro/nanorobots. Studies have shown that by manipulating the shape of the objects, DEP forces can be programmed without specific material design, enabling speed control in microrobots.^564^ Although electric-field-induced locomotion may not be practical for *in vivo* applications, its ease of use in microfluidic environments makes it advantageous for controlled microrobot motion.

Light-driven micro/nanorobots generate locomotion through nonuniform gradient fields around their bodies. Researchers have explored various asymmetric structures, such as Janus microrobots, to enable photothermal, photocatalytic, phototactic, or self-electrophoretic motion when exposed to light.^565,566,567^ The primary strategies for controlling speed include adjusting the light’s intensity, wavelength, or pattern, modifying the material properties of the microrobot, or changing environmental conditions. The intensity and duty cycle of light actuation can influence the generated field gradients or the kinetics of chemical reactions, thereby controlling speed. Additionally, different wavelengths of light can activate or deactivate specific reactions, affecting how fast or slow microrobots locomote. From a material and geometry perspective, factors such as surface coating/structure (*e.g.*, TiO_2_ and Pt), thermal conductivity, and the asymmetric shape of microrobots (*e.g.*, Janus particles or hybrid structures) can influence speed. Moreover, the direction of illumination plays a significant role in directing locomotion and can be used to control speed.

Acoustic wave-based actuation methods are gaining attention in biomedical applications due to their deep tissue penetration and biosafety. Acoustic actuation methods generate strong propulsion by converting acoustic energy into mechanical motion *via* oscillating microbubbles,^473^ flagella,^471,498^ or membranes^489^ integrated into the microrobot’s design. Acoustic waves can also exert a global push or pull radiation force on microrobots, depending on their acoustic contrast factor.^568^ However, while large thrust forces can be generated, controlling speed remains challenging. Although external control over ultrasound frequency and amplitude is straightforward, fine-tuning speed is not trivial. Stopping motion is easier due to low Reynolds number fluid physics, but initiating motion in microbubble-based microrobots is nonlinear as acoustic-induced microstreaming interacts with surrounding boundaries. Moreover, boundary fluid coupling affects the speed of microrobots during locomotion. In contrast, the use of first-order radiation force for acoustic powering allows for better speed control as it minimizes microstreaming effects and boundary interactions. However, creating a uniform acoustic pressure field in complex environments adds to the challenge of speed control. To address this, researchers have developed hybrid methods that combine magnetic field steering to control both the direction and potentially the speed of acoustically powered microrobots.

Magnetic actuation remains the most precise method for controlling and navigating microrobots, making it a popular choice among researchers. Bio-inspired helical microrobots and Janus microrollers are two key designs that generate efficient propulsion under low-intensity rotating magnetic fields. Inspired by bacterial flagella, helical micro/nanorobots can provide precise locomotion in 3D.^109,174^ Additionally, surface microrollers achieve high-efficiency rolling-based translational motion by coupling magnetic torque with their surface.^569^ In both cases, the transition from stationary to motion is rapid, owing to the short settling time in low Reynolds number environments. This situation enables precise control over speed, which can be modulated by altering the frequency or amplitude of the magnetic field.

In summary, controlling the speed of micro/nanorobots relies on a combination of chemical, physical, and external field-based actuation methods. Environmental factors, such as surrounding boundaries, can influence locomotion dynamics, either slowing down or accelerating microrobots. Additionally, medium properties, such as viscosity or shear-thinning effects, can significantly alter locomotion modes and impact speed, which should be considered when designing actuation mechanisms. These principles can be employed individually or in combination to achieve precise control over the locomotion of micro/nanorobots.

### 2.6 Good Practices in Analyzing and Reporting Micro/Nanorobot Speed

As we have seen in the previous sections, analysis of motion is a requirement for the field of micro/nanorobotics. In this regard, the first thing to do is the calculation of speed values. A recent tutorial elaborated on the caveats of this seemingly simple task by emphasizing some good practices to ensure the robustness of calculated speed values.^570^ Below, we briefly summarize these suggestions (interested readers are referred to ref 570 for more details).

Accurately measuring the speed of micro/nanorobots is inherently challenging considering the size scale. The small size leads to two challenges. First, the directional motion of a robot is constantly perturbed by thermal noise. As a result of this Brownian motion, it is often necessary to calculate and report the instantaneous speed, which varies with individual robots and time. The second challenge is to accurately track a single robot, which requires a careful selection of the threshold used in the tracking program, as well as high-quality optical micrographs. Otherwise, the error in the position will propagate to the speed values. It is also important to note that measuring the speed of nanorobots can become even more difficult than that of microrobots. For one thing, they may be below the diffraction limit of conventional optical microscopes, making it impossible to track their positions accurately. Even for moderately sized nanorobots, trajectories can be chaotic due to the Brownian motion, making the calculation of instantaneous speed insignificant. In such conditions, speed (or mobility) can be more accurately captured by measuring the mean squared displacement (MSD). In an MSD plot, the mean squared displacement of a nanomotor ⟨*L* ^2^⟩ scales with (Δ*t*)^2^ if the motor moves ballistically (*i.e.*, in the absence of any noise). If the robot moves diffusely (*i.e.*, dominated by noise), ⟨*L* ^2^⟩ scales with Δ*t*. The two scaling transitions around a characteristic time τ_r_ = 8πη*a* ^3^/*k*_B_ *T*, where *k*_B_ *T* is the thermal energy, η the viscosity of the medium, and *a* is the particle radius. Thus, a microrobot with a diameter of 3 μm moves ballistically if observed within a time window of ∼20 s but diffusively if observed for a much longer time. Because τ_r_ is on the order of 50 ms for nanomotors of *a* = 200 nm, they typically appear diffusive for a CCD of a frame rate lower than approximately 100 fps. Therefore, any straight trajectories reported for nanomotors are suspicious, suggesting macroscopic transport (such as convective drifts).

The power of the MSD analysis is that its parabolic part (for τ_r_ ≪ Δ*t*) and the linear part (for τ_r_ ≫ Δ*t*) can be each fitted to obtain the ballistic velocity *V* and the effective diffusivity *D*_eff_.^22^ Velocity *V* is more meaningful for microrobots than for nanorobots as a small τ_r_ of a nanorobot limits its MSD plot to the diffusive regime. On the other hand, *D*_eff_ is an appropriate and useful quantity for nanorobots, and can be obtained robustly by fitting the straight line of its MSD plot. Once obtained, the *D*_eff_ of a nanorobot serves as a representation of its “speed” or “mobility” and can be compared to other nanorobots having similar size but moving under different conditions. To minimize errors in MSD, it is recommended to collect data from as many robots as possible and to plot only 1/10 of the collected Δ*t* (*i.e.*, plotting Δ*t* ≤ 5 s for a video of 50 s).^571^

Due to the small size of micro/nanorobots and various ways in which their “speed” or “activity” can be misinterpreted, it is crucial to perform proper control experiments and eliminate possible drifts. In addition to a typical experiment involving micro/nanorobots and an activation source (*e.g.*, chemical fuels or external energy), we strongly recommend reporting the results for a micro/nanorobot in the absence of any activation, such as an inert micro/nanorobot in the presence of activation and an inert micro/nanorobot without any activation. These control experiments are extremely beneficial to confirm that a micro/nanorobot indeed demonstrates active locomotion, *i.e.*, not being advected or just performing Brownian motion.

## 3 Theoretical and Conceptual Frameworks for Quantitative and Predictive Understanding of Micro/Nanorobots

### 3.1 General Theoretical Considerations

There are many ways that theoretical and conceptual considerations can complement experimental research activities including applied research. Theoretical proposals can be presented involving feasible novel strategies for making specific types of micro/nanorobots, which require analysis of the governing rules of an environment that is not normally within our sense of intuition. Theoretical frameworks can provide tools to quantify and characterize the performance of the devices after they are built and observed. They can also provide valuable mechanistic insights, enhancing our understanding of how the systems operate. This knowledge enables the development of predictions about how the devices will interact with other components in the system or in real-world applications. Moreover, the appropriate conceptual frameworks such as active matter theory, stochastic thermodynamics, nonequilibrium statistical physics, and low Reynolds number hydrodynamics can help us build theoretical frameworks to predict and analyze collective dynamics and emergent properties of many-component swarm robotics at micro/nanoscale (Figure 6).

**Figure 6:**
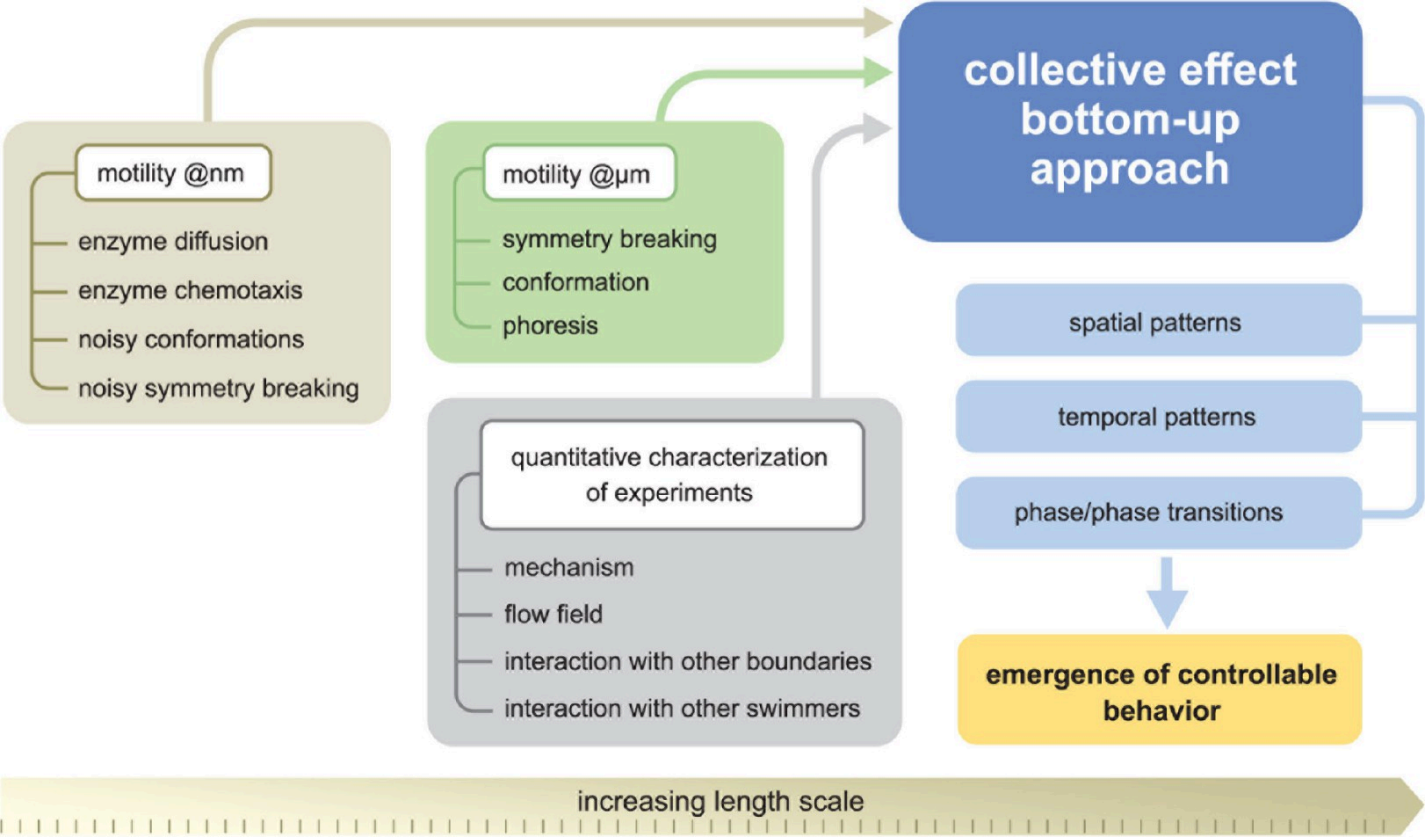
**Multi-scale theoretical strategy to understand and control the collective behavior of micro/nanorobots.** Different conceptual elements contribute to motility at nanoscale and motility at microscale. These elements, together with the quantitative characterization of various physical properties of these systems in systematic experiments, can inform a comprehensive bottom-up description of the system that enables us to make predictions of the corresponding collective properties of the system.

In the last two decades, an abundance of work has been carried out on the theoretical and conceptual front of this very active field of research. In this section, we will summarize some key aspects of these theoretical developments. There are a number of existing reviews on these topics, which readers can consult for more specialist accounts of the achievements in those areas.^572,573,574,575,576,577,578,579^

### 3.2 Motility at the Micrometer Scale: Microswimmers

Swimming or force-free (and torque-free) motion of systems that undergo cyclic conformational changes in a fluid depend sensitively on the symmetrical properties of the governing equations under time-reversal transformations. While for relatively larger systems the dynamics are governed by inertia that is symmetric (even) under time-reversal, for smaller systems, frictional dynamics that are odd under time-reversal symmetry dominate. This means that for small systems, two half-cycles of a deformation cycle would mostly cancel each other, hampering propulsion strategies that would normally work for larger systems unless one specifically designs a sequence of deformations that break time-reversal symmetry while being cyclic.^579,580,581^

It is also possible to design microswimmers that do not undergo cyclic conformational changes by taking advantage of the force-free (and torque-free) nature of the phoretic (interfacial) transport mechanisms.^578^ The idea will be to create an asymmetric structure, known as the Janus colloid, which can produce and maintain an asymmetric distribution of chemicals around the colloid through catalytic reactions, thereby leading to propulsion *via* self-phoresis.^21,176^

One advantage of using these simple design strategies is the ability to perform exact calculations with strong predictive power, even in cases of interaction between two such microswimmers *via* near-field effects.^582,583^ Another advantage is the comprehensive theoretical and mechanistic knowledge about the microswimmers that lends itself naturally to the development of control strategies for the behavior of the microswimmer,^584,585,586,587^ its energy consumption, and energy efficiency^588,589,590,591,592,593^ as well as optimization of its navigation strategies for targeted delivery.^594,595,596^ Moreover, the nature of the environment can also be accommodated in the analysis, be it a simple viscous fluid, a viscoelastic fluid,^183,597,598^ or even a fluid with odd viscosity.^598,599,600^

### 3.3 Quantitative Characterization of Experimental Systems

Theoretical perspectives go hand-in-hand with experiments that help us better understand the motility mechanisms in synthetic microswimmers, in addition to being able to propose new strategies to make self-propelled devices. More specifically, there are two additional aspects where theory can help experiments. The first is to provide a theoretical framework for quantitative characterization of the physical properties of microrobots in order to determine whether or not they self-propel, extract their self-propulsion speeds from among the buffeting background noise and determine their orientation and alignment properties such as angular velocity as well as rotational and translational diffusion coefficients, which can be complex tensorial quantities for devices with nonideal geometric shape.^22,601,602,603^ The second aspect of such theoretical characterization is the ability to extract mechanistic insight from the nonequilibrium activity of every system, such as the discovery of the existence of closed proton loops on the Pt surface in the Janus microswimmer,^604,605,606^ which could explain the observed alignment mechanism of the Janus microswimmer,^189,190^ as well as the flow field profile around the Janus sphere that determines its hydrodynamic interaction with other microswimmers^607^ and characterization of positional and orientational phoretic interactions between two catalytic Janus spheres.^608^ Similar insights have helped to design individually in magnetically actuated microswimmers^173,609,610,611^ that demonstrate a variety of intriguing collective properties.^612,613,614^

### 3.4 Motility at the Nanoscale: Enzymes and Stochastic Swimmers

To design and describe self-propelled systems at the nanoscale, many additional theoretical challenges need to be taken into consideration. For swimmers that undergo conformational changes, the key difference will be that these deformations would occur in the form of stochastic transitions for nanoscale swimmers. In light of this, the biggest challenge will be to ensure that the random sequence of stochastic conformational changes can still break the time-reversal symmetry as required by low Reynolds number hydrodynamics,^615,616^ which amounts to introducing the notion of coherence.^617^ It will also be important to devise a mechanism to actuate the stochastic conformational changes in the appropriate way, ideally by coupling the mechanics to chemical energy conversion.^618^ When such theoretical frameworks are constructed, they can be used to study the properties of the swimming device, including its characterization using the theory of stochastic thermodynamics.^619^ For phoretic nanoswimmers, the challenge will be to characterize the stochastic nature of the chemical cloud of reactants and products around the Janus colloid as all components of the systems undergo fluctuations, which introduces a multitude of competing time scales and leads to several novel dynamical regimes.^620^

There is an intriguing possibility that enhanced diffusion, which can originate from nanoscale swimming or other mechanisms,^621^ is already exhibited by enzymes undergoing catalytic activity and conformational changes at the same time.^211,622^ While numerous mechanisms exist to enhance or modify the diffusion properties of enzymes,^623,624,625,626^ it is crucial to exercise caution when interpreting experimental observations.^627,628^ The ability to model diffusion activity and responses to gradients generated by enzymatic catalytic activity^346,629^ opens exciting avenues for novel self-organization. These insights may shed light on the role of metabolism in structuring the living cell and its functions,^630^ including potential mechanisms for cooperation between different enzymes.^631,632,633^ It is possible to design alternative forms of robotic devices and motors at the nanoscale, with notable examples being artificial cilia-like structures^634,635,636,637,638^ and DNA-origami-based rotary nanomotors powered by an AC electric field^639^ and osmotic force (salt).^640,641^

### 3.5 Collective Effects and Emergent Behavior: Bottom-Up Approaches

When many micro/nanomotors interact with each other, there are many interesting possibilities for collective features to emerge depending on the conditions. A comprehensive theoretical understanding that is built on bottom-up systematic knowledge of the role of the control parameters in the ultimate phase behavior will thus open the possibility of designing swarm behavior in systems with many robots in a way that does not require every layer to be hard-wired and programmed from scratch. In the case of systems with cyclic mechanical and conformational activity, the possibility emerges for the phase of the conformational cycle to acquire interesting emerging features such as complete synchronization and formation of waves—such as metachronal waves—as well as moving patterns with defects and turbulent structures.^642,643,644,645,646,647^ An interesting feature in these systems is that due to the existence of nonreciprocal interactions, many features can be better controlled.^648^ Another striking phenomenon that shows macroscopic phase separation under nonequilibrium conditions arising from self-propulsion is the so-called motility-induced phase separation,^649,650^ which can be destroyed by hydrodynamic interactions.^651^ Similar nonequilibrium condensation phenomena can occur in magnetically actuated systems^651,652,653^ as well as metabolically active enzymatic suspensions,^654^ albeit due to very different underlying physical mechanisms, *e.g.*, a classical analog of Bose–Einstein condensation.^655,656,657^

Catalytically active Janus particles exhibit very interesting collective properties, including collapse instabilities that lead to their clustering and condensation,^658,659^ nontrivial collective response,^660^ as well as the possibility for competition between translational and orientational interactions leading to a diverse range of different behaviors,^660^ which have been studied using sophisticated techniques that include renormalization group theory of critical phenomena.^661,662,663^ While most theoretical and experimental studies of these systems concern cases where the reacting chemicals diffuse faster than the Janus colloids, the case of slow chemical trails has also been shown to entail a similarly rich range of diverse behavior.^664,665,666,667,668^

One of the exciting possibilities provided by phoretic active matter, *i.e.*, catalytic colloidal motors, is that they can exhibit nonreciprocal interactions in a way that appears to violate Newton’s third law, which leads to a variety of novel emergent phenomena originating in scalar systems without alignment^669,670,671,672,673^ as well as a polar system with nonreciprocal alignment.^674,675,676^ On general phenomenological grounds, one can construct nonreciprocal generalizations of theories of phase separation such as the Cahn–Hilliard model.^677,678,679^ These nonreciprocal active matter systems have so far offered possibilities for designing shape-shifting multifarious colloidal self-organizing systems^680,681^ and present possible scenarios for the fast and efficient self-organization of the first primitive metabolic cycles at the origin of life.^595,682,683^ Active nematics represent another notable class of active systems that exhibit novel collective features such as instabilities and defect turbulence,^684,685,686,687,688,689,690,691^ and that offer possibilities to build active micromachines that power microfluidic systems using mesoscale turbulence.^692^

### 3.6 Computational Methods to Study Micro/Nanomotors

Simulation has an important role to play in the investigation of the properties and phenomena that active diffusiophoretic particles possess. It can be used to complement and interpret experimental studies and, perhaps more importantly, can allow one to probe the details of mechanisms, discover new phenomena, and suggest new experiments. To achieve these goals simulation methods should be able to follow the dynamics both of single active particles as well as the collective behavior of many active particles with various geometries in bulk solutions and in the presence of confining boundaries, and possibly subject to external forces such as gravity, electric, or magnetic fields. The schemes should also account for the basic processes that accompany diffusiophoresis, including the local concentration and velocity fields that play an important part in propulsion. Furthermore, the methods should respect features arising from the underlying microscopic reversibility of dynamics and how detailed balance is broken to permit active motion.

The choice of simulation model depends on the characteristics of the system and the observables of interest. In some circumstances, deterministic continuum models are adequate, while stochastic or particle-based models must be used to account for strong thermal fluctuations that affect the dynamics of active particles with micro/nanometer dimensions. If the relevant space scales greatly exceed the particle and interaction lengths, then continuum models that include the coupling between the active particle density field and fluid concentration and velocity fields may be used. In most cases, the dynamics of small active particles in viscous fluids can be effectively simulated using overdamped dynamics, where viscous forces dominate over inertia, and the Reynolds number remains small.

Theoretical expressions for the self-propulsion of a single active particle by a diffusiophoretic mechanism involving nonsymmetric chemical activity on its surface have been derived (for example, see refs 21, 176, 693, 694, 695 ). For simple particle geometries, such as spherical and ellipsoidal Janus colloids or sphere dimers, analytical solutions are available for the translational and angular velocities of the active particle that depend on the chemical concentration gradients on the particle surface.^254,582,696,697,698,699^ As well, these solutions of the coupled reaction–diffusion and Stokes equations, subject to suitable boundary conditions on the particle surface, yield the corresponding concentration and velocity fields that are part of the self-diffusiophoretic propulsion mechanism. For active particles moving near boundaries or with more complex geometries, boundary-element,^700^ finite-element,^701^ Stokesian dynamics,^702^ lattice-Boltzmann,^255,703^ and other numerical methods^704^ can be used to obtain solutions. These solutions provide deterministic trajectories of the active particles’ translational and orientational dynamics along with the fluid fields that accompany this motion. The behavior of active particles with various geometries,^51,705,706,707^ near walls,^189,708^ in viscoelastic fluids,^183,709^ and in other applications, has been studied using these deterministic continuum methods.

Because small active particles experience strong thermal fluctuations that alter their translational and orientational motion, overdamped Langevin models are often employed to follow the stochastic dynamics of these active particles. Overdamped Langevin equations for the position *X* and orientation *u* of a particle can be written using phenomenological considerations.^704,710,711^ For the simple case of a spherical Janus colloid that catalyzes the conversion of reactants to products and is subject to an applied external force, they take the form:^712^

3.1 $$\frac{dX\left(t\right)}{dt}=V_{sd}\left(c\right)u\left(t\right)+D_{t}\frac{F_{\mathrm{ext}}}{k_{B}T}+V_{B}\left(t\right)$$

3.2 $$\frac{du\left(t\right)}{dt}=\Omega_{B}\left(t\right)\times u\left(t\right)$$

where **V**_*B*_(t) and **Ω**_*B*_(*t*) are fluctuating linear and angular velocities. The self-diffusiophoretic velocity *V_sd_* (*c*) depends on the interaction potentials between the chemical species and the particle as well as the chemical affinity that controls the nonequilibrium state of the system. The pre-factor of the external force **F**_ext_ depends on the particle translational diffusion coefficient *D_t_* and the temperature. These equations are coupled to the stochastic equation for the reaction rate for the net number *n* of reactive events that yield product:

3.3 $$\frac{dn}{dt}=R^{a}+\chi D_{\mathrm{rxn}}u\cdot \frac{F_{\mathrm{ext}}}{k_{B}T}+R_{B}\left(t\right)$$

where *R^a^* is the reaction rate on the colloid, *R_B_* (*t*) is the fluctuating reaction rate characterized by the reaction diffusivity *D*_rxn_, and χ = *V_sd_*/*R^a^* is the diffusiophoretic coupling. These stochastic equations can be solved using standard Brownian motion finite-difference algorithms. The resulting ensemble of stochastic trajectories can be used to compute average properties and provide a theoretical description of the active particle trajectories extracted from the experiment. The coupled equations for active particle motion and chemical concentrations can be used to study how external forces can alter the consumption of fuel and the production of product on a diffusiophoretically propelled active colloid.^712,713^ These simple Langevin equations illustrate some of the main contributing factors to active motion. For specific applications, they can be supplemented with other terms, including an additional diffusiophoretic contribution if the system has an imposed concentration gradient, an active contribution to the orientation depending on the symmetry of the particle catalytic surface, coupling between translation and rotation for nonspherical particles, and terms to account for interactions with walls. Similar coupled Langevin equations have been written for thermophoretic active particles.^714^

While the forms of these Langevin equations are based on physical considerations, they may also be deduced from the phenomenological theory of fluctuating nonequilibrium thermodynamics in a manner consistent with microscopic reversibility.^715^ Lastly, it is worth noting that a molecular derivation of the Langevin equations and boundary conditions provides correlation expressions for the parameters appearing in these Langevin equations.^716,717^

The methods discussed thus far consider the motion of active particles in fluids described by continuum concentration and velocity fields, and their coupling to the particle through boundary conditions involving parameters and transport coefficients. Instead, particle-based methods treat the surrounding medium as a collection of particles. While computationally more demanding than Langevin models, these methods have the advantage that once the intermolecular potentials among all particles are given, the dynamics will account for all coupling terms and inhomogeneous concentration and velocity fields that accompany the diffusiophoretic motion. For self-diffusiophoretic propulsion the way chemical reactions take place on the active particle, and possibly in the solution, must be specified. Finally, since the molecular dynamics is microscopically reversible, the mechanisms that maintain the system out of equilibrium and break detailed balance must also be specified fully.^718^ In simulations of chemically powered particles, nonequilibrium conditions can be implemented by directly coupling the system to reservoirs that maintain fixed concentrations of fuel or product at the boundaries (chemostats). Alternatively, these concentrations may be maintained out of equilibrium indirectly by their coupling to other chemical reactions in the fluid that are themselves out of equilibrium, much like in biological systems. For thermophoretic particles, other mechanisms, such as the absorption of radiation, operate to produce an inhomogeneous temperature field on the particle surface.^259,719,720^

The most direct way to carry out such computations is to use a full molecular dynamics description, where all details of the active particles and molecular solvent and solute species are given, along with their interaction potentials. Due to their computational demands, such simulations are rarely carried out for micro/nanomotors in solution, with a few exceptions.^721^ However, full molecular dynamics has been used to study the dynamics of small microswimmers^722^ and small chemically propelled sphere dimer active particles in simple solvents.^253,723^ The latter computations show that the diffusiophoretic mechanism operates on very small few-nanometer length scales.

In most applications of particle-based methods for active particle motion, the solvent particles are described at a coarse-grained level with corresponding effective interaction potentials. Two such methods that have been used often are multiparticle collision dynamics (MPCD)^724,725,726,727^ and dissipative particle dynamics (DPD).^728,729^ In MPCD, the evolution process involves alternating streaming and collision steps. During the streaming steps, solute and solvent particles interact with active particles *via* intermolecular potential and evolve according to Newton’s equations of motion. In the collision steps, the solvent and solute species undergo collective multiparticle collisions.

The dynamics are constructed to conserve mass, momentum, and energy. Reactions needed to describe chemically powered particles have been implemented in this method.^718,730,731^ In DPD, the fluid is again comprised of a set of particles, but these fluid particles interact through conservative, dissipative, and random pairwise forces chosen in a way that momentum is conserved. Energy conservation can also be imposed in the model. Conservation laws are important as they allow connection to the Navier–Stokes (and reaction–diffusion) equations on macroscopic scales and guarantee the force-free propulsion of self-diffusiophoretic particles.

These particle-based methods have been employed to investigate fundamental aspects of diffusiophoretic propulsion. Examples include exploring the details of the propulsion mechanism,^720,732,733^ examining the foundations of nonequilibrium fluctuation formulas for diffusiophoretic particles,^734^ and studying propulsion in nonideal and non-Newtonian fluids,^735,736,737,738^ which behave significantly differently from simple fluids. They have also been applied to scenarios involving confinement^739^ and a range of other contexts.^740^ Active particles with more complicated shapes than spherical have been constructed from spherical building blocks^256,741^ and their dynamics investigated by these particle-based methods. Active particles with helical, L-shape, torus, and propeller shapes have been made in the laboratory and simulation has contributed to our understanding of how they operate and are able to function as micro/nanoscale cargo delivery vehicles.^51,257,258,706,742^

Some of the most interesting and novel phenomena occur when many active diffusiophoretic particles act collectively to produce various nonequilibrium self-assembled structures.^577,743,744,745,746^ The description of the collective dynamics of ensembles of active particles requires the incorporation of additional features in the simulation algorithms. Because self-diffusiophoretic motion produces local inhomogeneous concentration and fluid velocity fields that decay slowly as power laws, when the system contains many such particles these fields give rise to long-range chemotactic and hydrodynamic coupling among the particles. These interactions, along with the direct intermolecular interactions among the active particles, lead to self-assembly processes that produce a variety of nonequilibrium structures that depend on the specific choice of system parameters.

Simulations of the rheology of active suspensions, both biological active organisms and synthetic active particles, have been carried out.^20,702,747^ Focus is often on how particle shape, density, and direct and hydrodynamic interactions determine the forms the collective behavior takes. Most of these simulations deal with squirmer models,^748,749^ where the flow field on the surface of the particle is prescribed and gives rise to the hydrodynamic interactions among particles. For self-diffusiophoretic particles, the surface slip velocity is generated by the asymmetric catalytic activity and depends on the chemical concentrations. This requires a solution of the reaction–diffusion and Stokes equations with boundary conditions on the active particles to account for both hydrodynamic and chemotactic interactions (for example see ref 707).

Langevin models for suspensions of diffusiophoretically active particles have been used extensively to investigate the self-assembled structures and cluster states of these systems. By way of illustration, for the simple case of a collection of identical active particles that catalyze the reaction *S* ⇌ *P* between substrate and product in a possibly reacting fluid environment, the Langevin equation for the position *X_α_* of active particle *α* that incorporates the contributions mentioned above can be written as:

3.4 $$\frac{dX_{\alpha }}{dt}=V_{sd}\left(c\right)u_{\alpha }\left(t\right)+\sum_{i=S}^{P}\mu_{\alpha i}\nabla c_{i}+\frac{F_{\alpha }}{\zeta_{t}}+V_{\alpha B}\left(t\right)$$

Here, the self-diffusiophoretic velocity depends on the concentrations of S and P, the second term on the right accounts for diffusiophoretic interactions due to concentration gradients from other active particles, the third term is the force on particle *α* from direct interactions with other particles divided by the translational friction coefficient ζ*t* of the particle, and the last term is again the random force. The Langevin equation for the orientation of particle *α* is:

3.5 $$\frac{du_{\alpha }}{dt}=\sum_{i=S}^{P}\gamma_{\alpha i}\left(u_{\alpha }\times \nabla c_{i}\right)\times u_{\alpha }+\Omega_{\alpha B}\times u_{\alpha }$$

where the first term on the right describes the active torques due to concentration gradients from other active particles. The corresponding reaction–diffusion equations for the concentration fields are:

3.6 $$\partial_{t}c_{i}\left(x,t\right)=D_{i}\nabla^{2}c_{i}\left(x,t\right)+\sum_{\alpha }R_{i}^{a}\left(c\right)\delta \left(x-X_{\alpha }\right)-R_{i}^{b}(c)$$

The two reactive terms in this equation account for reactions on the active particles and in the fluid phase. In writing these equations, it has been assumed that the expressions for active linear and angular velocities due to an external gradient for a single active particle can be used when the gradients are produced by other active particles in the suspension. In addition, reactive fluctuations in the reaction–diffusion equations are neglected, and fluid velocity effects enter only indirectly through their effects on the single-particle expressions. Nonetheless, chemotactic interactions are very important and these equations can describe a wealth of interesting aspects of the clustering dynamics seen in chemically active particle systems,^744,745,750,751,752^ and have aided the interpretation of such phenomena seen in laboratory experiments. Furthermore, extensions of these equations to describe multicomponent collections of motors with different chemical activities on their surfaces, including complex nonlinear chemical kinetics in the fluid environment, have pointed to the extensive variety of nonequilibrium structures such systems can support.^671,672,683,753^

The simulation of active particle collective dynamics using particle-based methods is straightforward because the only additional component is the inclusion of the direct intermolecular interactions among the particles. Because all solute and solvent species are present (albeit usually at a coarse-grained level of description) the dynamics as described above will produce the chemical and fluid velocity fields that are responsible for the chemotactic and hydrodynamic interactions in the active many-body system. Particle-based simulations of the collective behavior of diffusiophoretic and thermophoretic active particles with various geometries have been carried out and show how the interplay among direct, hydrodynamic, and chemotactic interactions determines the forms that clustering and swarming can take.^754,755,756,757,758,759,760^ Thus, these methods provide a way to understand the relative roles of these interactions play in determining the forms of self-assembled structures. These methods have also been applied to investigate active complex and confined systems (for example see refs 761, 762, 763 ).

A particular advantage of such methods is that it is possible to selectively turn off the chemotactic or hydrodynamic interactions among motors while retaining the diffusiophoretic self-propulsion of the individual active particles. Hydrodynamic interactions can be turned off by either interchanging solvent velocities after collision or sampling from a Boltzmann distribution.^764,765^ Chemotactic interactions can be removed by labeling product molecules so that other active particles do not feel their gradients.^755^ For example, in an investigation of the collective dynamics of Janus colloids,^755^ it was found that turning off chemotactic interactions while retaining those due to hydrodynamic interactions led to the dissociation of previously formed clusters when both interactions were present, although in other circumstances both effects played important roles.

To simulate the active self-assembly and other processes that occur on very large length scales in systems containing macroscopic densities of active particles, continuum descriptions that couple the local densities of active particle properties to the fluid velocity and concentration fields are used. The development of such models typically starts with the formulation of an equation for the distribution function for particle positions and orientations, *f*(**x**,**u**,*t*) = ∑_*i*=1_^*N*^ *δ*(**X**_*i*_ − **x**)*δ*(**u**_*i*_ − **u**), of a system with N active particles.^658^ Because such equations are complicated, one usually considers moments of this distribution, such as the local active particle density *n_c_* (**x**,*t*), the polarization density **p**(**x**,*t*), and higher moments. The hierarchy of moments must be truncated at some point and, in addition, other simplifying approximations, such as the use of pair or single-particle data, mean-field approximations, *etc.*, are made. Several different but related local equations of motion have been constructed depending on the precise details of how approximations are introduced.^743,766,767,768^ The equations for the active particle density fields should be solved along with the reaction–diffusion equations for the reactive species. Numerical solutions of these equations allow one to see how clustering and phase segregation take place under nonequilibrium conditions for different system parameters. In addition, bifurcation analyses of these equations indicate when the homogeneous active particle state becomes unstable and phase diagrams showing the parameters where different cluster states are stable can be constructed. Such field equations also allow for the possibility that the environment in which the motors move may undergo instabilities leading to chemical patterns. The mutual interaction between chemical patterns and active particle clustering can lead to new self-assembled states.^769^

## 4 Collective Behaviors of Micro/Nanorobots

Micro/nanorobots exhibit tremendous potential across a diverse range of fields owing to their diminutive dimensions.^2,123^ However, while their small size confers plenty of advantages, it also imposes significant limitations. For instance, the force generated by an individual micro/nanorobot is relatively minimal, resulting in constrained capabilities for object transportation and manipulation. Additionally, their ability to withstand external environmental disturbances is considerably limited. Therefore, in the research and application of micro/nanorobots, it is typically necessary to consider scenarios involving multiple micro/nanorobots rather than just a single one.^111,365^ It is noteworthy that the presence of multiple micro/nanorobots not only represents a quantitative increase but also results in substantial interactions among them that warrant careful consideration. These interactions can potentially lead to emergent collective behaviors that are not observable in individual micro/nanorobots. Leveraging the collective behavior of micro/nanorobots to enhance system complexity, functionality, and efficiency while deepening our understanding of natural swarming phenomena, represents one of the key research topics in this field.

### 4.1 Interaction between Individual Micro/Nanorobots

Micro/nanorobots can interact through various mechanisms, such as magnetic forces, hydrodynamic forces, chemical signaling, electrostatic forces, and acoustic radiation forces (Figure 7a).^770^ These interactions exert significant influence on each individual, altering their behaviors and serving as the fundamental basis for the collective behavior of micro/nanorobot systems.

**Figure 7:**
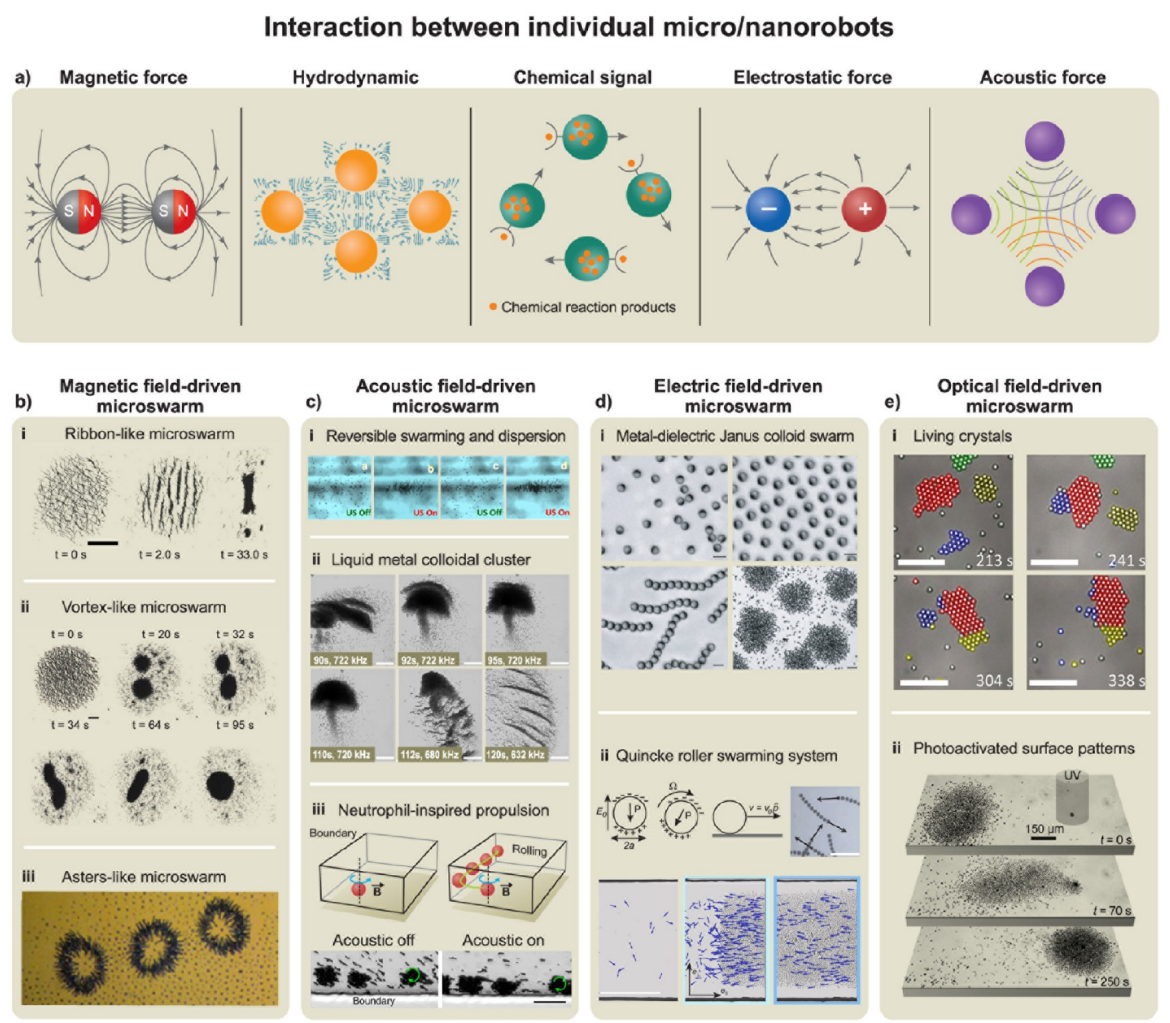
**Interactions and swarming behaviors of micro/nanorobots. a)** Schematic diagram showing different types of interactions between individual micro/nanorobots. **b)** Magnetic field-driven microswarms: (i) Ribbon-like Fe_3_O_4_ nanoparticle swarm actuated by oscillating magnetic fields. Reproduced with permission under a Creative Commons CC-BY License from ref 775, Copyright 2018 Springer Nature. (ii) Vortex-like Fe_3_O_4_ nanoparticle swarm actuated by rotating magnetic fields. Reproduced from ref 808, Copyright 2018 SAGE Publications. (iii) Aster-like microparticle swarm actuated by a vertical alternating magnetic field at the liquid–liquid interface. Reproduced from ref 809, Copyright 2011 Springer Nature. **c)** Acoustic field-driven microswarms: (i) Reversible swarming and dispersion of self-propelled Pt-Au nanowires under acoustic fields. Reproduced from ref 118, Copyright 2015 American Chemical Society. (ii) Dandelion flower-like swarm of liquid metal nanorods under an ultrasound field. Reproduced from ref 814, Copyright 2020 WILEY-VCH. (iii) Neutrophil-inspired acousto-magnetic microswarm. Reproduced with permission under a Creative Commons CC-BY License from ref 491, Copyright 2017 Springer Nature. **d)** Electric field-driven microswarms: (i) Metal-dielectric Janus colloidal microparticles show different collective behaviors in a vertical alternating electric field. Reproduced from ref 517, Copyright 2016 Springer Nature. (ii) Directed collective motion of electric field-driven Quincke rollers. Reproduced from ref 83, Copyright 2013 Springer Nature. **e)** Optical field-driven microswarms: (i) Living crystals formed by colloidal particles under the illumination of blue light. Reproduced from ref 818, Copyright 2013 American Association for the Advancement of Science. (ii) Nematic colloid particle swarm controlled by photoactivated surface patterns. Reproduced from ref 819, Copyright 2014 WILEY-VCH.

#### 4.1.1 Interactions via Magnetic Forces

The realization of interactions among micro/nanorobots through magnetic forces necessitates the incorporation of magnetic components into their structures, thereby facilitating control through external magnetic fields. The manipulation of the assembly behavior of micro/nanorobots is achieved through the strategic application of different magnetic fields, allowing for collective control and the emergence of distinctive patterns. Rotating magnetic fields are commonly used to induce interactions among micro/nanorobots.^609,652,771^ A representative example involves the imposition of a rotating field polarized within the plane of the substrate (*x̂*,*ŷ*). This configuration is expressed as follows:

$$B\left(t\right)=B_{0}\left[\cos\left(2\pi ft\right)\mathrm{x̂}-\sin\left(2\pi ft\right)ŷ\right]$$ 4.1

Here, *B*_0_ represents the field strength and *f* is the frequency. For sufficiently high frequencies, the rotating field induces attractive dipolar interactions that are isotropic when time-averaged. The dipolar interaction between two identical dipoles **m**_*i,j*_ situated at a distance **r**_*ij*_ = **r**_*i*_ - **r**_*j*_ is given by:

4.2 $$U_{m}=-\mu_{0}/4\pi \left\{\left[3\left(m_{i}\cdot r_{ij}\right)\left(m_{j}\cdot r_{ij}\right)/r^{5}\right]-\left(m_{i}\cdot m_{j}\right)/r^{3}\right\}$$

and becomes maximally attractive (repulsive) for particles with magnetic moments parallel (normal) to *r_ij_*.^772^ Performing a time average of the potential yields an effective attractive interaction within this plane. Instances of demonstrated magnetic interactions among micro/nanorobots can be attributed to the diverse magnetic behaviors exhibited by materials. Ferromagnetic materials, such as Fe, Co, and Ni, maintain magnetization even after the removal of an external magnetic field.^773,774^ In contrast, paramagnetic materials can be magnetized in a magnetic field but lose their magnetism when the external field is off.^775,776,777^ To elucidate the impact of differences in materials, we classify magnetic building blocks into two categories: paramagnetic micro/nanorobots^778,779,780,781,782^ and anisotropic micro/nanorobots.^783,784^ The anisotropic features of magnetic building blocks significantly influence agent–agent interactions, potentially yielding different phenomena that are challenging for an isotropic magnetic micro/nanorobot system to achieve.^783^ In comparison to ferromagnetic counterparts, paramagnetic micro/nanorobots circumvent remanent magnetization-induced aggregation when there are multiple ones, making them widely applicable in targeted delivery applications.^785,786^

#### 4.1.2 Interactions via Hydrodynamics

Because micro/nanorobots typically operate in fluidic environments, their motion disturbs the surrounding fluids, leading to deformations at interfaces like air–liquid and liquid–liquid boundaries. Thus, micro/nanorobots can interact with each other through the liquid medium and the hydrodynamic interactions among them assume a crucial role in the establishment of micro/nanorobot systems with numerous individuals. The hydrodynamic drag force encountered by an individual micro/nanorobot undergoing rotation is defined as:^782,783^

4.3 $$F_{i}^{d}=6\pi \eta av_{i}$$

where *η* is the dynamic viscosity of the fluid, **v**_**i**_ is the velocity of the micro/nanorobot, and *a* is the radius of the micro/nanorobot. Considering the linear Stokes equation, a reasonable assumption is that the velocity perturbations induced by distinct micro/nanorobots can be linearly superimposed. In other words, the total velocity perturbation of the flow field at position **r**_i_ caused by (*N-1*) micro/nanorobots equals:^776,781,783^

4.4 $$u_{i}=\sum_{j=1,j\neq i}^{N}o\left(r_{ij}\right)F_{i}^{h}$$

where **o** (**r**_*ij*_) is the Oseen–Burgers tensor. An instance of hydrodynamic interactions among micro/nanorobots can be found in a rotating polymer colloid system.^787^ Magnetic sphere particles are driven by a rotating magnetic field to roll in the same direction on the substrate. Due to their small magnetic moments, the magnetic interactions between them are exceedingly weak, with hydrodynamic inter-particle interaction predominantly governing the system. The propagating shock front of the particles becomes unstable due to the inter-particle hydrodynamic interaction, gradually forming finger-like patterns. As the particle density in the shock region increases, the fingertips become much denser and move faster than the remaining particles.

#### 4.1.3 Interactions via Chemical Signaling

Micro/nanorobots employ chemical reactions to secrete ionic reaction products, generating concentration gradients in their vicinity. This, in turn, influences surrounding micro/nanorobots and induces their coordinated behaviors.^117,304,788,789,790^ As an illustrative example, when exposed to UV light, AgCl particles collectively demonstrate specific behaviors.^791^ The chemical reaction mechanism is expressed as:

$$4\mathrm{AgCl}+2H_{2}O\overset{hv,Ag^{+}}{\to }4\mathrm{Ag}+4H^{+}+4{\mathrm{Cl}}^{-}+O_{2}$$

The production of *H* ^+^ and *Cl* ^–^ induces an electrolyte gradient, resulting in particle motion. Subsequently, these particles aggregate, facilitated by short-range repulsive electrostatic interactions that prevent direct physical contact. Palberg’s group has demonstrated the formation of dynamic complexes resulting from self-assembly processes between an ion-exchange particle, which generates chemical gradients, and a swarm of negatively charged passive colloids.^792,793^ The interaction of the chemically active particle with the passive particles has been explained as the result of electro-osmotic flows promoted by the gradient of the active particle and the interaction with the charged wall acting as a pump. When isolated, the components of the assembly show no movement beyond diffusion. When brought together, they self-assemble into a complex capable of directed swimming, increasing its speed with the number of passive particles in the swarm until reaching a saturation speed. Propulsion is launched by the asymmetric distribution of colloids around the chemically active ion-exchange particles. The assembled passive colloids actively disrupt the radial symmetric flow generated by the ion-exchange particle, setting the swimmer in motion. Cationic ion-exchange particles can replace trapped protons by other cations, generating a local electric field due to the different diffusion coefficients of the interchanged ions.^669^ Esplandiu and co-workers have observed powerful collective motion with ion-exchange Nafion-based nanomotors.^307^ The nanomotors, made of Nafion micro/nanorods capped by a passive material, interact among themselves, leading to the formation of motile clusters driven by the interplay of their self-generated chemical gradients and electric fields. As the nanomotors assemble, their capability to pump nearby matter toward the collective motile structure increases, attracting more nanomotors. The velocity of the mobile Nafion-based clusters increases with size and can reach more than 20 times the velocity of individual nanomotors. Another example of interactions by chemical signals has been reported by Tang’s group.^303^ They have observed very interesting communication-dependent activity leading to the emergence of collective behavior on a hierarchical scale through ion-exchange interactions between ZnO nanorods and sulfonated polystyrene microbeads. Chemical communication is established as polystyrene microbeads release protons, which activates Zn cation release from the ZnO structures, with the Zn cations being incorporated into the polystyrene microbeads. This interaction enhances the reactivity and motion of both the nanorods and the microbeads, resulting in the formation of an active swarm of nanorod–microbead complexes. They have demonstrated that the swarm is capable of macroscopic phase segregation and intelligent consensus decision-making.

Another mechanism for generating signaling and interactions between microrobots, particularly droplets, is *via* locally generated chemical gradients that induce motility due to the Marangoni effect. Sessile droplets on a solid substrate can interact and organize based on vapor-mediated signaling between droplets.^795^ Oil-in-water droplets stabilized by surfactants can interact with neighbors *via* the local release of chemicals by interfacial reactions or interfacial transport. Depending on the specific droplet chemical compositions,^796^ droplets can attract,^337^ repel,^797^ or even chase,^798^ which is a nonreciprocal interaction that is only allowable under nonequilibrium conditions. Nonreciprocal chemotactic chasing between oil-in-water droplets was explained by using a source–sink framework,^798^ where kinetic asymmetry in the transport of oil between droplets led to Marangoni flow-driven motion and predator-prey-like motion. Meredith *et al*. observed a variety of droplet cluster shapes formed by chasing oil droplets^798^ as well as spinning clusters of Janus oil droplets,^799^ where the cluster motion was tied to the cluster symmetry. Liu and Kailasham *et al.* demonstrated that the self-organization of chasing droplets could be tuned by varying the physical parameters (droplet diameter, number ratios) and chemical composition.^794^ In a system with isotropic droplets of identical composition, hexagonally packed clusters formed spontaneously at various surfactant concentrations due to hydrodynamic effects.^800^

#### 4.1.4 Interactions via Electrostatic Forces

Electrostatic forces play a pivotal role in governing interactions between micro/nanorobots, representing one of the most utilized mechanisms in nanoparticle interplay. The nature of electrostatic forces, whether attractive or repulsive, depends on the charges carried by the micro/nanorobots. Additionally, the range of these forces, whether long-ranged or short-ranged, is tunable and can be adjusted based on experimental conditions. To account for the interaction between dimers and the conducting substrate, image dipoles beneath the electrode are employed. The micro/nanorobots’ dipole moment **p**_*i*_ is induced by the sum of electric fields, arising from both externally applied fields and those generated by neighboring dipoles,^801^ and expressed as:

4.5 $$p_{i}=\alpha_{i}\left[E_{0}\left(r_{i}\right)+\sum_{j\neq i}E_{\mathrm{ind},j}\left(r_{i}\right)\right]$$

where **E**_ind,*j*_(**r**_*i*_) is the field generated by the induced dipole *j*. To generate electrostatic forces between micro/nanorobots, it is imperative to establish an electrostatic imbalance within the micro/nanorobot structure. This imbalance ensures that micro/nanorobots carrying disparate charges can effectively interact with one another. Various methods can be employed to induce electrostatic imbalances, including the utilization of a self-generated electric field,^802^ polarization under external electric fields,^517,801^ and photogenerated charge separation.^803^ The propulsion mechanism of bimetallic nanorods is ascribed to a self-electrophoretic process.^802^ Distinct electrochemical half-reactions taking place at opposing ends of the nanorod result in a gradient of ion concentration and, subsequently, a localized electric field that propels the nanorod. The attractive forces between opposite ends enable the dynamic assembly of nanorods into staggered doublets and triplets in H_2_O_2_ when moving in the same direction. The polarization of micro/nanorobots under external electric fields can induce dipolar interactions, which play a crucial role in mediating the formation of diverse collective states among micro/nanorobots.^801^ The manipulation of micro/nanorobot assembly into various clusters has been successfully demonstrated using alternating current electric fields.^517^ Additionally, the inherent properties of photocatalytic materials to generate electron–hole charge separation upon exposure to light offer another avenue for manipulating the interaction between micro/nanorobots through light exposure. Leveraging light-switchable electrostatic interactions, researchers have successfully demonstrated light-controlled dynamic interactions between TiO_2_/Pt Janus microrobots composed of TiO_2_ microspheres and Pt caps.

#### 4.1.5 Interactions via Other Forces

Forces such as acoustic radiation force, van der Waals force, optical force, hydrophobic force, and capillary force have also been proposed to facilitate interactions between micro/nanorobots. Acoustic radiation forces encompass primary forces acting on individual micro/nanorobots within the acoustic field and secondary forces that engender interactions between micro/nanorobots.^118^ Primary forces prompt the migration and assembly of micro/nanorobots in response to acoustically generated pressure gradients. Micro/nanorobots exhibit movement toward low-pressure regions, enabling their localization and swarming through the manipulation of acoustic fields. Van der Waals forces suggested assembling tadpole-shaped microrobots composed of silica microspheres and TiO_2_ arms into spinning dimers, representing another intriguing phenomenon.^59^ Analogous to acoustic fields, optical fields induce mechanical forces on micro/nanorobots proportional to the gradient of light intensity. Light scattering engenders interparticle forces, known as optical binding, allowing the formation of particle patterns within an optical trap. The cooperative interaction of optical binding and optical forces has been successfully employed to assemble Au nanoparticles into well-organized arrays.^804^ Leveraging the properties of hydrophobic forces, microrobots featuring hydrophobic hemispheres spontaneously form assemblies with like-facing hydrophobic sides.^119^ Hydrophobic interactions provide each microrobot within the assembly a range of orientational freedom while Pt caps on the opposing sides generate a net force propelling the assembly. In the realm of bubble-propelled micro/nanorobots, the expelled bubbles serve as signaling agents to mediate interactions.^805^ Attractive capillary forces between bubbles exert a sufficiently strong pull on attached microtubes, facilitating the formation of assemblies. However, it is noteworthy that the experiments demonstrating firm bubble attachment to microtubes were conducted in a thin aqueous film of propylene carbonate with H_2_O_2_, a condition not universally applicable to normal operating conditions in aqueous solutions. The assembly and disassembly of microtubes are determined by the delicate balance between attractive capillary forces and repulsive driving forces. The introduction of a drop of H_2_O_2_ results in the gradual disassembly of microtubes.

### 4.2 Micro/Nanorobot Swarms

The emergent collective behaviors of numerous individual micro/nanorobots led to the construction of micro/nanorobot swarms (microswarms). These swarming systems can be triggered and powered by diverse strategies, such as magnetic, acoustic, light, and electric fields. Additionally, chemical signaling can also generate swarms, as demonstrated by earlier studies on chemically induced swarming behaviors^229^ and recent work on enzyme-powered nanomotors exhibiting 3D collective buoyancy-driven dynamics from Sánchez’s group.^806^

#### 4.2.1 Magnetic Field-Driven Swarms

The magnetic field, characterized by its ease of generation and regulation, high penetrability, and excellent biocompatibility, is extensively employed for the actuation of micro/nanorobots and their swarming systems. In addition to magnetic force and moment exerted by the field, magnetic micro/nanorobots also generate magnetic dipole–dipole and hydrodynamic interactions among themselves. The emergence of magnetic microswarms is a consequence of the synchronized response of numerous magnetic micro/nanorobots to the applied field, coupled with the interactions among them. Based on the different formation mechanisms of magnetic microswarms, they can be classified into two types: internal interaction-dominated microswarms and external field-dominated microswarms.

The interactions among building blocks play a major role in the formation and maintenance of the interaction-dominated microswarms, which can be effectively regulated by adjusting the applied field. As previously mentioned, dynamic magnetic fields can induce attractive interactions between magnetic agents, leading to their assembly into microswarms. For example, under rotating magnetic fields, magnetic Janus particles self-assemble into a hexagonal pattern,^807^ while paramagnetic colloidal particles closely packed into carpet-like swarms without vacancies.^652,772^ Oscillating magnetic fields enable Fe_3_O_4_ nanoparticles to form ribbon-like microswarms with ultra-extensible ability (Figure 7b­(i)).^775^ Ferromagnetic peanut-shaped Fe_2_O_3_ particles can be configured into another kind of ribbon-like microswarm through attractive dipolar interactions under a precessing magnetic field.

Magnetic agents in motion can interact with each other through hydrodynamic interaction, which plays a dominant role in some microswarm systems and determines the swarm state. The rotation of paramagnetic nanoparticle chains perturbs surrounding fluids to generate a vortex, imposing long-range attractive hydrodynamic interaction on other chains. Therefore, the circular vortex-like microswarm is generated under rotating magnetic fields, with its morphology being altered by adjusting the field parameters (Figure 7b­(ii)).^808^ Ferromagnetic particle chains confined at the interface of two liquids undergo periodic oscillations under a vertical alternating magnetic field, causing interface deformation and leading to the excitation of interfacial waves and quasi-static hydrodynamic streaming flows.^809^ These interactions result in the formation of aster-like particle swarms with capabilities for locomotion and pattern transformation (Figure 7b­(iii)). Similarly, micro/nanorobots on the liquid–air interface can also exhibit a variety of swarming behaviors through hydrodynamic interactions.^774,810,811^

In magnetic field-dominated microswarms, all building blocks engage in independent synchronous motion under the influence of global input and the agent–agent interactions are often negligible. Microswarms are predominantly driven by magnetic field gradients that predominantly fall into this category. The movement of disk-shaped microrobots on the air–water interface is regulated by an array of permanent magnets, resulting in the formation of microswarms that are primarily governed by gradient-induced magnetic forces.^773^ In addition, when the magnetic moment of the individuals is small or the distance between them is far apart, their motion is also completely dominated by the external field rather than agent–agent interactions. For instance, under a rotating magnetic field, a swarm of slippery helical micropropellers can move freely in the vitreous humor and reach the retina,^54^ and a swarm of bacteria-like microrobotic flagella can perform directional propulsion in the peritoneal cavity of a mouse.^812^ The movement of individuals within these microswarms is relatively independent and noninterfering, exhibiting no significant difference compared to those moving alone.

#### 4.2.2 Acoustic Field-Driven Swarms

Acoustic fields provide a wireless, noncontact, and biocompatible approach for controlling micro/nanorobots and their swarms, and the utilization of nodes and antinodes leads to diverse microrobotic collective behaviors. Pt-Au nanowires perform free self-propulsion in H_2_O_2_ solution and rapidly aggregate into tight circular swarm patterns upon application of an acoustic field (Figure 7c­(i)).^118^ This is due to the primary acoustic radiation force driving the nanowires toward the nearest pressure nodes or antinodes, while the secondary acoustic radiation force further promotes the aggregation of the nanowires. In another example, Au-Ru microrods aggregate into ring-shaped swarm patterns on the central nodal plane under the influence of acoustic waves, with the swarm pattern dependent on the structure of the nodes and the reflection of the waves.^813^ The core–shell-structured liquid metal nanorods can form stripe-like patterns under an ultrasound field.^814^ By reducing the frequency of the sound waves, the stripes can be merged, ultimately presenting a dandelion flower-like swarm pattern (Figure 7c (ii)).

The combination of acoustic and magnetic fields enables the microswarm to mimic the motion of natural microswimmers such as neutrophils, spermatozoa, and bacteria.^491^ A rotating magnetic field is employed to aggregate paramagnetic microparticles into the swarm. An acoustic field is then applied to push the swarm toward the wall, causing the swarm to execute a rolling-type motion (Figure 7c­(iii)). A similar bio-inspired swarm actuation strategy is achieved with two piezoelectric transducers, facilitating the controlled navigation of microbubble swarms against the blood flow.^481^ The first transducer initiates the self-assembly of bubbles into microswarms and their subsequent movement toward the wall, while the second transducer generates an acoustic field parallel to the channel, propelling the swarm along the wall. This strategy is further expanded to exploit multiple transducers for navigating microbubble swarms in the brain vessels of mice, demonstrating robustness against high flow rates.^132^

#### 4.2.3 Electric Field-Driven Swarms

Micro/nanorobots driven by electric fields exhibit various collective behaviors based on different mechanisms, primarily including electrohydrodynamic (EHD) and electrostatic interactions. Metal–dielectric Janus colloidal microparticles demonstrate different collective behaviors in a vertical alternating electric field (Figure 7d­(i)).^517^ At low frequencies, around the kilohertz range, the electrostatic interactions between particles can be disregarded, resulting in the microparticles existing in a gas state. Elevating the frequency (20–50 kHz) weakens the ionic screening effects and the repulsion between the particles results in a coherent swarm state. As the frequency is further increased to the megahertz range, the particles begin to exhibit attractive interactions, leading to a chain-like state. Furthermore, the introduction of salt into the medium can manipulate dipole interactions, thereby inducing the appearance of a cluster state. Asymmetric colloidal dimers generate dipole interactions under an alternating electric field, forming chiral clusters. Their asymmetry generates an electrohydrodynamic flow in the surrounding medium, driving rotation in the direction opposite to their chirality.^801^

Electrohydrodynamics can be explained as the phenomenon where an electric field induces the accumulation of free charges on the surface of dielectric agents, generating electrostatic stress that is balanced by a fluidic flow. Microparticles of varying sizes and dielectric properties aggregate into microswarms and perform motion due to the unbalanced EHD flow.^815^ Within these microswarms, some particles act as leaders while others serve as followers. Speed of the microswarms can be regulated by adjusting the voltage, frequency, and particle proportion. Particles with identical sizes and dielectric properties can also aggregate into microswarms, but the induced EHD flow is symmetric; hence, the formed microswarms remain stationary. The introduction of new particles with different sizes or dielectric properties can disrupt the symmetry of the EHD flow, enabling the newly formed microswarms to exhibit coordinated movement. Particles with low conductivity exhibit Quincke rotation behavior under a sufficiently strong direct current electric field, which also belongs to the EHD phenomenon. In an electric field-driven system, Quincke rollers are realized using poly­(methylmethacrylate) beads immersed in a hexadecane solution, filling the interstitial gap between two conducting glass slides.^83^ Upon subjecting an insulating sphere immersed in a conducting fluid to an electric field of sufficient amplitude, infinitesimal fluctuations disturb the charge distribution on the sphere’s surface, inducing a net electrostatic torque that propels the sphere to rotate at a constant speed in random directions. At low densities, the rollers exhibit uniform rotation in random directions, constituting an isotropic gaseous phase. Beyond a critical area fraction, a macroscopic band spontaneously materializes, propagating at a constant velocity through the isotropic phase. With further escalation of the area fraction, the propagating bands eventually converge within the racetrack, giving rise to a homogeneous polar-liquid phase (Figure 7d­(ii)). A new mechanism for rationally creating multimodal, reconfigurable, patterned swarms has recently been proposed and validated in an electrically driven semiconductor micromotor system. By using light to modulate the electric polarization of semiconductor particles, various swarming modes have been achieved through the controlled balancing of two effects, electrostatic attraction and electrorotation, governing swarm motion in distinct manners.^73^

#### 4.2.4 Optical Field-Driven Swarms

Photosensitive materials, such as AgCl and TiO_2_ particles, are commonly used to construct light-driven microswarms. The application of optical fields generates concentration gradients around these materials, inducing the emergence of collective behaviors. Under laser illumination, graphite-SiO_2_ Janus colloidal particles exhibit propulsion in a water–lutetium mixture caused by diffusiophoresis.^816^ Due to the self-trapping effect of Janus particles, small clusters form when the particle density is low, transitioning to large clusters surrounded by a dilute gas phase as the particle density increases. TiO_2_/SiO_2_ Janus particles generate localized chemical gradients under UV light, resulting in propulsion due to the diffusiophoresis effect.^817^ Because the diffusiophoresis center is located at the TiO_2_ reaction site, inactive silica particles are attracted to the TiO_2_ surface, forming large two-dimensional self-assembled swarms. In a study involving synthetic colloids, Palacci *et al*. demonstrated the ability to toggle self-organized clustering on and off (Figure 7e­(i)). This dynamic control allowed for the formation of crystals that dissolve upon deactivation of the light source.^818^ These particles comprise a Fe_2_O_3_ cube partially enclosed within a polymeric sphere capable of catalyzing chemical reactions under light exposure. When illuminated with blue light, Fe_2_O_3_ catalyzes the decomposition of H_2_O_2_, creating chemical concentration gradients and triggering osmotic and phoretic effects. The observed self-assembly behavior emerges from a synergistic interplay of propelling forces, osmotic effects, and interactions between colloidal and tracer particles.

In general, locomotion direction of micro/nanorobots propelled by electrophoresis is difficult to control. Patterned substrates and movable light spots can facilitate the controllable movement of optical field-driven microswarms. On substrates chemically modified with photosensitive self-assembled monolayers, polystyrene microparticles exhibit a *cis* configuration under UV light exposure and revert to a *trans* configuration under blue light exposure (Figure 7e­(ii)).^819^ The application of an alternating electric field leads to the formation of aster-shaped or vortex-like microswarm patterns. Movement of the formed microswarm along a pre-designed path can be achieved by altering the position of the light spots and adjusting the liquid-crystal-enabled electrophoresis mechanism. TiO_2_ microparticles spontaneously aggregate into flocks in water due to electrolyte diffusiophoresis.^113^ After irradiation by UV light, their behavior changes from random Brownian motion to dilatational negative phototaxis with high velocity due to the nonelectrolyte diffusiophoretic interaction. Controlled navigation of the microswarm can be achieved by applying periodic pulses of UV light. The microswarm also exhibits adaptive restructuring, such as reversible dilatation and splitting, which is advantageous for adapting to complex environments. Moreover, the temperature gradient between the NIR light exposure area and the unexposed area results in natural and Marangoni convections in the liquid medium.^820^ Tiny objects can be attracted to the central area of the convection and form microswarms based on agent–agent interactions such as local electrostatic attractions or diffusiophoretic repulsions. Consequently, this method has been employed to induce various types of microswarms, with TiO_2_/Pt, SiO_2_/Pt, TiO_2_, and ZnO microparticles, as well as microorganisms (*e.g.*, *E. coli*) serving as building blocks. The controlled locomotion of the formed microswarms can be achieved simply by moving the near-infrared spot.

#### 4.2.5 Concentration Field-Driven Biohybrid Swarms

The biotic components in biohybrid microrobots are whole cells with highly sensitive receptors for a multitude of chemical (known as “chemoeffectors”) and physical stimuli (*e.g.*, light). Cells detect the presence of environmental stimuli gradients, process their sensory inputs, and synthesize a prioritized collective response when multiple cues are present. Biased migration and pattern formation responses are of particular interest in microrobotic swarm design. Collective behavior achieved through broadcasting a signal that elicits a response from the entire microrobotic swarm is known as “centralized control”. In early works, Whitesides’ group harnessed phototaxis (*i.e.*, migration to higher light intensity) response in photosynthetic algae *Chlamydomonas reinhardtii* to steer microparticle-carrying algae using light as an input.^94^ Behkam’s group established a chemoattractant concentration field to guide *S. marcescens*-propelled microparticles to the chemoattractant source.^121^ Later, they investigated the role of microrobot geometry and the chemoattractant’s spatiotemporal distribution on the swarm’s collective behavior.^821,822^ Martel’s group showed the assembly of a miniature version of the Pyramid of Giza by centralized control of a swarm of thousands of magnetotactic bacteria.^120^

### 4.3 Further Development of Microswarms

The microswarm composed of a large number of individual micro/nanorobots gains enhanced capabilities and additional functionalities through collective behaviors.^105,823,824,825^ Compared to single micro/nanorobots, microswarms can load more cargo, move at faster speeds, provide higher contrast imaging, and generate greater forces. Building upon research on microswarm construction, the modalities and functionalities of microswarm need further development to adapt to intricate application environments and meet complex task requirements.

#### 4.3.1 Pattern Transformation

Artificial micro/nanorobot swarms typically possess reconfigurable characteristics, enabling them to transform their morphologies akin to natural swarms.^59,776^ The pattern transformation endows fixed-form micro/nanorobots with enhanced flexibility, granting them even more prominent deformation capabilities than those of some soft robots. Consequently, despite the overall size of microswarms far exceeding that of individual micro/nanorobots, they can still adapt to complex and confined environments.

The pattern transformation of a microswarm can occur actively or passively. Active pattern transformation refers to altering the swarm morphology through the adjustment of external inputs. For instance, the vortex-like microswarm composed of paramagnetic nanoparticles exhibits reversible pattern transformation behaviors, such as expansion, extension, and contraction when the parameters of the rotating magnetic field are adjusted.^373,808^ The large-scale reversible elongation and shrinkage of oscillating magnetic field-driven ribbon-like microswarms are achieved by changing the field ratio.^775^ This mechanism is used to mimic the structure and function of ant bridges to realize applications in the electronics field.^779^ Furthermore, changes in external inputs can also lead to different swarming states, manifesting as a multi-modal microswarm system corresponding to different input parameters. Janus colloidal microspheres display different swarm modes in alternating electric fields of various frequencies.^517^ Reversible switching between liquid, chain, vortex, and ribbon-like states of Fe_2_O_3_ colloidal particle swarm can be achieved by changing the type of input magnetic fields (Figure 8a).^783^ The passive pattern transformation of a microswarm is dominated by the external environment, with no significant changes in inputs. For instance, the optical field-driven microswarm performs cordon, splitting, and re-joining behaviors when it bypasses a prism obstacle, and can adaptively contract to pass smoothly when entering a narrow channel (Figure 8b).^113^ The pattern of a magnetic array-driven microswarm changes upon contact with the boundary, but strong internal repulsive interactions preserve the orderliness of the swarm pattern.^773^

**Figure 8:**
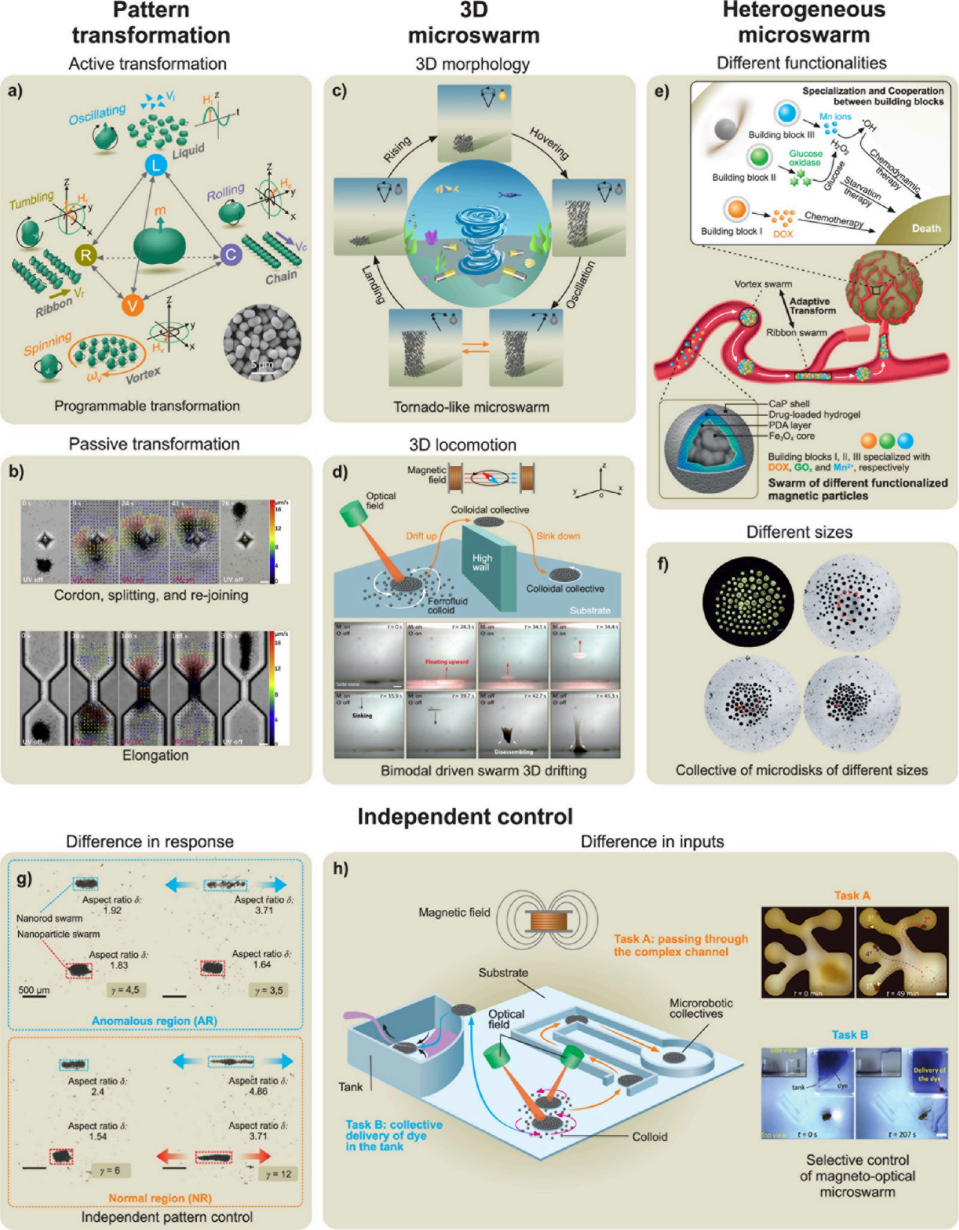
**Expansion of modalities and functionalities of microswarms. a)** Active pattern transformation of Fe_2_O_3_ colloidal particle swarm through adjusting input magnetic fields. Reproduced from ref 783, Copyright 2019 American Association for the Advancement of Science. **b)** Passive pattern transformation of a light-driven microswarm due to contact with boundaries. Reproduced from ref 113, Copyright 2019 Elsevier. **c)** Tornado-like microswarm with 3D morphology. Reproduced from ref 835, Copyright 2020 American Chemical Society. **d)** 3D drifting of a microswarm underwater based on a bimodal actuation strategy. Reproduced with permission under a Creative Commons CC-BY License from ref 837, Copyright 2023 American Association for the Advancement of Science. **e)** Heterogeneous microswarm consisting of different functionalized Fe_3_O_4_ nanoparticles. Reproduced from ref 838, Copyright 2021 WILEY-VCH. **f)** Heterogeneous microswarm formed by microdisks of different sizes. Reproduced with permission under a Creative Commons CC-BY License from ref 842, Copyright 2023 National Academy of Sciences. **g)** Independent morphological control achieved based on different responses of microswarms to the magnetic field. Reproduced from ref 843, Copyright 2021 American Chemical Society. **h)** Selective control of microswarms realized based on nonuniform external inputs. Reproduced from ref 844, Copyright 2024 WILEY-VCH.

Overall, the pattern transformation ability of microswarms stems from the fact that the interactions among building blocks are adjustable rather than fixed. From both theoretical and practical perspectives, further progress needs to be made in the study of microswarm pattern transformations, including achieving more types, greater degrees, and faster speeds of pattern transformations.^778^ The key lies in the meticulous design of external inputs and building blocks to regulate internal interactions.

#### 4.3.2 3D Microswarm

To date, most micro/nanorobot swarms have been constructed on 2D interfaces and possess 2D morphologies. However, natural swarms typically have 3D morphologies and can move freely in space. Constructing 3D microswarms contributes to the comprehension of natural collective behaviors from a microrobotic perspective. Moreover, 3D swarms can exert influence over an entire space more effectively than their 2D counterparts, which is advantageous for specific practical applications, such as embolization.^786,826^

The construction of 3D microswarms encompasses two significant aspects: developing microswarms with a 3D morphology and achieving their 3D locomotion. Overcoming gravity and facilitating vertical self-assembly of building blocks are crucial for 3D swarm generation. By enhancing the interaction among building blocks, microswarms can stand on the substrate with a structure akin to an erected thin sheet.^777,827,828,829^ Even though these types of microswarms retain a 2D structure, they provide novel perspectives for swarm systems to overcome gravity and achieve dimensions in the vertical direction. 3D self-assembly is an effective method to obtain microswarms with 3D structures.^830,831,832,833^ Recently, it has been reported that magnetic microparticles are assembled into 2D thin sheets by in-plane oscillating magnetic fields and further curled into hollow 3D microtubes.^834^ The hybrid field-driven method is also applied to constructing 3D microswarms. Acousto-magnetic field-driven microswarms exhibit spherical 3D morphologies rather than 2D thin sheets.^491^ A tornado-like microswarm driven by optical and magnetic fields is a typical example of a 3D microswarm (Figure 8c).^835^ The rotating magnetic field serves to gather magnetic nanoparticles while the photothermal effect produced by laser irradiation generates convection, inducing nanoparticles to further gather toward the center and rise vertically.

Enabling microswarms to overcome gravity and achieve 3D locomotion with an intact pattern has long been a major challenge in this field. Vertical thin sheet-like microswarms can perform efficient translational movements, enabling them to overcome vertical obstacles or ascend along an inclined substrate.^776,828,829^ Externally applied forces, such as magnetic and acoustic radiation forces, can cause microswarms to rise or even move against the flow in inclined tubes.^132^ Although these microswarms are still unable to detach from the substrate, they can achieve vertical displacement, suggesting potential for applications within complex 3D lumens in the human body. Microswarms composed of micro/nanorobots with inherent three-dimensional movement capabilities, such as helical swimmers, can move freely in space.^54,836^ However, their lack of internal interaction constraints poses challenges for precise localization and navigation. Another example of the free 3D motion behavior of microswarms is achieved by using thermal convection. A bimodal actuation strategy for artificial colloidal systems is proposed, *i.e.*, combining magnetic and optical fields (Figure 8d).^837^ The magnetic field triggers the self-assembly of magnetic colloidal particles to form a microswarm, maintaining numerous colloids as a dynamically stable entity. The optical field allows the colloidal microswarm to generate convective flow through the photothermal effect, enabling it to use fluidic currents for 3D drifting.

#### 4.3.3 Heterogeneous Microswarm

The current micro/nanorobot swarms primarily consist of similar building blocks, resulting in limited functionalities. To expand the capabilities of microswarms and enable them to perform multiple tasks, two strategies can be employed. One approach is to enhance the functions of individual building blocks, although this presents challenges in terms of intricate design and complex fabrication at the micro- and nanoscale.

The alternative method involves constructing heterogeneous microswarms. By using different functionalized Fe_3_O_4_ nanoparticles, microswarms with heterogeneous structures can be formed, enabling synergistic therapy through domino reactions (Figure 8e).^838^ Similarly, a heterogeneous sensor-carrier microswarm has been developed, in which sensor-bots exhibit pH mapping capability through structural-color changes in response to pH variations, while DOX-loaded carrier-bots achieve selective adhesion to acidic targets and drug release through pH-responsive charge reversal.^839^ Under synchronization conditions, different micro/nanorobots can form a stable heterogeneous microswarm through local hydrodynamic interactions. In contrast, under desynchronization conditions, the microswarm undergoes a phase segregation process and spontaneously forms a leader-follower hierarchical structure.^840^ In an AC electric field, the utilization of microparticles with varying sizes and dielectric properties leads to the formation of hierarchical leader–follower-like microswarms.^815^ Similarly, the combination of two species of passive microparticles exhibits predator–prey swarming behaviors.^841^ Magnetic microdisks of varying sizes can form heterogeneous microswarms at the liquid–air interface, and the size of the microdisks directly correlates with the aggregation, dispersion, motion, and morphology of the microswarm (Figure 8f).^842^

Heterogeneous microswarms can be endowed with diverse functionalities, which enables them to efficiently perform multiple tasks. It is important to note that heterogeneous microswarms go beyond simply mixing different building blocks. A comprehensive understanding of the properties and interactions among the building blocks is crucial in the design process. Additionally, the functions of each component within a heterogeneous microswarm, such as sensing, actuation, loading, and treatment, should be clearly defined. In summary, the construction of heterogeneous microswarms provides an effective strategy for developing multifunctional robotic systems at small scales without the need for sophisticated equipment or high costs.

#### 4.3.4 Independent Control

In the research domain of microswarms, the topic of independent control over micro/nanorobot swarms holds considerable significance. The capability to independently govern multiple microswarms under the same input bestows upon the entire swarm system the capability to undertake an array of tasks concurrently and amplify its functionalities through the synergy of diverse microswarms. A viable method for independent microswarm control is the application of building blocks with different structures or attributes, which consequently induce differential responses to a consistent input field. It is reported that two ribbon-like microswarms, composed of Ni nanorods and Fe_3_O_4_ nanoparticles, respectively, exhibit different pattern transformation behaviors within the specified magnetic field parameter intervals.^843^ The difference in the pattern transformation behavior of these two types of microswarms is used to achieve independent control of their morphologies (Figure 8g). This study demonstrates that microswarms composed of different building blocks can exhibit diverse collective behaviors under the same input. This approach can be further employed to achieve independent motion control of microswarms, which bears substantial significance for practical applications. Additionally, hybrid field actuation is also suitable for achieving independent control, given the more pronounced discrepancy in the response of building blocks to diverse applied fields. Recently, a selective control strategy for microswarms that combines light and magnetic fields has been developed (Figure 8h).^844^ The magnetic field serves to stimulate the formation of microswarms from colloidal particles while the light generates convection that drives the 3D motion of the microswarm through the photothermal effect. Thanks to the small influence area of the light spot, several microswarms can be selectively driven in sequence to complete different tasks. During operation, the continuously applied global magnetic field plays a crucial role in maintaining the integrity of the swarm pattern.

#### 4.3.5 Cooperative Task Completion

In biohybrid microrobots, the biotic component is endowed with rapid and species-specific agent–agent communication machinery, which can be harnessed for cooperative task completion. For example, bacteria use low molecular weight, highly diffusive chemical communication signals (known as “autoinducers”) to count the number of neighbors. This attribute enables them to exhibit a population density-dependent cooperative response called “quorum sensing”. Behkam’s group used synthetic biology approaches to build quorum sensing circuits with different sensitivity levels.^845^ Later, they developed a multiscale data-driven statistical model that predicts emergent behaviors of bacterial biohybrid systems. They showed genetic circuit sensitivity and spatial organization of the agents strongly influence the response time and robustness of the emergent behavior.^540^ They also showed that quorum sensing could be leveraged for decentralized control of microrobotic swarms in targeted cancer therapy, wherein each agent interrogates its immediate environment and makes independent decisions about therapy release based on its interaction with other agents and its local environment.^540^

Cooperative behavior has also been observed across many species of spermatozoa and is thought to be leveraged by sperms to enhance their migration to the fertilization site. This cooperative behavior is characterized by bundle formation, where sperm heads overlap, attach, and swim together. It was observed experimentally that most sperms bundle into pairs and synchronize their beat parameters after a transition period to achieve more efficient swimming.^846,847^ The underlying mechanisms have not been thoroughly investigated yet. Preliminary studies suggest that viscosity does not affect bundle formation but that the level of motility and the biochemical state of the sperm cells, which affect the membrane composition and cell–cell adhesion, directly affect this cooperative behavior.^847^ Once more insight is gained into the underlying mechanisms of this cooperative behavior of sperm, it is envisioned that these strategies can be transferred to the design of biohybrid and bio-inspired microrobots, *e.g.*, integrating multiple synchronized flagella for more efficient swimming and the ability to pair and single-out on demand.

## 5 Intelligence

Realizing intelligence and building more smart machines are consistent endeavors for the whole scientific community beyond micro/nanorobots. Inspired by human intelligence, the realization of nanorobot intelligence may also be multifaceted, where off-board or on-board intelligence may be applied independently. More importantly, the intriguing swarming behaviors observed in micro/nanorobots imply that these minuscule machines may be constructed to accomplish intricate tasks. Despite the limited demonstration of such an intelligent swarm, it offers a promising way to explore the potential intelligence these micro/nanorobots embody.

### 5.1 Intelligence and Embodied Intelligence

“Intelligence” is challenging to define, yet it consistently relates to the capacity for perceiving, storing, processing, and adapting to information. Traditional theories view intelligence as an agent’s ability to adapt to the environment using cognitive processes rooted in the brain (Figure 9a). The brain’s collective intelligence enables complex problem-solving, and mimicking this network structure in artificial neural networks has demonstrated impressive abilities, sometimes surpassing human brain performance. Broadly speaking, autonomous behavior, therefore, can be described as the ability of the agent to perform on its own by utilizing external inputs but without the need for the external guidance of a separate agent. It is worth noting that in many cases, the autonomy of biological agents is maintained even if they perform collectively in which individual agents are a part of a larger group. Examples of such behavior, known as “swarming”, can be seen in bird flocks, fish schools, and bacteria swarms. Effective operation within a swarm or eliciting function by a swarm indeed illustrates higher-level examples of embodied intelligence, which are elaborated in later sections.^848,849,850,851,852^

**Figure 9:**
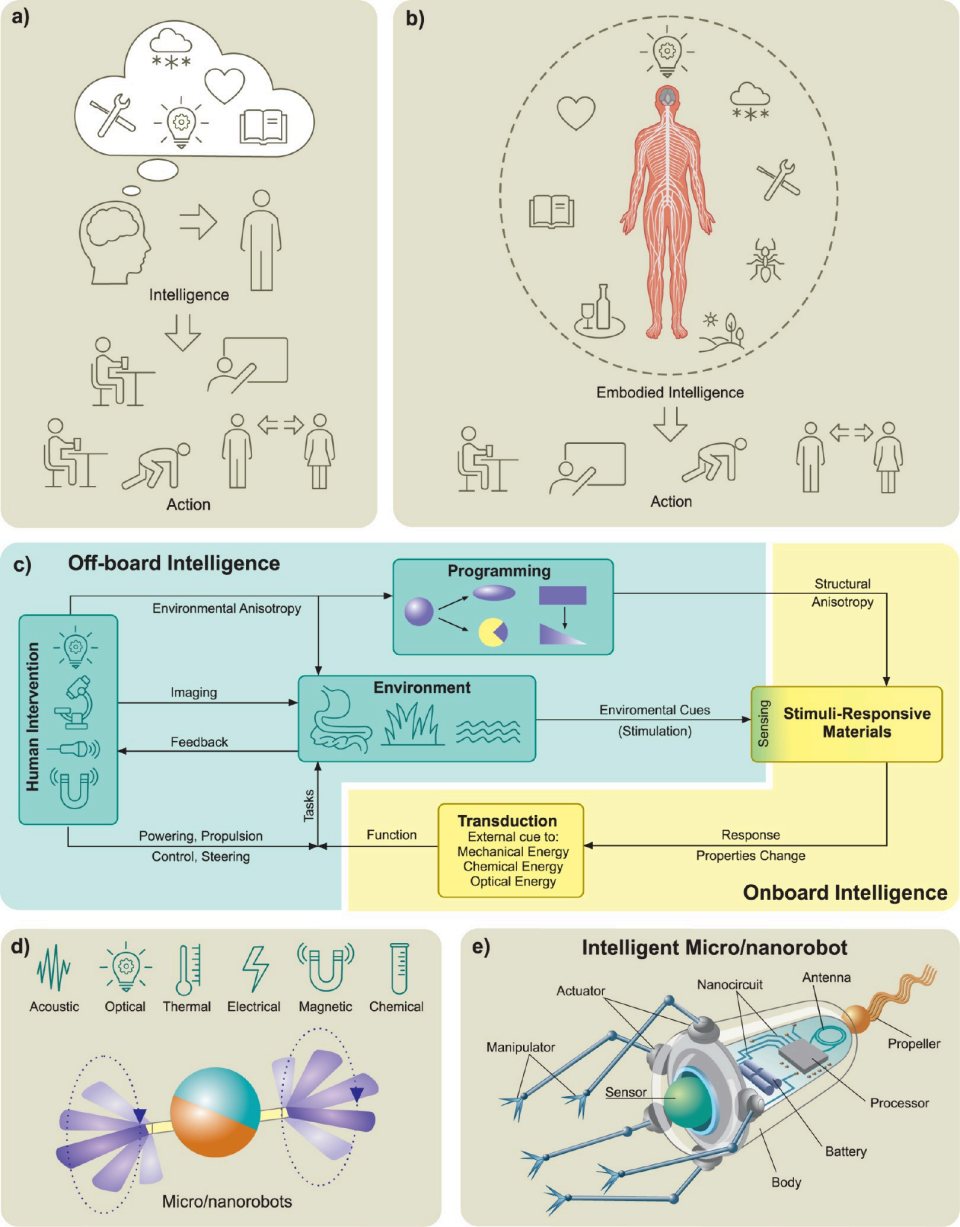
**Concept of intelligence. a)** Traditional understanding of intelligence which is mainly rooted in cognitive and rational processes taking place in the brain. There is a duality between brain and body when they interact with the environment; **b)** Embodied intelligence in which the integration of environment, brain, and body is necessary for cognition. **c)** Onboard and off-board elements of embodied intelligence at small scales. **d)** Embodied intelligence in stimuli-responsive materials for micro/nanorobots. **e)** holy grail for the field of micro/nanorobotics.

Embodied intelligence theories, however, challenge the notion that cognition is purely brain-centered. They emphasize the interaction between the body, brain, and environment, where sensory, motor, and perceptual systems all play crucial roles in cognition. This approach integrates the body and environment into cognitive processes, viewing them as interconnected (Figure 9b). The degree of reliance on bodily or cognitive aspects varies depending on the complexity of the brain and body. Humans and animals with advanced nervous systems process cognition differently than simpler organisms like unicellular organisms, plants, and invertebrates with decentralized systems (*e.g.*, jellyfish). In such organisms, cognition is domain-specific, decentralized, and relies on local knowledge structures, allowing for quick access to achieve specific goals, unlike centralized cognition in humans.^848,853,854,855^

#### 5.1.1 Molecular Communication

For embodied intelligence, it is challenging to directly use wireless signals or electromagnetic waves between microrobots for communication. However, molecular communication, widely adopted in living systems, can be employed for communication among micro/nanoscale robots. This method leverages chemical signals to communicate, akin to biological cells. This innovative approach enables precise and efficient coordination among swarms of tiny robots in environments where traditional electromagnetic communication fails, such as inside the human body. This communication method also takes advantage of the natural diffusion of chemicals, resulting in minimal energy consumption and enhanced compatibility with biological systems, making it ideal for medical applications such as targeted drug delivery and complex microsurgery. Furthermore, molecular communication can facilitate robust, adaptive responses to dynamic changes in the environment, significantly enhancing the functionality and versatility of microscale robotic networks. For example, it has been demonstrated that a chemical “message” can be sent from a moving activator motor to a nearby activated (receiver) motor by releasing Ag^+^ ions from a Janus polystyrene/Ni/Au/Ag activator motor to the activated Janus SiO_2_/Pt nanomotor.^788^ The transmitted Ag signal rapidly translates into a dramatic speed change associated with the enhanced catalytic activity of activated motors. Selective and successive activation of multiple nanomotors is achieved through sequential localized chemical communications. The concept of establishing chemical communication between different synthetic nanomotors paves the way for intelligent nanoscale robotic systems capable of cooperating with each other.

#### 5.1.2 Physical Intelligence

Physical intelligence, as demonstrated through soft and deformable bodies, characterizes natural machines ranging from large mammals to single-celled organisms. These organisms leverage soft materials to adaptively interact with their environment without relying on precomputed processes. Inspired by these natural soft machines, employing materials like thin films, polymers, and hydrogels holds promise for fostering dynamic interactions between a robot and the environment for rapid decision-making.^856,857^ Due to distributed actuation of the soft body, soft microscale robots can automatically adapt to various geometries and environments, reacting more effectively without computation. For instance, researchers have demonstrated magneto–elastic small-scale soft robots made from magnetically responsive elastomers to be capable of versatile movements.^858^ These robots swim inside and on the surface of liquids, scale liquid menisci, roll and walk on solid surfaces, jump over obstacles, and navigate narrow tunnels. They seamlessly transition between liquid and solid terrains and can switch between different locomotion modes. Moreover, they perform tasks such as pick-and-place and cargo release. Theoretical models are also provided to elucidate the mechanisms underlying their movements. Similar to large-scale robots used for studying locomotion, these small-scale soft robots could be employed to investigate the soft-bodied locomotion observed in small organisms. Shape-deformable micromachines can also be constructed using thin-film structures. For example, researchers have developed a method to encode multiple shape-morphing commands into micromachines by controlling the magnetic states of arrays of single-domain nanomagnets on interconnected panels.^859^ This programming involves applying a precise sequence of magnetic fields to nanomagnets with customized switching characteristics, resulting in specific shape changes in the micromachines when subjected to an applied magnetic field.

### 5.2 Intelligence at Small Scales

Artificial intelligence and autonomy in micro/nanorobots can be achieved only if one considers them as complex microsystems capable of independently accomplishing specific goals or missions in an inherently uncertain and highly variable microscale world, where many of the physical effects of energy dissipation, electromagnetism, fluid dynamics, *etc*. become nonintuitive. From the machine intelligence standpoint, a robot must possess all the necessary functions, analogous to a biologically intelligent counterpart, which not only allows it to run automatically but also enables the robot to perceive and respond to the world around it. For this, it is necessary to merge many components, such as mechanisms, sensors, controllers, actuators, power sources, and interfaces, into a single but complex system.^30,860^ Traditional macroscale robots, which are more analogous to humans and animals in terms of form and function, have the luxury of space for the incorporation of all such components (Figure 10). These components are usually rigid and bulky and rely on rather sophisticated processing units and computers for their cognition and operation. Micro/nanorobots, on the contrary, are artificial analogs of microorganisms that are generally soft, small, and lack sophisticated nervous systems; indeed, micro/nanorobots mainly rely on bodily aspects of artificial embodied intelligence. Achieving artificial embodied intelligence on small scales is extremely challenging due to the engineering trade-offs between miniaturization and intelligence. Such a trade-off makes it extremely hard to effectively integrate all functional components in a single small-scale robot.^860,861,862^

**Figure 10:**
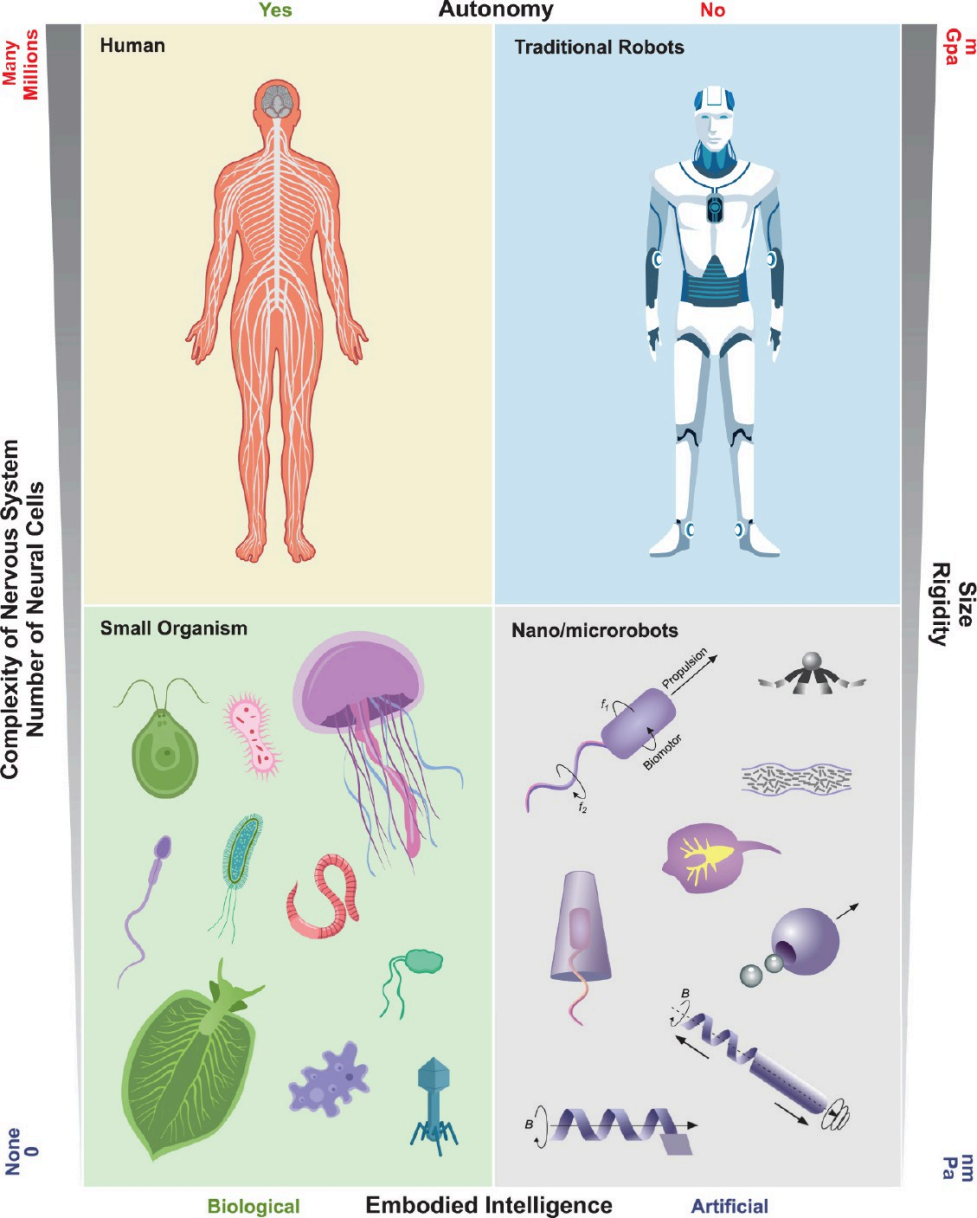
**The landscape of embodied intelligence in biological and artificial agents.** The size of the biological and artificial agents is directly proportional to the complexity of their nervous system.

Considering such limitations, scientists in the past two decades have devised many innovative strategies that give small-scale robots artificial embodied intelligence and functionality. In general, two alternative strategies have been used to create intelligent micromachines depending on the size of the robots and the viability of integrating a multitude of functional components in a small-scale agent: the *top-down* and *bottom-up* approaches.

#### 5.2.1 Top-Down and Bottom-Up Approaches

In the *top-down* approach, small-scale robots are essentially viewed as integral system frameworks with embodied intelligence and the goal becomes the miniaturization of this system to ensure their versatile functionalities even at smaller scales (Figure 9e). Centimeter and millimeter-scale robotic systems, such as insect-inspired micromachines,^863,864,865,866,867,868^ soft-bodied machines,^858,868,869,870,871^ and multi-agent robots^872,873^ are some examples developed through this approach. It is noteworthy that nowadays, the evolution of micro/nanofabrication technologies has also led to the emergence of intelligent micrometer-scale robotic systems. One method for creating such microrobots with integrated functionalities is to combine CMOS-compatible microfabrication techniques, selective lift-off methods, and strain engineering to create 3D microstructures from functional materials. Various functional 3D microcomponents, such as sensors,^874,875,876^ actuators,^877,878,879^ and power sources^880^ have been developed through this method. A big advantage of the CMOS-compatible top-down approach is its feasibility for standardization of the procedure, upscale fabrication, and facile integration of multifunctionality. For example, You *et al*. recently reported highly integrated miniaturized reconstructive spectrometers with an automatic performance-optimizing algorithm, which could be used for robots’ visual perception.^881^

In the *bottom-up* approach, miniaturization of single-functional components is the starting point. This is followed by augmenting their functionality to the point where embodied intelligence is achieved. *Bottom-up* approaches, indeed, resemble biological processes such as cell reproduction, self-assembly, morphogenesis, and growth. Small-scale soft walkers,^882^ swimmers,^504,883^ and some of the biohybrid robots^884^ are examples of robotic systems developed through this approach. Achieving embodied intelligence through *bottom-up* approaches at micro/nanoscale requires the most advanced nanotechnology tools and techniques to endow foundation building block materials with capabilities such as mobility, transduction, adaptability, programmability, memory, wireless power transfer and communication, and data processing and computing.^860^

#### 5.2.2 On-Board and Off-Board Intelligence

In the majority of reported micro/nanorobotics systems, whether realized by *top-down* or *bottom-up* approaches, the cognitive and bodily aspects of artificial embodied intelligence have been physically separated. Typically, the control over locomotion, assembly, and on-demand transformation of micro/nanorobots is accomplished remotely through established wireless engineering techniques. These processes can be deemed as cognitive aspects of artificial embodied intelligence at small scales, which are referred to here as “off-board intelligence” (Figure 9c). The bodily aspects of artificial embodied intelligence of micro/nanorobots mainly include their ability to sense environmental cues and transduce those cues to desirable functionalities. These features must all be embedded within the small-scale robots’ building block materials to create “on-board intelligence”.^860,885,886,887,888^ The application of AC electric field-powered propulsion in motile semiconductor circuits opens an interesting route for the future fabrication of microrobots that can perform multiple autonomous functions, including motility, sensing, logical operations, and biological interfacing.^380,564,889,890^ An example of such a device has been reported by Han *et al*.^533^ This particle may self-propel by harvesting electric energy from an external electric field along the x-axis. A magnetic patch can be used to impart torque for steering, in this case, *via* an external magnetic field in the x/y-plane. The external electric field rectified by a microdiode can be used to power up internal electrical circuits on microchips. Microsensors can be used to detect and respond to external stimuli (*e.g.*, light). Bioaffinity binding sites (*e.g.*, for antibodies or aptamers), can be used to impart biological or medical functionality. Thus, the particle-circuits may serve as a self-propelling biosensor or biomedical bot with internal logic and potential communication capabilities. Early examples of motile microcircuits powered by AC fields showcase their ability to move and assemble or disassemble on demand, highlighting some of these advanced capabilities.^530,891^

Embodied intelligence in micro/nanorobotics can be realized only if *on-board intelligence* and *off-board intelligence* are integrated. The requisite and the result of such an integration is the flow of information to and from micro/nanorobots while interacting with their environment, humans, and other micro/nanorobots. A two-way, or reciprocal, transfer of information from the output of one system to its input forms a feedback loop. Feedback loops represent the most essential feature of intelligent systems enabling them to perform multiple and adaptive tasks and deal with complex dynamic situations. In this context, embodied intelligence of micro/nanorobots can be achieved only if they possess all of the following features: (a) control components to perceive, interpret, process, and react to information gained from different sources; (b) adaptive mechanisms that can transmit or suitably modify some forms of information; and (c) feedback, which returns information to the system for creating interactions (Figure 9c).^860^

### 5.3 Smart Materials for Micro/Nanorobotics

Stimuli-responsive materials play a crucial role in the realization of on-board intelligence in the design of functional micro/nanorobots. Not only do these materials form the building blocks of micro/nanorobots, but they also facilitate their adaptation to and interaction with surroundings *via* response to environmental cues. External cues include but are not limited to, chemicals, heat, light, magnetic, electric, and acoustic fields, and are either innately present in the environment or remotely applied to the environment by human intervention through off-board intelligence. The responsiveness to external stimuli, in many instances, is inherent to the chemistry of the material itself, such as the sensitivity of acrylate-based hydrogels to changes in pH.^892^ However, responsiveness can also be induced in passive materials *via* the modification of their bulk or surface chemistry by stimuli-responsive materials, *e.g.*, magnetoactive composite elastomers.^893^ In Section 6, we will elaborate on the materials commonly used in micro/nanorobotics, including stimuli-responsive materials.

The response of stimuli-responsive materials to external cues can be essentially deemed as the transduction or the transformation of one form of energy to another, *e.g.* chemical energy to mechanical energy.^894^ Such responses manifest in the form of a change in materials’ chemical formulation,^895^ morphology, actuation,^896^ and/or their optical,^883^ magnetic,^897^ and electric^898^ properties (Figure 9d). In the case of taxis, the material responds to external cues by changing its location and moving toward or away from the source of stimuli.^104^ Stimuli-responsive materials indeed provide micro/nanorobots with a primitive form of the sensorimotor system that responds to environmental cues. By responding to changes in their environment, micro/nanorobots made from or containing stimuli-responsive materials can exhibit dynamic behaviors similar to the adaptive capabilities of living organisms.^899,900^ In some cases, the response of stimuli-responsive materials to external stimuli is reversible, meaning that their original properties will be retrieved when the stimuli are removed. Reversibility in stimuli-responsive materials, which is similar to the memory effect in biological agents, can be utilized as an ON/OFF switch and provides flexibility in the design of micro/nanorobots, especially those that must perform a desirable task over repetitive cycles.

Stimuli-responsive materials are pivotal in facilitating adaptation to dynamic environments, particularly in the bio-responsive materials field extensively utilized in drug delivery. These materials can response and adjust to environmental cues. For instance, microrobots can be encapsulated in biomaterials that dissolve only at specific body temperatures or in response to pH changes, such as those occurring in tumors or the gastrointestinal tract. This ensures that the microrobots are triggered and medications are released precisely at the target sites. This targeted approach reduces side effects and enhances treatment effectiveness, paving the way for a future where drug delivery is more intelligent and precise. For example, pH-responsive enteric micromotor systems are designed for precise positioning and controllable retention within specific segments of the gastrointestinal tract.^901^ These motors are composed of Mg-based tubular structures coated with an enteric polymer layer, serving as a reliable tool in nanobiotechnology for targeted gastrointestinal delivery. Upon dissolution of their enteric coating at the designated location, these micromotors initiate propulsion, enabling localized tissue penetration and subsequent retention to deliver payloads effectively. Such smart materials would enable the decision-making and response in complex biological environments.

While the interaction of living organisms with their environment is adaptive and autonomous, small-scale robots’ interaction with their environment is generally reactive, and not autonomous. In the majority of cases, the response of micro/nanorobots to external stimuli is programmed within their building block materials. For example, predetermined stimuli-responsive actuation profiles can be achieved through encoding molecular^902,903^ or morphological^895,904^ anisotropy within the stimuli-responsive materials, so-called structural anisotropy. In some cases, predetermined responses of materials to external cues are achieved through encoding gradients of stimuli and heterogeneity in the environment,^905^ so-called “environmental anisotropy”. These two methods together give a robust tool for programming the response of the micro/nanorobots upon exposure to the environmental cues. For example, researchers have developed a method to encode multiple shape-morphing commands into micromachines by controlling the magnetic states of arrays of single-domain nanomagnets on interconnected panels.^859^ This programming involves applying a precise sequence of magnetic fields to nanomagnets with customized switching characteristics, resulting in specific shape changes in the micromachines when subjected to an applied magnetic field.

Additionally, new technologies such as AI can be employed to regulate the operation of the micro/nanorobots, *i.e.*, sensing and transduction, which adds to the programmability toolbox.^53^ For example, the utilization of path programming algorithms and machine learning has made it possible to implement directional and collision-free movement, facilitating the targeted function of the microrobot in confined and tiny spaces at hard-to-reach locations, where collision between them may adversely affect their operation.^885,906,907,908^

Like microorganisms, micro/nanorobots may need to form into swarms to be able to perform a desired task. For example, for many applications, such as drug delivery, the response of individual micro/nanorobots is infinitesimal and not impactful. In these instances, the response of swarms will have a significant effect. Structural and environmental programming, hand-in-hand with off-board AI tools, can be utilized to engineer the interactions between individual micro/nanorobots, by predicting or regulating swarm formation. Introducing local asymmetry in the flow, inducing magnetic or electric dipole–dipole attraction between the adjacent micro/nanorobots, utilizing Bjerknes forces under the influence of an ultrasound field, and employing diffusiophoretic forces are among the common methods used for implementing the swarming behavior.^909^

### 5.4 Intelligent Functionality for Micro/Nanorobotics

In small-scale robotics, programmed stimulation—representing off-board intelligence—and predetermined responses from stimuli-responsive materials—representing on-board intelligence—can be integrated to achieve meaningful functionalities that enable predefined tasks. Here, we classify the functionality of micro/nanorobots based on the nature of the transduction or transformation of incoming stimuli, focusing on: 1) mechanical adaptation, including changes in shape, microstructure, or position, 2) chemical recognition, such as breaking existing or making new interatomic/molecular interactions, and 3) optical burst, such as fluorescence and quenching. Micro/nanorobots utilize each or a combination of these functionalities to perform delicate and targeted tasks in tiny environments. These tasks include cargo transport, chemical sensing/biorecognition, imaging, tracking, and visualization, to name a few (Figure 11).

**Figure 11:**
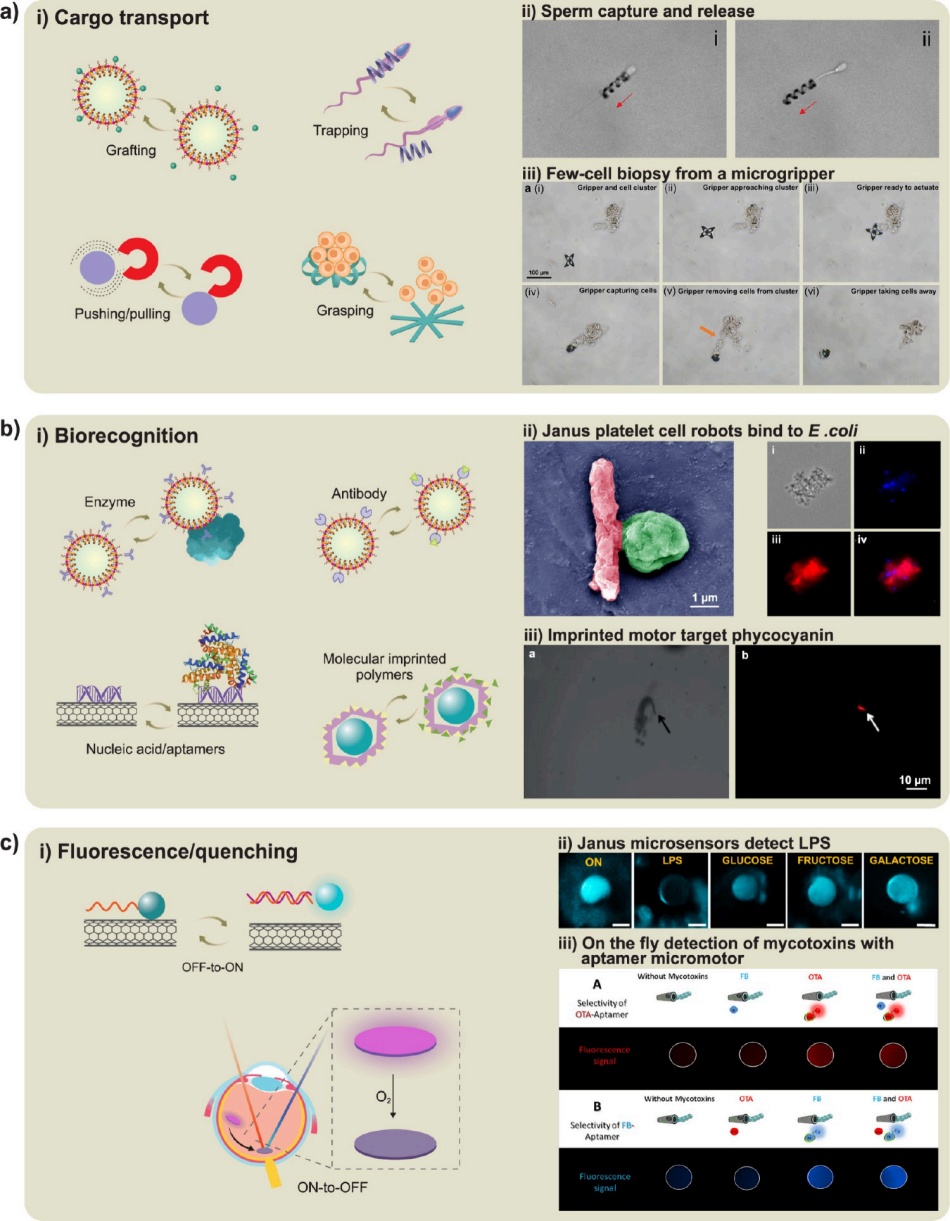
**Snapshot of three major functionalities of micro/nanorobots rooted in embodied intelligence at small scales. a)** Cargo transport by means of mechanical adaptation. Panel (ii) is reprinted with permission from ref 910, Copyright 2016 American Chemical Society. Panel (iii) is reprinted with permission from ref 911, Copyright 2020 American Chemical Society. **b)** Biorecognition by means of chemical recognition. Panel (ii) is reprinted with permission from ref 36, Copyright 2020 The American Association for the Advancement of Science. Panel (iii) is reprinted with permission from ref 912, Copyright 2015 the Royal Society of Chemistry. **c)** Fluorescence and quenching by means of optical burst. Panel (ii) is reprinted with permission from ref from ref 913, Copyright 2018 American Chemical Society. Panel (iii) is reprinted with permission from ref 914, Copyright 2025 American Chemical Society.

#### 5.4.1 Mechanical Adaptation

Transduction of the external stimuli into different forms of mechanical energy in micro/nanorobots offers a promising solution for the manipulation and transport of small-scale objects in complex environments (Figure 11a). These microrobots transduce chemical or physical stimuli, such as pH gradients and magnetic fields, into mechanical energy, such as motion and shape-change, allowing for precise control over locomotion and transport. Just as the evolution of organisms in nature has led to more advanced forms of motion, micro/nanorobots also require such advancements to enable functionalities like seek-and-find and pick-and-place. The use of stimuli-responsive materials, including thin films, atomistic films, and gels, has opened up exciting possibilities for complex shape change in microrobots.^915,916,917,918,919,920,921,922^ In preliminary studies, researchers have shown how simple sequentially swelling and shrinking stimuli-responsive bilayers can locomote when placed on ratcheted or confined substrates and move preferentially along different directions based on the type of substrate patterning.^923^ Apart from the ratcheted pattern of the substrate, the shape of a microrobot itself, as enabled by the innovative patterning of domains or segments, can endow the advanced functionality of steering. Pantula *et al*. have shown that two 3D-printed thermally responsive bilayers of different dimensions connected by a flexible linker can break symmetry autonomously on heating and cooling cycles based on dissimilar contact areas with the substrate at different points along the microrobot.^924^ This study points to the importance of shape in endowing intelligence in modern robots. Elsewhere, magnetic patterning, such as by inclusion of nanoparticles has enabled magnetic field gradient-dependent steering, including complex micro-loop crawling, reminiscent of a caterpillar.^925^ Hu *et al*. have shown how multimodal locomotion with the capacity for swimming, climbing, rolling, and jumping can be achieved using magnetic field-induced torques and pulling forces.^858^ Scientists have also used photoactive polymers to generate directional motion in response to light.^883,926,927^ Despite significant progress, several challenges remain, including further miniaturization to micrometer and nanometer length scales, enabling autonomy and remote operation, and coupling sensing to steering to enable primitive intelligence seen in simple organisms and bilaterians, such as nematodes.^928^

Similar to steering, shape-change is an important concept that can endow significant functionality in small-scale and untethered robots. A variety of stimuli-responsive and external field-driven self-folding structures have been demonstrated.^925,929,930,931,932,933^ Shape change can endow functionality for pick-and-place (open and close), gastric obstruction, compact packaging, and biopsy. For example, untethered microgrippers composed of triggered pre-stressed thin film bilayers have been deployed for biopsy in hard-to-reach places such as the bile duct *in vivo* ^934^ and used to capture and transport small delicate cargo, including a single live cell.^883,895,911,935^ Elsewhere, unfolding and gripping drug delivery devices have shown the capacity to increase bioavailability and inject drugs through the gastrointestinal mucosa.^936,937,938,939^ Shape-changing devices have also been deployed for alternate surgical tasks, such as patching wounds in *in vitro* phantoms and complex sense–decide tasks.^940,941^ The micro/nanoscale patterning of heterogeneous materials can endow complex shape change. For example, Shi *et al*. have shown how segments of DNA-responsive polymerization gels when put together by lithography with microscale resolution can allow complex shape changes such as the transformation of alphabet shapes or even/odd numbers merely by the removal and addition of DNA hairpins.^942^ The use of biomolecules such as enzymes^943^ or DNA^944^ to trigger shape change can be important to achieve autonomy especially when coupled to biochemical and synthetic biology networks in the body. Apart from surgical and drug delivery tasks shape change can also enable locomotion at small scales.^945^ Also, bending and circumferential expansion and buckling can be achieved, which is important for biomimetics and shape-adaptive implants.^946^ Similar to steering, challenges abound mainly to enable autonomy, temporally programmed patterns of shape change, and enhanced complexity and function. In this regard, researchers can look toward the abundant shape-change processes in nature, including cellular division, phagocytosis, embryonic development, germination, and metamorphosis.

#### 5.4.2 Chemical Recognition

Biorecognition processes in microrobots can be categorized as the functionalities associated with the transformation of stimuli into chemical energy. These processes involve the specific capture of target analytes or bioanalytes by recognition elements, either biological or synthetic, such as antibodies, enzymes, or molecularly imprinted polymers (Figure 11b). The biorecognition process provides microrobots with the ability to selectively bind to biomarkers and other target molecules. This capability, combined with self- or externally propelled mechanisms, allows microrobots to perform a wide range of tasks. For example, many motile cells and single-celled microorganisms can perform chemotaxis, which refers to directed motion up or down a gradient in chemical concentration. Chemotaxis is a critical capability in many biological scenarios. For instance, it allows bacteria to seek nutrients and avoid predators, enables immune cells to pursue those same bacteria, and allows spermatozoa to find and fertilize an ovum, among others.^868^ Inspired in part by this utility, researchers have aimed to realize chemotaxis in artificial active matter systems. In practice, this is not trivial as artificial micro/nanorobots lack the sensing proteins, signaling pathways, and motion redirection capabilities that motile cells use to perform chemotaxis. Nevertheless, compelling evidence for chemotaxis has been presented in a few studies, for example with active droplets^869^ and some enzymatic systems.^858,870^ For phoretic Janus particles, which self-propel due to asymmetry in surface chemistry and/or properties, the theoretical criteria for observing chemotaxis were analyzed. It was found that chemotaxis primarily depends on the asymmetry in chemical activity and/or the phoretic mobility of the two opposing faces.^871^ Enzyme-driven particles are popular model systems for artificial chemotaxis^870,872,873^ as reviewed in ref 874. Note that the physics underlying enzyme-driven chemotaxis are not fully understood and remain the subject of ongoing research and debate.^875,876^

#### 5.4.3 Optical Burst

The transduction of environmental cues to optical signals is another functionality through which stimuli-responsive materials endow micro/nanorobots with on-board intelligence. This functionality becomes important in biomedical applications where the visibility of micro/nanorobots to existing medical imaging modalities. The visibility of mobile micro/nanorobots enables the real-time monitoring of their location and the efficiency of the tasks they carry out, such as drug release and cell manipulation (Figure 11c).^563,947,948^ Moreover, monitoring micro/nanorobots promises precise procedures in tiny workspaces where traditional invasive imaging techniques, such as angiography and laparoscopy are not applicable.^949^ Generally, the molecular structure on the surface or the bulk of micro/nanorobots must be modified to comprise compounds visually detectable by noninvasive imaging modalities such as ultrasound, X-ray, and magnetic resonance imaging (MRI), and computed tomography (CT) techniques.^950^

Fluorescent microscopy is, perhaps, the most common noninvasive imaging method for the monitoring of micro/nanorobots containing fluorophores. Luminescent nanoparticles,^951^ carbon nanodots,^952^ fluorescent-labeled aptamer-based^914^ and DNA-based^563^ targeting probes, and graphene quantum dots^953^ are some examples of materials used in micro/nanorobotic systems for incorporating fluorescence properties. Monitoring the fluorescence of micro/nanorobots can be achieved by tracking the changes in fluorescence intensity and lifetime over time upon interaction with the surrounding environment. This is typically done by embedding stimuli-responsive materials that enable the transduction of external cues to optical signals. Switching and maintaining the optical signals in the ON state is called “fluorescence” (OFF-to-ON) while switching and maintaining the optical in the OFF state is called “quenching” (ON-to-OFF). Fluorescence and quenching are essentially rooted in the intermolecular interactions between micro/nanorobots fluorophores and environmental cues, such as specific biomarkers and toxins. Additionally, techniques such as aggregation-induced emission (AIE) result in intensified fluorescent emission due to the cumulative effect of individual fluorescent components.

Integrating fluorescence properties with self-propulsion and biorecognition capabilities enables micro/nanorobots to perform real-time, rapid, and precise detection and monitoring of specific targets in a media, such as biomolecules or pathogens, bacteria, mi-RNA, and toxins, *etc.*, for applications such as theranostics and environmental remediation. This approach, also known as “on-the-fly” detection, promises opportunities for more sophisticated biomedical applications, such as early-stage cancer diagnosis,^954^ real-time monitoring of oxygen concentration in the retina for diagnosing hypoxia,^947,955^ detecting of traces of explosive compounds in aqueous solutions, and high-precision food poisoning detection.^955^

As an example of “on-the-fly” detection techniques, a tubular micromotor based on 2D MoS_2_ nanosheets/Pt was synthesized through an electrodeposition method.^956^ The microtube was surface-modified with fluorescent dye-labeled single-stranded DNAs (ssDNA) and FITC-aptamers for the recognition of miRNA-21 and thrombin biomarkers, which are overexpressed in tumor samples and regulate tumor growth, metastasis, and angiogenesis. The delocalized electron network of the MoS_2_ nanosheets facilitates π–π stacking interactions with dye-labeled nucleotides, resulting in rapid adsorption and instantaneous Förster Resonance Energy Transfer quenching of the dye tag. Additionally, the self-propeller micromotor shows motility through a bubble-generating mechanism in the presence of H_2_O_2_ fuel, enabling “on-the-fly” monitoring of the targeted biomarkers. Upon interaction of the micromotor with the tumor samples, ssDNA forms a duplex with miRNA-21, and the FITC-aptamer specifically recognizes the thrombin protein. This recognition results in the release of ssDNA and FITC-aptamers, thus recovering the fluorescence signal of the dye tag. The recovery of the fluorescence properties in this example indicates the presence of cancerous cell-associated biomarkers that can be utilized in diagnostic applications for cancer.

### 5.5 Collective Intelligence for Micro/Nanorobotics

The rise of artificial intelligence has opened the door to exploring the feasibility of intelligence emerging from synthetic material systems. To explore this possibility, it is beneficial to consider some examples of intelligent emergence in nature and computers. The human brain stands as the most prominent example of intelligence—a highly complex system that exhibits extraordinary processing power while operating through relatively simple neurological responses. Each human brain contains approximately 86 billion neurons, with each neuron having between 1,000 and 10,000 connections. This network is far more complex than even the most powerful supercomputer. One of the key challenges in science is to understand how intelligence and consciousness emerge from a neural network, despite each neuron possessing only limited computational power.

While only limited understanding of how intelligence and consciousness arise from this network, we do know that the intelligence of the brain is intrinsically collective. This characteristic allows humans to solve complex problems and make decisions that are beyond the capabilities of any individual neuron. The application of the complex network of brain architecture has been proven to be extremely successful. As shown in Figure 12a, artificial neural networks have demonstrated remarkable capabilities in mimicking the brain’s neural networks and, in some cases, surpassing the performance of the human brain. For the simplest neuron network architecture, the branching model, neurons are divided into different layers and connections are constructed between neurons in adjacent layers, which allows information to be processed as chemical signals.

**Figure 12:**
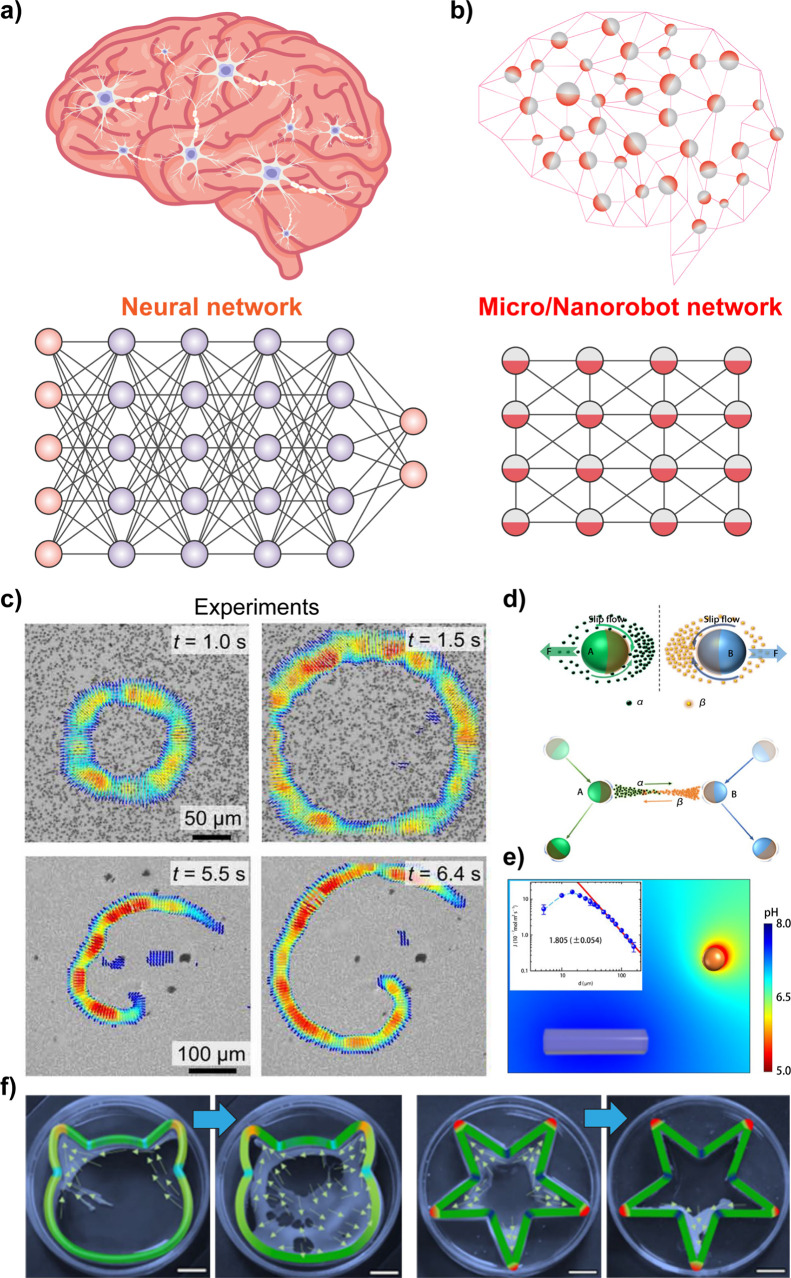
**From neural network to intelligent micro/nanorobot network. a)** Human brain modeled as a neural network in a branching model, where information is processed through layers of neurons. **b)** Envisioned intelligent micro/nanorobot networks connected through chemical interactions. **c)** Self-catalyzed reaction in AgCl/H_2_O_2_ system enables target wave and spiral wave. Adapted with permission from ref 959, Copyright 2022 the American Association for the Advancement of Science. **d)** Sulfonated polystyrene and ZnO can exchange ions, which couples two active particles as a chemically active swarm.^303^ **e)** Chemical reaction rate is sensitive to particle–particle distance, which creates feedback to regulate system activity.^303^ **f)** Active colloid swarm placed in an irregularly shaped container, demonstrating macroscopic phase separation and quorum sensing ability by moving toward one sharp corner in the macroscopic container. Scale bar is 1 cm. (d−f) are adapted with permission from ref 303. Copyright 2021 Springer.

Inspired by this neural network architecture, we may consider using micro/nanorobots to replace the nodes in the neural network to realize material intelligence^957^ as shown in Figure 12b. While this is still far from being materialized, we may list a few prerequisites for realizing this material intelligence:

1. Information propagation between micro/nanorobots.
2. Self-regulation and homeostasis in the micro/nanorobot network.
3. Adjustable connection weighting by training in the network.

In the human brain, information is processed in the form of an electric pulse, which is propagated by secreting and receiving neurotransmitters at synapses. In principle, any chemical signal may be applied as an information carrier as far as it can be maintained with sufficient time for data processing. However, as the chemical diffuses and the signal diminishes over time, it will be essential to amplify the chemical signal to maintain the propagation of chemical information.

Fortunately, in micro/nanorobots systems, self-catalyzed reactions are routinely used, which results in information propagation in the form of chemical waves. One example of such a case is the H_2_O_2_ decomposition reaction catalyzed by AgCl. In 2012, Sen’s group discovered the chemical wave of self-propelled AgCl particles in H_2_O_2_ under UV radiation.^789^ This chemical wave was extensively studied by Wei’s group.^958,959,960,961^ In this system, the H_2_O_2_ decomposition is catalyzed by Ag as decomposed from AgCl by UV light:

$$\begin{array}{l} 2\mathrm{AgCl}+H_{2}O\overset{\mathrm{UV light}}{\to }2\mathrm{Ag}+1/2O_{2}+2H^{+}+2{\mathrm{Cl}}^{-}\\ 2\mathrm{Ag}+H_{2}O_{2}+2{\mathrm{Cl}}^{-}\overset{\mathrm{UV light}}{\to }2\mathrm{AgCl}+2{\mathrm{OH}}^{-} \end{array}$$

The reaction regulates the local pH of the solution, which affects the reaction rate. As a result, the AgCl colloid particles generate a pH gradient that propagates through the solution as a chemical wave. As shown in Figure 12c, the target wave and spiral wave are both observed in this AgCl colloid system.

A similar chemical wave could potentially be harnessed for information transmission. With the implementation of appropriate regulations and rules, it may also pave the way for chemical computation to be realized.^962^ In this system, information is stored in the form of patterns of colloid particle composition, where the chemical reaction connects particles into a reaction network. Going forward, we will need more chemical micro/nanorobot systems with such chemical wave propagation ability and better controllability to enable data processing. To sustain effective information processing, chemical reactions and particle–particle interactions must be maintained at an optimal level. Insufficient chemical interactions hinder information propagation while excessively strong interactions render the system overly sensitive, allowing minor noise to amplify into system-wide fluctuations. Both extremes are detrimental to the system’s information processing capabilities.

Recently, Tang’s group discovered that the ion-exchange reaction can sustain strong interactions in colloidal particles and that the rate of this chemical reaction can be effectively regulated.^303^ As shown in Figure 12d, this system is composed of two chemically active colloid species, acidic sulfonated polystyrene and ZnO, coupled by simple acid–base neutralization reaction by exchanging H^+^ and Zn^2+^ ions through the solution. Importantly, the reaction rate is distance-dependent, where closer particle–particle interaction leads to a stronger exchange reaction and stronger coupling (Figure 12e). In this system, the reaction rate is self-regulated, which helps maintain the balance of chemical interactions.

Interestingly, this system allows the active colloid solution to show distinct macroscopic phase segregation and form an active swarm that shows decision-making abilities (Figure 12f) similar to the quorum sensing strategy seen in nature.^963^ While this ability is far from sophisticated intelligence, it suggests the feasibility of emulating intelligent behavior with micro/nanorobotic systems. However, to move forward, such a swarming system needs to self-regulate the interaction between individual active colloid units, which requires a fundamental understanding of the active phase behaviors similar to the human brain.^964^

### 5.6 Intelligence for Autonomous Control of Micro/Nanorobots

The necessity for autonomous control of micro/nanorobots, particularly in the biomedical field, is closely linked to the accuracy and precision required for specific procedures. Controlling an untethered micro/nanorobot involves not only precise actuation mechanisms but also robust and accurate control schemes.^885,965,966,967^ Given that micro/nanorobots often operate in dynamically changing environments via complex actuation mechanisms, manual or open-loop control becomes highly challenging.^968^ To address this issue, various closed-loop control methodologies have been employed to ensure accurate and robust navigation of micro/nanorobots.^969,970,971^

Proportional–integral–derivative (PID) controller is the most widely used type of closed-loop controllers. Its popularity and utility in robotics and various industries have also led to extensive applications in the field of micro/nanorobotics. For example, a PID controller has been used to achieve precise in vivo navigation of microrobots carrying stem cells in a nude mouse for cancer therapy.^969^ Additionally, proportional-integral (PI) controller has been utilized to stabilize microbubble oscillation, thereby minimizing damage to blood vessels.^972^ A PID controller has also shown effective performance in vivo, such as transporting mesenchymal stem cells into zebrafish yolk using magnetic microrobots.^973^ In practical biomedical applications, a PID controller can be also instrumental in the accurate control of magnetic microparticles with ultrasound feedback.^974^

While PID controllers are the most commonly used linear controllers in various control schemes, they are susceptible to nonlinear dynamics, e.g., Brownian motion, external fluid forces, and inaccuracies within the actuation system.^885^ To address these challenges, time-delay estimation (TDE) control has been proposed. TDE accommodates nonlinear magnetic actuation behaviors and offers improved response time and positional accuracy compared to traditional PID controllers.^975^ Additionally, TDE can be enhanced with an anti-windup scheme and a forgetting factor to further refine its performance.^976^

Adaptive control systems are critical for compensating errors caused by inaccurate mathematical modeling of both the system and its environment. They account for the non-Newtonian behavior of bodily fluids and the impact of electrostatic and contact forces. An adaptive fuzzy sliding mode control (AFSMC) is a model-free approach for positioning control of a magnetic microrobot. This method is capable of approximating uncertain forces and mitigating errors arising from unknown characteristics of the medium and limitations in imaging precision.^977^

In the sophisticated domain of soft microrobotics, advanced control schemes have also been applied. For instance, *Caenorhabditis elegans*, modified through optogenetic and biochemical techniques, can act as soft and highly controllable microrobots with excitable muscle cells.^978^ A predictive proportional controller has been successfully implemented for closed-loop control of this organism. Furthermore, a two-DoF system, which is commonly utilized in industrial servo applications, has been adapted for microrobot control to ensure robust performance amidst external disturbances and model uncertainties, particularly those arising from hydrodynamic effects.^979^

Since individual micro/nanorobots are often insufficient for most applications, the concept of swarming is often employed in the field.^980^ Controlling a swarm of micro/nanorobots presents more significant challenges than actuating individual micro/nanorobots. The primary difficulty lies in managing the interactions between the swarm and their fluidic environment, which can significantly influence shape formation and actuation. The collective dynamics of the swarm, including collision avoidance, synchronization, and cooperative task execution, introduce additional layers of complexity to achieve the intended goals during the applications. One strategy for managing these complexities is to utilization of Proportional-Integral (PI) controller integrated with an electromagnetic coil system and an ultrasound imaging system. This configuration has been successfully used to navigate microswarms within confined environments.^970^ For more stable control of the swarm, a fuzzy logic-based control scheme utilizes the control experience of an operator to navigate a magnetic microrobot swarm.^970^ This method is particularly beneficial in dynamic and unpredictable environments where rigid control algorithms might fail to adapt in a short interval.

Path planning is crucial for full autonomous navigation of micro/nanorobots, enabling them to navigate complex and often unpredictable terrains efficiently and effectively. The challenge of path planning comes from the necessity to navigate through dynamic and heterogeneous environments, which may include obstacles, varying fluid dynamics, biological particles, and tissue interfaces. Conventional path planning algorithms used in micro/nanorobotics include the A* algorithm,^981^ employed for the automated manipulation of magnetic microrobots (Figure 13a).^906^ A* iteratively evaluates potential paths using a cost function that accounts for both the distance traveled and the estimated distance to the target. This heuristic approach allows A* to effectively manage the complex navigation tasks required in micro/nanorobotics, ensuring that robots reach their destinations with minimal energy use and maximum precision.

**Figure 13:**
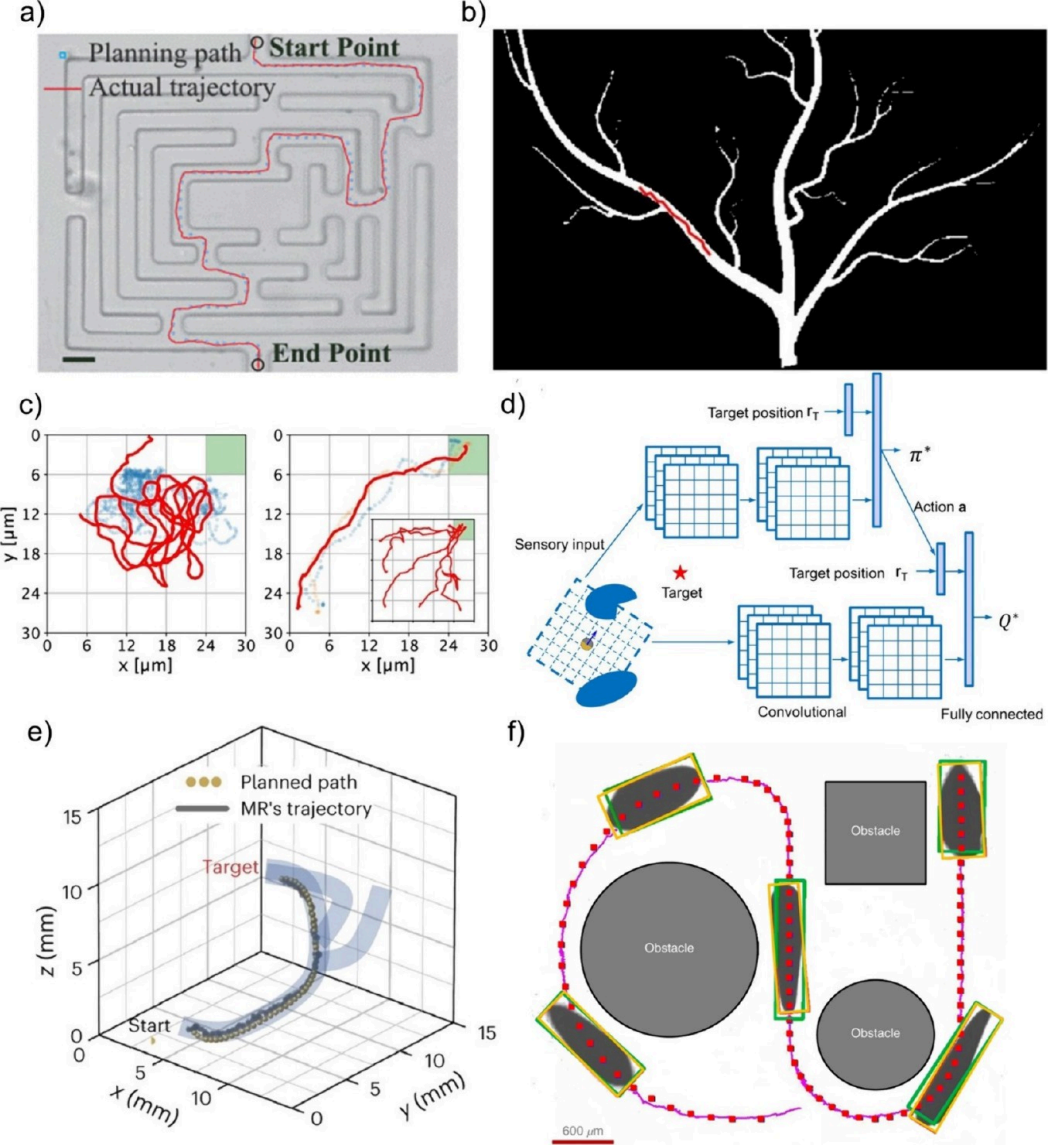
**Autonomous micro/nanorobot navigation via machine learning. a)** Path planning and navigation of a microrobot in a maze using the A* algorithm (scale bar is 20 μm). Reproduced from ref 906, Copyright 2018 IEEE. **b**) Simulation of path planning in a plant vein structure using RRT*-Connect. Reproduced with permission under a Creative Commons CC-BY License from ref 985, Copyright 2023 Frontiers. **c)** Learning process for the navigation of a self-thermophoretic microrobot using Q-learning. Reproduced from ref 971, Copyright 2021 American Association for the Advancement of Science. **d)** Neural network architecture of a DRL algorithm for the navigation of various micro/nanorobots. Reproduced with permission under a Creative Commons CC-BY License from ref 991, Copyright 2020 WILEY-VCH. **e)** Path planning and reinforcement learning for fully autonomous navigation of a magnetic microrobot. Reproduced from ref 967, Copyright 2024 Springer Nature. **f)** Supervised learning for autonomous navigation of a magnetic nanorobot swarm around obstacles. Reproduced from ref 994, Copyright 2022 Springer Nature.

Path planning optimization has also been explored using Particle Swarm Optimization (PSO).^982,983^ Inspired by the social behaviors of birds flocking or fish schooling, PSO adapts to changing conditions and identifies optimal paths even in the presence of strong currents or fluctuating fluid properties. Additionally, Rapidly Exploring Random Tree*-Connect (RRT*-Connect) algorithm^984^ has shown promise in navigating magnetic microrobots through environments that mimic plant veins (Figure 13b).^985^ RRT*-Connect combines the bidirectional nature of RRT-Connect,^986^ a sampling-based path planning algorithm, with the efficiency and stability enhancements of the RRT*-algorithm.^987^ When integrated with fuzzy PID controllers, RRT*-Connect facilitates smooth and efficient navigation by continuously adjusting the robot’s trajectory in response to real-time feedback. This integration highlights the potential for advanced path planning techniques to significantly enhance the navigational capabilities of micro/nanorobots in complex biological environments.

In the broader field of robotics, machine learning (ML) and artificial intelligence (AI) promise significant advancements over conventional methods.^988^ Similarly, groundbreaking research has been conducted in micro/nanorobot navigation and control using ML and AI.^885^ Three primary learning approaches are employed in robotics: supervised learning, which utilizes labeled datasets; unsupervised learning, where unlabeled data is employed; and reinforcement learning (RL), in which an agent learns to perform tasks by interacting with the environment and learning from the outcomes of its actions.^989^ RL has been actively used autonomous navigation of micro/nanorobots.^885^ For instance, as an RL algorithm, Q-learning has been implemented for the efficient navigation of colloidal micro/nanorobots.^990^ It was also used to autonomously actuate self-thermophoretic microrobots under dynamic Brownian motion (Figure 13c).^971^ Q-learning was employed to establish an optimal action selection policy based on the environmental state.^30^ Given transition states *s* and *s'*, actions *a* and *a'*, and reward *R*, a Q-matrix is created that includes the agent’s experiences. The Q-matrix is updated using the following equation:

5.1 $$Q_{t+\Delta t}\left(s,a\right)=Q_{t}\left(s,a\right)+\alpha \left[R\left(s^{\prime }\right)+\gamma \;\max\;Q_{t}\left(s^{\prime },a^{\prime }\right)-Q_{t}\left(s,a\right)\right]$$

The discount rate *γ* and learning rate *α* determine the value of future rewards and the rate of information update, respectively.^971^ Deep Reinforcement Learning (DRL), which combines RL with artificial neural networks, shows potential in navigating various types of micro/nanorobots (Figure 13d).^991^ A combination of local and global Q-matrices was used for the ultrasound-driven microrobot swarms.^992^ The local Q-matrix mitigates the overgeneralization induced by the global Q-matrix in a spatially and temporally dynamic system. The updates of the local Q-matrix and final Q-matrix are given as:

$$Q_{\mathrm{local}}\left[n+1\right]=\left(1-\alpha \right)\;Q_{\mathrm{local}}\left[n\right]+E\left[\alpha \;Q_{\mathrm{local}}\left[n+1\right]\right]$$ 5.2

5.3 $$Q\left[n\right]=\beta \;Q_{\mathrm{global}}+\left(1-\beta \right)\;Q_{\mathrm{local}}\left[n\right]$$

$E\left[\alpha \times Q_{\mathrm{local}}\left[n+1\right]\right]$ is calculated from an average number of past steps, and *β* represents the bias towards global behavior. RL has also proven effective in modeling adaptive behavior in complex flow states for navigating microswimmers.^993^ Fully autonomous control can be achieved by integrating an RL agent, specifically using the Proximal Policy Optimization (PPO) algorithm, with a path-planning system for real-time navigation. This combination allows the RL agent to effectively manage the actuation system while the path planning algorithm ensures optimal routing and navigation decisions are dynamically made (Figure 13e).^967^ PPO is based on the actor-critic algorithm, where the actor learns a policy *π* for decision-making, and the critic evaluates the decisions based on the value function *V*.

5.4 $${V^{\pi }}_{s_{i}}=\sum_{t}\gamma^{t}r_{\left(s_{i+t+1},a_{i+t+1}\right)}$$

where *r* is the reward given to the state and action. An optimal policy π* is achieved using an iterative process, and it represents the policy that provides the maximum cumulative reward defined by *V*. For complex tasks such as magnetic microrobot swarm navigation, supervised machine learning approaches have proven more efficient, robust, and faster compared to conventional methods (Figure 13f).^994^

Given the complexities of actuation, navigation, and dynamic environments, manual control of micro/nanorobots, can be impractical particularly for biomedical applications. Traditional closed-loop control systems, enhanced with ML and AI techniques, have successfully modeled the navigation of micro/nanorobots and understood the dynamics of their physical actuation systems.^967^ However, there remains a need for further research, particularly in developing a comprehensive end-to-end solution for navigation, path planning, and actuation control. ML algorithms are highly promising in this regard, potentially leading to robust systems capable of adapting to varied dynamic environments while minimizing errors in accuracy and reducing stress on the actuation systems. Furthermore, integrating closed-loop control with virtual reality (VR) enables highly intuitive and remote operations of micro/nanorobots.^995^ With advancements in VR technology and graphical processing units, the synergy of AI/ML-driven control with VR is poised to become a foundational technology for real-time autonomous control of micro/nanorobots in vivo. This approach could significantly enhance the precision and effectiveness of such devices in complex and sensitive environments.

## 6 Materials Design

The design of materials in micro/nanorobots is crucial for determining their propulsion efficiency and functionality as it enables precise control over locomotion, responsiveness to stimuli, and targeted task execution. In this section, we cover different types of materials utilized in the structure of micro/nanorobots with defined functions.

### 6.1 Material Requirements for Individual Micro/Nanorobots

#### 6.1.1 Materials for Propulsion and Functionality

##### 6.1.1.1 Material Design for Chemically Powered Micro/Nanorobots

Chemically powered micro/nanorobots constitute a category of small-scale robots that can utilize chemical fuels from their environment to generate energy and accomplish self-directed movement through chemical reactions.^996^ These robots make use of a variety of chemically reactive compounds, such as precious metals (*e.g.*, Au, Pt, and Ag),^29,997,998^ transition metals (*e.g.*, Fe,^367^ Zn,^999^ and Mg^1000^) (Figure 14a), reactive minerals (*e.g.*, CaCO_3_),^154^ alloys,^1001^ semiconductors,^107^ and bio-catalytic enzymes (*e.g.*, urease,^153^ catalase,^1002^ and glucose oxidase^1003^), ion-exchange polymers (Nafion,^307^ sulfonated polystyrene,^732^ sulfonated polystyrene-divinylbenzene block polymer^303^), among other materials. The initial artificial catalytic micro/nanorobots utilized a bimetallic design, primarily composed of Au and Pt sections.^26^ When exposed to H_2_O_2_ fuel, these micro/nanorobots produce self-electrophoretic gradients on their surfaces, facilitating movement. The creation of surface currents on the micro/nanorobots is a result of uneven reduction and oxidation electrochemical half-reactions taking place at each section.^1004^ In addition to micro/nanorobots based on bimetallic materials, there are also multi-component sections consisting of both inert and active materials.^1005^

**Figure 14:**
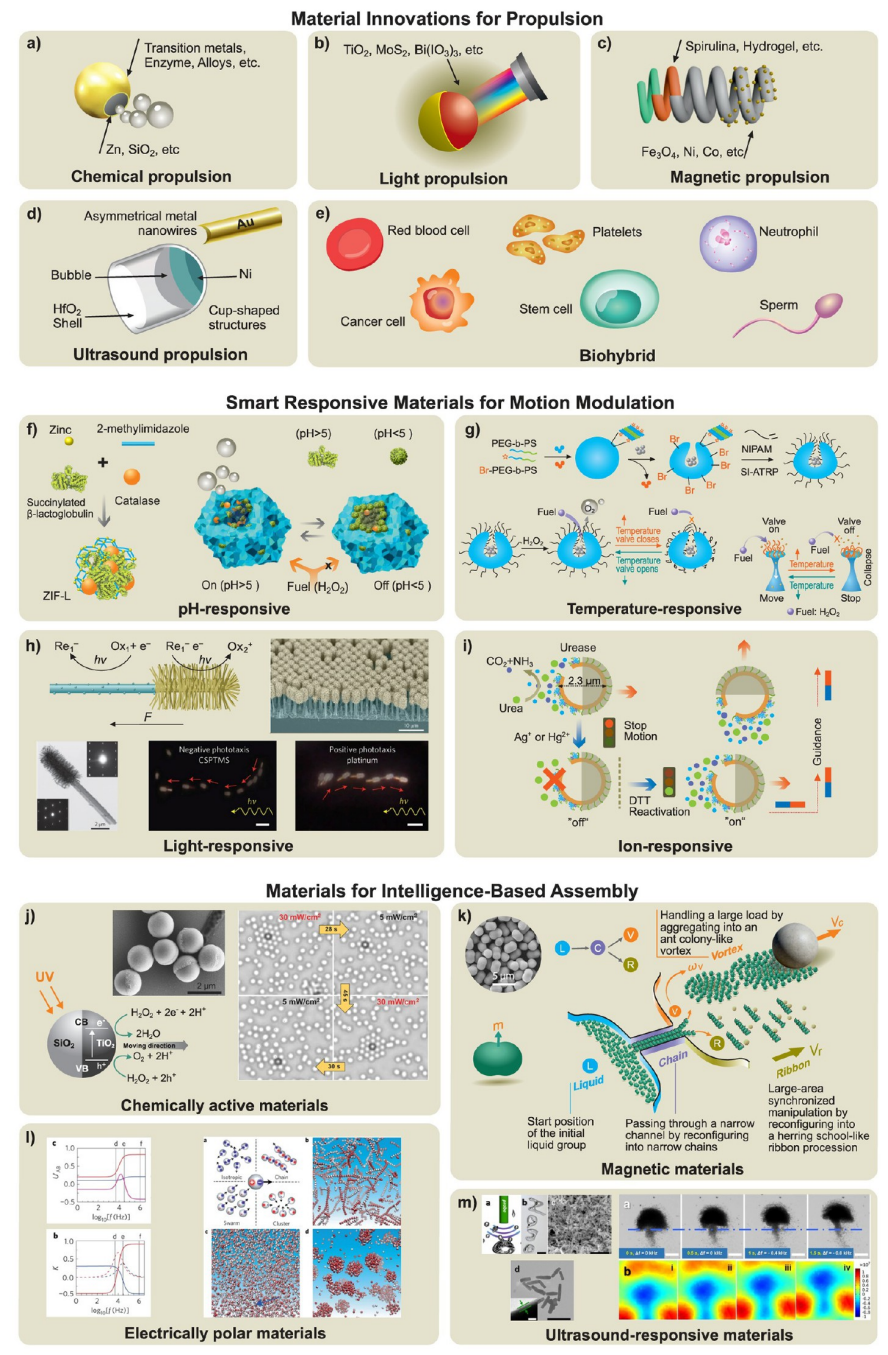
**Materials for micro/nanorobots and their dynamic assembly. a)** Typical materials for chemical propulsion. Reproduced from ref 264, Copyright 2021 WILEY-VCH. **b)** Typical materials for light-powered propulsion. Reproduced from ref 72, Copyright 2017 American Chemical Society. **c)** Typical materials for magnetic propulsion. Reproduced with permission under a Creative Commons CC-BY License from ref 1181, Copyright 2022 WILEY-VCH. **d)** Typical structures and materials for ultrasound propulsion. Reproduced with permission under a Creative Commons CC-BY License from ref 265, Copyright 2023 WILEY-VCH. **e)** Cells for the construction of biohybrid micro/nanorobots. Reproduced from ref 1182, Copyright 2018 Elsevier. **f)** Super-assembled biocatalytic porous framework micromotors with reversible and sensitive pH-speed regulation. Reproduced from ref 1183, Copyright 2019 WILEY-VCH. **g)** Self-propelled supramolecular nanomotors with temperature-responsive speed regulation. Reproduced from ref 1184, Copyright 2017 Springer Nature. **h)** Programmable artificial phototactic microswimmer. Reproduced from ref 107, Copyright 2016 Springer Nature. **i)** Urea-powered biocompatible hollow microcapsules. Reproduced from ref 1185, Copyright 2016 American Chemical Society. **j)** Nonequilibrium assembly of light-activated colloidal motors. Reproduced from ref 1186, Copyright 2017 WILEY-VCH. **k)** Reconfigurable magnetic microrobot swarms. Reproduced from ref 783, Copyright 2019 American Association for the Advancement of Science. **l)** Reconfiguring active particles by electrostatic imbalance. Reproduced from ref 517, Copyright 2016 Springer Nature. **m)** Reconfigurable assembly of active liquid metal colloidal cluster. Reproduced from ref 814, Copyright 2020 WILEY-VCH.

However, the propulsion mechanisms of such micro/nanorobots are influenced by environmental factors. For instance, the velocity of self-electrophoretic micro/nanorobots is influenced by the magnitude of the electric double layer that encompasses them, resulting in suboptimal performance in high-salinity environments and limiting their practical applications in real-world and medical settings.^1006^ Moreover, the breakdown of chemical fuels on surfaces characterized by nonuniform compositional distribution results in self-diffusiophoresis. This phenomenon involves the movement of molecules participating in catalytic reactions toward areas of lower concentration, thereby generating flow fields in response to solute gradients.^226^ These environmental factors impose constraints on the propulsion mechanisms and performance of micro/nanorobots.^1007^

An alternative method of localized propulsion for chemically driven micro/nanorobots involves the generation of gas microbubbles within their internal cavities and their continuous expulsion, akin to the launch propulsion strategy of a rocket.^185^ To regulate the locomotion of micro/nanorobots, they are coated with inert materials (*e.g.*, silica,^1008^ parylene,^1009^ polypyrrole,^155^ graphene,^1010^ *etc*.), creating a pathway for fuel entry and chemical reactions at a single opening. Typical micro/nanorobot designs feature hollow channels with catalytic functionality on the interior, inert materials on the exterior, and Janus microspheres with different levels of surface coverage.^1011^ However, chemically driven micro/nanorobots have certain limitations. Some commonly used fuels, such as hydrogen peroxide,^1012^ hydrazine,^1013^ and urea,^1014,1015^ may be toxic. Furthermore, alternative biocompatible micro/nanorobots constructed from materials such as Mg, Zn, and CaCO_3_ exhibit reduced longevity. In addition, the secondary products of chemical reactions have the potential to modify the pH levels in the immediate environment or elevate the presence of ionized substances. Micro/nanorobots made of ion-exchange polymers just fueled by innocuous salts are recently emerging but still need to be assessed for their potentialities in different complex environments.^307^ Therefore, these constraining factors must be taken into account when selecting appropriate fuels and materials.

##### 6.1.1.2 Material Design for Light-Powered Micro/Nanorobots

Light energy can be utilized as a power source for micro/nanorobots.^278,1016,1017,1018^ Micro/nanorobots can utilize a range of materials as suitable options for extracting energy from various segments of the electromagnetic spectrum and transforming it into kinetic energy.^1016^ One category of materials employs light to amplify or facilitate the chemical reactions necessary for the self-propulsion of micro/nanorobots. Hence, light can initiate various processes such as self-electrophoretic,^278,1019^ , self-diffusiophoretic,^8,817^ and bubble propulsion.^1020^ Light-sensitive materials can increase photochemical processes such as the splitting of water or the breakdown of substances like H_2_O_2_. Common materials used for light-induced water degradation or decomposition include silicon, bismuth iodate, metal oxides, and especially TiO_2_ (Figure 14b).^1021^ Another class of candidate materials converts light energy into heat by designing asymmetric absorption properties, enabling micro/nanorobots to induce NIR light absorption, leading to the creation of a temperature difference on the surface of the micro/nanorobots, causing them to move on their own through thermophoresis.^1022^ Recently, light-driven mechanisms based on interfacial tension gradients, such as the conversion between *trans* and *cis* isomers in photochromic materials and the deformation of liquid crystal elastomers through photomechanical processes, have also been suggested for micro/nanorobots.^504,1023^ Light-powered micro/nanorobots provide a high level of adjustability and the possibility of achieving autonomous movement in large-scale applications beyond the controlled environment, utilizing various wavelengths of the electromagnetic spectrum including ultraviolet, visible, and infrared light.^1024^ Nevertheless, light-powered micro/nanorobots encounter several obstacles, such as restricted light penetration, especially in scenarios involving biological samples and environments with high ionic strength.

##### 6.1.1.3 Material Design for Magnetically Powered Micro/Nanorobots

Externally powered systems provide numerous possibilities for the locomotion of micro/nanorobots.^1025^ Micro/nanorobots with magnetic materials can respond to external magnetic fields, allowing their controlled locomotion.^1026^ By incorporating magnetic materials into the design, the axes of micro/nanorobots can be reoriented with the magnetic field, enabling them to follow predetermined paths. Moreover, magnetic materials are commonly utilized in micro/nanorobots to convert applied magnetic fields into mechanical motion. For example, magnetic helical micro/nanorobots need to rotate around their central axis to move forward in fluids with low Reynolds numbers while the necessary torque is applied using oscillating magnetic fields. The resulting spiral movement converts magnetic forces into mechanical propulsion.^174^ The achievement of motion and control of micro/nanorobots depends on the integration of magnetic components such as Fe, Ni, or Fe_x_O_y_ into the main structure and the determination of their magnetization direction (Figure 14c). One category of magnetically driven micro/nanorobots utilizes flexible structures, where oscillating magnetic fields induce undulations in the flexible sections, propelling the entire micro/nanorobot structure.^1027^ In the same way, researchers have developed flexible magnetically driven micro/nanorobots by combining solid Au heads, flexible porous middle sections, and magnetic nickel tails.^408^ The development of such flexible hinge-like structures has given rise to various types of “swimming” behaviors.^416^ Magnetic materials incorporated into rigid helical micro/nanorobots can be propelled by externally oscillating magnetic fields, resulting in various propulsion mechanisms, with helical motion being the most notable.^51,174^ Magnetic microrobots can be built through the directed assembly of engineered particles, which can thus be reconfigured in a sequence-dependent manner.^1028^ These systems reconfigure when the external magnetic field is removed due to effective dipolar interactions, which can be useful for swimming in shear-thinning environments like mucus or sensing and metrology.^1029,1030^ The primary advantages of magnetically driven micro/nanorobots lie in their high precision control and sustainable motion. However, this approach also has limitations such as a limited operating range and the requirement for specialized equipment.^1031^

##### 6.1.1.4 Material Design for Ultrasound-Powered Micro/Nanorobots

The discovery of ultrasonic propulsion marks a new stage in the research of self-propelled synthetic micro/nanorobots. Ultrasonic propulsion holds promising characteristics, such as biocompatibility and versatility, which can bring tremendous benefits when used as a propulsion mechanism for micro/nanorobots in biomedical scenarios. In 2012, Mallouk’s group was the first to report ultrasound-driven nanorobots using Au-Ru bimetallic rods.^59,1032,1033^ To explore how ultrasound drives colloidal particles to perform directional motion,^471,472,1034^ they also reported synthetic ultrasound-driven microrods and built acoustic microrobots based on red blood cells loaded with DOX and iron oxide nanoparticles. Wang’s group fabricated Pt micro/nanotubes using an electrochemical deposition strategy by adjusting the pore size of ring-shaped porous polycarbonate templates.^1035,1036,1037^ Mallouk’s group sputtered a layer of Ni and a layer of Au on the surface of porous materials.^472^ Sitti *et al*. used the directional sputtering coating method to anisotropically deposit a layer of magnetic Ni nanofilm on the microrobot to achieve steering control of the ultrasonic microrobot under a uniform magnetic field, thereby achieving directed motion.^471^ Mallouk and co-workers fabricated HfO_2_ swimmers that could be ultrasonically driven by scraping a silicon wafer with a micropipette tip (Figure 14d).^1034^ Ultrasound-driven micro/nanorobots still face key challenges that need to be solved, such as the elucidation of the ultrasonic propulsion mechanism, precise control of movement speed, direction, and mode, and precise control of ultrasonic propulsion. Sakar’s group utilized two-photon polymerization to 3D-print ultrasound-driven soft robotic microsystems based on polyethylene glycol diacrylate, pentaerythritol triacrylate hydrogels, 4,4'-bis­(diethylamino) benzophenone photosensitizer, and Irgacure 369 photoinitiator.^1038^ Printing parameters were optimized to avoid strong adhesion to the substrate and seamlessly release the hydrogel microstructure, achieving precise control of ultrasonic propulsion.^59^ In a study by Wang’s group,^1039^ researchers used a membrane template-assisted electrodeposition method to develop an ultrasound-driven nanowire robot with nanoporous Au segments, and subsequently reported the design of a hemisphere through a ball template process. Both the shape and shell-shaped ultrasonic Au/Ti nanorobots have verified the control of speed and direction by ultrasound. The use of nanorobots also requires a large number of clinical experiments to verify the feasibility of ultrasound-driven micro/nanorobots in biomedical applications. In summary, ultrasound-driven nanorobots have become one of the most promising micro/nanorobots due to their biocompatible propulsion mechanism and good compatibility with current ultrasound diagnostic systems.

##### 6.1.1.5 Material Design for Biohybrid Micro/Nanorobots

Biohybrid micro/nanorobots made from biological cells (such as red blood cells^1040^ platelets,^36^ neutrophils,^1041^ cancer cells,^1042,1043^ stem cells,^1044^ and sperm cells^552,909,1045^ ) and synthetic materials can combine the driving functions of synthetic materials with the special functions of different biological materials, exhibiting excellent biocompatibility, high load capacity, long *in vivo* lifespan, and the ability to actively target disease sites (Figure 14e).

Red blood cells are the most abundant cells in blood circulation and considered one of the most promising manufacturing materials for biological hybrid micro/nanorobots. In contrast to other cells, they have a double concave shape, and, thus, a very large internal volume and expandable cell surface for large drug loading. A sufficiently long blood circulation time of red blood cells can significantly improve the pharmacokinetics of the loaded drug *in vivo*. Moreover, the isolation of red blood cells is simple and low-cost. Therefore, red blood cells have become a material for preparing micro/nanorobots with biocompatibility, long cycling, high capacity, and cost-effectiveness. Platelets are tiny anucleated cell fragments that can be recruited to sites of vascular injury and play multiple important roles in antimicrobial host defense, with specialized functions to hemostasis and prevent thrombosis.^1046^ Therefore, the development of platelet-based micro/nanorobots is an attractive direction in medicine. Inspired by platelets, micro/nanorobots were developed to effectively avoid immune rejection by covering platelets on the surface of the micro/nanorobots and to target specific tissues and cells.^1047^ Tang *et al*. developed Janus platelet microrobots by asymmetrically functionalizing the urease on the platelet surface.^36^ Neutrophils are the most abundant leukocytes in the blood and play a central role in innate immunity.^1048^ As another advantage, neutrophils are the fastest cells to reach the site of inflammation, with the ability to respond quickly and target it efficiently.^1049,1050^ Moreover, neutrophils have a short life span, only 8 hours in circulation, and low drug toxicity to normal tissues.^1051^ Thus, they can be a promising material for the preparation of micro/nanorobots to target drug delivery to inflammatory and tumor sites. Previous studies used neutrophil delivery of liposomes loaded with the anticancer drug paclitaxel for postoperative glioma treatment, delivery of the monoclonal antibody against the melanoma gp 75 antigen TA99 for melanoma treatment, and delivery of bovine serum albumin for the treatment of lung inflammation.^1052,1053,1054^ All these neutrophil-based micro/nanorobots showed promising targeting capabilities toward disease sites. Importantly, in addition to their homing capabilities, neutrophil-based micro/nanorobots are also able to cross the blood–brain barrier for the treatment of brain diseases. Therefore, neutrophil-based micro/nanorobots have been utilized to deliver liposomes loaded with various anticancer drugs for the treatment of brain diseases, demonstrating effective therapeutic outcomes.^1053,1055,1056^ Cancer cell-based micro/nanorobots, manufactured using cancer cell membranes to camouflage nanoparticles, have also been widely used in biomedical applications. Compared to other cells derived from blood, cancer cells have unique “homo-targeting” properties and thus show great potential for the treatment of isotype tumors.^1057,1058^ In addition, certain membrane proteins on the surface of cancer cells can inhibit their uptake by macrophages, helping to evade immune rejection and extend the retention time of micro/nanorobots in the bloodstream. This mechanism facilitates the efficient accumulation of drugs at the tumor site.^1059,1060^ In addition, due to the rapid value-added ability of tumor cells, it is convenient for large-scale *in vitro* culture to obtain abundant cell membrane material.^1061^ Multiple types of cancer cells, such as 4T1, breast cancer, and glioblastoma cells, are used as source cells for membrane acquisition. Due to their unique characteristics, micro/nanorobots based on cancer cell membranes have been widely used in the fields of drug delivery, photothermal therapy, photodynamic therapy, and immune regulation in tumor therapy. Stem cells can differentiate into various cells. According to their origin and plasticity, stem cells can be roughly divided into two types: embryonic stem cells and adult stem cells. Adult stem cells can be obtained from patients and expanded *in vitro* before injecting into the patients. Moreover, stem cells that can be induced to differentiate into specific cells under certain conditions are promising materials for targeted drug delivery.^1062^ Among the various stem cells, mesenchymal stem cells are the most attractive and widely used because they are nonhematopoietic multipotent cells and can be obtained from various types of human tissues, including bone marrow,^1062^ skin,^1063^ blood,^1064^ adipose tissue,^1065^ and placenta.^262^ In tumor therapy, mesenchymal stem cells possess the innate ability to migrate to inflammation sites, similar to leukocyte migration to inflammation sites, allowing for tumor-targeted delivery.^1066,1067,1068^ Mesenchymal stem cells have been used to deliver apoptosis inducers,^1069^ drug-loaded nanoparticles,^1070^ tumor tissue-specific precursor drugs,^1071^ and lysing viruses^1072^ for the treatment of inflammation and tumors owing to their low immunogenicity and homing ability to inflammation sites. Many studies have used mesenchymal stem cells to directly load paclitaxel nanoparticles for glioma-targeted therapy.^1073^

#### 6.1.2 Smart Responsive Materials for Micro/Nanorobots

##### 6.1.2.1 Smart Responsive Materials

Smart responsive materials refer to materials capable of making visible and tangible responses to changes in their surrounding environment or external stimuli (such as stress, electricity, magnetism, light, heat, pH, or chemical compounds) (Figures 14f–h). These materials possess unique adaptability, self-sensing, or memory capabilities.^1074,1075,1076,1077^ From classic smart responsive materials, such as shape memory alloys, piezoelectric materials, and photosensitive materials, robotics has rapidly evolved from rigid structures to soft robots composed of flexible materials.^1078,1079,1080^ Inspired by this, a new paradigm for the design of micro/nanomotors has emerged, where smart responsive materials are used as building blocks to develop intelligent, reconfigurable, and adaptive micro/nanomotors. The integration of smart responsive materials endows micro/nanomotors with the potential to grow, regenerate, and change shape in response to physical and chemical environments.^1081,1082,1083^ As a result, smart responsive materials have become the foundational materials for enabling intelligence in micro/nanomotors, supporting their potential applications in fields such as medicine, environmental remediation, and micromanufacturing.^14,103,824,1084^ Currently, smart responsive materials can be categorized into chemical stimuli-responsive materials, biological stimuli-responsive materials, and physical stimuli-responsive materials.

The range of chemical stimuli-responsive materials is relatively wide, including precious metals (Au, Ag),^29,997^ other metals (Fe, Zn, Hg),^367,1085,1086^ active minerals (CaCO_3_),^224^ alloys,^1087^ polymers, and semiconductors *etc*.^1088^ Chemical stimuli-responsive materials are often used to regulate the movement of micro/nanomotors. For example, pH-sensitive responsive materials can be employed to control the movement direction of micro/nanomotors, enabling them to target specific types of cells and deliver targeted drugs.^1089^ Additionally, a series of pH-sensitive photonic micro/nanorobots integrate real-time pH sensing and self-regulated drug release through structural color changes in response to pH variations.^1090^ Meanwhile, these robots enable precise photothermal therapy, utilizing thermochromic hydrogels for temperature mapping and selective tumor heating.^1091^ Moreover, they can actively detect, visualize, and treat tumors through motile-targeting mapping and stimulus-responsive drug release.^1092^ Micro/nanorobots based on MOFs can adjust their speed or start/stop motion through chemical inhibition and the addition of chelating agents.^1093,1094^ Moreover, chemical stimuli-responsive materials can sometimes impart unique properties to micro/nanomotors, such as displaying bright structural colors or enabling color change.^1095,1096^ In developing biocompatible micro/nanomotors, many studies directly use natural active substances (enzymes, sperm, algae, colon bacteria, *etc*.^201,205,1097^ ) as stimuli-responsive materials (Figure 14i).^1098,1099^ Proteins are particularly interesting as a structural building block for micro/nanorobots due to their versatility. Proteins are composed of amino acids arranged in a specific sequence that self-assembles into well-defined, responsive nanostructures. While enzymes have a chemical function and are used to catalyze chemical reactions for propulsion, structural proteins have a primarily mechanical function. For example, collagen forms helical fibrils and fibers that compose connective tissue such as tendons, cartilage, and ligaments, or silk, which is the main structural component of tough and strong spider webs and silkworm cocoons.^1100^ Because of their excellent mechanical properties, their intrinsic biocompatibility, versatility in microfabrication (including 3D printing and lithographic techniques), and responsiveness to biochemical environments, structural proteins present many advantages as a responsive structural component for micro/nanorobots. First, structural proteins are intrinsically biodegradable in a variety of biochemical environments through several proteolysis mechanisms. For example, enzymes can target specific amino acids to break down the main chain into smaller peptides. Matrix metalloproteinases can degrade collagen and gelatins as well as other extracellular matrix proteins. Gelatin (in its methacrylated version) has been used to 3D-print hydrogel structures and microrobots that are biodegradable in the presence of matrix metalloproteinases.^366,1101^ Alternatively, nonenzymatic degradation mechanisms include using pH or denaturing biomolecules to disrupt the folded protein nanostructures and disassemble the material. Semi-crystalline structural proteins like silk or squid suckering have been used to fabricate chemical microrobots and degraded with acidic stimulus (disrupting their β-sheet nanocrystalline structures and solubilizing the protein).^317,1102^ Therefore, protein microrobots can be rationally designed to degrade in the presence of specific biochemical cues for operation in biological environments.

Physical stimuli-responsive materials share a common characteristic: under external stimuli (such as light, temperature, magnetic fields, or electric fields), they typically change their shape, composition, or mechanical properties.^1103^ Thus, micro/nanorobots containing physically responsive materials can exhibit novel functions under the control of external physical fields. For instance, a class of flexible magnetic responsive materials can exhibit undulating locomotion in an oscillating magnetic field, propelling the entire microstructure and functioning as artificial flagella.^416,417^ The advantage of magnetic-responsive materials lies in their interaction with the magnetic field being reversible, instantaneous, and remotely controllable, laying the foundation for designing complex, multifunctional, and programmable micro/nanomotors. Additionally, as ultrasound can penetrate various biological tissues, micro/nanomotors combined with ultrasound-responsive materials show great potential for *in vivo* operation. When constructing such micro/nanomotors, ultrasound-responsive materials with high-density contrast relative to the surrounding fluid are mainly selected.^473,1104^ These materials are induced by ultrasound to oscillate, causing bubbles within the microstructure to vibrate.^486,1105^ When selecting electrical-responsive materials, basic criteria include conductivity, polarizability, deformability, fast response, and high efficiency. For example, metallic materials exhibit stronger polarizability compared to dielectric materials. The application of new electroactive materials can enhance energy efficiency and control precision.^75,77^ The use of electrical-responsive materials in micro/nanomotors holds great potential for developing artificial muscles and organs, microfluidic devices, and wearable electronics. Based on the working mechanism, light-responsive materials can be classified into photochemical, photothermal, photoisomerization, and photodimerization materials. Common light-responsive materials include diethylene, azobenzene, anthracene derivatives, salicylaldehyde-based compounds, spiropyran derivatives, phenylbutadiene derivatives, and other olefin-based substances.^1106,1107,1108,1109,1110^ Photoisomerization materials, such as E/Z isomerization, valence isomerization, cycloaddition, and tautomerization, are often used to design deformable soft polymer micro/nanomotors.^1111,1112,1113,1114,1115^ Under light irradiation, photothermal materials effectively absorb specific wavelengths or broad-spectrum light and convert it into thermal energy. These triggers preset mechanical works through phase changes in polymer networks or small molecules. Common light-absorbing materials include carbon nanostructures, plasmonic nanomaterials, and photochromic molecules.^1116^ Light-responsive materials offer high tunability and have the potential for large-scale control by harnessing different regions of the electromagnetic spectrum (*e.g.*, UV, visible light, and infrared). However, their light-penetration capabilities in biological samples and high ionic strength environments are limited. Thermo-responsive materials include, but are not limited to photothermal materials such as PNIPAM, PDMS, liquid crystalline elastomers. These materials not only deform when the temperature changes to propel micro/nanomotors but also give them new functions. For example, micro/nanomotors made from PNIPAM-based temperature-responsive materials can reversibly control the propulsion.^1117^

##### 6.1.2.2 Integrated Approach

Integrating smart responsive materials into micro/nanomotors represents a key research direction, focusing on enhancing manufacturing techniques and reducing production time. In the early days, when preparing jet micro/nanomotors based on strain-engineered rolled nanofilms, the nanofilms were first prepared using a photolithography process, then electron beam deposited catalytic materials, and finally the nanofilms were dried and rolled at an optimal supercritical point.^185^ As this technique is compatible with semiconductor on-chip technologies, rolled-up micromotors were recently integrated into fully fledged flexible microelectronic robotic systems for the first time.^1118^ Micro/nanomotors, on the other hand, can be quickly produced in the disciplines of microfluidics and self-assembly, with programmable performance and applications. Current methods for designing smart responsive materials into micro/nanomotors include electrochemical/chemical deposition, membrane template-assisted electrodeposition, asymmetric bipolar electrodeposition, physical vapor deposition, swept angle deposition, self-rolling method of spiral nanomaterials, and three-dimensional direct laser writing and layer-by-layer assembly, among others.^13,1119^ Another noteworthy manufacturing process is the inclusion of various materials into water-in-oil droplets, followed by emulsification, solidification, and direct assembly of asymmetric catalytic/magnetic nanostructures.^1120^ Examples include a three-step electrochemical potential technique for Co-Pt/Au micro/nanorobots, shape-controlled production of polymer-based microrobots utilizing polydimethylsiloxane templates, and template electroplating of graphene micro/nanorobots. Synthesis has been achieved using polymer micro/nanorobots doped with Pt nanoparticles/carbon nanotubes and evaporation-induced self-assembly of controlled crystals of ferrocene-based metal–organic materials.^1010,1121,1122^ Furthermore, coupling natural active substances as smart responsive materials with synthetic structures provides unique capabilities for micro/nanomotors. Numerous interaction-based techniques, including covalent bonds, electrostatic contacts, and physical clamping, can accomplish this.

##### 6.1.2.3 Design Principle

The practical application of smart responsive materials imposes strict requirements on their selection, design, and fabrication. Biocompatibility is a key factor when designing micro/nanomotors for biomedical applications. When choosing synthetic materials, careful consideration must be given to their safety, toxicological properties, and biocompatibility to minimize or eliminate any potential harmful side effects on the human body. Materials such as chitosan, mesoporous silica, and Mg are known for their biocompatibility. Additionally, due to the short lifespan of micro/nanomotors in corrosive environments (*e.g.*, stomach acid, blood, mucus), the high viscosity of biological fluids, and their susceptibility to contamination in complex biological environments, it is essential to select materials with protective coatings (such as fluoride, enzyme, or iron oxide coatings).^52,1123^ The ideal way to achieve biocompatibility is through the development of biohybrid microstructures, including cell-based micro/nanomotors composed of synthetic structures attached to living cells or synthetic micro/nanomotors coated with natural cell membranes.^1124,1125^ Novel smart materials are also expected to be degradable, allowing them to autonomously decompose into harmless ions or smaller polymer fragments within the body, thus avoiding potential toxicity issues.^126,1126^ Degradable micro/nanomotors made from transient metal catalysts (*e.g.*, Mg, Zn, or Fe) can be powered by reactions between the transient metals and water or acids, demonstrating effective movement and degradation in biological environments. Layer-by-layer assembly of functional proteins on micro/nanomotors is another attractive approach as it not only enhances the biocompatibility of the microstructures but also serves as a biodegradable support structure. Additionally, to develop microrobots capable of traveling through narrow channels without causing blockages or damage, materials with a Young’s modulus similar to that of cells are required. These should be soft, deformable, or reconfigurable materials, such as hydrogels and rubber elastomers.^1127^

#### 6.1.3 Shape of Micro/Nanorobots

Researchers have built and designed self-propelled micro/nanorobots with a variety of propulsion methods using a variety of materials. The design of micro/nanorobots encompasses both structural and component aspects. This design process directly influences the synthesis method, size, morphology, power source, and practical applications of micro/nanorobots, highlighting the inseparable connection between micro/nanorobot design and material selection.^1128^ Metals, metal oxides, and enzymes are three types of active materials commonly utilized in self-propelled micro/nanorobot design. For magnetically driven micro/nanorobots, magnetic materials such as Fe_3_O_4_, γ-Fe_2_O_3_, and FePt should be prioritized. To achieve asymmetric chemical propulsion for microrobots in fuel-containing environments, catalytic materials including Zn, Mg, Pt, MnO_2_, and enzymes might be needed. In micro/nanorobots that do not release bubbles, anisotropic geometries or compositions, commonly known as Janus particles, are frequently utilized. Currently, the majority of transition metal components are used heavily in the design and construction of micro/nanorobots.^123^ These materials are generally available for the design of micro/nanorobots in different sizes. The typical dimensions of micro/nanorobots can range from several nanometers to several micrometers. The structures of micro/nanorobots can be categorized as 1D, 2D, or 3D based on their morphological characteristics.^1129^

Among the types of micro/nanorobots, 1D structures are the most distinctive.^1130,1131^ 1D micro/nanorobots are structurally anisotropic and exhibit a large specific surface area, providing a good site for functional modifications, and the anisotropy of the 1D structure permits the design of 1D micro/nanorobots for different modes of motion (*e.g.*, axial and rotational motion).^1132^

In 2D micro/nanorobots, one dimension is generally confined to the nanoscale while the other two dimensions extend beyond the nanometer range. Owing to their enormous surface area, conjugation effects, and distinctive biochemical characteristics obtained from diverse substances, two-dimensional nanomaterials have significant potential for biological applications and perform very well in drug transport and catalysis.^1133^ The substantial specific surface area of two-dimensional nanomaterials facilitates the presence of numerous catalytic sites and anchoring points, which results in elevated levels of catalytic activity and loading capacity. Layered double hydroxides represent a prominent category of 2D nanomaterials that exhibit a range of advantageous properties, including multifunctionality and enhanced biocompatibility. Zhang *et al*.^1134^ developed nanomotors based on 2D nanosheets with chemotaxis toward the tumor microenvironment. The 2D structure of the nanomotors exhibit significant catalytic activity, allowing it to effectively engage in rapid, sustained, and relatively long-range chemotaxis in response to high-gradient H_2_O_2_ fuel. This capability underscores the potential of the nanomotors to function as an intelligent carrier, thereby maximizing its operational efficacy.

3D morphology improves mechanical stability, facilitates the design of artificial shapes, and promotes adhesion in comparison to 1D/2D nanomaterials, providing a unique bionic structure that could open new avenues for biomedical applications.^1129^ Natural structures with unique morphological features, such as pollen or microalgae, have been used as templates for micro/nanorobots.^1135^ Nanomaterials or catalytic metals responsible for propulsion can be easily combined with 3D templates. In addition, the effect of morphology on micro/nanorobots enables more precise manipulation of motion.^1136,1137^ It has been demonstrated that specific shapes as well as surface morphologies and properties are beneficial for further promoting chemotactic control. For instance, micro/nanorobots designed based on animal morphology exhibit increased vibrancy and engagement. Given that synthetic micro/nanorobots typically operate within diverse liquid environments, there has been a surge in studies inspired by fish morphology. The anatomical features of these fish-inspired structures comprise a body, a tail embedded with Pt, and a head containing Fe_3_O_4_. The microfish exhibit efficient autonomous movement facilitated by catalytic Pt nanoparticles while their ability to navigate in a predetermined direction is attributed to the presence of Fe_3_O_4_ nanoparticles. In light of this, polydiacetylene nanoparticles may be added to the microfish to enhance their ability to neutralize toxins.^1138^

The advancement of nanotechnology has enhanced the versatility of micro/nanorobots. However, several critical challenges remain to be addressed, including inadequate biocompatibility, simplistic morphological characteristics, limited preparation methodologies, and restricted motion behaviors. Nevertheless, emerging evidence suggests that the biological behavior and toxicity of micro/nanorobots can be influenced by the regulation of their structural, size, biohybrid, and functional groups.

#### 6.1.4 Size of Micro/Nanorobots

In the field of micro/nanorobots, size plays a pivotal role. These micro/nanorobots exhibit a wide range of sizes, spanning from a few nanometers to hundreds of micrometers, and are primarily synthesized using top-down and bottom-up strategies or a combination thereof. Due to their wide size distribution, micro/nanorobots with different sizes ranges have different requirements in terms of synthesis strategies, propulsion mechanisms, and applicability. There are no special requirements in terms of materials—it mainly depends on the above three aspects. Large-sized microrobots are generally constructed using top-down technologies such as lithography technology, vapor deposition technology, contact printing technology, *etc*.^13,564,705,965^ Effective displacement is achieved through nonreciprocal behaviors, such as deformation, tumbling, rotation, and bubble release.^46,504,966^ In terms of application, due to the size scale, it can have applications in the treatment of organ diseases in external environments, such as stomach, intestinal, and bladder.^1139,1140,1141^ However, to meet the requirements for use in organs within the internal environments of the body, size reduction is inevitable. Bottom-up strategies such as chemical synthesis or self-assembly techniques (such as solution self-assembly, soft template self-assembly, layer-by-layer self-assembly, and supramolecular self-assembly) have demonstrated suitability for fabricating smaller micro/nanorobots or building blocks for micro/nanorobots.^1142^ In general, top-down strategies often need to be integrated into the synthesis of micro/nanorobots to achieve their self-propulsion ability. Small-sized micro/nanorobots can utilize similar motion modes as large-sized microrobots, enabling effective displacement. In addition, phoretic effects such as self-diffusiophoresis, self-thermophoresis, and self-electrophoresis are preferred for small-sized micro/nanorobots. For instance, H_2_O_2_-driven Pt-SiO_2_ microrobots predominantly rely on bubble propulsion when their size exceeds 10 μm, whereas the phoretic effect becomes the primary driving mechanism when their size falls below 5 μm.^1143^ The size dependence of micro/nanorobots’ motility is an interesting research topic. Several studies have explored the correlation between size and motility in micro/nanorobots with identical structures. Notably, it has been observed that Janus microrobots employing a self-diffusiophoretic mechanism exhibit an increase in velocity as their size decreases.^602^ Conversely, bubble-driven tubular microrobots experience a decrease in velocity with decreasing size.^1144^ Additionally, thermophoresis-propelled microrobots may display a reduced motility tendency as their size diminishes.^1145^ These findings highlight distinct trends in the relationship between micro/nanorobot motility and size across various propulsion mechanisms and structures. It should be noted that these experimental results are limited to a few specific sizes. If the size decreases further, then the micro/nanorobot faces increased viscous resistance and stronger Brownian motion, potentially resulting in enhanced Brownian motion of micro/nanomotors instead of directional movement. The size of micro/nanorobots must be carefully selected to suit different application scenarios, especially in the biomedical field. Unique requirements for micro/nanorobot size exist in various biological barriers;^1146^ hence, the appropriate size and synthesis strategies should be selected based on application requirements and environmental conditions.^263,1147,1148^

### 6.2 Materials and Nanoarchitectonics for Intelligence-Based Dynamic Assembly of Micro/Nanorobots

Collective behavior is a prevalent phenomenon in nature, where many individuals gather together through communication to form various types of swarms.^1149,1150,1151^ The swarm intelligence and emergent behavior in nature inspire the design of micro/nanorobot systems that can complete tasks that are difficult to achieve with individual micro/nanorobots.^1152,1153^ Chemical fields, electric fields, magnetic fields, ultrasound, and light have all been effectively employed to assemble and aggregate micro/nanorobots until now.^25,119,1154^ The physical and chemical characteristics of micro/nanorobots, as well as their interactions with various energy sources, are critical in building programmable and intelligent swarms at small scales.

#### 6.2.1 Requirement for Materials

Highly sensitive catalysts are promising candidates for equipping micro/nanomotors with communication capabilities to form intelligent swarms. These catalysts efficiently and rapidly catalyze the decomposition of fuels or target species, enhancing chemical gradients in their vicinity and facilitating assembly.^565,1155^ Materials that are easily polarized or charged can use electrostatic interactions to induce assembly. Van der Waals forces and short-range hydrophobic interactions between bigger biomolecules or other organic materials have evolved as the primary means of communication between neighboring micro/nanomotors. In addition, external physical fields can add interactions between materials to enrich the types of micro/nanomotor swarms, depending on the properties, intensity, and frequency of the fields. In summary, the range of materials used for building blocks gradually expands from rigid to soft materials, from homogeneous to hybrid materials, and from special to general materials.^1156,1157^

##### 6.2.1.1 Biochemically Active Materials

The collective behavior induced by chemically active materials is based on surface reactions.^1158^ These materials are generally catalysts, including metal materials (Pt, Mg, Zn, Ag), organic materials (acid catalysts, alkali catalysts, metalloid organic catalysts), inorganic materials (TiO_2_, Ag_3_PO_4_, ZnO, Fe_2_O_3_), biologically active materials (enzymes, cytochromes, nucleic acids), ion-exchange polymers, *etc*. The essence of these chemically active materials is to create an unbalanced chemical field through chemical reactions and convert chemical energy into the motion of micro/nanomotors and the flow of solvent around them, providing power for the assembly and aggregation of micro/nanomotors. In addition, biochemically active materials avoid the generation of bubbles during chemical reactions in high-density motor swarms, eliminating the influence of spurious effects caused by bubbles.^153,232,563^ Micro/nanomotors embedded with chemically active materials often exhibit versatile self-assembly and collective behaviors and are organized into dynamically stable aggregates of limited size (Figure 14j). Their self-organization is dominated by nonreciprocal interactions caused by chemical fields, although DLVO forces, magnetic or electrostatic dipole interactions, depletion interactions, and capillary interactions also play a role. Each motor’s nonreciprocal interactions include its reaction to chemical fields created by other motors as well as chemical fields coupling between motors. These are long-range forces that typically exhibit an r^–2^ decay with distance *r* from the motor’s center of mass, which are very helpful in producing structurally stable assemblies and swarms.^743,750^ Thus, inert particles surrounding chemically active micro/nanomotors may be both attracted and temporarily decomposed or reconfigured by switching the nature of nonequilibrium effective interactions from attraction to repulsion. It is also possible to consider adding chemically active materials with pulsating activity to the assembled structures to cause the effective interactions to change repeatedly.^789,1159^ For example, when submerged in an aqueous solution containing KCl and H_2_O_2_ and treated with UV radiation, active Ag/PMMA micro/nanomotors could generate a self-assembled structure of periodic oscillation (“beating” structure).

As advancements in micro/nanomotor research and materials manufacturing technologies continue, increasingly complex chemically active materials are being utilized. For instance, under UV light, a Fe_2_O_3_-polymer bead complex generates anisotropic diffusiophoresis, facilitating the assembly of seven micro/nanomotors into a rotating gear structure.^1160,1161^ This might be considered one of the first examples of hierarchical dissipative dynamic assembly. Similarly, microspheres demonstrated reversible assembly after being functionalized with the proteins PhyB and PIF6. When collectives are built from multiple building blocks, motor swarms emerge in a hierarchical order, exhibiting biomimetic behaviors and similar functions such as leader–follower behavior, predator–prey chasing, collective migration, and phase transitions or reconfigurations.^798,815^

##### 6.2.1.2 Magnetic Materials

Magnetic materials can assemble into dipole chains or rings through self-generated magnetic dipole–dipole interactions (Figure 14k).^1162,1163^ Furthermore, external magnetic fields can amplify these interactions. External magnetic fields, with their exact control, can be utilized to adjust the balance between field-induced interactions and other interactions. Therefore, external magnetic fields are complementary to other self-assembly methods and can be used in combination to achieve a variety of interesting assemblies or collective phenomena. Magnetic materials usually refer to ferromagnetic (*e.g.*, Fe, Co, Ni) and ferrimagnetic materials (*e.g.*, Fe_3_O_4_, CoFe_2_O_4_, and NiFe_2_O_4_). When the size of a ferromagnetic or ferrimagnetic material is reduced to the nanoscale, the material can become superparamagnetic, *i.e.*, it has no remnant magnetization but is highly responsive to external magnetic fields. The dynamic behavior of magnetic materials is influenced by the input field (such as field strength and alternating frequency) as well as the environment (such as viscosity and ionic strength). The magnetization curve of ferromagnetic materials (*e.g.*, Fe, Co, and Ni) demonstrates that they will remain magnetized after being magnetized by an external magnetic field. When a weak magnetic field is supplied, the magnetic moment aligns with the external field. When the external magnetic field is removed, the magnetism of ferromagnetic materials remains, whereas the magnetism of superparamagnetic materials dissipates. Compared to ferromagnetic materials, superparamagnetic materials prevent aggregation caused by residual magnetism and are commonly employed in targeted delivery applications.

Superparamagnetic Fe_3_O_4_ nanoparticles are commonly used materials to construct magnetic assemblies or swarms. At low Reynolds numbers, moving chains of magnetic particles are influenced by the balance of magnetic driving force and viscous resistance. Therefore, the magnetic particle population can exhibit a reversible diffusion–aggregation process.^779^ Superparamagnetic polymer particles are typically generated by a homogeneous dispersion of iron oxide nanoparticles inside a polymer matrix (like polystyrene). They have a relatively insensitive magnetic response and greater particle dispersion than Fe_3_O_4_ nanoparticles, which prevents aggregation and allows us to monitor the activity of individual particles in swarm systems. Under the influence of a magnetic field, superparamagnetic polymer particles aggregate and form swarms by a combination of dipolar attraction (particle–particle interactions inside the chain) and multipolar repulsion (chain–chain interactions). Anisotropic magnetic particles exhibit unique characteristics such as building blocks for assemblies and swarms because of their anisotropic nature and responsiveness to external stimuli. In general, anisotropic magnetic particles can be divided into two categories: shape anisotropy and magneto-crystalline anisotropy due to their shape or crystal structure. In contrast to spherical particles, peanut-shaped Fe_2_O_3_ colloidal particles contain a persistent moment along their short axis and show frequency dependence on an external magnetic field.^783^ Under different magnetic input fields, they exhibit different collective states, including liquid, chain, vortex, and belt states. It is important to note that if microrobots are designed for biomedical applications, then their biocompatibility must be also considered.^1164^ Among various magnetic materials, Co and Ni are generally considered toxic because the human body has a low tolerance for these elements. Fe is usually considered as a biocompatible alternative. Currently, the only FDA-approved magnetic nanomaterials for clinical use are magnetite and maghemite, highlighting the need of developing biocompatible magnetic materials with high magnetic response.

##### 6.2.1.3 Electrically Polar Materials

The qualities of polar materials and carrier media have an impact on the assembly of micro/nanomotors (Figure 14l). Carrier media comprise nonconductive liquids, mildly conductive liquids, and conductive electrolytes. In comparison to the carrier medium, the polarization of polar materials, as well as the distribution of mobile charges (such as scattered ions) and fixed charges (such as covalently bonded surface charges), are significant in the induction of micro/nanomotor assembly. The surface of polar materials distributed in an electrolyte frequently bears an intrinsic charge, causing short-range electrostatic repulsion or attraction between micro/nanomotors.^1165^ As a result, even when no external field is present, electrostatic interactions can cause self-assembly. This self-assembled structure is influenced by the screening of electrostatic contacts and pH-induced changes in charge distribution.^1166,1167^ However, with the help of an external electric field, larger-scale assembly structures and motor groups can be formed. An external electric field induces the polarization of polar materials; then, the micro/nanomotors arrange according to dipole interaction and finally form a dipole chain.^798^ Charged micro/nanomotors are efficiently driven under an electric field and their characteristic parameter is electrophoretic mobility. Through electrophoresis, even uncharged micro/nanomotors with effective polarization can be driven and assembled under the action of nonuniform electric fields. To date, examples of electric field-driven self-assembly, particularly those involving temporary structures, have primarily focused on inducing polarization in polar materials. This polarization can be achieved using either DC or AC electric fields. DC electric fields create high currents in the electrolyte, which can damage the material and disrupt the dispersion of free carriers. Research using DC electric fields has focused on poorly conductive solutions or systems with weak current intensity requirements. To overcome this constraint, high-frequency AC electric fields are commonly utilized to polarize solid dielectric particles. Simultaneously, low- to medium-frequency AC electric fields may be applied to most particle suspensions. To produce active structures using an external field, a dielectric difference between the polar material and the carrier fluid is also necessary. Any substance becomes polarized in an electric field and tends to create dipole chains. As the applied electric field intensity increases, so do the generated dipolar interactions, allowing some systems to have many reversibly stable phases. If the mobility of building blocks is high enough, then reversible switching between configurations is possible.^801^ Compared with chemically active materials, the assembly induced by polar materials does not require any other features or forms and the response time is quite fast, only a few seconds. The assembly produced by an external electromagnetic field is reversible and the electric field intensity and frequency may be continually tuned to many orders of magnitude accuracy to fit the properties of varied systems. In addition to metallic coatings that act as ideally polarizable materials, semiconducting layers can be deposited. These layers are responsive to light and can continuously change their electronic conductivity with varying optical light intensity, thus dynamically bridging the gap between dielectric and conducting properties in a controllable manner.^1168^ However, if not well controlled, electric fields can harm cells. An interesting method of controlling electrical fields is to use acoustic waves or magnetic fields to activate electrically polarizable materials, generating electric fields locally. These localized electric fields can influence cell behavior in various ways, such as inducing differentiation and enhancing proliferation.^366,1169,1170,1171^ Additionally, the fields can be used to control drug release, minimizing drug leakage during targeted drug delivery.^1172,1173^ This localized field generation with electrically polarizable materials adds extra functionality to microrobots.^1174^

##### 6.2.1.4 Other Materials

For micro/nanomotors composed of materials without chemical activity and electromagnetic properties (such as certain polymer materials) to self-organize into assemblies and swarms, new assembly methods need to be explored. Among many operating techniques, ultrasonic manipulation, in addition to being compatible with a wide range of materials, is also the fastest and most biocompatible method to trigger the self-organization of micro/nanomotors. By adjusting the amplitude and frequency of sound waves, tiny liquid metal nanorods can be wirelessly controlled and form a swarm of liquid metal micro/nanomotors.^1175^ During the assembly–dispersion process, the sound pressure distribution changes with frequency, *i.e.*, the acoustic radiation force induced by standing waves varies with input parameters. The liquid metal nanorods experience stresses along the acoustic wave energy gradient, resulting in various motion states. In addition to managing the swarm mode, collective navigation may be accomplished by altering the input sound source. For example, in a study, Pt-Au nanowires were placed in an H_2_O_2_ solution. In the absence of an acoustic field, nanowires exhibit electrophoresis-dominated self-propelled motion. The standing wave gradient generated by the sound wave drives the nanowires to aggregate and move toward the low-pressure region, thereby forming a high-density nanowire swarm. Swarm movements may be accomplished by varying the frequency of sound waves, which shifts the position of pressure nodes. The size and position of the swarm vary depending on the characteristics of the ultrasonic field, the size and height of the chamber, and the density of micro/nanomotors. The interaction of particles with the ultrasonic field provides the basis for ultrasound-induced collective behavior (Figure 14m). No unique materials, shapes, or sizes are necessary. The response time is merely a few seconds and it is safe to use with practically any solution or material. Furthermore, optical tweezers can operate to construct micro/nanomotors using photophysical fields. Optical tweezers are devices that employ highly concentrated beams to accurately grasp and manipulate micro/nanoscale objects.^1176,1177,1178^ The intensity gradient created by the convergent beam polarizes materials at micro/nanoscale, causing them to gravitate toward the light field’s maximum gradient. Using highly regulated light pressures, many structured colloidal assembly patterns were exhibited.^1179,1180^ Because light does not necessitate complicated device fabrication methods, it is the most straightforward way to generate collective or assembly activity.

#### 6.2.2 Reconfigurability

The reconfigurability of collective behaviors paves the way for advancing the self-adaptability and intelligence of micro/nanorobots. The occurrence of reconfigurability requires a mechanism to break and reconstruct the interaction between micro/nanorobots. Based on whether the response of micro/nanorobots to the breaking mechanism exhibits spontaneous properties, assemblies are categorized as either spontaneously reconfigurable or reconfigurable by an external field.^1187^ This spontaneity is usually determined by the design and control strategy of micro/nanorobots as well as the material properties of micro/nanorobots (catalytic properties, magnetic, dielectric properties, various surface properties, *etc*.). Spontaneously reconfigurable assembly generally exists for chemical-driven micro/nanorobots, which mainly realizes the reconstruction behavior by changing the chemical field around the micro/nanorobots. For example, the swarm system consists of at least two or more chemical-driven micro/nanorobots with different catalytic properties. Through different chemical reactions, chemical fields that affect each other are generated, which leads to reconstruction. For example, the swarm system composed of different materials ZnO and TiO_2_, respectively, shows the reconstruction behavior of predator and prey clusters,^841^ whereas the swarm system composed of ZnO and sulfonated polystyrene microspheres intelligently selects the most suitable configuration in a confined environment of different shapes.^303^ Additionally, other chemical substances can be introduced, or the chemical reaction can be altered by adjusting light intensity, to achieve reconstruction behavior.^117,1159,1188^ The external field-controlled reconfigurable assembly depends on the applied external field and the configurations are changed by adjusting some parameters of these external fields.^814,1189,1190^ External field-controlled reconfigurable assembly has relatively low requirements on materials and only needs basic material properties that can be driven, such as magnetic and electric field control. The external field-controlled reconfigurable assembly has high controllability and can be transformed between multiple configuration modes according to artificial preferences to achieve the corresponding purpose. Recent demonstrations show that spatially patterning electric fields via optical patterning of a photoconductive substrate induces optoelectronic-driven forces on both passive and active particles, enabling trajectory control,^1191,1192^ and directed self-assembly.^1192^ This has great application potential in the biomedical field. In addition to the basic material properties, the shape of the micro/nanorobots is also an important parameter. Different shapes of micro/nanorobots have different motion modes under the magnetic field, which affects the part of the flow field and leads to differences in collective behavior. In addition, specific surface interactions, such as electrostatic interactions and van der Waals forces, are material factors that affect reconfigurability; hence, collective behavior is modulated by adjusting these parameters.

### 6.3 Design and Fabrication Methods

#### 6.3.1 Bottom-Up Fabrication

Bottom-up controlled self-assembly technology is a promising candidate for versatile and controllable fabrication of multifunctional smart micro/nanorobots.^1193^ It is a kind of spontaneous organization relying on noncovalent interactions of elemental units, such as molecules, nanomaterials, or larger materials. Micro/nanorobots fabricated by self-assembly are equipped with spatially asymmetric structures and engines for creating an imbalance of diffusional force around themselves, inducing propulsion.^1194^

##### 6.3.1.1 LbL Self-Assembly

Some of the earliest documented self-assembled micro/nanorobots include Pt-loaded stomatocyte-shaped nanorobots developed by Wilson’s group using a solution self-assembly technique, and Janus polyelectrolyte capsule micromotors created by Wu *et al*. in 2012 using a layer-by-layer (LbL) self-assembly approach.^1195^ This marked a new era of self-assembled micro/nanorobots. Typically, various polyelectrolyte multilayer films (*e.g.*, PSS/PAH,^1195,1196,1197,1198,1199,1200,1201,1202,1203^ PAA/PAH,^1204^ PSS/PEI,^1205^ PEI/CAT,^1206^ PDDA/PSS,^1207^ and CHI/ALG^1012,1208,1209^ ) can be readily LbL-assembled on different sacrificial templates (such as spherical colloidal particles (usually SiO_2_, CaCO_3_), porous polycarbonate membranes), and combining microcontact printing and metal sputtering deposition methods to form Janus microrobots, which cover a wide range of diverse forms (Figure 15a).^1210^ Among them, the polyelectrolyte-based capsule microrobots not only possess controllable autonomous motion by chemical reaction or NIR irradiation but also outstanding delivery capabilities and stimulus release of cargo, overcoming the shortcomings of previous micro/nanorobots that require surface modification in advance when loading objects. It is worth noting that the polyelectrolyte-based tubular micro/nanorobots can display high speed and controllable directional movement due to the asymmetry of their morphological structure,^1012,1196,1207,1211^ and even can achieve subcellular photomechanical perforation of the membrane.^1202^ Controllable preparation of polymer multilayer tubular microrobots can also be achieved by combining LbL assembly with the rolled-up nanomembrane technique.^1209^ In addition, the practical application of synthetic micro/nanorobots in the biomedical field requires both their degradation into nontoxic compounds and high biocompatibility.^1212^ Hence, substrates are not limited to polyelectrolytes but also can be heparin,^1213^ protein,^1214^ enzymes,^1206^ and molecular motor^1215^ to develop micro/nanorobots with better biocompatibility in a controlled manner. Biodegradable tubular microrobots constructed using biocompatible bovine serum albumin and poly­(L-lysine) as LbL-assembled materials not only can deliver DOX to targeted cancer cells, but can also achieve controlled release of DOX under NIR irradiation. Importantly, these microrobots can be biodegraded by trypsin treatment.^1214^

**Figure 15:**
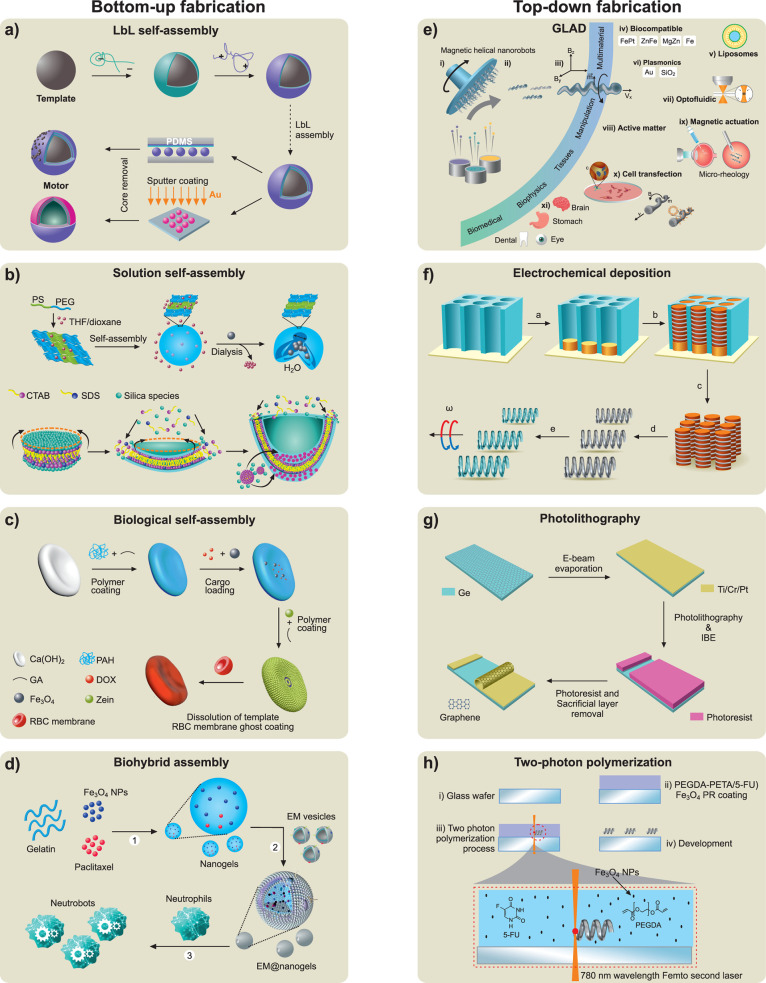
**Fabrication of micro/nanorobots. a)** Synthetic procedures of microrobots by combining the LbL self-assembly technique with the metal sputter-coating method and PDMS; reproduced from ref 1205, Copyright 2022 WILEY-VCH; reproduced with permission under a Creative Commons CC-BY License from ref 1213, Copyright 2018 American Chemical Society. **b)** Stomatocyte-like nanorobots formed by the solution self-assembly of block copolymers PEG–PS in THF/dioxane solvent; reproduced from ref 1216, Copyright 2012 Nature Portfolio. One-pot solution self-assemble approach for large-scale synthesis of shuttlecock-shaped nanomotors; reproduced from ref 1225, Copyright 2020 Elsevier. **c)** membrane-camouflaged microrobots formed by biological self-assembly and **d)** Cell-based microrobots. Reproduced from ref 1041, 1235 , Copyright 2022 American Chemical Society; Copyright 2021 American Association for the Advancement of Science. **e)** Glancing angle deposition.^1251^ Adapted with permission from ref 51 Copyright 2009 American Chemical Society. Adapted with permission from ref 1251, Copyright 2021 Royal Society of Chemistry. **f)** Template-based fabrication of helical magnetic nanorobots by electrochemical deposition. Reproduced from ref 1326, Copyright 2014 Royal Society of Chemistry. **g)** Fabrication process of rolled-up tubular micromotors utilizing lithography technology. Reproduced from ref 1334, Copyright 2019 WILEY-VCH. **h)** Fabrication of degradable hyperthermia microrobot utilizing 3D printing technology. Reproduced from ref 1323, Copyright 2019 WILEY-VCH.

##### 6.3.1.2 Solution Self-Assembly

The assembly technique mentioned above makes micro/nanorobots well-positioned for the rational design and construction of multifunctional micro/nanorobots as a gentle and straightforward method. However, these processes usually require sacrificial templates and a time-consuming and labor-intensive workload. In contrast, an approach based on solution self-assembly can address these issues, offering alternative solutions for the large-scale production of micro/nanorobots. Among them, the most prominent work is the self-assembly of amphiphilic block copolymer into stomatocyte-like nanorobots (Figure 15b).^1216,1217,1218,1219,1220,1221,1222^ In addition, assembling of the temperature-sensitive PNIPAM polymer brushes *via* surface-initiated atom transfer radical polymerization, or addition of host–guest self-assembly complexes on the surface of silica-based nanorobots^230^ or stomatocyte-like nanorobots could, respectively, lead to temperature-responsive^1184^ and light-responsive speed regulation.^1223^ To fabricate more biocompatible stomatocyte-like nanorobots, degradable PEG-PDLLA block copolymers are used to replace nondegradable PEG-PS.^236,1224^ Furthermore, integrating an aggregation-induced emission (AIE) fluorophore into the block copolymer seems to be a promising way for biomedical imaging.^1014^ In addition to various amphiphilic block copolymer-based self-assembly approaches, large-scale self-assembly of inorganic micro/nanorobots has also been demonstrated. Examples include shuttle-shaped silica nanorobots formed through a simple one-step self-assembly process using two surfactants (cetyltrimethylammonium bromide and sodium dodecyl sulfate) (Figure 15b), MOF-based microrobots created by efficiently assembling MOF nanoparticles *via* a transient Pickering emulsion method,^1226^ and self-assembled Ag-PCL microrobots relying on noncovalent interactions of multifunctional materials.^1227^ To adapt to various applying environments, functional salt-responsive PNIPAM brushes are selectively assembled onto micro/nanorobots to reversibly regulate the speed of robots in fuel owing to the changeable wettability of the brushes by ion-exchange.^1228^

##### 6.3.1.3 Biological Self-Assembly

Common self-assembly technology is usually incapable of withstanding considerable immune attacks and bio-adhesion, which restricts their usage in many applications such as cell surgery, where biological self-assembled micro/nanorobots would enable advances.^1229^ The first bio-functionalized interfacial polymeric nanoparticles, self-assembled by Zhang’s group through membrane camouflage strategy, achieved extended retention periods in blood circulation and enhanced enrichment ability in the tumor site.^1230^ Importantly, the assembly of biological units endows micro/nanorobots not only the inherent biological functions of natural cells, protein, and/or bacteria, *i.e.*, escaping immune phagocytosis, long-term circulation *in vivo*, target recognition, and crossing biological barriers, but also diverse locomotion modes under the action of smart artificial parts.^1231^ As a result, various examples of natural cells, such as red blood cells with unique immunologic function and oxygen-carrying capability,^453^ neutrophils with the ability of chemotaxis toward pathogens and inflammatory sites (Figure 15d),^1041,1232^ and platelet cells-immobilized urease asymmetrically *via* biotin-streptavidin-biotin binding complex,^36^ have been employed to obtain hybrid cell-based micro/nanorobots. In addition, assembling membranes derived from red blood cells (Figure 15c),^1032,1233,1234,1235^ *Escherichia coli*,^1041,1232^ platelets,^1236^ macrophage cells,^1237^ cancer cells,^1238,1239^ leukocytes,^1125^ lipid,^209,1240^ and photosynthetic bacterial membranes^1215^ on the surface of particles *via* electrostatic attraction to fabricate membrane-camouflaged micro/nanorobots with bio-functionalized interfacial structure could also provide more universal approaches of biocompatible hybrid micro/nanorobots production. Hemoglobin assembled as erythrocyte-like microrobots displays a promising platform for cancer therapeutics owing to the inherent avoidance of immune phagocytosis and targeted transport of O_2_.^1233,1241^ These advancements allow micro/nanorobots to navigate and adapt to the complex biological environment, and contribute to a deeper understanding of biological processes and the further development of biomedical applications.

#### 6.3.2 Top-Down Fabrication

##### 6.3.2.1 Physical Vapor Deposition (PVD)

Exhibiting a distinctive asymmetric structure, Janus micro/nanorobots seamlessly integrate functional materials, each possessing distinct physical and chemical properties, on both opposing sides. The synthesis of Janus micro/nanorobots encompasses various stages and materials development. De Gennes first mentioned “Janus” to describe particles with two different hemispherical surfaces at the Nobel Prize awarding conference in 1991.^1242^ The preparation of Janus particles has always been a great challenge. Janus particles are classified from the perspective of the substrate materials that constitute them, which are mainly four types: inorganic, polymer, inorganic–polymer hybrids, and biohybrid particles.^1243^ Janus particles with different morphologies can be prepared from different substrate materials. Owing to the distinctive asymmetrical configuration of Janus particles, their broad application extends to the intricate design and production of Janus micro/nanorobots.

PVD stands out as the most prevalent method for the fabrication of Janus micro/nanorobots. PVD can easily deposit catalytic or magnetic materials onto the substrate material to prepare Janus micro/nanorobots with asymmetric structures. Inorganic particles (*e.g.*, SiO_2_, TiO_2_) are often chosen as the substrate material, and Janus micro/nanorobots are prepared by depositing metals with catalytic (Pt) or magnetic (Fe, Co, Ni) properties on the surface. Pt stands as the most extensively utilized material in catalytic Janus micro/nanorobots. SiO_2_ microspheres are sputtered with Pt to obtain the Janus Pt@SiO_2_ micro/nanorobots, that can be propelled in H_2_O_2_.^189^ To precisely regulate the motion, a self-propelled bimetallic Janus micro/nanorobots was designed by combining a catalytic material (Pt) with a magnetic material (Ni).^1244^ Ni was initially coated onto a SiO_2_ surface, succeeded by the deposition of a slender layer of Pt. SiO_2_/Ni/Pt micro/nanorobots can be propelled in H_2_O_2_, and their trajectory guided by an externally imposed magnetic field. Nevertheless, the utilization of H_2_O_2_ fuel in Pt/H_2_O_2_-based reactions has impeded the application of micro/nanorobots in biomedical contexts given the detrimental effects of H_2_O_2_ on biological systems. Water-driven micro/nanorobots were also investigated. Al-Ga alloys were prepared by microcontact printing technique and then half-coated with metal Ti by electron beam evaporation technique.^1087^ Al-Ga/Ti micro/nanorobots move by decomposing water into H_2_. Active metals can be an optimal choice for crafting Janus micro/nanorobots due to its biocompatibility and environmental friendliness. Degradable Mg/ZnO micro/nanorobots were crafted by using the interaction of Mg and water.^1245^ The monolayer of Mg particles was distributed on a glass sheet, and ZnO was sputtered onto the exposed facets of the Mg particles through electron beam evaporation (Figure 14f). This encapsulation minimizes the exposure of Mg during the subsequent reaction with water. Also, Zn/Fe Janus micro/nanorobots was prepared by depositing Fe on a hemisphere of Zn particles.^1245^ This self-destructing micro/nanorobots showed efficient movement and controlled degradation in biofluids. Beyond chemically propelled micro/nanorobots, Au assumes a pivotal role in micro/nanorobot preparation owing to its facile modification and excellent photothermal efficacy.^1246^ SiO_2_/Au micro/nanorobots driven by self-thermophoresis were obtained by partially coating Au onto the SiO_2_ particles.

##### 6.3.2.2 Glancing Angle Deposition

In 2007, at the Rowland Institute at Harvard University, United States, a Postdoctoral Fellow (Ambarish Ghosh) and a Rowland Fellow (Peer Fischer) considered how one may separate handed chiral propeller-shaped objects using physical forces. The idea was based on a theoretical prediction from Baranova and Zel’dovich, who had proposed that biologically important handed (chiral) molecules could be separated using circularly polarized microwave radiation.^1247^ The electric field was predicted to rotate dipolar molecules and thus induce corkscrew motion, which should drive the left- and right-handed molecules in opposite directions. However, to increase the magnitude of the effect and to permit the applications in water, the team at Harvard looked beyond molecules and at magnetic instead of electric fields. Indeed, fabricating corkscrew-shaped structures that could be dispersed in fluids was not so common and most previous demonstrations of magnetized corkscrews ranged anywhere between centimeters (from Tohoku, Japan^1248^) to tens of micrometers.^49^ A critical, innovative step used by Ghosh and Fischer was to employ a powerful physical vapor deposition technique called glancing angle deposition (GLAD), where the vapor from a heated material is incident on a substrate at an extremely shallow or “glancing” angle.^1249^ The shallow angle causes shadowing. Rotation of the substrate at optimal speeds then leads to the simultaneous growth of large numbers of helical corkscrews on the substrate (Figure 15g). In their first attempt, the group used GLAD to fabricate about one billion helical-shaped propellers per cm^2^.^51^ The propellers were typically 200–300 nm in diameter, about 1–2 μm long, and made from SiO_2_ with a ferromagnetic element integrated into the helical colloids. Incidentally, Howard Berg, Linda Stern, and co-workers were also working at the Rowland Institute at the same time, and their research interest was to elucidate the mechanism by which bacteria swim using corkscrew-shaped flagella.^1250^ It was thus natural to examine if the GLAD corkscrews could also be moved through liquids with the same finesse that is seen in bacteria. Indeed, the nanopropellers could, despite their small size, be manipulated with very high spatial precision through water using only small (∼5 mT) rotating magnetic fields.^1251^ The nanorobotic system thus consists of the magnetic propellers and a suitable magnetic coil system to induce motion. The scalable and multi-material fabrication technique and the exquisite control achievable at such small sizes suggested tremendous technological and commercialization potential (US Patent: [US20110270434A1]), some of which have already been realized. Before the subtleties related to such small-sized helical robots and their possible applications are discussed, it is worth mentioning that the Baranova and Zel’dovich prediction was indeed demonstrated later, where a rotating magnetic field could separate left- and right-handed helical propellers.^1252^ An interesting question is how tiny these nanorobots can be and how size affects their swimming performance. The experiments and theoretical analysis showed that orientational diffusion about their short axis puts an ultimate limit on steerability even when the magnetic field strength is large enough to keep the magnetic moment always aligned to the applied field direction.^1253^ For example, a robot actuated at 70 Hz in water (viscosity ∼1 cP) will start losing its steerability in the chosen direction once the nanorobot length reduces below a micrometer. The counteracting measure will be to increase the field frequency nonlinearly, marked by a cubic dependence, or use a fluid of higher viscosity. For example, if one reduces the robot length to 100 nm, the same steerability can be obtained if the frequency could be increased 1000-fold, which is impractical, or when operated in a medium of viscosity of 1000 cP. Thankfully, many biological media are highly viscous (more than hundreds of cP), implying that these small-sized robots are ideal for such environments. Steerability is also a function of the shape and it was established that the optimal shape consists of about one helical turn.^400^ Significant efforts have been made to reduce the robot width and length, for which a scalable substrate patterning technique, i.e., block copolymer micellar lithography, has been used with GLAD evaporation with a cryogenically cooled substrate holder.^1254^ So far, the helical nanorobots fabricated by GLAD have widths ranging between 50 and 500 nm and lengths between 200 nm and 5 micrometers.

For biological applications, as we discuss later, the choice of materials is of paramount importance. Recently, helical nanorobots containing Fe, Au, and silica generally recognized as safe according to toxicity tests conducted with multiple cell lines and rodent populations. The no-observed-adverse-effect level was 55 mg/kg of mouse body weight, and the robots remained in blood circulation.^1255^ It has been possible to make the robots biodegradable in body fluids by using Zn and Mg as the scaffold material of the helical nanostructure.^1256^ While Co and Ni have been used as magnetic elements, Fe-based materials are preferred for biological applications. FePt is exceptionally biocompatible and has high remnant magnetization and is thus particularly promising for biomedical applications.^1257^ The fabrication technique also allows integration with other materials and configurations, thereby enabling record local surface plasmon sensitivities^1258^ or their application in nanorheological measurements.^1259^ By adding plasmonic nanoparticles, the nanorobots could also work as motile plasmonic tweezers to trap, transport, and release colloidal cargo at low optical powers.^1260^ To maneuver nanorobots in human blood, the corrosion from the ionic fluid was countered by incorporating a microwave-synthesized layer of ZnFe, which rendered the robots stable against physical magnetic field-induced agglomeration.^52^ Interestingly, the same ZnFe layer was shown to have high magnetic hyperthermia potential, allowing targeting and killing of cancer cells.^1261^ It is possible to integrate molecular moieties using chemical functionalization; for example, the movement of helical nanorobots through gastric mucin gels was achieved by surface-immobilizing urease on the robot surface.^1123^ In another example, the nanorobots’ surface charge played a vital role in their adherence near the cancer cells in the extracellular matrix of a cancer-normal cell co-culture, suggesting their applicability in cancer targeting.^1262^

The dynamics of helical nanorobots show not only corkscrew motion, but also tumbling and precessional motion, which can be shown to follow from the solution of the Stokes equations.^395^ The study of the dynamics has given rise to an interesting new technology, where the nanorobots could be used as mobile rheometers^1263^ to measure the mechanical properties of complex (*e.g.*, shear thinning, viscoelastic^1264^), and heterogeneous fluidic environments with very high spatial resolution and at high speed. In most microfluidic experiments, their motion is studied in a thin fluid layer (Hele–Shaw geometry) and the robots are typically within a few micrometers of the bottom surface.^1265^ The resultant fluid flow has been calculated and imaged, and can be understood primarily as a line of distributed rotlets superimposed on the flow. The tiny robots could be trapped and manipulated in an optical bowl, showcasing the combined effect of optical force and magnetic torque on the robots.^1266^ Typical nanorobots are subject to considerable thermal fluctuations, especially when placed in low-viscosity fluids such as deionized water. One effect of the thermal noise is to induce bistable dynamics under certain experimental conditions, where the helices randomly switch between tumbling and propulsion states.^1267^ In another case, it was shown that Brownian noise can induce thermal rachet-like behavior under the application of an external oscillating (not rotating) magnetic field. The form of the external magnetic drive was critical in this study. When the oscillating drive was symmetric, the robots moved back and forth like Purcell’s “reciprocal swimmer”.^1268^ However, the robots behaved autonomously^1269^ under optimally asymmetric oscillating magnetic fields, forming a new class of artificial active matter.^1031^ One imagines futuristic applications where external magnetic fields power a swarm of autonomous nanorobots whose collective properties are governed by their interactions with each other and the surrounding environment.

This powerful system has demonstrated various applications in microfluidics and biophysical investigations, and suggests a path toward targeted delivery. Their small size is an essential distinguishing feature as most biological environments are porous and highly heterogeneous on a submicrometer scale, allowing the nanorobots to be maneuverable only when they are below a certain size, as shown in hyaluronic acid gel^174^ and Matrigel.^1262^ For motion through the vitreous humor, it was necessary to make the robots slippery by functionalizing them with a perfluorocarbon surface coating.^54^ The nanorobots were shown to be internalized by living cells through endocytosis, and surprisingly they remained mobile post-internalization^1270^ and the cells remained viable, suggesting that this experimental system can be used to address important cellular biophysics questions.^137^ The nanorobots have been used to make microrheological measurements inside living cells^1271^ and to deliver genetic material at single-cell resolution.^1257^ Their small size allowed them to also be maneuverable within the dentinal tubules and could provide rich therapeutic functionality related to root canal treatments.^1272^ Some of these proof-of-concept technologies have been commercialized and startups have been incubated to translate this technology to clinics and the market. In the future, it should be possible to engineer the nanorobots further, such as by driving them to many more biomedically-relevant locations.

##### 6.3.2.3 Electrochemical Deposition

Electrochemical deposition is a convenient, cost-effective, and simply equipped technique for fabricating controllable and different-sized structures compared to PVD, and is now widely used in the preparation of micro/nanorobots. Among them, metals, alloys, magnetic materials, semiconductor materials, and conductive polymer materials have been fabricated.^1243^ Among these, commonly employed membranes include templates made of porous alumina and polycarbonate. The pore walls of the templates sculpt the shape of the deposited material, resulting in the distinctive structure of the micro/nanorobots. The uniform porosity of the template contributes to the mass production of micro/nanorobots with analogous structures. For example, Pt/Au nanorods were prepared from porous aluminum membranes and the resulting nanorods rapidly propelled in H_2_O_2_ solution.^26^ Magnetic materials can be seamlessly integrated through the sophisticated process of membrane template-assisted electrodeposition. The first flexible nanowire motor driven by magnetic force is made with partially dissolved weak Ag bridges connecting Au and Ni segments.^1273^ To fabricate micro/nanorobots with dual motion modes, rod-shaped Au/Fe nanomotors were fabricated, activated by visible light, and guided by an external magnetic field.^1274^ Ag layers were employed as working electrodes on an anodized aluminum oxide film, succeeded by the electrochemical deposition of Ag, Au, and Fe segments within the pores, subsequently undergoing additional heat treatment. This approach enables the cost-effective and scalable production of micro/nanorobots that are driven by visible light and magnetically controllable. An alternative electrochemical deposition approach to generate Janus objects is based on the concept of bipolar electrochemistry. Intrinsically, it allows breaking the symmetry of (semi)­conducting objects in solution *via* their polarization in the presence of an electric field.^1275^ The main advantage is that Janus particles can be obtained in the bulk of the solution without the need of an interface or surface to break the symmetry.^1276,1277^ This enables the rational design of Janus particles with very sophisticated surface structures and that cannot be obtained with any other physicochemical modification strategy.^1278,1279,1280^ The resulting asymmetric objects can then be directly used as microrobots.^1281^ Wang’s group has developed an effective and simple template electrodeposition method for the large-scale preparation of extremely small helical magnetoswimmers. Figure 15h illustrates the scheme for fabricating spiral nanoswimmers. In this method, the preparation of Pd nanospirals relies on the electrochemical co-deposition of Pd^2+^ and Cu^2+^ in the nanopore of the aluminum oxide membrane template in an acidic environment. Au nanorods are first electrodeposited inside the nanopore to form a uniform solid substrate. Pd/Cu nanorods were then grown using a solution containing PdCl_2_, CuCl_2_, and HCl. OH^−^ groups on the surface of alumina and H^+^ in an acidic solution, as well as suitable reduction potentials, are necessary for the effective reduction of Pd^2+^ to form a crystal structure on the nanopore wall and the etching of Cu from the nanorods produces Pd nanospirals. The electron beam then vaporizes the nanometer-thick layer of magnetic nickel onto the Pd nanospring, forming a tiny magnetic spiral nanoswimmer.^1281^

##### 6.3.2.4 Enzyme Janus Structure

High catalytic efficiency and biocompatibility of enzymes, compared to inorganic catalysts, make enzymes more competitive components for micro/nanorobots. While noble metal catalysts are very efficient and extensively used as model catalysts in active matter and environmental applications, the toxicity of the high concentrations of H_2_O_2_ required for propulsion limits their use in biomedical applications. Alternatively, enzymes have emerged as a biocompatible alternative with high versatility in enzyme–substrate configurations. Since the first examples of bi-enzymatic reactions used to power large fibers at the air–liquid interfaces^200^ to the propulsion of multi-walled carbon nanotubes,^201^ the field has grown in the pursuit of more biocompatible combinations of fuel–substrate using enzymatic reactions. For instance, catalase and urease have been the most commonly used enzymes, constituting about two-thirds of all enzymatically powered micro/nanobots reported so far.^202^ Catalase was used to replace Pt inside the walls of tubular microjet engines, enabling the reduction in the concentration of H_2_O_2_ fuel.^33^ Other enzymes, like lipase,^1282,1283^ collagenase,^203^ acetylcholinesterase,^153^ glucose oxidase,^35^ combinations of glucose oxidase and catalase,^204^ trypsin,^205^ aldolase, and others have been used as well as combinations of enzymes and inorganic catalysts. A comprehensive review of the different types of enzymes, the types of motors, materials, shapes, and sizes have been recently reported.^202^ The influence of fundamental aspects of enzyme catalysis such as conformational changes and catalytic turnover has been studied.^153^ The effect of the ionic media where they navigate was also investigated for micrometer-sized motors.^1284^ Moreover, some enzymes are very sensitive to the way they are bound to the surface of the micromotors as described for spherical nanomotors using lipase where, depending on whether the hydrophobic or hydrophilic domains are linked, they present different performances. Purification of as-received commercial enzymes is key for improving the motion of micromotors as well as for ensuring their reusability.^1285^ Enzyme amount and location are of paramount importance in the performance of micro/nanomotors as demonstrated for urease-based motors. A seminal paper described that molecular asymmetry observed by super resolution microscopy is sufficient for motors to present self-propulsion beyond the physical asymmetry presented for traditional Janus particles.^1097^ The same results were demonstrated for nanomotors, where it was also reported that the protein corona was reduced in the presence of enzyme coating.^1286^ Beyond inorganic materials, MOFs,^207,1287^ PLGA, coacervates, DNA nanotubes, and liposomes^209^ among others, can also be used as chassis for the motion of enzyme motors.^208^ Tactic phenomena were described for urease and catalase motors^211^ and, later on, following the Hoffemeister series for urease–liposome-based nanomotors.^38^

Enzymes like urease have been demonstrated to be extremely useful in biomedical applications. Such enzymes decompose the urea contained in urine.^1288^ Urease micromotors enhance the delivery of anticancer drugs in HeLa cells and can be coated with DNA switches for pH sensing and with molecular gates for triggered release specifically into tumor cells. Antibody-coated nanomotors have shown targeted penetration into bladder tumor spheroids *in vitro*. In this regard, the collective motion of radio-labeled urease-nanomotors was imaged by PET-CT imaging techniques in the bladders of living mice.^229^ Recent studies demonstrated that these nanomotors accumulate in the tumor site and can reduce the bladder tumor size by 90% with only one intravesical administration.^37^

Catalase-based nanomotors have also been used in the form of single particles,^1289^ clusters^1290^ and swarms of nanomotors.^1291^ When clusters or large populations of catalase-nanomotors are placed in H_2_O_2_ fuel, they generate O_2_ bubbles, which can be used both for propulsion and for the disruption of mucus. The latter has been recently demonstrated *in vitro* in human colon cells and *ex vivo* in mucus-secreted tissues from mice.^1291^ Zhou *et al*.^1292^ prepared two enzyme-catalyzed reaction-driven nanomotors by loading glucose oxidase and catalase into the cavities of poly­(pentose) flask-like colloidal particles with different surface wettability.

##### 6.3.2.5 Lithography

Lithography technique utilizes the characteristics of photosensitive compounds (photoresists) to chemically react with light, transferring the pattern onto the substrate material, thereby achieving the fabrication of micro/nanostructures.^1293^ With the demand for high-performance miniaturized systems, the development of lithography technology based on new materials and processes continues. Lithography technology includes photolithography,^1294^ electron beam lithography,^305,306,1295^ focused ion beam lithography,^1296^ nanoimprint lithography,^1297^ deformation lithography (*e.g.*, wrinkling,^1298^ cracking,^1299^ collapsing^1300^), and colloidal lithography.^306,307,1301^ Complex micro/nanostructures can be fabricated through lithography technology, such as micro-mechanical devices,^1302^ microfluidic channels^1303^ (*e.g.*, linear, curved,^1304^ Y-shaped,^1305,1306^ and other microfluidic channels), nanowires,^1307,1308,1309^ *etc*. These structures can be used to fabricate various parts of micro/nanorobots, such as sensors and actuators, in order to achieve the desired motion and function.

Photoresists are mainly used in lithography technology for fabricating micro/nanorobots due to their high resolution and controllable photochemical reactions. Polymers such as polyester and polyolefins exhibit good plasticity, mechanical properties, and processability. They are both cost-effective and abundant. Combining technologies like 3D printing with lithography^1310^ can also fabricate micro/nanorobots. Nonetheless, the plasticity of polymers may lead to issues like deformation and creep, and some polymers are unstable under specific conditions. In addition to polymers, lithography technology can also be combined with techniques like electron beam evaporation, sputtering, or electrochemical deposition to deposit metals (*e.g.*, Au, Pt) onto nanoparticles and fabricate micro/nanorobots;^1311^ for example, Janus particles can be half-covered in metal to be driven by chemical reactions.^1195^ This approach offers good stability but the preparation process is relatively complex and some metals may be harmful to the biological environment. Moreover, semiconductor materials, such as Si and SiC, possess excellent electrical properties and controllability to fabricate photo-driven micro/nanorobots; however, they are expensive and require extensive preparation conditions. Composite materials, such as metal–polymer and metal–semiconductor composites, combine the advantages of different materials to achieve outstanding mechanical, chemical, and electrical properties. Additionally, the distinct components of composite materials can fulfill various functions, such as magnetic and catalytic propulsion. With the continuous development of materials and lithography technology, there will be more suitable materials to meet the needs of lithography technology.

By utilizing traditional lithography techniques, various micro/nanorobots shapes can be fabricated on a two-dimensional plane. Through post-processing methods, such as peeling or folding, these planes can be transformed into three-dimensional structures (Figure 15g).^1312^ Currently, research has demonstrated that lithography technology can convert polyelectrolyte multilayer films into microtubules and further into microrockets. By incorporating Pt nanoparticles, microrockets with high speed and significant drag force can be achieved.^1209^ This advancement better aligns with the performance requirements of micro/nanorobots. With the continuous advancement of technology, three-dimensional lithography techniques, such as two-photon polymerization, can directly manufacture micro/nanorobots with precise three-dimensional shapes.^1313^ These micro/nanorobots can be manipulated to navigate in three-dimensional space by responding to external stimuli, such as light and magnetic fields. This capability enables the fulfillment of the motion requirements for micro/nanorobots through the application of external conditions. Moreover, different lithography techniques can also be combined with metal deposition and wet/dry reactive ion etching to achieve high versatility in defining asymmetric nanomotors made of organic or inorganic materials.^306,307^ Under this context, colloidal lithography, including physical deposition techniques and reactive ion etching (RIE), has been successfully used to fabricate multicomponent nanomotors composed of both organic and inorganic materials. In this process, multicomponent materials are deposited layer by layer onto a substrate in an additive manner, using physical evaporation methods or spinning processes. Colloidal nanoparticles then self-assemble and are used as masks for selective etching of components via dry etching (RIE) with appropriate gases, depending on the material to be etched. This enables the creation of multicomponent nanorod structures, which are subsequently released into the water medium through sonication, scraping of the support, or by utilizing sacrificial layers. The diameter of these nanorod motors can range from 100 nm to a few micrometers.

Meanwhile, micro/nanorobots can undergo functionalization through lithography. This process involves introducing various functional materials or modifiers, such as photosensitive polymers,^1314^ metal nanoparticles,^1195^ and biomolecules,^1315^ to achieve diverse functionalities for micro/nanorobots. These functionalities include light sensing, chemical reactions, biometric recognition, and more. Current research has demonstrated the ability to assign specific chemical functions to the surface of micro/nanorobots through chemical grafting or physical adsorption.^1086^ Alternatively, functionalized material can be introduced to provide responsive performance. For instance, magnetic or fluorescent material can be incorporated to facilitate the movement of micro/nanorobots through external manipulation using magnetic fields or light. This can be achieved by fabricating Janus particles. Furthermore, lithography technology is also employed in the preparation of Janus particles, which can be modified to adapt and fulfill different roles in diverse environments.^1195,1316^

Although lithography technology offers numerous advantages in the fabrication of micro/nanorobots, it is not exempt from challenges. One key challenge is the continuous improvement of resolution and fabrication speed, as well as the reduction of associated costs. Currently, no single lithography technology fully meets all the requirements for manufacturing nanoscale features. Therefore, a combination of different lithography techniques is often employed to overcome the limitations of each technique. As a result, current research focuses on enhancing the resolution and speed of lithography processes, while also exploring cost-effective alternative solutions. Overall, lithography remains a promising manufacturing method for producing micro/nanorobots with exceptional accuracy, flexibility, and functionality. It is expected to maintain its prominent and preferred status for manufacturing precise, flexible, and functionalized micro/nanorobots.

##### 6.3.2.6 3D Printing

3D printing is the fabrication method most likely to produce complex structures and high-resolution nanorobots.^1317^ Research has shown that the IP-series polymers (IP-L,^1094^ IP-DIP^1318^) are used to fabricate 3D-printed nanorobots and extensively utilized for printing small and arbitrary substructures by two-photon polymerization, using a photosensitive material to form the exact pattern with a focused laser.^1317^ The IP-series polymers applied as photoresists in the two-photon polymerization system can improve the resolution and precision of nanorobots, enabling high stability and low shrinkage characteristics to print small feature structures. However, although IP-series polymers have better biocompatibility, it is difficult for the human body to metabolize and excrete them. Similarly, SU-8 has the same concerns as a photoresist.^1319^ Therefore, it is essential to seek biocompatible and biodegradable materials for the fabrication of medical nanorobots.

The development of advanced materials has made a hot research topic of biodegradable hydrogel polymers. PEG-series has excellent biodegradability and biocompatibility, which can be applied to a variety of applications such as drug delivery. The incorporation of photoinitiators or photosensitizers with PEG-series compounds (*e.g.*, PEGDA) is employed to fabricate highly biodegradable nanorobots using two-photon polymerization^1320^ and stereolithography.^1321^ Stereolithography is a manufacturing technique that constructs structures layer-by-layer from curable liquid materials using UV light.^1322^ However, PEGDA must compensate for its lower mechanical strength; hence, the use of hybrid gels in the fabricated nanorobots enables them to exhibit advantages in terms of cross-linking as well as mechanical strength (Figure 15h).^1323^ Similarly, gelatin methacrylates have the same effect with fast UV curing and have lower toxicity compared to PEG.^366^

3D-printed fabricated nanorobots can be functionalized using special materials, usually to confer magnetic properties. Soft paramagnetic particles, including Ni,^1318^ Fe,^367^ Fe_3_O_4_,^376^ show high saturation magnetization lightness, magnetization rate, and excellent biocompatibility.^812^ The surface of the nanorobots can be coated with magnetic particles or combined with the aforementioned materials during fabrication.^1324^ This approach not only equips the nanorobots with magnetic propulsion but also enables controlled pathways for continuous or triggered cargo release, enhancing their functional capabilities.

#### 6.3.3 Utilizing Wet Chemical Etching for Structural Modification

Unlike additive manufacturing techniques, subtractive manufacturing involves creating structures of the desired shape and size by removing material. Due to its wider range of material compatibility, subtractive manufacturing methods are also utilized in the production of micro/nanorobots. Two common methods of subtractive manufacturing are wet chemical etching (WCE) and dry etching. WCE is a technique in which materials are etched by immersing them in an etching solution. It is suitable for almost all inorganic and organic materials. Furthermore, WCE offers advantages such as strong adaptability and good surface uniformity. Notably, wet etching is more suitable for the mass manufacturing needs of micro/nanorobots compared to dry etching.

WCE can selectively remove materials, providing them with powerful structural modification capabilities. This allows it to be combined with most fabrication methods for micro/nanorobots, thereby expanding their structural versatility. The closest integration with WCE is template-assisted electrodeposition, which is the most used method for fabricating rod-shaped micro/nanorobots. The modification of the rigid–flexible properties of rods can be achieved using WCE. By depositing Ag segments inside the rods and subsequently partially etching them with WCE, Ag segments can be turned into flexible joints. Utilizing the flexible characteristics of Ag segments in combination with the magnetic actuation ability of Ni segments, it is possible to fabricate micro/nanorobots that swim like fish^1325^ or freestyle swimming like humans.^417^ In addition, rods can be modified to have defects at one end by removing sacrificial materials, such as polystyrene spheres, pre-filled in the membrane template using WCE. It is worth mentioning that this structure allows for faster speeds under ultrasonic driving.^460^ In addition, by removing Cu from rods obtained through the co-deposition of Cu and Pd using WCE, the rod structure can be modified into a helical structure.^1326^ This method even allows for the fabrication of composite structures combining rods and spirals.^87^ In addition to combining with template-assisted electrodeposition, WCE allows selective material removal from bi-spherical structures fabricated using microfluidic synthesis methods with phase separation techniques, modifying them into spherical structures with depressions.^1327,1328^ Furthermore, by removing the spherical substrate of Janus spheres fabricated using PVD through WCE, a hemispherical shell structure consisting of a functional coating can be obtained. Compared to the convex catalytic structure of Janus spheres, the concave surface is more favorable for bubble generation, resulting in enhanced kinematic performance.^1329^ Additionally, micro/nanorobots with this shell structure can be driven by ultrasound and exhibit efficient cargo transport.^1330^ WCE can also be used to modify biological structures, thereby conveniently providing more distinctive structures for micro/nanorobots. For example, sunflower pollen can be transformed into a hollow sea urchin-like micro/nanorobot using WCE.^1331^ The large inner cavity structure of sunflower pollen offers strong cargo loading capacity while the ordered spikes on its surface facilitate efficient cell penetration.

In addition to modifying the overall 3D structure, WCE can also alter the surface microstructure of micro/nanorobots, significantly enhancing their performance. WCE is isotropic, allowing for the creation of uniform surface microstructures. For instance, after full-surface etching, Fe_3_O_4_ particles exhibit a dense burr-like microstructure on their surface, which increases their specific surface area and active sites, thereby enhancing their motility and cargo loading capacity.^1332^ Moreover, localized microstructures can be achieved by selectively etching the surfaces of rods fabricated using template-assisted electrodeposition.^1333^

In addition to modifying structures made by other fabrication methods, WCE plays a crucial role in the final release of micro/nanorobots in many fabrication techniques. For instance, in template-assisted electrodeposition, WCE is necessary to remove the template and release the micro/nanorobots.^185,416,483^ In addition, WCE is essential for fabricating tubes and spirals using the self-rolled method. The core of this method involves releasing stresses within the functional film material. WCE is the most commonly used technique to achieve stress release in functional thin film materials by etching the sacrificial photoresist layer, thereby facilitating stress relief in the functional material film.^185^

In conclusion, WCE exhibits powerful structural modification capabilities, offering substantial potential for developing multifunctional and high-performance micro/nanorobots with complex structures. However, challenges in controlling fabrication accuracy and surface quality have constrained the advancement of high-performance micro/nanorobots with intricate overall and surface microstructures. Thus, the future focus should be on developing more selective and controllable WCE methods to enhance the fabrication of high-performance micro/nanorobots.

### 6.4 Imaging Modalities and Materials for Micro/Nanorobots

For precise navigation and manipulation of micro/nanorobots, real-time observation of their positions is essential, demanding efficient imaging modalities. *In vivo* imaging faces challenges, including tissue thickness and interference from blood flow, making it impractical to effectively monitor the precise position and motion of individual micro/nanorobots. The use of micro/nanorobot collectives can provide better contrast due to high agent concentration. Systems involving medical imaging show promising applications in localizing and tracking swarms of microrobots, such as fluorescence imaging,^123,375,1335^ magnetic resonance imaging (MRI),^375,1336,1337^ ultrasound (US) imaging,^372^ positron emission tomography (PET),^1338^ and optical coherence tomography imaging. These well-established techniques find extensive use in hospitals and clinics, seamlessly integrating with microrobotic control systems.

#### 6.4.1 Radionuclide Imaging

Radionuclide imaging (RI) has gained recognition as a highly effective modality for in-depth tissue examination and imaging due to its extensive scanning range, sensitivity, accuracy, and noninvasiveness.^1339^ RI employs minute quantities of radionuclide material to diagnose and evaluate various diseases within the body. Radionuclides possess the inherent property of spontaneous radioactive decay, during which the nuclide emits charged particles such as gamma or beta rays. The information pertaining to these rays, as captured by the detector, is converted into a digital signal and subsequently processed and reconstructed by a computer. Commonly encompassed within RI are techniques such as positron emission tomography (PET), single photon emission computed tomography (SPECT), and γ-scintigraphy.^1340^ Noteworthy radionuclides employed in conjunction with nanomaterials include Tc-99m,^1341^ Zr-89,^1342^ I-131,^1343^ Ra-233,^1344^ C-14,^1345^ Cu-64,^1346^ Lu-177,^1347^ and Ga-68,^1348^ among others. Nuclides that are clinically approved for use today include Tc-99m for sentinel lymph nodes^1349^ and Y-90/Ho-166 for liver radioembolization.^1350^

Incorporating radionuclides into nanoparticles can be accomplished by encapsulating the nuclide within nanostructures, employing physical adsorption or electrostatic interactions, or immobilizing them through chemical functionality.^1351^ During the preparation of micro/nanomotors, factors such as the mode of actuation, the surrounding humoral environment, and other conditions must also be taken into account. RI allows for the *in vivo* tracking of radiolabeled micro/nanorobots over several hours, which holds great significance for controlling their movement and biomedical applications.^1352^ For instance, Vilela *et al*. utilized PET-CT to monitor bubble-propelled microtubules functionalized with I-124.^1338^ These microtubules were prepared using template-directed electrodeposition and metal evaporation processes. However, it is important to acknowledge that exposure to ionizing radiation is inevitable during the RI procedure, and striking a balance between the radiation dose and imaging performance remains a concern. Furthermore, due to the lengthy data acquisition time associated with PET and SPECT, promptly tracking the positional movement of micro/nanorobots may not always be feasible. Augmenting RI with other imaging modalities may thus serve as the most effective approach to compensate for the spatial and temporal resolution limitations.

#### 6.4.2 Photoacoustic Imaging

Photoacoustic imaging is a noninvasive technology with broad applications in early cancer diagnosis, detecting oxygen supply to organs, and identifying other diseases and for scientific research.^1353^ The technology is based on the photoacoustic effect discovered by Bell in 1880,^925^ where biological tissue absorbs energy from pulsed laser light, causing thermal expansion and the production of sound waves. Ultrasonic transducers receive and reconstruct the ultrasonic signal and finally present the image of the imaging agent in the biological tissue.^1354,1355,1356^

Photoacoustic imaging currently offers significant advantages for *in vivo* imaging of micro/nanorobots by combining optical and acoustic imaging. This combination achieves higher spatial resolution ranging from a few micrometers to 100 μm, video imaging speed up to 50 Hz, and imaging penetration depth of a few micrometers to 10 mm.^1357^ In addition, using photoacoustic imaging in low-energy fields can produce more signals while causing less damage to biological tissue.^123,1358^

As technology has advanced, photoacoustic imaging has expanded into various applications, such as optical resolution photoacoustic microscopy (OR-PAM), photoacoustic computed tomography (PACT), and fast-scanning OR-PAM. PACT, as a crucial branch of photoacoustic imaging technology, represents an exceptional optical imaging technique that overcomes the optical diffusion limit, allowing for high-resolution deep-tissue images using optical contrasts. With its remarkable spatiotemporal resolution, substantial penetration depth, and capability to provide both anatomical and molecular contrasts, PACT shows great promise for imaging a variety of micro/nanorobots.^1141,1359^ It can monitor the real-time locomotion process of micro/nanomotors in the body and their effectiveness in drug release as a drug carrier. Currently, real-time signal tracking in isolated tissues can reach a depth of 10 mm.^1141,1360^ General biomedical imaging technology, including PCAT, can only observe the motion trajectory or tissue distribution of micro/nanomotors in a macroscopic way, with a current resolution limit of 125 μm.^1361^ The superior resolution of OR-PAM is beneficial for visualizing superficial tissues, achieving a resolution of up to 3.2 μm. Nevertheless, its lack of real-time imaging capability represents a notable limitation. Fast-scanning optical-resolution photoacoustic microscopy (OR-PAM) integrates the strengths of photoacoustic computed tomography (PACT) and traditional OR-PAM. It offers high-resolution imaging (∼6.3 μm) for real-time tracking of the shallow epidermis and enables precise, real-time visualization of micro/nanomotors.^969,1141,1362,1363,1364^

The imaging conditions for photoacoustic imaging technology typically fall within the visible infrared band, so the treatment of diseases using micro/nanorobots should be combined with ultrasonic imaging technology. The prepared materials should exhibit visible light luminescence and a high NIR light absorption rate to make them an ideal photoacoustic contrast agent. The material selection can be broad, including metals, quantum dots, polymers, fluorescent dyes, and natural pigments. Metals with superior NIR absorption properties, such as Au, Ni, and Ti, have been widely used in the optoacoustic imaging applications of micro/nanomotors. It is well-documented that specific biological tissues or tissue fluids possess the ability to absorb light across various wavelengths. For example, the presence of blood will significantly elevate the level of background noise in photoacoustic imaging. The method is adequate for displaying the photoacoustic signal, which distinguishes it from the blood background at shorter wavelengths, leading to a more distinct imaging outcome. Moreover, it is capable of producing a more potent photothermal driving capacity at extended wavelengths.^969,1360,1362,1365^ Quantum dots, similar to other light-responsive materials, are distinguished by their diminutive dimensions and exceptional biocompatibility. For example, black phosphorus quantum dots and micro/nanorobots function as carriers to mitigate the susceptibility of the quantum dots to oxidation and degradation upon exposure to H_2_O and O_2_. This functionality enables micro/nanorobots to perform live imaging while under 808 nm laser exposure and to produce ROS to treat tumors.^1366^ Some polymers, including polydopamine, NIR zone I/II fluorescent dye DPP-BT, and natural chlorophyll materials exhibiting photoluminescent properties, have been employed in the realm of bioimaging for micro/nanomotors.^1367,1368,1369^

Based on existing research, photoacoustic imaging encounters difficulties in achieving real-time, high-resolution, and sufficient depth imaging simultaneously as a modality. As a result, it is commonly combined with nuclear magnetic imaging, ultrasonic imaging, fluorescence imaging, and other technologies. The application of multi-modal imaging requires the deployment of micro/nanorobots capable of meeting the essential requirements of multiple imaging technologies simultaneously. For example, Ni demonstrates both photothermal properties and outstanding magnetic characteristics, rendering it suitable for applications in nuclear magnetic imaging and photoacoustic imaging. Certain fluorescent dyes can be employed for both photo-ultrasonic imaging and fluorescence imaging.

Photoacoustic imaging is widely recognized as an advanced and thorough biomedical imaging technique for real-time monitoring of micro/nanomotors within living organisms. Nevertheless, due to the underdeveloped nature of the algorithm and its restricted depth of penetration, its application in clinical settings has not been realized.^1370^ The continued advancement of photoacoustic imaging technology, particularly its integration with ultrasound, nuclear magnetic resonance, and other modalities, is poised to greatly enhance its utility. These innovations are expected to play a pivotal role in advancing disease treatment through the use of micro/nanorobots.

#### 6.4.3 Ultrasonic Imaging

Ultrasound imaging stands as a widely used, well-established, and radiation-free medical imaging technique. This imaging method relies on the interaction of reflected acoustic waves with the objects being imaged and their surrounding environments. In comparison to other medical imaging technologies, such as MRI and CT, ultrasound imaging offers high temporal resolution, ensuring fast imaging speed. This temporal precision is crucial for acquiring real-time feedback of moving small-scale objects.^365^ When ultrasound waves encounter an interface between two media with different acoustic properties, these waves are partially scattered, partially reflected, or absorbed.^1371^ Ultrasound imaging is based on the interaction of high-frequency sound waves and tissues with variable reflective properties, which produce reflected sound waves to create an image. The frequency of the ultrasound wave determines the depth of penetration and resolution. Higher-frequency ultrasound allows for higher resolution while tissue penetration is better at lower frequencies.

Ultrasound imaging is noninvasive, real-time, portable, safe, provides high temporal resolution, is cost-effective, requires no contrast agent, and has a high depth of tissue range. Used to track micro/nanomotors in biological tissues with minimal adverse effects, it provides real-time feedback on their location, allowing real-time control. However, low signal-to-noise ratio, low resolution, and interference by bone introduce limitations in terms of localization errors, background signal, and artifacts.^950^ Ultrasound imaging systems operate at frequencies between 1 and 100 MHz^1371^ and imaging relies on high-frequency acoustic waves, typically in the diagnostic range of 1–40 MHz, and primarily on high-frequency acoustic waves of 2–18 MHz,^1352^ and provide a spatial resolution of millimeters to micrometers, a temporal resolution of seconds to minutes,^263^ and a penetration depth of 1–10 cm.^123^

Currently, bubble-driven and enzyme-driven micro/nanomotors, as well as magnetic-trait micro/nanomotor clusters, have been utilized in ultrasound imaging techniques for tracking inside biological tissues. Two different ultrasound imaging modes, B-mode and Doppler mode, are mainly used for micro/nanorobot tracking. For bubble-driven micro/nanomotors, B-mode imaging can be utilized due to its possible resolution of sub-millimeter accuracy, which makes it possible to detect and localize individual microbubbles. For example, both Olsen *et al*.^1372^ and Sánchez *et al*.^1373^ performed ultrasound imaging by using micro/nanomotors to track the trajectory of O_2_ microbubbles produced by H_2_O_2_. For the ultrasound imaging of bubble-driven micro/nanomotors, the main limitation of the system is the self-propelled micro/nanomotor that the system tracks through indirect imaging of the microbubbles. The imaging system detects the microbubbles responsible for propulsion, not the micro/nanomotor. Therefore, the acquired data points must be processed to provide the exact position of the micro/nanomotor.^1356^ Moreover, the presence of cytotoxicity of H_2_O_2_ fuel and gaseous products (O_2_ or H_2_), which are usually involved in the catalytic propulsion process, may pose a high health risk to the human body. Enzymes, as biocatalysts, have emerged as an alternative to catalytically propelled micro/nanomotors for biomedical purposes due to their high biocompatibility. Xu *et al*.^1374^ proposed urease-driven liquid metal-based nanorobots. Polydopamine encapsulated liquid metal particles with grafted urease and drug molecules (*e.g.*, cefixime antibiotic trihydrate) on the outer surface provided the possibility to be detected with ultrasound. An alternative strategy for achieving direct ultrasound imaging of microrobots involves entrapping microbubbles within the microrobot. The oscillation of these entrapped microbubbles facilitates acoustic propulsion, while their high imaging contrast ensures high-resolution in vivo imaging with ultrasound.^1375^

Several studies have demonstrated ultrasound imaging of micro/nanorobots driven by magnetic fields in the absence of any air bubbles, *e.g.*, Wang *et al*.^1190^ detected a diffusion–reaggregation process of magnetic nanoparticles within an isolated organ. In addition, the combination of ultrasound imaging and magnetic control systems can enable precise motion of the micro/nanomotor, as demonstrated by Khalil *et al*.^1376^ who used an ultrasonic feedback system for wireless motion control of magnetic particles.^1352^ For magnetically driven micro/nanorobots, clusters are particularly easy to image because they are much larger than a few micro/nanorobots. And they provide better imaging contrast to close the loop of image-guided control.^1377^ Moreover, real-time tracking and navigation of magnetic nanoparticle populations can also be applied to Doppler imaging modalities. Doppler imaging relies on the Doppler effect, a traveling frequency shift caused by the interaction between ultrasound waves and moving objects.^1378^ Depending on the extent of the wave red (or blue) shift, the speed at which the wave source is moving in the direction of observation can be calculated. This technique has recently been investigated for localization. Doppler imaging has also been applied to real-time tracking and navigation of nanorobot populations. Wang *et al*.^496^ reported a strategy to navigate microswarms in real time under ultrasound Doppler imaging guidance for active endovascular delivery.

Currently, ultrasound imaging is used in combination with other imaging techniques to overcome the limitations of ultrasound imaging and to reduce the material limitations of the motor. To maintain temporal resolution and penetration depth while improving spatial resolution and molecular specificity, scientists have developed a hybrid optical ultrasound imaging technique, also known as photoacoustic imaging. Medina-Sánchez *et al*. proposed the use of photoacoustic imaging for the visualization of moving medical micromotors.^1379^ This method requires that the dimensions are within the spatial resolution and that it is made of IR-absorbing materials, such as metal films or nanomaterials.^1360^ In addition, ensuring that the motion change signal is stabilized when performing ultrasound imaging and ensuring the consistency of the micro/nanorobot throughout the detection process will be required for the development of micro/nanomotors.

#### 6.4.4 Magnetic Resonance Imaging

MRI is a radiological technique employed to capture detailed images of the body’s anatomy and physiological processes. MRI, with the advantages of nonionization, high spatial resolution, and free radiation, is a noninvasive diagnostic tool for analyzing internal structures by providing 3D tomography images of soft tissue with high resolution and better contrast, and is widely used in the clinic. Based on the principle of atomic spin relaxation, MRI imaging obtains physical and chemical information about the molecules at the imaging location by collecting the electrical signals and energy emitted by the nuclei of abundant hydrogen atoms in tissues and biological materials under the action of high-intensity static magnetic fields.^1356^

In the field of microrobotics, magnetic materials in hybrid micro/nanorobots, in addition to being used as driving media, are often also used as contrast agents for targeting motion and MRI-based tracking. Researchers are exploring the use of MRI scanners as auxiliary tools for navigation control within vascular networks. MRI imaging puts forward size requirements for the magnetic contrast agent in the micro/nanorobot. Under a magnetic field, the medical contrast agent can amplify the image about 50 times. That is to say, when using MRI to track the locomotion of micro/nanorobots, the magnetic material they carry should not be less than 1/50 the size of the robot to obtain a reliable imaging performance.^1353,1380^

Servant *et al*.^1381^ demonstrated for the first time the concept of *in vivo* directed motion using a clinical MRI platform. They successfully controlled and tracked magnetic beads in the carotid arteries of live pigs, establishing the feasibility of untethered devices or robots for *in vivo* navigation. Zheng *et al*.^1382^ constructed Gd-doped NIR-driven nanorobots that can locomote under NIR, enabling accumulation and deep penetration in solid tumors while providing MRI contrast in tumor tissues. Yan *et al*.^375^ prepared Fe_3_O_4_-containing spiral algae-based microrobots using a one-step method. Driven by an external magnetic field, the robot population in the stomach of rodents was noninvasculatively tracked by MRI. This work demonstrates the potential of image-guided therapy for microbial robotic vectors and broadens the application of MRI-guided nanobots.

The spatial and temporal resolution of MRI is a major challenge in the field of micro/nanorobots. In general, MRI can only be used to detect micro/nanorobot locomotion as clusters, not individually. While increasing the MRI acquisition time can enhance image contrast, it comes at the expense of reducing the frame rate and time resolution. In addition, when conducting magnetic navigation and MRI-integrated systems for micro/nanorobots, researchers must take into account the magnetic interference of two different signals. Therefore, operating both types of magnetic fields simultaneously is a challenge and reduces the feasibility of operating in real-time. Alternatively, there may be a delay between the microrobot drive and image processing when two fields are applied simultaneously.^1356,1383^ By combining MRI with other imaging strategies, such as fluorescence imaging, it is possible to minimize the delay and instability generated during the switching process.^1339^ Despite the incompatibility of MRI with traditional magnetic actuation systems, the tracking and control of microswarms can be achieved through alternative methods or by utilizing the gradient coil for magnetic actuation.^372^

#### 6.4.5 Magnetic Particle Imaging

The concept of magnetic particle imaging (MPI) was proposed by Bernhard Gleich and Jurgen Weizenecker at the Philips Research Laboratory in Hamburg in 2001 as an emerging tomography technique. MPI can track and quantitatively measure the spatial distribution of superparamagnetic iron oxide nanoparticles and can detect iron particles with concentrations close to picomole level.^1384,1385^ Magnetic nanoparticles are the contrast agent and the only signal source of MPI. By collecting the nonlinear magnetization behavior of nanoparticles in the applied magnetic field to reconstruct the image of their concentration, tissue penetration, and quantifiable signal strength can be maximized.^1386^

In 2019, Bakenecker *et al*.^1387^ coated magnetic nanoparticles onto a 3D-printed model to obtain a spiral microrobot. The MPI scanner ran alternately in the imaging and driving modes to realize the tracking and navigation of micro/nanorobots in the vascular model, which verified the possibility of 2D visualization and driving. In 2021, his research group further improved and developed a smaller spiral magnetic microrobot, which navigated toward the aneurysm model by rotating the focal field of the MPI scanner through the anatomy of the middle cerebral artery of human beings.^1388^

In contrast to MRI, clusters of magnetic nanoparticles that produce stronger imaging signals than individual ones, individual magnetic nanoparticles produce stronger MPI signals and therefore can be used to track the locomotion of individual micro/nanorobots. The process of drug release *in vivo* can also be tracked quantitatively through continuous monitoring of MPI signal changes.^1389^ In addition, when using MPI to visualize magnetically actuated micro/nanorobots, the magnetic fields of existing MPI scanners can be used for magnetic propulsion, so there is no need to integrate imaging technology and actuation devices. On the other hand, a significant disadvantage of MPI is the lack of morphological information, so it is inevitable to integrate with other imaging methods, such as MRI and CT imaging. Further development of specialized tracers is also needed to improve the current spatial resolution at the millimeter level.

#### 6.4.6 Fluorescence Imaging

Fluorescence, a prevalent luminescent phenomenon in nature, can be harnessed to track micro/nanorobot swarms.^1339,1356^ The preparation of various building blocks involves introducing fluorescent substances, primarily by incorporating intrinsically fluorescent materials for fabrication and functionalized agents with fluorescent probes.^128^ Nanorobots are different from the environment when combined with markers such as fluorescent probes and organic dyes. On the one hand, this improves visibility of the position, indicating a significant advantage in biological imaging technologies.^1383^ Moreover, fluorescence imaging has the advantages of low cost, strong operability, and high safety, and is widely used in the field of biomedicine.^950^

As early as 2002, Li and Tan fabricated a single DNA nanomotor with both fluorophore and quencher organic molecules, which enables real-time observation of DNA nanomotor movements through monitoring fluorescent signals.^1390^ Since then, the use of fluorescent probes to characterize nanomotors has become one of the most common methods. Materials used to fabricate nanomotors for fluorescence imaging can be classified into two categories: those modified with organic molecules, such as fluorescent probes or quantum dots, and those constructed from autofluorescent materials.^1391^ Aggregation-induced-emission (AIE) is one of the methods of fabricating aggregation-induced-emission-based fluorescent nanomaterials. It is powerful enough to exhibit highly stable fluorescence in assembling nanomotors. Consequently, van Hest *et al*. presented nanomotors decorated with AIE motifs and driven by asymmetric Au nanoshells. Under NIR-TP irradiation, these nanomotors demonstrated powerful self-propelled directional motion and were applied in phototherapeutics.^1392^ Song *et al*. developed NIR-II light-driven dual plasmonic antimicrobial nanomotors. Under NIR-II light at 1064 nm wavelength, the AuNR-Cu_7_S_4_ interface formed an enhanced photothermal field. At the same time, it boosted ROS production, which allowed detection of the nanomotor’s penetration effect within the organism.^1393^

Organic fluorescent dyes can have disadvantages, such as photobleaching and fluorescence quenching, when they are used in fluorescence imaging. Additionally, the broad spectral ranges of different fluorescent dyes overlap with each other, which increases uncertainty when they are employed in the imaging a nanomotor. The development of semiconductor nanomaterials such as quantum dots have solved these challenges. Quantum dots show the advantages of high sensitivity and penetration due to their intrinsic luminescence and photocatalytic properties. Quantum dots could be used to fabricate and characterize nanomotors.^1394^ Dong *et al*. utilized quantum dots to construct the first nanomotor based on PbS. In their system, quantum dots can be actuated by NIR-I light and show excellent motion characteristics.^266^ Biosensors based on Förster Resonance Energy Transfer have also been applied to fabricate nanomotors.^137^ Graphene oxide is a multifunctional nanomaterial with fluorescence-quenching capabilities and possesses electron transfer ability and amphiphilicity.^1395^ Wang *et al*. constructed a biosensor by using graphene oxide nanomotors to target miRNA detection.^138^

Furthermore, autofluorescent materials were also used to construct micro/nanorobots. Intrinsic fluorescent materials of different wavelengths exist extensively in nature. Zhang *et al*. fabricated microrobots using *Spirulina* microalgae. *Spirulina* microalgae is a natural autofluorescent material. By modifying *Spirulina* microalgae and assembling Fe_3_O_4_ nanoparticles on its surface, not only do microrobots retain their intrinsic fluorescence but also become superparamagnetic. This allows microrobots to be utilized for *in vivo* fluorescence imaging, tracking, and real-time monitoring.^375^ Additionally, with intrinsic fluorescence and biocompatibility, chlorophyll, lignin, and photopolymer are widely employed in the fluorescence imaging of nanomotors.^1396^

Currently, fluorescence imaging is not only applied in medical imaging but is also integrated with other imaging modes, such as MRI, CT, and PET. The cooperation between fluorescence imaging and other imaging modes indicated the power of deep infiltration and imaging of nanomotors *in vivo* and will advance the development of precision medicine.^1397^

#### 6.4.7 Coherent Anti-Stokes Raman Spectroscopy Imaging

Coherent Anti-Stokes Raman Spectroscopy (CARS) imaging was introduced as an advanced technique to enhance very slow Raman scattering signal acquisition for imaging applications.^1398^ NIR lasers (a tunable pump laser and a Stokes laser) are employed to generate signals, providing deeper imaging based on the lower scattering of the longer wavelengths, and helping to minimize auto-fluorescence and prevent photo-bleaching.^1398^ CARS imaging is based on the interaction of multiphotons with the sample (the pump photon, the Stokes photon, and the probe photon). Their interaction generates a coherent anti-Stokes signal when the frequency difference between the pump and Stokes lasers matches the vibrational frequency of a particular molecular bond vibration.

Most existing techniques for tracking molecules within micro/nanorobots typically require the addition of labels to enhance imaging contrast.^1399,1400^ In contrast, CARS imaging offers significant advantages as a label-free, rapid, accurate, real-time, and high-resolution imaging technique with the ability to penetrate deeply into biological tissue.^1401^ In 2022, microrobots were characterized and imaged using CARS microscopy for the first time.^1402^ Self-propelled Mg-Au microrobots were loaded with furosemide and the CARS imaging technique confirmed the successful loading of the drug onto the microrobot’ outer surface. Due to the low solubility of furosemide in aqueous solutions, this method facilitates the long-term detection of microrobots with the CARS. Since then, CARS imaging to characterize micro/nanorobots has become an important tool in Boisen’s group. The same group extended their research on Mg-Au microrobots by loading them into microscale containers.^1403^ The CARS signal was generated with two NIR laser beams to confirm the presence of paracetamol on the surface of the microrobots. When bare Mg-Au microrobots were imaged with a CARS microscope, no CARS signal was observed. However, after loading paracetamol onto the Mg-Au microrobots, strong CARS signals were detected by CARS detectors, demonstrating that this technology has good potential as a detection tool in biomedical research. In the field of micro/nanorobots, CARS microscopy serves not only as an imaging technique but also as a highly attractive platform for activating and observing the movements of nanorobots.^1404^ Maric *et al*. constructed mesoporous SiO_2_-Au nanorobots designed to disrupt the integrity of PAO1 biofilm using NIR laser beams at a power scale in the mW range.

Although CARS imaging is a revolutionary technology, it is still not widespread in micro/nanorobot research. The major challenges include cost, as it requires specialized equipment with advanced microscopy facilities. Additionally, CARS microscopy necessitates complex and precise alignment of optical components, which can be further complicated by the movement of micro/nanomotor, hindering the acquisition of high-resolution images. Integration with high-speed imaging systems is crucial for effectively capturing the swift movements of micro/nanorobots and facilitating their application in clinical settings.

#### 6.4.8 Optical Coherence Tomography

Optical coherence tomography (OCT) is a noncontact imaging technique renowned for its high spatial resolution. Widely employed in ophthalmology, OCT plays a crucial role in diagnosing conditions such as glaucoma and retinal diseases by offering a quantitative assessment of retinal layers. In clinical applications, OCT has been utilized to observe a swarm of helical microrobots near the retina of bovine eyeballs.

While each imaging technique offers distinct advantages, it is essential to acknowledge their limitations. Consequently, the integration of multiple imaging methods, known as multimodal imaging, can yield more comprehensive and improved results. This approach enhances the accuracy of diagnosis and treatment, providing a more nuanced understanding of the subject under examination.

## 7 Applications

Micro/nanorobots hold significant potential for navigating in the body, operating in difficult-to-reach environments, and addressing specific health problems. Recent advancements in micro/nanorobots show considerable promise in overcoming challenges of the human body, making these tiny devices invaluable for biomedical applications (Figure 16).

**Figure 16:**
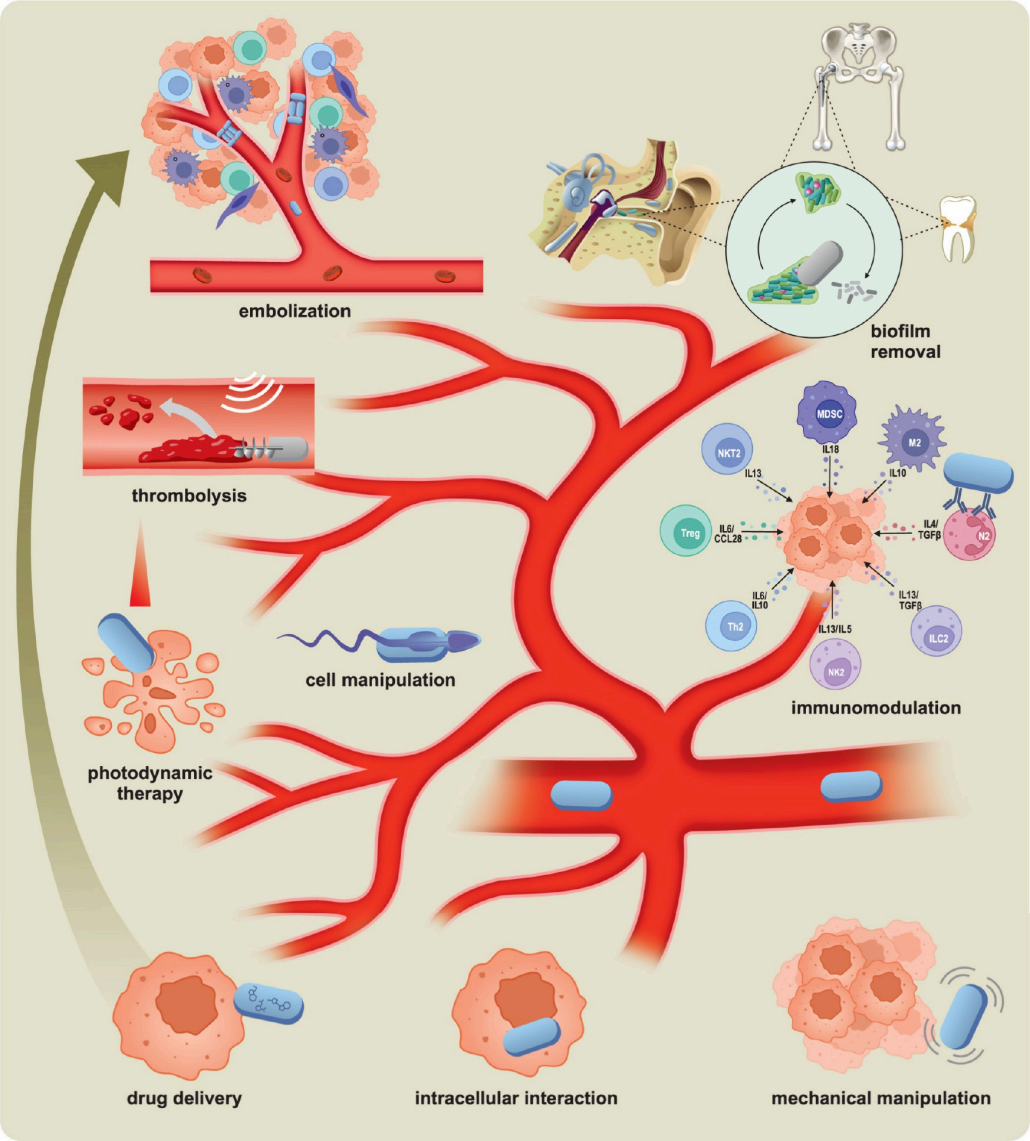
**Biomedical applications of micro/nanorobots across diverse fields.** Micro/nanorobots perform mechanical manipulation and enable intracellular interactions on the cellular level. They offer precise drug delivery and conduct photodynamic therapy to target specific cells. These robots manipulate single cells for diagnostics and regenerative medicine, dissolve blood clots for thrombolysis, and block blood supply to tumors via embolization. They modulate immune responses for treating diseases and effectively remove resistant biofilms on implants or tissues, demonstrating their potential to address complex medical challenges with precision and efficiency.

### 7.1 Micro/Nanorobot Applications in Biomedicine

#### 7.1.1 Drug and Cell Delivery

##### 7.1.1.1 Drug Delivery

An ideal drug carrier should possess four characteristics: (1) stabilizing the drug of interest, (2) transporting the drug to specific tissues, (3) controlling the release of the drug to maximize its time in the therapeutic window, and (4) degrading over clinically relevant timescales for elimination or clearance without adverse reactions. Discoveries in materials engineering, formulation science, and pharmacology have led to the development of new nanocarriers that realize most of these characteristics (*i.e.*, stabilization, controlled release, and degradation).^1405^ However, the delivery of drugs to specific tissues with minimal off-target accumulation is likely the most elusive characteristic to realize. Micro/nanorobots offer the potential to address this gap due to their ability to propel, change shape, and interact with diseased tissues in a controllable manner (Figure 16).^1406^

Drugs can be incorporated into micro/nanorobots through multiple means. This includes dissolving or embedding the drug directly within the bodies of the microrobots, embedding drug-loaded nanoparticles into the bodies of the microrobots, or conjugating drugs to the surfaces of the microrobots. How drugs are loaded into microrobots depends on the type of drug (*e.g.*, small molecule, nucleic acid, protein), its properties (*e.g.*, solubility, stability, charge density), and route of administration. Manufacturing processes that subject the drug to harsh conditions (*e.g.*, organic solvents, higher temperatures, and high-intensity UV light) limit the range of drugs that can be incorporated and may require alternative fabrication or drug-loading strategies.

How microrobots move should be coupled with their route of administration. Some routes of administration subject the carrier to conditions that necessitate a certain type of loading. For example, pH, salinity, and the presence of phagocytes are important considerations for drug formulation. Intravenous delivery provides microrobots rapid access to multiple organs. However, blood is replete with plasma proteins, cells, and other factors that adsorb to the surfaces of microrobots, which can affect their function and utility. Growing evidence suggests that intravenously administered nanoparticles fail to home to diseased tissues with specificity, regardless of their physical properties (*e.g.*, shape, surface coating). In the case of solid tumors, one retrospective analysis reported that a median of 0.7% of intravenously injected nanoparticles reach solid tumors.^1407^ For microrobots administered by inhalation, important considerations are size and the mechanism of propulsion. Dense or large particles (*e.g.*, >1 mm) generally fail to reach the distal airways of the lungs.^1408^ Further, mucus is a shear-thinning, viscoelastic, and heterogeneous fluid, which requires substantial thrust for propulsion. Finally, microrobots administered orally or by bladder installation are subjected to bulk fluid flows and shear forces that reduce residence times. Designing microrobots with adhesive, selective binding, or mechanical pinning capabilities can enhance their residence time and performance.^480^

As microrobots become more efficient at targeting diseased tissues, careful consideration must be given to their manufacturability. The capabilities of microrobots generally come with their complexity.^533,1409^ However, more complicated shapes, compositions, and designs typically require more advanced fabrication methods with limited scalability, weakening their clinical potential. Thus, high-throughput manufacture of biodegradable materials at the micro/nanoscale is critical to enable clinical translation.^1410^ In addressing these gaps, drug carriers can reach their full potential and enable new treatment paradigms for some of our most challenging diseases.

##### 7.1.1.2 Biohybrid Delivery

As an alternative to purely synthetic microrobots for drug delivery, biohybrid microrobots incorporating cells are emerging as candidates for next-generation delivery vehicles for cargo, such as bioactive drugs and contrast agents, to inflamed tissues. Due to their expression of key receptors (*e.g.*, integrin, chemokine), cells like monocytes, dendritic cells, neutrophils, macrophages, T cells, and mesenchymal stem cells are sensitive to inflammatory signals, endowing them the ability to extravasate at specific regions of the vasculature and crawl toward regions of inflammation. Recent pre-clinical studies show that combining nanoparticles with chemotactic cells can improve nanoparticles’ ability to target inflamed tissues. These strategies include internalizing nanocarriers within the cell (*e.g.*, through phagocytosis) or attaching nanocarriers to the outside of the cell.^1411,1412,1413^ Once the cell is localized to the site of interest, internalized particles can be probed or actuated remotely to detect accumulation or trigger drug release. Cell-based nanoparticle delivery approaches have been used for applications in cancer immunotherapy, medical imaging, traumatic brain injury, and the treatment of autoimmune diseases.^1414,1415,1416,1417,1418,1419^

Biohybrid microrobots are envisioned to overcome nanoparticle-mediated cancer therapy limitations through self-propulsion or an external guiding force. Broadly, two strategies have been implemented: one strategy uses single eukaryotic and prokaryotic cells as nanomedicine transporters, and the other uses magnetic field-based control strategies to guide localization and penetration of living cells (bacteria). Bashir and co-workers reported one of the first biohybrid microbots, using nanoparticle-carrying *Listeria monocytogenes* bacteria for gene delivery in 2007.^1420^ Irvine and co-workers introduced adjuvant nanomedicine-loaded T cell biohybrids for enhanced adoptive T cell cancer therapy in 2010.^1421^ Since these early examples, there has been burgeoning interest in biohybrids for cancer treatment, with a particular focus on bacterial biohybrids. Compared to eukaryotic cells, bacteria are smaller in size (can extravasate to interstitial spaces), have tropism to cancerous tissue (facilitate targeted delivery to primary tumor and metastases), present anti-tumor immunologic effects, have relatively smaller and well-known genomes (amenable genomic manipulation to enhance safety), have well-established synthetic biology toolboxes (enabling surface engineering for optimized nanocargo attachment or implementation of custom anticancer functions), and their intrinsic or engineered taxis (biased migration in gradient fields) can be harnessed for autonomous (*e.g.*, aerotaxis) or externally guided (*e.g.*, magnetic field^128,1422,1423^ or ultrasound^1424^) motion control. The rapid development of bacterial biohybrids for cancer therapy is further supported by the availability of probiotics, generally recognized as safe (GRAS) bacteria and attenuated pathogens (*e.g.*, vaccine strains) for safe human administration.^1425^

The relative ease of bacterial surface engineering has enabled the development of a diverse variety of biohybrid combinatorial cancer treatments, wherein the bacteria immunotherapeutic effects are synergistically combined with surface-conjugated chemotherapy,^128,144,1426^ photothermal therapy,^1427,1428^ radiation therapy,^1429^ and hyperthermia high-intensity focused ultrasound therapy.^1430^ Behkam and co-workers^144^ developed an autonomous drug delivery system by interfacing clinically safe tumor-selective *Salmonella typhimurium* VNP20009 with PLGA nanoparticles. These nanorobots could autonomously penetrate tumors through the intercellular spaces and enhanced retention without requiring any externally applied driving force or control input. Martel’s group reported synergistic bacteria-mediated chemotherapy by covalently bounding drug-containing nanoliposomes on *Magnetococcus marinus* strain MC-1. The peritumorally introduced biohybrid microrobots leveraged magneto–aerotactic behavior under an externally applied magnetic field to penetrate tumors. Up to 55% of the biohybrid population penetrated the hypoxic region of HCT116 colorectal xenografts.^128^ Wang’s group demonstrated the use of algae-based microrobots for drug delivery aimed at treating pneumonia and lung cancer, such algae-based active drug delivery systems show prolonged tissue retention and improved therapeutic outcomes.^131,1431^ Bacterial biohybrids have also been investigated for photothermal therapy involving surface-loaded photosensitizers, which upon autonomous or externally guided activation post-localization in tumor tissues, generate ROS, killing tumor cells. Gu *et al*.^1428^ leveraged the advantages of VNP20009 biotherapy and polydopamine-mediated photothermal therapy to enhance the efficacy of melanoma treatment. The approach eliminated tumors without relapses or metastasis with only one injection and laser irradiation. In a recent study, Yan *et al*. integrated an engineered, gas vacuole-containing, acoustic-responsive bacteria with doxorubicin coating to enable real-time imaging and hyperthermia high-intensity focused ultrasound-induced anti-tumor gene expression.^1430^ Upon exposure to ultrasound, the bacteria expressed IFN-γ to kill tumor cells and stimulate immune response. The doxorubicin coating further enhanced the anti-tumor efficacy of the biohybrid system in a triple-negative murine breast cancer model. Systemic administration of the drug-coated bacterial biohybrids resulted in a 30% higher survival rate than engineered acoustic-responsive bacteria.

To realize the potential of cells as living microrobots, several clinical factors must be considered. This includes how nanoparticles affect the phenotypes and functions of the cells that carry them. Cells loaded or coated with nanoparticles change their expression of integrand and chemokine receptors,^1432,1433^ potentially altering their ability to infiltrate sites of interest. Additionally, the act of carrying nanocarriers presents an added barrier that can reduce the ability of cells to cross physiological (*e.g.*, endothelial) barriers. Thus, not only should future work investigate the quantitative efficiency of cells to improve the delivery of nanoparticles to diseased tissues, but also how nanoparticles affect the physiology of cells to better understand and exploit the cooperative interactions between these entities.

Certain routes of administration present barriers that render cell localization challenging. Examples include (i) inhalation due to mucosal barriers, salinity, and variable levels of moisture, (ii) oral administration due to variable levels of pH and shear forces, and (iii) fertilization. In these scenarios, microrobots can serve as vehicles to transport cells.^1434^ Recent work has shown that microrobots can effectively move cells by rotating magnetic fields or optical trapping.^511,910,1435,1436^ These microrobots possess the ability to transport cells through biological barriers such as mucosal barriers, tissue barriers, and barriers associated with high shear flows.^135,474^ When using living cells in microrobots, however, several considerations must be made. A leading consideration is the cell source (*i.e.*, from the patient (autologous) *vs*. a donor (allogeneic)). While autologous cells avoid risks associated with host rejection, they require time, resources, and cost for modification and deployment, which can preclude their use in certain clinical scenarios. While allogeneic approaches simplify deployment and enable the possibility of banking cells and administering under shorter timescales, these cells risk host rejection or adverse side reactions. One solution is to deploy cellular microrobots immediately after chemotherapy or radiation when the immune system is in a suppressed state. Finally, immune-evasive cells like mesenchymal stem cells are promising candidates for microrobots due to their ability to evade rejection and maintain chemotactic mobility.^1437^ Further studies on the type and source of adoptive cell transfers will accelerate the development and translation of these systems.

##### 7.1.1.3 Sperm-Driven Microrobots for Drug Delivery

Sperm-driven microrobots were also employed for drug delivery, contributing to the hybrid approach of developed micro/nanorobots for biomedical applications. For example, a 3D-designed microstructure was fabricated to capture motile, drug-loaded sperm cells and release them by a mechanical trigger on its four-armed front end.^1438^ Targeted local biochemical manipulation of spermatozoa was achieved by template-based gelatin microtubes.^1439^ The biodegradable gelatin micro-cartridges showed a pH response and this property was exploited for pH-triggered drug delivery. Spermatozoa must travel through fluids with a wide range of pH (from about pH 4–8) from the seminal fluid through the vagina, cervix, and uterus to the fallopian tubes. The pH response of gelatin can be used to load drugs at pH 5 and release them when pH 8 is reached. Hence, an approach was undertaken to demonstrate that sperm cells can be capacitated *in vitro* upon demand by the release of heparin from the microstructure. Heparin is a sperm-activating agent known to induce capacitation, a crucial sperm maturation step before fertilization. The goal of this approach was to demonstrate that sperm capacitation can be achieved by *in situ* pH-triggered release of heparin from the sperm-capturing structures. Heparin not only induces capacitation but also has a positive effect on sperm motion. The results showed not only that the velocity of the heparin-loaded spermbots was increased from 14 μm s^–1^ to 20 μm s^–1^, but also the capacitation amount was elevated from 22% to 40% of the cells at pH 8.^1439^ Additionally, in the case of 4D multimaterial sperm carriers, a thermo-responsive hydrogel valve was used for the local release of heparin. These microstructures are also adorned with polymersomes sensitive to slight pH changes and loaded with hyaluronidase, an enzyme that interacts with the hyaluronic acid in the extracellular matrix of the cumulus complex surrounding the oocyte. This interaction loosens the cumulus complex, facilitating the sperm’s journey toward the oocyte. This approach is particularly crucial in cases of low sperm count, where the cooperation of multiple sperm to navigate various biological barriers toward the oocyte is less effective.^1440^

The generation of ROS, such as hydroxyl radicals, induces oxidative damage. This is one of the main causes of sperm damage during *in vitro* handling and reduces the lifetime of spermatozoa *in vivo* and *in vitro*. The antioxidant effect of gelatin microtubes on sperm was demonstrated by an over 90% reduction in radicals.^1439^ Hence, gelatin-based microrobots have been proven to serve as protective agents against oxidative stress. A similar effect was observed with magnetotactic sperm, attributed not to gelatin but to polystyrene-based particles. The hypothesis posits that these particles prompt the formation of a protein corona due to the presence of bovine serum albumin in the sperm medium. Bovine serum albumin is known to sequester reactive species, thereby enhancing the antioxidant properties of the particles. This is evident in the viability results, where after 6 and 24 hours, the viability of magnetotactic sperm is nearly twice as high compared to that of free-swimming sperm. Similarly, there was a notable enhanced motility compared to the control, with levels exceeding three times that of free-swimming sperm after 6 and 24 hours.^1045^

The actuation of nonmotile sperm is of interest in the case of low or no sperm motility. When spermatozoa cannot move fast enough or show no motility, microrobots can serve as transportation devices. Spiral microstructures that rotate in an external magnetic field and perform a screw-like motion, can be used to transport single spermatozoa.^910^ Rotating magnetic fields are applied to capture the cells, drive them to the target location, and release them by backward rotating motion. Another approach is to use magnetic particles and attach them directly to the membrane of spermatozoa by electrostatic interaction. These particle-covered spermatozoa can then be actuated by rotating or oscillating magnetic fields, which results in a wave-like motion similar to live sperm.^1441^ This has been demonstrated thus far with dead bovine sperm, which were then utilized as sperm-templated flexible microrobots, and with live sperm for motile sperm separation and guidance.^1442^ Bovine serum albumin-hyaluronic acid microflakes have been utilized to transport multiple immotile sperm cells. However, upon enzymatic degradation, the sperm cells remain in the vicinity of the oocyte without reaching the *zona pellucida*. Due to their lack of motility, they are unable to penetrate further and fuse with the oocyte. As a result, alternative strategies need to be explored.^1443^

##### 7.1.1.4 Active Therapy Based on the Byproducts of Micro/Nanorobots

In addition to utilizing micro/nanorobots as active drug carriers, the direct utilization of *in situ* byproducts formed in response to the microenvironment or external stimuli during the motion process emerges as an appealing solution for practical. Based on their characteristics, the byproducts of micro/nanorobots can be sorted into chemical byproducts and physical byproducts. Chemical byproducts are routinely the result of spontaneous chemical reactions between micro/nanorobots and substrates readily available in the microenvironment. Tu and Peng’s group utilized biocompatible and biodegradable Mg-based Janus microrobots for the active H_2_ therapy of acute ischemic stroke.^1444^ Mg-based Janus microrobots are powered by recoil forces generated during the expulsion of H_2_ bubbles produced by Mg–H_2_O reactions. The bubbles are released through a small opening to conserve momentum effectively. Apart from providing a driving power source, H_2_ also acted as a therapeutic component to regulate the inflammatory microenvironment, which was systematically substantiated in a classical middle cerebral artery occlusion rat model. NO-driven nanorobots, consisting of medically used fluorescent hyperbranched polyamide and L-arginine, were also developed by Shen and Mao’s group.^1445^ By catalyzing the decomposition of L-arginine, the produced nitric oxide was employed not only as a continuous thrust but also as a therapeutic component to fight cancer. Moreover, Tu, Peng, and their co-workers demonstrated a urease-based nanorobot for the active therapy of bladder tumors.^1446^ By decomposing endogenous urea into CO_2_ and NH_3_, urease-actuated nanorobots allowed enhanced diffusion *via* the generation of byproduct gradients while realizing potential anti-tumor effects derived from ammonia toxicity.

Physical byproducts generally refer to physical phenomena that occur during the motion process. He’s group developed Au nanoshell-functionalized polymer multilayer rocket in the shape of a conical cylinder.^1447^ NIR laser irradiation to the Au nanoshells with strong plasma resonance absorption in the NIR region permitted the formation of a local thermal gradient surrounding the robot, which in turn gave rise to a thermophoretic force to propel the rocket forward. Upon being attached to HeLa cells, the photothermal toxicity of the rocket contributed to the apoptosis of the targeted cell *in vitro*. Peng’s group designed a light-powered robot based on an asymmetric TiO_2_-Au nanowire.^1448^ Upon exposure to UV irradiation with low intensity, the motor could be steered in pure water based on photochemical water splitting, which led to a self-generated local electric field. Once in contact with the targeted neuronal retinal ganglion cells, the inherent photoelectricity produced during the motion process would serve as a stimulus to trigger cell activation. By directly making full use of the byproducts generated *in situ* from micro/nanorobots for active therapy, adverse reactions such as toxic side effects can be remarkably avoided, turning waste into treasure.

#### 7.1.2 Micro/Nanosurgery

Untethered micro/nanorobotic tools, including nanodrillers,^196^ microgrippers, and microbullets, offer unique capabilities for minimally invasive surgery.^1449^ With dimensions compatible with those of small biological entities, they present significant advantages for high-precision, minimally invasive procedures (Figure 16). Powered by diverse energy sources, these micro/nanorobots, equipped with nanoscale surgical components, can directly penetrate and retrieve cellular tissues, enabling unparalleled precision in surgery.^1450^ In contrast to their larger robotic counterparts, these miniature robots can navigate through the body’s narrowest capillaries and conduct procedures at the cellular level. Untethered microgrippers signify a significant stride toward developing autonomous robotic tools for microsurgery.^1451^ These mobile microgrippers can capture and retrieve tissues and cells from challenging and inaccessible locations.^929^ Typically, conventional microgrippers are tethered and respond to mechanical or electrical signals generated by control systems through external connections such as wires and tubes. This tethering property limits their miniaturization and maneuverability. Like their larger tethered counterparts, untethered microgrippers commonly operate by opening and closing.

Magnetically actuated micro/nanorobots have demonstrated significant promise for minimally invasive *in vivo* surgical operations. Magnetic fields, with their ability to penetrate thick biological tissues, make these microrobots particularly effective for such procedures.^129,1452^ Recently, a mechanical nanosurgery approach using swarms of magnetic carbon nanotubes as “nanoscalpels” was reported.^1452^ The swarms were functionalized with antibodies to improve tumor cell recognition and extend their retention inside tumor cells. Upon actuation, the swarms applied mechanical torque to tumor cells, disturbed the metabolism of intracellular organelles such as mitochondria, and mechanically induced tumor cell apoptosis. This nanosurgery approach was validated in mice with brain tumor glioblastoma multiforme. Its efficacy was also proven to extend the survival of chemo-resistant mice.

Recent advancements in ultrasound actuation have led to the development of powerful microrobots with remarkable tissue penetration properties. Kagan *et al*. demonstrated ultrasound-triggered, high-velocity propulsion resembling a “bullet-like” motion, achieved through the rapid vaporization of biocompatible fuel, specifically perfluorocarbon.^58^ These conically shaped tubular microbullets, housing the fuel source, exhibit ultra-fast movement, reaching speeds exceeding 6 m s^–1^ (equivalent to 160,000 body lengths per second) when subjected to external ultrasound stimulation. This remarkable speed equips them with ample thrust for achieving deep tissue penetration, ablation, and destruction.

The miniaturized structure endows micro/nanorobots the potential to perform microsurgery and bio-signal sensing at the single-cell level with subcellular precision.^1449,1453,1454^ However, such a task is hindered by the vigorous Brownian motion at micro/nanoscale. Therefore, feedback control is often required to actively counter the Brownian motion for high-precision and steady control.^885,1455^ Li *et al*. reported the unmatched performance of the 3D electrokinetic tweezers. This recent invention can control a nanowire’s position and 3D orientation at a precision of 20 nm and 0.5 degrees, respectively, in a solution under a standard microscope. They successfully maneuvered a nanowire to probe a single bacterium at multi-designated locations and detected metabolite release from a single cell.^1456^

Another proposed microrobotic approach for surgery involves the use of smart microcatheters, which enables the integration of microactuators based on electroactive polymers. The inclusion of a gripper in the distal part of the catheter shows promise for capturing and releasing particles as small as 100 micrometers, akin in size to oocytes and early embryos, suggesting potential applications in minimally invasive oocyte retrieval and embryo transfer procedures. These microcatheters also feature sensors capable of measuring mechanical and/or biochemical properties, all powered by an external power supply. Additionally, their hollow structure facilitates drug delivery while integrated anisotropic magnetoresistance sensors allow for long-distance magnetic-phase-encoded tracking of the structure. This functionality proves particularly advantageous in organs like the skull, where ultrasound imaging is impractical. Under laboratory conditions, these microcatheters demonstrate a high tracking resolution of 72 micrometers. Compared to magnetic sensors, anisotropic magnetoresistance sensors offer greater compactness and eliminate the need for additional packaging.^1457^

#### 7.1.3 Embolization and Thrombolysis

Embolization is a medical procedure that blocks blood vessels to control bleeding or cut off the blood supply to a tumor. With their active locomotion capabilities, micro/nanorobots can enable embolization at targeted locations. For instance, magnetic microparticle swarms have been employed for selective embolization.^786^ The magnetic particles, coated with thrombin to induce fibrinolysis, were actuated by dynamic magnetic fields to selectively embolize blood vessels within a targeted region (Figure 16). *In vivo* trials in porcine kidneys demonstrated that the microrobotic swarms were able to achieve selective embolization, providing a potential solution to mitigate complications associated with existing passive, nonselective embolization techniques. For precise aneurysm embolization, magnetic microfiberbots^1458^ and pH-responsive microgel swarms^826^ have also been developed. The magnetic microfiberbots were able to propel through blood vessels and aggregate to embolize an aneurysm or block blood flow in a femoral artery under magnetic field actuation. The microgel swarms were similarly magnetically driven and aneurysm embolization was triggered by the injection of an acidic buffer solution, which induced self-healing of the microgels to form a single entity.

Conversely, micro/nanorobots can be used to facilitate thrombolysis, *i.e.*, to degrade the blood clots that block a blood vessel. Micro/nanorobots can be controlled to exert mechanical forces to remove blood clots,^1459,1460,1461^ generate convective flows to enhance the diffusion of tPA molecules (*i.e.*, drugs for blood clot lysis),^1462,1463^ and induce hyperthermia^1213,1464^ to accelerate thrombolysis. In recent work, tPA-anchored magnetic nanorobots were used for thrombolysis at submillimeter segments in rats and rabbits.^1459^ The combination of mechanical interaction and chemical lysis reduced the required tPA dose by approximately 42-fold while enhancing the thrombolysis rate by approximately 20 times, providing an efficacious method for blood clot dissolution in hard-to-reach vessels.

#### 7.1.4 Biofilm Removal

Biofilms—resilient microbial communities embedded in an extracellular matrix—are a major challenge in treating persistent infections, particularly those involving medical devices, chronic wounds, and respiratory tract infections.^1465,1466^ Their resistance to conventional antibiotics is due to both the protective matrix and the altered metabolic states of the microorganisms.^1467^ Micro/nanorobots offer a promising solution with their ability to navigate and penetrate biofilm structures by disrupting the extracellular matrix and delivering targeted treatment, potentially revolutionizing the approach to combating biofilm-associated infections (Figure 16).^1468,1469^

##### 7.1.4.1 Dermatologic Infections

Biofilm-related skin infections are often chronic and can be challenging to treat.^1470^ Common bacteria associated with biofilm formation on the skin include *Staphylococcus aureus* and *Pseudomonas aeruginosa*. Fenton reaction and nitric oxide generation are the most commonly applied mechanisms for micro/nanorobots to tackle superficial tissue infections.^1471,1472^ In 2022, Liu *et al*. developed L-arginine-coated nanorobots composed of dendritic mesoporous silica nanoparticles and Au nanoparticles, which effectively eradicated biofilms and significantly reduced *Staphylococcus aureus* infection in a wound model by generating nitric oxide through a cascade reaction.^1473^ Similarly, Ma *et al*. designed pH-responsive nanorobots that, within the acidic biofilm microenvironment, generated reactive nitrogen species to efficiently disrupt bacterial structures and self-propel through biofilms. This approach achieved a 12-fold improvement in diffusion efficiency and over 99% bacterial eradication, substantially accelerating wound healing.^1368^ Light-triggered microrobots, such as NIR-propelled MOF-based structures, offered a novel approach to biofilm eradication. These nanomotors, asymmetrically coated with Au and DNase I, demonstrated self-directed movement effectively eliminated bacterial infections and promoted wound healing *in vivo* under light and ultrasound irradiation.^1474^ Another example of light-driven photosensitive microrobots with self-degradable capabilities holds promising improvements in antibacterial activity against *E. coli* and *S. aureus* through a combined action mechanism. These microrobots, made of Ag_3_PO_4_, can release Ag and generate ROS, leading 93% reduction in biofilm viability and highlighting the importance of their physicochemical properties in enhancing antibacterial efficacy.^1475^ Koo *et al*. developed magnetically actuated microrobots with nanozymes for targeted fungal pathogen eradication. Tested on a model of the oral mucosa, these microrobots demonstrated precise delivery and effective elimination of *Candida albicans*, significantly reducing fungal viability.^1476^

##### 7.1.4.2 Implant Infections

Biofilm infections on medical implants, caused by bacteria adherence and colonization on implant surfaces, are a common and challenging issue.^1477^ For instance, biofilms on dental implants can lead to gum inflammation, implant failure, and costly treatments.^1478^ Pumera’s group developed hybrid microrobots using photoactive BiVO_4_ microparticles and Fe_3_O_4_ nanoparticles together with micellar PEI polymer for oral biofilm removal. The rotating magnetic field drives the Fe_3_O_4_ component, ensuring even distribution of oxidative species from BiVO_4_, which helps break down oral biofilm on dental implants by approximately 50–90%.^1479^ Additionally, another design of the tubular black-TiO_2_/Ag microrobots with extended light absorption wavelengths, which propel under UV and blue light, effectively reduced bacterial biofilm on Ti miniplate implants by about 40% within 30 minutes.^1480^ Considering the deep penetration of light sources for practical applications, Qu’s group developed an NIR-driven mesoporous silica nanostructure with half-shell Au functionalization and vancomycin loading, enabling effective photothermal and antibiotic therapies.^1481^ These nanorobots demonstrated remarkable mobility and complete biofilm penetration within minutes, confirming nearly total bacterial elimination. In a mouse implant-related periprosthetic infection model, the nanorobots led to a swift temperature increase and a rapid reduction in wound size with minimal damage to surrounding tissues. Zhang *et al*. developed metal oxide-based hybrid microrobots that can efficiently produce H_2_O_2_ to eliminate bacterial biofilms in tympanostomy tubes. Their efficacy was successfully demonstrated *ex vivo* using an endoscope.^1482^ In addition to tympanostomy tubes, hard-to-reach areas like biliary stents can also be treated using magnetic microswarms. These microswarms, made of natural urchin-like sunflower pollens integrated with magnetic liquid metal droplets, effectively eradicate biofilm in biliary stents by using their sharp edges and excellent mechanical disruption of the EPS matrix under magnetic field control.^1483^

##### 7.1.4.3 Tooth Infections

The warm, moist, and nutrient-rich oral environment promotes the growth of microorganisms and the formation of pathogenic biofilms on tooth surfaces, contributing to diseases like dental caries and periodontitis. Advances in micro/nanorobots offer promising solutions for targeting and disrupting these biofilms, addressing limitations due to the intricate topographies and inaccessible locations.^1469^ Pumera’s group proposed chemically powered tubular TiO_2_/Pt microrobots for efficient disruption of dental biofilm by ROS generation.^1484^ However, existing methods face challenges in effectively removing strongly adherent biostructures from intricate surfaces. Oh *et al*. developed magnetic field-directed surface topography-adaptive robotic superstructures that adjust their shape, length, and stiffness to precisely remove biofilms and perform diagnostic sampling on *ex vivo* human teeth, achieving complete biofilm eradication.^1485^ Similarly, Hwang *et al*. built 3D-molded catalytic antimicrobial robots propelled by a corkscrew-like motion at 5 mm s^–1^ under a rotating magnetic field, effectively clearing biofilm clogs and navigating tooth canals, reaching the end within 17.7 seconds.^1486^

#### 7.1.5 Microrobotic Probes for Cell and Tissue Mechanobiology

Advancements in fabrication and wireless actuation techniques within the field of micro/nanorobotics have led to the development of an innovative tool for mechanobiology research: microrobotic probes. Microrobotic probes are untethered biocompatible cell-sized machines that can generate forces to interact with living cells either *via* direct physical contact or through the microenvironment, particularly the extracellular matrix. The probe can mimic certain morphological, structural, and mechanical properties of prokaryotic or eukaryotic cells depending on the research question.^1487^ Robotics technology allows continuous monitoring and closed-loop actuation of the probe, enabling spatiotemporally resolved dynamic and adaptive stimulation of cellular processes as well as *in situ* characterization of biomechanical properties (Figure 16).

Magnetic actuation is the primary choice for microrobotic probes because the type of magnetic fields that are used to actuate these probes (*i.e.*, low strength and low frequency) do not have any reported impact on cellular physiology. In addition, several programmable magnetic field generators have been introduced that can be seamlessly interfaced with optical microscopes for dexterous manipulation of the probes.^1488^ At the lowest size scale, a multipole magnetic manipulation system has been developed to navigate submicrometer magnetic beads inside a living cell to measure the mechanical properties of the nucleus.^385,1489^ At the cellular scale, magnetic probes in the form of rod-shaped bacteria have been maneuvered around macrophages and programmed to resist phagocytosis following various biomimetic strategies to study the mechanobiology of immune responses.^1490^ Two-photon polymerization allows fine-tuning over the shape of microbe-sized probes, providing exciting opportunities to study the role of cellular geometry and topography in the mechanobiology of immune clearance.^1491^ Magnetic microswimmers and their appendages, such as flagella, can also be fabricated from soft stimuli-responsive hydrogels,^1492^ bringing mechanics and fluid–structure interactions under the spotlight. At the multicellular scale, magnetic probes have been deployed inside microtissues to quantify the stiffness of tumor colonies and developing tissues in embryos.^1493,1494^ Robotically actuated magnetic probes have also been integrated into natural and synthetic extracellular matrices to study the effect of dynamic mechanical stimulation on the multicellular organization of connective, muscle, and cancer tissues.^1495,1496,1497^

Optical actuation is an alternative solution for the manipulation of microrobotic probes, providing superior spatial resolution compared to magnetic actuation. A holographic optical tweezer is capable of individually trapping and independently manipulating multiple probes around living cells in order to study cell migration^1498,1499^ or to mechanically probe cells inside living embryos.^1500^ Optical power can also be harnessed to generate compressive forces by synthesizing thermo-responsive hydrogel nanocomposites, specifically using Au nanorods.^1501^ Photothermally triggered actuation allows highly localized, directional, and reversible probe deformation, with control at scale ranging from individual receptors to cell clusters, and response time on the order of milliseconds.^1502,1503,1504,1505,1506^ Optical control over both translation and deformation could be realized to create microrobotic probes that mimic crawling contractile mammalian cells or bacteria with contractile pili.^1507^

#### 7.1.6 Biomedical Applications *In Vivo*

*In vivo* application of micro/nanorobots presents a variety of challenges and characteristics due to the diverse environments they encounter within the body. These applications can be broadly categorized into several primary regions: the gastrointestinal (GI) tract, vascular system, the urogenital tract, the respiratory tract, joint cavities, solid tissues, and cerebrospinal fluid (CSF) systems. Each of these environments requires tailored microrobot designs to optimize their performance and address the specific characteristics and demands of the target region (Table 1). For example, the GI tract, blood vessels, urogenital tract, and joint cavities primarily possess a liquid or liquid–solid interface environment characterized by the presence of bodily fluids or blood with inherent viscosity and fluid dynamics. In contrast, the respiratory tract typically features a gaseous or gas–liquid interface environment. Understanding these unique conditions is crucial for optimizing the design and operation of microrobots in order to ensure their efficacy and precision in performing therapeutic and diagnostic tasks within these complex biological systems.

**Table 1:** **Comparison of Conventional Drug Delivery Methods *vs*. Microrobot-Based Delivery Approaches** ^a^

Body region	Conventional delivery methods	Challenges and limitations	Microrobot design and mechanisms	Applications	Advantages of microrobot delivery
**Gastrointestinal tract**	-Administration through oral routes (e.g., tablets or capsules)	-Reduced bioavailability caused by enzymatic breakdown and pH fluctuations	-Magnetic robots	-Targeted drug delivery to specific gastrointestinal regions	-Noninvasive delivery to minimize patient discomfort
	-Delivery via endoscopic methods	-Restricted precision in targeting	-Ultrasound-driven robots	-Tissue sampling and diagnostic applications	-Localized drug delivery at high concentrations
	-Systemic distribution through the bloodstream	- Invasive endoscopic procedures	-Autonomous robots		-Stability in dynamic conditions (e.g., peristalsis, gastric acid)
**Vascular system**	-Systemic administration through intravenous injection	-Nonspecific drug distribution leading to systemic side effects	-Magnetic robots	-Targeted delivery to specific organs	- Precise delivery to prevent side effects
	-Localized delivery utilizing catheter-based methods	-Invasive insertion of catheters	-Chemically driven robots	-Treatment of thrombolysis or embolism	Reliable navigation through complex blood flow
		-Targeting disrupted by hemodynamic forces			-Noninvasive procedures to lower surgical risks
**Urogenital system**	-Systemic administration through oral or intravenous routes	-Reduced retention time caused by urinary flow	- Magnetic robots	-Targeted drug delivery to bladder, kidneys, or reproductive organs	-Low-risk delivery methods to minimize infection
	-Targeted delivery using catheters or stents	-Low targeting precision for specific tissues	-Ultrasound-driven robots	-Localized treatment of urogenital diseases	-Prolonged placement within urinary flow
		-Invasive catheter procedures with a risk of infection			-Resilience in high-flow conditions
**Respiratory tract**	-Inhalation-based treatments	-Difficulty in penetrating deep lung regions	-Autonomous robots	-Delivery of drugs to deep lung regions	-Efficient penetration through mucus barriers
	-Systemic administration of drugs for pulmonary diseases	-Uneven distribution of drugs	-Magnetic robots	-Precise therapy for pulmonary diseases	-Adaptability to airflow variations for accurate navigation
		-Rapid drug removal by mucociliary clearance			-Noninvasive techniques to enhance patient comfort
**Solid tissues**	-Systemic administration of medications	-Challenges in penetrating dense tissues	-Magnetic robots	-Targeted drug delivery to solid tissues	-Deep tissue penetration with accurate targeting
	-Targeted delivery through direct injection or implanted devices	-Nonspecific targeting potentially harming healthy tissues	-Chemically driven robots	Tissue repair and regeneration	-Optimized drug dosage for enhanced therapeutic outcomes
		-Restricted drug diffusion within tissues			-Reliable movement through dense tissues
**Joint cavities**	-Arthroscopic procedures for precise drug administration	-Invasive procedures with potential complications	-Magnetic robots	-Drug delivery to specific intra-articular regions	-Less invasive delivery to reduce post-procedural complications
	-Intra-articular delivery for the treatment of arthritis or joint discomfort	-Rapid clearance of drugs from joint cavities		-Cartilage repair and tissue regeneration	-Precise access to cartilage fissures for improved effectiveness
		-Low precision in reaching cartilage fissures		-Extended retention at injury sites	-Accurate drug delivery to minimize wastage
**Cerebrospinal fluid system**	-Drug administration via lumbar puncture	-Blood-brain barrier restricts drug delivery to the CNS	- Magnetic robots	-Targeted drug delivery to specific regions of the CNS	-Targeted delivery to overcome the blood-brain barrier
	-Intravenous injection	-Low diffusion efficiency within the CSF	- Autonomous robots	-Therapy for neurological disorders	-Controlled drug diffusion for better efficiency
		-Challenges in targeting specific brain regions			-Low-risk minimally invasive approaches to enhance safety

a The comparison is across various body regions, highlighting challenges, microrobot design mechanisms, applications, and advantages.

##### 7.1.6.1 Gastrointestinal Tract

Micro/nanorobots used for GI delivery are typically actuated by chemical fuels or magnetic fields. In the GI tract, microrobots must contend with the corrosive effects of digestive fluids and the mechanical resistance posed by the plicae. To overcome these challenges, microrobots are designed to either passively avoid or actively harness the corrosive nature of these digestive fluids to power their controlled movement. For example, in the stomach, microrobots composed of materials like Mg or Zn react with gastric acid to produce H_2_ bubbles that propel them, allowing efficient navigation through the environment. This capability enhances drug delivery to specific targets such as the gastric mucosa for treating conditions like *Helicobacter pylori* infection.^126,127,901,1089,1508,1509,1510^ An example of this is Zn-based microrobots developed by Gao *et al.*,^126^ which dissolves in gastric acid, releasing its therapeutic payload as it self-destructs without leaving toxic residues. In the intestine, similar microrobots are equipped with enteric coatings that protect them from the stomach’s acidic conditions, activating instead in the more neutral intestinal fluids.^901^ These microrobots are particularly effective for delivering drugs over extended distances within the intestine. Subsequently, the development of microrobots has significantly evolved, advancing from simple orally administered solutions to more sophisticated forms such as capsule-enclosed microrobots and tablet formulations.^133,134^ These advancements offer enhanced stability, controlled release, and improved patient compliance.

However, the GI environment also presents challenges such as peristalsis and complex fluid dynamics, which can complicate the precise delivery of microrobots. Researchers have begun to exploit the directional nature of peristalsis to transport microrobots over long distances while employing external fields to achieve high-dose accumulation at localized disease sites. To ensure prolonged and effective drug release at specific locations within the GI tract, it is crucial to anchor the microrobots once they reach their target. Current theragnostic GI residence strategies include freestanding systems, anchored systems, and exogenous control systems.^1511^ Freestanding systems utilize mechanisms such as geometric deformation, floating, and self-propulsion to maintain prolonged residence within the GI tract. For instance, self-propelling microrobots have demonstrated the ability to achieve precise positioning and extended retention times through chemical or biohybrid propulsion mechanisms. Anchored systems, in contrast, use methods like muco-adhesion, such as polydopamine coatings, and microstructures (like microneedles) to secure devices in place for extended periods.^1512^ Exogenous control systems employ external energy fields such as magnetic, electric, or photoacoustic controls to remotely manipulate and navigate devices within the GI tract.^1140^ Recent efforts by Wang’s group demonstrated robotic pills for improved drug bioavailability by embedding Mg-based Janus microstirrers within conventional oral pharmaceutical pills.^134^ The released microstirrers have been shown useful to enhance the bioavailability of aspirin and L-dopa in a porcine model. Oral capsules loaded with macrophage-cell membrane coated-algae biohybrids were shown useful for efficient “on-the-fly” removal of inflammatory cytokines for treating IBD.^1513^ These advancements indicate the considerable potential of microrobots for controlled drug delivery and targeted diagnostic interventions within the complex and dynamic environment of the GI tract.

##### 7.1.6.2 Vascular System

In the vascular system, microrobots must navigate a highly challenging environment characterized by high viscosity and rapid blood flow. The presence of red blood cells, white blood cells, and other suspended particles in the bloodstream poses a significant obstacle to the movement of these microrobots while the elasticity of vessel walls and the pulsatile nature of blood flow further complicate their stability and control precision. Effective navigation and precise localization in such a complex environment require microrobots to possess advanced maneuverability and adaptability to hemodynamic forces. For instance, in cases of downstream flow, researchers can exploit blood circulation to transport microrobots over long distances, using external fields to concentrate them at targeted disease sites for high-dose drug delivery.^1041^ However, the upstream movement of microrobots is particularly challenging due to the high blood flow velocities within the body, which can range from approximately 30 to 100 cm s^–1^ depending on the location. To overcome these challenges, a promising strategy involves mimicking the rolling of neutrophils along the vessel walls,^491^ allowing individual microrobots or swarms of microrobots to move along the vascular walls, thereby minimizing the impact of high flow rates on their control and movement.^496^ The vascular system’s extensive reach, which encompasses nearly all organs in the body, makes it a vital conduit for drug delivery to various diseased tissues. Consequently, microrobots have demonstrated considerable potential in medical applications, such as the dissolution of intravascular thrombi,^1514,1515^ embolization of aneurysms,^826,1458,1516,1517^ and treatment of glioblastoma *via* the circulatory system.^1041^ By leveraging the vascular network, microrobots can achieve targeted therapeutic interventions with a high degree of precision, opening new avenues for minimally invasive treatments across a wide range of vascular-related conditions. Additionally, due to their fascinating taxis abilities,^1518^ spermatozoa can swim against fluid flow.^1519^ This was explored in a study to evaluate the sperm’s ability to swim against the bloodstream in an on-chip setting.^909^ Tubular microcaps were designed and fabricated with two-photon lithography with a diameter of 13 μm and coated with magnetic material to allow remote guidance of the sperm-hybrid motors. Bovine spermatozoa were captured inside the microcaps and their swimming performance was tested in a microfluidic chip with fluid flow applied like physiological blood flow rates. The captured sperm cells showed the ability to swim against fluid flow of diluted blood and, further, to deliver heparin, an anti-coagulation agent, by liposomes attached to the microcaps. This approach shows potential in using spermatozoa as propulsion and delivery microrobots for applications in blood vessels.

##### 7.1.6.3 Urogenital Tract

The fluid environment of the urogenital tract typically presents various electrolytes and organic substances. Compared to the GI and vascular systems, the flow rate in the urogenital tract is generally lower, but the composition and viscosity of the fluid can vary significantly. Microrobots operating in this environment must adapt to these dynamic changes and achieve effective movement with relatively low energy input. Recent *in vivo* studies have demonstrated how microrobots can achieve precise drug delivery and effective treatment within this electrolyte-rich environment. Sánchez *et al*.^37,229^ demonstrated how self-propelled microrobots can utilize the electrolytes and organic substances present in the urinary tract as energy sources. These microrobots are designed to operate autonomously without relying on external energy sources and thereby achieve effective drug delivery and disease treatment within the urogenital system. The self-propulsion mechanism allows the microrobots to better adapt to environmental changes within the urinary tract, enhancing therapeutic efficiency and reducing the need for external intervention. In addition to self-propelled microrobots, Tang and co-workers^1520^ developed a magnetically driven Janus cell robot loaded with oncolytic adenovirus for targeted virotherapy of bladder cancer. These cell robots, equipped with an asymmetric coating of magnetic nanoparticles, achieve precise movement and directional navigation within the bladder. The research demonstrates that these cell robots not only penetrate deeply into bladder cancer tissues but also enhance the efficacy of cancer treatment through virus replication and transmission, significantly improving therapeutic outcomes. Such innovations underscore the versatility and effectiveness of microrobots in navigating the complex and variable environments of the urogenital tract.

##### 7.1.6.4 Female Reproductive Tract

Microrobots are being developed for two main areas of applications in the female reproductive tract: 1) assisted fertilization and 2) drug delivery to treat cancer. The release of captured spermatozoa is of importance on the one hand for assisted fertilization purposes, whereby spermatozoa need to be delivered to the site of fertilization and, on the other hand, for the release of the drug-loaded spermatozoa at the cancer treatment site. The release of spermatozoa has been achieved in four ways so far: i) opening of the microstructure by unfolding of microtubes to set free the confined cell. The incorporation of thermo-responsive material and a small temperature change was used to open the rolled-up microtubes^552^ or by shape-changing the stimuli-responsive 3D-printed carriers;^1440^ ii) a mechanical release mechanism involving four bendable arms on the front end of the sperm-capturing structure. When hitting an obstacle, a mechanical trigger bends the arms of the tetrapod structure to release the sperm cells from the front opening;^1438^ iii) friction forces acting on magnetotactic sperm when in contact with the cumulus complex were demonstrated as a release mechanism that leads to the spreading of magnetic particles and thus removal from the sperm’s surface;^1045^ and iv) enzymatic release of motile sperm cells from a hyaluronic acid–albumin microflake by proteases and hyaluronidases.^1443^

The previously mentioned magnetotactic sperm have been further optimized, with detailed characterization of sperm DNA integrity, motility, acrosome intactness, and other parameters. Their use in the first demonstration of oocyte *in vitro* fertilization has yielded promising results, paving the way for future *in vivo* applications. Additionally, various strategies for sperm assembly and the transport of multiple sperm are suggested to further enhance the chances of successful oocyte fertilization as well their visualization in *ex vivo* tissue using ultrasound and photoacoustic imaging suggests the possibility of using them for future *in vivo* assisted oocyte fertilization.^1442^ A concept similar to the sperm-carrying microrobots was applied to carry zygotes *in vitro* with a helical micropropeller by external magnetic rotating fields.^1521^ In this case, the robot had a spiral shape tuned to the size and shape of murine and bovine fertilized eggs. This approach is promising to provide higher embryo transfer success rates as well as to assist embryo implantation, in cases of recurrent embryo implantation failure.^547^ Studies on the locomotion of these carriers in cell-lining microfluidic chips, as well as the closed-loop control of using dual ultrasound–photoacoustic imaging, have been demonstrated.^1522,1523^

The second area of application inside the female reproductive tract is the potential of sperm-driven microrobots for cancer therapy.^1524^ Bovine spermatozoa were loaded with the anti-cancer drug doxorubicin hydrochloride. Surprisingly, the motility of the drug-loaded spermatozoa was not affected. Then, 3D-printed polymeric microstructures were coated with an Fe and Ti layer and used for capturing the drug-loaded bovine sperm.^1438^ While the sperm cells acted as the propulsion source, weak magnetic fields were used for directional guidance of the sperm cells to cancer spheroids, demonstrating the suitability of the system for drug delivery applications. The 3D-printed microstructure contained a four-armed front end, which offered a mechanical cell release mechanism. As the next step, human sperm was explored for drug delivery. Even though they load smaller drug amounts per cell (due to their smaller volume), they were successfully demonstrated to act as drug delivery robots.^1525^ Multiple drug-loaded human sperm were captured in semi-ellipsoid hollow microstructures. To increase the therapeutic effect, the anticancer drug CPT was immobilized on the artificial microstructure. Then, up to three human sperm were captured in the stream-lined cap, guided, and delivered to patient-derived ovarian cancer spheroids *in vitro*.^1525^

The protein–microflake carrier mentioned earlier was utilized for transporting up to 100 drug-loaded bovine sperm cells to cervical cancer tumor spheroids. This method also provides an alternative means to control drug dosage by adjusting the number of trapped drug-loaded sperm cells. Successful cell killing within these tumor spheroids and subsequent drug distribution following the somatic fusion of sperm and cancer cells was demonstrated.^1443^

##### 7.1.6.5 Joint Cavity

In the joint cavity, microrobots face a complex environment characterized by a liquid–solid interface filled with highly viscous synovial fluid. This challenge is further compounded in the case of cartilage damage, where the repair process is notably slower than that of other soft tissues and organs within the body. The movement of the joint can induce fluid disturbances, causing microrobots to deviate from the damaged site, thereby reducing the effectiveness of the repair process. Therefore, for successful application within the joint cavity, microrobots must possess the capability for long-term stable anchoring at the site of injury. This requirement is critical to ensure that therapeutic interventions remain effective despite the dynamic nature of the joint environment. Choi and co-workers^1526^ developed a medical microrobot system based on human adipose-derived mesenchymal stem cells specifically designed for the regeneration of knee cartilage. This microrobot is magnetically driven, enabling precise three-dimensional navigation, and utilizes a fixed magnetic ring to anchor the robot *via* lateral magnetic attraction at the site of cartilage injury. Such targeted delivery is crucial in ensuring that therapeutic agents are not dispersed by synovial fluid movement, thereby maximizing the regenerative potential of the treatment. Furthermore, the system’s efficacy was validated in an *in vivo* model, where it showed promising results in a rabbit model of knee cartilage defects. The ability to maintain stable anchoring and precise delivery of therapeutic agents at the injury site not only enhances the specificity and efficacy of treatment but also mitigates the drawbacks associated with traditional invasive methods. This innovation represents a significant advancement in the minimally invasive treatment of joint cartilage injuries, offering a novel solution that could revolutionize current therapeutic approaches.

##### 7.1.6.6 Respiratory Tract

In contrast, the respiratory tract primarily represents a gaseous or gas–liquid interface environment. The movement of air within the respiratory system and the surface tension at the gas–liquid interfaces significantly influence the motion of microrobots. Studies have shown that self-propelled microrobots hold potential in the treatment of pulmonary diseases, particularly in addressing bacterial pneumonia and lung cancer. Wang and co-workers demonstrated a biohybrid microrobot that combines green microalgae with DOX-loaded PLGA nanoparticles encapsulated by a red blood cell membrane.^1431^ This microrobot is capable of autonomously propelling itself deep into lung tissues to distribute drugs effectively. In a mouse model of lung metastasis, these microrobots, administered *via* intratracheal injection, were able to achieve efficient drug distribution within the lungs and significantly extend drug retention time. Compared to traditional passive drug delivery systems, these microrobots exhibited superior therapeutic outcomes, including a substantial reduction in metastatic burden and an extension of survival time, owing to their autonomous movement capabilities. This ability to navigate and deliver drugs within the complex lung environment highlights the microrobots’ potential to provide targeted and sustained therapeutic interventions. Beyond lung cancer treatment, Wang’s group also demonstrated that biohybrid microrobots could be utilized for the efficient treatment of pneumonia.^131^ They developed a similar natural microalga hybrid microrobot for pneumonia treatment. These microrobots enable active drug delivery and autonomous movement, making them particularly effective for *in vivo* treatment of lung infections. The research demonstrated that these microrobots could move rapidly in simulated lung fluid and distribute evenly throughout deep lung tissues with a mouse model of acute pneumonia, exhibiting low clearance by alveolar macrophages and exceptional tissue retention time. These findings underscore the microrobots’ substantial potential for use in intensive care settings, particularly in combating antibiotic-resistant bacterial infections. In summary, compared to traditional methods for treating pulmonary diseases, microrobots offer several distinct advantages: (i) their ability to autonomously navigate within the complex lung environment allows for efficient drug delivery; (ii) microrobots can extend the retention time of drugs at targeted sites, thereby reducing systemic toxicity; and (iii) microrobots can be precisely controlled using external fields, such as magnetic fields, enhancing the safety and efficacy of treatment. These advantages position microrobots as highly promising tools in the clinical treatment of lung diseases, potentially revolutionizing current therapeutic approaches and improving patient outcomes.

##### 7.1.6.7 Solid Tissues

Solid tissues represent a gel-like solid-phase environment. In such environments, microrobots should be small and slippery enough to navigate through or penetrate dense cellular matrices and polymer networks while minimizing damage to tissues and cells. To effectively move through these viscoelastic media, microrobots typically require specialized shape designs and propulsion mechanisms that provide sufficient thrust while reducing collateral damage to surrounding tissues. Fischer *et al*.^54^ successfully demonstrated the ability of microhelix propellers to penetrate the solid tissues of the eye’s vitreous body while minimizing tissue damage. In gel-like solid-phase environments, such as the vitreous humor of the eye, microrobots must overcome dense cellular gaps and polymer networks to avoid damaging tissues and cells. To achieve effective movement within these viscoelastic media, the researchers coated these microhelix propellers with a super-lubricant liquid layer, enabling them to penetrate the vitreous body and reach the retina without being impeded by the biological polymer networks present in the retinal tissue. The experimental results showed that these micropropellers, controlled by an external magnetic field, could achieve long-distance, high-speed propulsion while avoiding strong adhesion to tissues, thereby minimizing physical damage to the retina. This study highlights the critical role of surface treatments and structural designs in enhancing the penetration capabilities of microrobots within solid tissues, paving the way for more precise and less invasive therapeutic interventions. Additionally, Martel *et al*.^128^ demonstrated the penetration efficiency and drug delivery potential of microrobots within solid tumor tissues by leveraging the ability of magnetotactic bacteria (MC-1) to accumulate in hypoxic tumor regions and the guidance of external magnetic fields. The researchers used magnetic guidance and collective migration behavior to successfully transport drug-loaded nanoliposomes into the hypoxic zones of tumors. In a mouse tumor xenograft model, peritumoral injection of MC-1 bacteria combined with a directional magnetic field significantly increased the concentration of these bacteria in the central hypoxic region of the tumor. MC-1 bacteria, guided by the external magnetic field, were able to effectively penetrate deep into the tumor tissue, achieving a drug-targeting ratio of up to 55%. This work demonstrated that magnetotactic and oxygen-seeking microorganisms could significantly enhance the therapeutic efficacy of nanocarriers in the hypoxic regions of tumors. Compared to traditional passive drug delivery systems, magnetically driven active delivery systems exhibit superior tissue penetration capabilities, particularly in targeting difficult-to-reach hypoxic tumor areas, thereby significantly improving drug targeting and therapeutic outcomes. The remarkable advantages of microrobots in penetrating solid tissues are evident in their unique design and surface treatment technologies, which allow for effective navigation in complex physiological environments and precise delivery of drugs or other therapeutic agents to target sites, greatly enhancing the accuracy and efficacy of treatment while minimizing damage to healthy tissues.

##### 7.1.6.8 Cerebrospinal Fluid System

The cerebrospinal fluid (CSF) system, encompassing the central nervous system (CNS), including the brain and spinal cord, is a critical pathway for treating various neurodegenerative diseases and spinal cord injuries.^1527^ However, traditional drug delivery methods face significant challenges due to the complexity of the CSF space, the presence of the blood-brain and blood-spinal cord barriers, and the limited efficiency of conventional approaches. Common methods, such as lumbar puncture and ventricular catheters, enable drug delivery or sampling but have notable limitations, including restricted drug retention due to CSF flow, difficulties in targeting specific brain regions, and invasive procedures that may cause infections or brain damage.^1528^ Recently, microrobotic technologies have demonstrated great promise in addressing these limitations by enabling precise and efficient delivery of therapeutic agents within the CSF. The use of microrobots in combination with lumbar puncture provides a minimally invasive approach for navigating the spinal canal via cerebrospinal fluid, enabling precise neural connection restoration and localized drug delivery with high spatial precision. Compared to conventional methods, microrobots offer significant advantages, including noninvasive or minimally invasive procedures that reduce infection risks, precise navigation, prolonged retention in CSF flow environments, and improved drug bioavailability in the brain. Researchers have developed magnetically actuated microrobots and chemically driven nanomotors capable of traversing biological barriers and delivering neuroprotective drugs or regeneration-promoting factors with high precision.^1529,1530,1531^ For instance, magnetoelectric microrobots modulate the local microenvironment to promote spinal cord regeneration,^1529^ nanomotors utilize chemical gradients to achieve autonomous motion and deliver nerve growth factors,^1530^ and SCASRs self-assemble from stem cells and magnetic particles to restore neural connections through differentiation and secretion.^1531^ These microrobots enable targeted drug delivery to specific CSF regions, overcoming the limitations of conventional methods in precision and retention, and provide a robust foundation for advancing CSF-based therapeutic strategies.

Overall, the application of microrobots within different *in vivo* environments requires their optimization based on the unique physicochemical properties of each setting. This optimization is essential to ensure that microrobots can effectively perform their intended functions under complex and dynamic physiological conditions. To address the challenges posed by various internal environments, the design of microrobots must consider factors such as fluid dynamics, environmental viscosity, surface tension, and the density of cells and tissues. Through precise morphological design, the selection of propulsion mechanisms, and the application of surface treatment technologies, microrobots can achieve efficient movement, accurate localization, and stable therapeutic outcomes in diverse physiological environments. Only with such optimized design can microrobots realize their full potential in a wide range of clinical applications and offer more precise and effective treatment and diagnostics.

### 7.2 Micro/Nanorobots in Environmental Applications

The global water crisis, amplified by population growth, industrialization, and climate change, poses a significant challenge as less than 1% of Earth’s freshwater reserves are accessible.^1532^ The escalating threats of water pollutants have severe ecological implications and represent a danger to the health of all living beings^1533,1534,1535,1536,1537^ while current water remediation methods present several limitations.

In this context, self-propelled micro/nanorobots emerge as a promising solution, combining the characteristic size-dependent properties of micro/nanomaterials with the active motility, which allows them to enhance the mass transfer in diffusion-limited reactions, such as those occurring in conventional water purification approaches, without external agitation.^145,931,1538,1539,1540,1541^ For example, micro/nanorobots can be programmed to leverage electrostatic^1542^ or phoretic interactions,^1543^ to adsorb pollutants “on-the-fly”. In addition, micro/nanorobots can perform advanced oxidation processes, achieving the efficient degradation of contaminants. Fenton,^1544^ Fenton-like,^1545^ photo-Fenton^1546^ reactions, and heterogeneous photocatalysis^1547^ are prominent methods, generating ROS, such as superoxide radicals (O_2_^–•^), hydroxyl radicals (^•^OH), and singlet oxygen (^1^O_2_), which cleave water pollutants into smaller compounds until they are completely mineralized into water and CO_2_.

Additionally, peroxymonosulfate-based advanced oxidation processes have recently gained significant attention for the ability of this strong oxidizing agent to generate sulfate radicals (SO_4_^–•^).^1548^ Of note, advanced oxidation processes are activated by catalysts, such as Fe-based materials in Fenton and photo-Fenton reactions, or photocatalytic semiconductors in photocatalysis. An alternative and more sustainable approach exploits enzyme catalysis to induce the decomposition of water pollutants.^233^

The synergy between the self-propulsion ability and these remediation mechanisms has innovated water purification approaches. For example, micro/nanorobots’ powerful locomotion accelerates the capture of heavy metals in water, like As,^1549^ Cd,^1550^ Pb,^1551^ Hg,^1552^ and Cu,^1553^ which pose significant concerns to human health. Moreover, micro/nanorobots exploit autonomous movement and (photo)­catalytic or enzymatic reactions to boost the adsorption and degradation of toxic organic pollutants from water, as demonstrated for pesticides,^1554^ antibiotics,^1555^ hormones,^1556^ psychoactive drugs,^1557^ dyes,^1558,1559,1560^ and other dangerous compounds.^1561,1562,1563,1564^ Microplastics (<5 mm), nanoplastics (<1000 nm), and polymer chains have been successfully tackled by micro/nanorobots by various strategies,^1565,1566^ including their removal by electrostatic or phoretic interactions,^1567,1568^ or physical separation methods,^1569^ and their subsequent decomposition by photocatalytic,^1570,1571,1572,1573,1574^ photo-Fenton,^1575^ or enzymatic^1576^ mechanisms. Taking advantage of hydrophobic properties, micro/nanorobots show also potential in managing the alarming issue of oil spills in water bodies, by removing^782,1577,1578,1579,1580^ and, ultimately, degrading oil droplets.^1282^ Furthermore, micro/nanorobots promise to address the issue of water contamination from pathogenic bacteria and viruses by antibacterial^1581,1582^ and antiviral properties,^1583^ or by photocatalysis,^1583^ which is particularly effective in the eradication of bacterial biofilms.

Instead of further describing the strategies associated with the removal or degradation of a specific type of pollutant, this section shifts the focus toward the key factors for further advancing the application of micro/nanorobots in water purification, including (1) the imperative for enhanced performance, (2) multifunctionality, (3) sustainability and environmentally friendly aspects, and (4) potential in soil remediation, which are schematically summarized in Figure 17.

**Figure 17:**
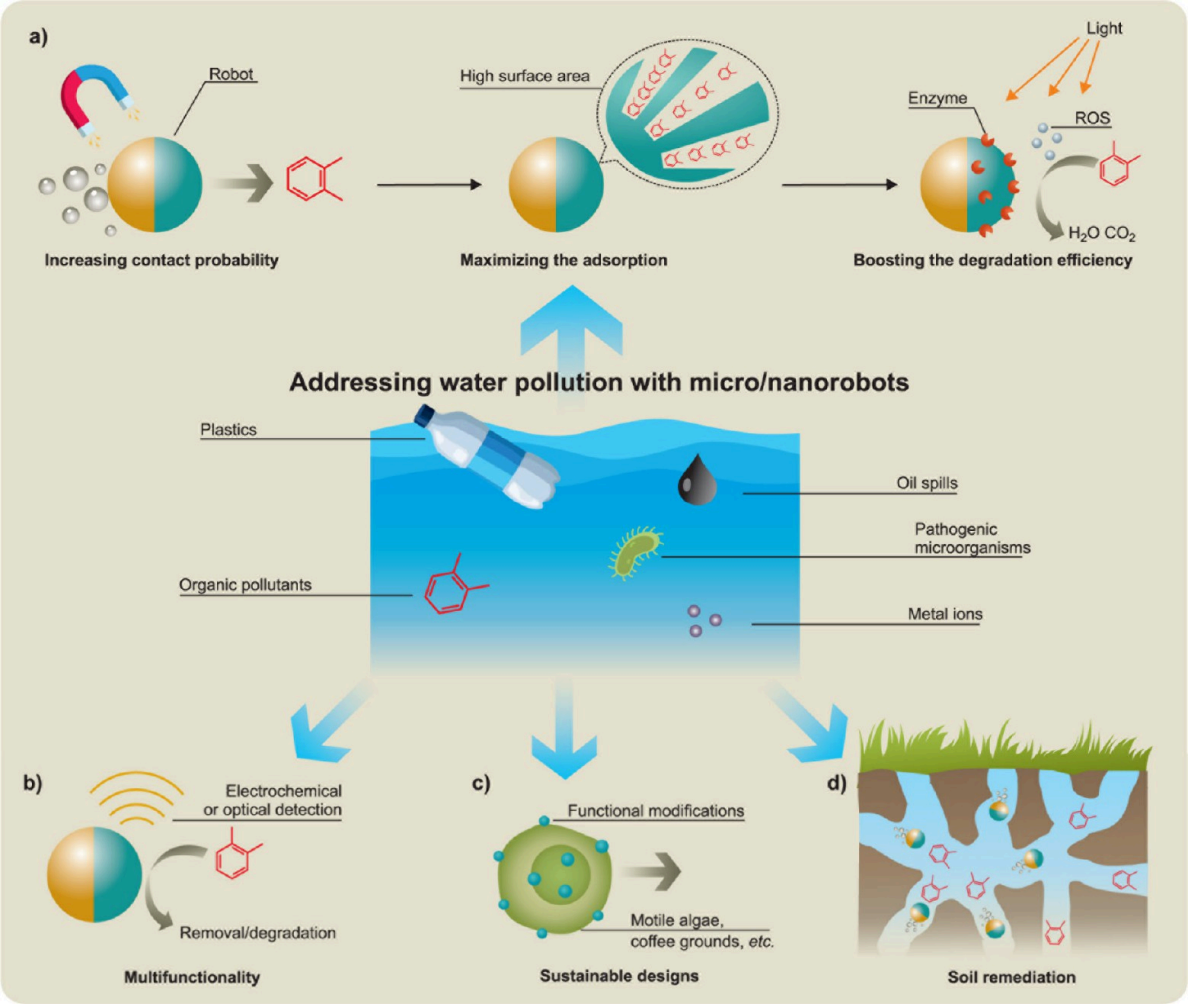
**Key strategies to enhance performance, multifunctionality, and sustainability, and extend the applicability of micro/nanorobots for environmental remediation. a)** Magnetic or bubble propulsion and motion mode optimization enhance pollutant removal/degradation efficiency by improving micro/nanorobots’ speed and interaction with pollutants. Integration of high-surface area materials increases adsorption sites while photocatalytic and enzymatic mechanisms synergistically improve the degradation efficiency. **b)** Multifunctionality allows micro/nanorobots to remove or degrade pollutants (heavy metals, organic molecules, and nanoplastics) while enabling simultaneous detection through colorimetric or electrochemical methods. **c)** Sustainable designs focus on reducing environmental impact by using self-motile microorganisms (bacteria and microalgae) as engines, hybrid bio-inorganic systems (*e.g.*, loading magnetic nanoparticles onto microorganisms), and exploiting captured pollutants as active components for further purification processes or as fuels. **d)** Self-propulsion enhances catalyst diffusion in the soil subsurface, improving the degradation of pollutants such as toxic pesticides.

#### 7.2.1 Strategies to Enhance Performance

Improving efficiency is key in water purification due to the rising challenges posed by water pollution. Micro/nanorobots show great promise, but enhancing their efficiency, *i.e.*, pollutant removal/degradation rate, is essential. This depends on three factors: (i) contact probability with pollutants, (ii) number of adsorption sites, and (iii) (photo)­catalytic activity for pollutant degradation. This section explores innovative strategies to improve these aspects, paving the way for more effective micro/nanorobot applications in treating contaminated water.

With a few exceptions,^1584^ stronger self-propulsion leads to more interactions with pollutants and an improved removal or degradation efficiency, driving efforts to increase micro/nanorobots’ speed. However, each propulsion mechanism has limitations that must be addressed for practical use. For instance, light-driven micro/nanorobots rely on self-electrophoresis or ionic self-diffusiophoresis, which are less effective in high-ionic-strength environments like seawater.^1585^ In response, magnetically actuated and bubble-propelled micro/nanorobots have gained attention.^1586,1587^ An effective strategy combines magnetic and photocatalytic properties for effective motion and pollutant degradation. In this context, a noteworthy example is a magnetic photoactive microswarm, consisting of magnetic Fe_3_O_4_ nanoparticles coated with a Bi_2_O_3_/Ag photocatalytic shell and designed to magnetically navigate, adhere to, and photocatalytically degrade polypropylene microfibers from COVID-19 face masks.^1588^ While magnetic micro/nanorobots allow precise motion control, photocatalysis can influence their navigation. Fe_3_O_4_@BiVO_4_ microrobots show that UV light alters magnetic movement direction, but without affecting performance.^1589^ This synergy between magnetic and photocatalytic effects improves the pollutant removal efficiency, achieving 73% degradation of microplastics and model dye in 30 minutes.

While combining magnetic fields and light ensures maximum efficiency, it increases energy consumption. Therefore, enhancing the speed of micro/nanorobots actuated by a single mechanism is of great interest. Investigating different motion modes is a valuable strategy to improve the speed and performance of micro/nanorobots. Inspired by the flic–flac movement of the Moroccan spider *Cebrennus rechenbergi*, Fe_3_O_4_@Ce-MOF nanorobots, consisting of a Ce-based MOF combined with magnetic Fe_3_O_4_ nanoparticles, were developed with rotating and tumbling motion modes.^1132^ These nanorobots can rotate at ∼500 rpm or tumble at ∼17 μm·s^–1^ when driven by an appropriate magnetic field. Switching from rotation to tumbling increases the dye adsorption rate compared to the static state due to the stronger flow field generated by the tumbling mode, enhancing interaction between the dye and nanorobots.

A similar concept is applied to light-driven nanorobots with motion controlled by changing light wavelength.^1590^ In this study, CAT-PAH@COF nanorobots were prepared by loading catalase and spiropyran on the covalent–organic framework (COF) RT-COF-1, chosen for its large surface area and pore size. Catalase decomposes H_2_O_2_ fuel for self-propulsion while spiropyran, a molecular photoswitch, changes from hydrophobic to hydrophilic upon switching from red to blue light, favoring the diffusion of H_2_O_2_ within the COF structure and, thus, boosting nanorobot speed 1.8-fold (11.9 μm·s^–1^) and dye removal efficiency from 18% to 79.8% in 30 minutes.

Besides, buoyancy-driven catalytic microrobots show promise for vigorous self-mixing in water, accelerating the degradation of contaminants *via* advanced oxidation processes.^1591^ Microrobots decorated with Ag and Pt nanoparticles float to the surface due to O_2_ bubbles from Pt-catalyzed H_2_O_2_ decomposition, then sink as bubbles evolve, creating fast motion (∼12 mm·s^–1^) for up to 6 hours. This intermixing, along with the peroxidase-mimicking activity associated with the production of ^•^OH and ^•^O_2_^–^ upon the reaction between Ag and H_2_O_2_, enhances MB degradation from ∼32% (without Pt) to ∼90% (with Pt) in 15 minutes.

After exploring the mechanisms to enhance the speed of micro/nanorobots, the focus shifts to improving their adsorption capacity by increasing the binding sites, crucial for boosting pollutant removal efficiency. A key strategy involves using MOFs, which offer tunable chemical properties, nanoporous structures, and high surface areas to ensure pollutant accessibility and abundant binding sites. As a consequence, MOFs have significantly impacted water purification, particularly by addressing the adsorption of organic pollutants and metal ions.^1226,1592^ One example involves nanorobots featuring a magnetic MnFe_2_O_4_ core and a hierarchical MOF structure made by MIL-53­(Fe) for its magnetic properties and UiO-66­(Zr) for its high stability, low toxicity, and surface carboxylic groups (−COOH), promoting the attraction of metal cations.^1593^ These nanorobots bubble-propel through H_2_O_2_ decomposition and exhibit high adsorption capacities for Pb­(II) (92.72%) and Cd­(II) (84.14%) in tap water due to the enhanced contact probability resulting from the active motion and the hierarchical MOF structure providing numerous adsorption sites. Additionally, their magnetic properties enable easy collection and recycling. Moreover, Nafion-based micro/nanorobots and pumps have been demonstrated to selectively and efficiently remove cadmium ions in the presence of other saline cations, with the heavy metal itself acting as the fuel to activate the interfacial fluid flows and the self-propulsion of Nafion system.^302,305,307^ These findings highlight the importance of integrating an excellent adsorber for efficient pollutant adsorption. On the other hand, to completely purify the water, an ideal micro/nanorobot should also integrate a catalytic material to perform the degradation of the captured pollutants.

The catalytic properties of micro/nanorobots play a key role in water purification efficiency but their performance can be limited by environmental conditions, such as pH levels. For instance, the Fenton reaction, essential for producing ^•^OH, works effectively in acidic conditions (pH ∼3), whereas real-world contaminated water is usually neutral, requiring broader pH adaptability. Additionally, low pH can reduce the mobility or stability of some micro/nanorobots, emphasizing the need for high catalytic activity in neutral water. In this regard, Fe-MnO_2_ core–shell microrobots, obtained by growing flower-like MnO_2_ nanoplates on Fe microspheres, reach high bubble-propulsion speeds up to 300 μm·s^–1^ in solutions of H_2_O_2_ and surfactants, enhancing the degradation of tetracycline through a combination of adsorptive bubble separation and Fenton/Fenton-like reactions catalyzed by Fe and MnO_2_.^1594^ While the adsorptive bubble separation method facilitates the removal of the tetracycline trapped in the foam produced during the microrobots’ propulsion, H* was found to contribute to the degradation process for the first time. This breaks down H_2_O_2_ to produce ^•^OH even in neutral water, which results in an 85% tetracycline degradation efficiency in 2 hours. Despite requiring higher H_2_O_2_ concentrations and surfactants, these microrobots show great potential for water purification across a broad pH range. In addition, hydrophobic microrobots—based on alkanethiol-modified tubular microengines—have been shown useful for efficient “on-the-fly” removal of oil droplets.^147^

Efforts to enhance pollutant degradation efficiency in micro/nanorobots have also focused on integrating multiple methods. A promising approach combines photocatalysis with enzymatic degradation, as shown by Au-incorporated ZnO microrobots modified with laccase enzyme.^1595^ These microrobots propel *via* self-electrophoresis under UV light in H_2_O_2_ solutions, increasing interactions with pollutants like oxytetracycline, an animal antibiotic. The synergy between ZnO–Au photocatalysis and laccase activity increased oxytetracycline degradation efficiency (65% in 12.5 minutes), outperforming individual methods.

In summary, research on micro/nanorobots has identified several promising strategies to improve their performance for water purification. Magnetic actuation of photocatalytic micro/nanorobots, nature-inspired motion manipulation, and buoyancy-driven movement show great potential for enhanced interaction with pollutants. The use of large surface area materials, such as MOFs, improves adsorption, while novel mechanisms effective in neutral conditions, and the combination of photocatalysis with enzymatic degradation, further advance their practical application. However, challenges remain, such as optimizing energy-efficient propulsion, reducing the use of toxic fuels, and deepening the understanding of micro/nanorobot–pollutant interactions to fully exploit their capabilities.

#### 7.2.2 Sense and Remediate

In the quest for more effective water purification, multifunctional micro/nanorobots are emerging as a groundbreaking solution, being able to excel at removing and also detecting specific contaminants, offering a dual function that marks a significant leap forward in water quality management. For instance, a study presents multifunctional self-propelled structural color cylindrical robots designed for efficient and intelligent heavy metal ions adsorption and self-reporting.^1596^ Synthesized through the formation of a tubular colloidal crystal template, followed by the infiltration of a carboxymethyl chitosan/polyacrylamide solution and the addition of Pt nanoparticles for catalytic propulsion, these robots (1 mm in size) are capable of self-propulsion in H_2_O_2_ solutions up to 30 mm·s^–1^ speed to enhance heavy metal ion adsorption and possess a unique real-time self-reporting capability, which uses structural color changes to signal the presence of heavy metal ions: initially red, the self-propelled structural color cylindrical robots shift to green upon heavy metal ion adsorption due to structural shrinkage, with optical reflectance peaks correlating with heavy metal ion concentrations. The detection limits for various heavy metal ions, such as Cd­(II), Cu­(II), Fe­(III), and Pb­(II), are impressively low (∼1–2 mg·L^–1^). Additionally, the self-propelled structural color cylindrical robots can undergo multiple adsorption and desorption cycles, demonstrating durability in both tap and lake water.

The colorimetric detection of contaminants recently gained increasing attention due to the high sensitivity and selectivity of this approach without requiring expensive materials or sophisticated instruments. This sensing strategy is based on the enzyme-like activity of some micro/nanorobots’ designs.^1597,1598,1599^ Among them, there are the hierarchical horseradish peroxidase-MIL-100­(Fe)/Mn_2_O_3_@TiO_2_@Fe_3_O_4_ Janus magnetic bubble-propelled microrobots, which can perform the colorimetric quantification and degradation of hydroquinone, a compound extensively used in industry.^1600^ The detection of hydroquinone is based on the powerful motion of the microrobots, enhancing the contact probability, and the horseradish peroxidase and MOF MIL-100Fe, acting as natural and nanoenzymes. This strong peroxidase-like activity causes a chromogenic substrate like 3,3',5,5'-tetramethylbenzidine to oxidize and reduce to a colorless form upon introducing hydroquinone. This variation of color, appreciated by the naked eye or by UV-Vis spectroscopy, allows for sensitive and selective detection of the pollutant with a low detection limit of 1.84 μM without requiring expensive or complex equipment operated by trained personnel, such as mass spectroscopy techniques or electrochemical sensors, and it is also suitable for the on-site monitoring of water quality. Furthermore, the microrobots exhibit excellent performance in water purification, photocatalytically degrading hydroquinone with 90% efficiency in 80 minutes.

Building on the same principle, an alternative nanorobot design eliminates the use of horseradish peroxidase and instead leverages the strong nanozyme activity of novel nanorobots. These consist of ascorbic acid-functionalized NiMn-layered double hydroxides on halloysite nanotubes, modified with Ag nanoparticles to enable bubble propulsion. This design is capable of calorimetrically detecting phenol in water with a detection limit of 0.225 μM. Furthermore, the nanorobots efficiently degrade phenol by a Fenton-like reaction assisted by AA, reaching 95% degradation efficiency in 90 minutes.^1601^

The previous examples deal with the detection and removal of atomic- or molecular-scale (organic molecules) entities, presenting limitations when applied to larger, solid pollutants such as micro/nanoplastics. Specifically, nanoplastics are extremely elusive due to their tiny size, so they easily remain suspended in water while most microrobots are confined to the bottom of the vessel due to their higher weight. Addressing these challenges, a study introduces MXene-derived γ-Fe_2_O_3_/Pt/TiO_2_ microrobots for capturing and detecting nanoplastics due to a negative photogravitactic movement in 3D space.^151^ By exploiting the synergy between light-driven 3D motion, pH-programmable surface charge, and layered structure, the microrobots efficiently and rapidly trap oppositely charged polystyrene nanoplastics with a diameter of 50 nm between the layers, achieving 97% removal efficiency within 1 minute. In addition, the microrobots can be magnetically transferred to a miniaturized electrode system to electrochemically detect nanoplastics, enabling quantification in the concentration range of 10^6^–10^14^ particles per milliliter.

In addressing the multifunctionality of micro/nanorobots for water purification, it becomes evident that their versatility in pollutant detection and degradation is closely tied to intricate designs involving multi-material compositions and multi-step preparation methods, raising concerns about their scalability for real-world applications. Therefore, it is imperative to strike a balance between the desired multifunctionality and the practical constraints associated with the fabrication and use of micro/nanorobots.^1602^ The central theme of sustainability is equally important, as emphasized by the need for environmentally conscious approaches ensuring the long-term viability and applicability of this innovative technology in water purification.

#### 7.2.3 Sustainable Designs

The integration of micro/nanorobots in water purification is continuously advancing, with sustainability becoming a key focus. This section highlights the importance of designing these systems to effectively remove pollutants while being environmentally friendly and resource-efficient. Sustainable designs use eco-friendly materials, biodegradable components, and greener propulsion mechanisms to minimize the environmental impact without compromising efficiency.

The utilization of microorganisms, such as bacteria or algae, in micro/nanorobots’ design offers a sustainable and cost-effective approach to avoid costly or hazardous materials and complex synthesis processes. These microorganisms provide several advantages, such as natural motion, biological functionality, and biocompatibility, aligning with sustainability principles and offering an eco-friendly solution for propulsion and pollutant removal in water purification. In this context, natural algae can be utilized to obtain sustainable microrobots for water purification. Functionalized with ACE2 receptors to target SARS-CoV-2 spike proteins, autonomously swimming algae have been employed to formulate biohybrid microrobots that achieved efficient virus capture and removal without external energy inputs. With high-speed and long-lasting movement (>24 hours) in various aquatic environments, these microrobots outperformed their static counterparts, achieving up to 95% removal of viral proteins and 89% of pseudovirus.^1583^ In the field of bio-based micro/nanorobots, *Magnetospirillum magneticum* strain AMB-1 shows also great potential as a biological self-motile and magnetic structure for the removal of organic pollutants from water.^1603^ The key feature of these magnetotactic bacteria is the presence of magnetosomes—membrane-enclosed magnetic particles composed of iron minerals that act like tiny compass needles—which enable bacteria to move along the geomagnetic field lines to reach optimal oxygen and nutrient conditions within their habitats. By using an external magnetic field, these bacteria are turned into biobots without any further modification, enabling the removal of chlorpyrifos (an organophosphate pesticide) from real water samples with ∼70% efficiency in 10 minutes.

As an alternative to intrinsically magnetic microorganisms, magnetic properties can be introduced to biological structures, such as *Chlorella vulgaris* algae cells, by integrating magnetic Fe_3_O_4_ nanoparticles, realizing biological–inorganic hybrid microrobots to remove micro/nanoplastics from water.^1604^ Owing to the strong fluid flow generated during their magnetic movement and surface properties, the microrobots attract oppositely charged polystyrene microplastics (1.5 μm in size) and nanoplastics (50 nm in size) in water with efficiency approaching ∼70% and 90% within 40 minutes, respectively, showing also excellent reusability and efficiency retention in real water samples.

Instead of using living microorganisms but aiming at emphasizing sustainability, magnetic microrobots can be produced also by modifying spent coffee grounds, which are a biodegradable, readily available, and cheap material, with magnetic iron oxide nanoparticles by a green chemistry approach.^1605^ The resulting CoffeeBots exhibit fast movement (∼5500 μm·s^–1^) and can efficiently capture oil droplets and microplastics from seawater thanks to their intrinsic hydrophobicity, achieving 99% oil removal efficiency in 3 minutes and 64% microplastic removal efficiency in 1 hour. Reusable and recyclable, CoffeeBots are expected to have low environmental impact and can even degrade organic pollutants when loaded with ascorbic acid, removing 90% of a model dye in 40 minutes. This work underscores the potential of waste materials for sustainable water purification. Envisioning a more sustainable future for micro/nanorobots in water purification, harvested microplastics could be repurposed as valuable resources for developing next-generation microrobots, fostering a greener and more eco-friendly approach.

Electronic waste is another material with the potential for valorization. Electronic components contribute to soil and water pollution by releasing dangerous substances, such as toxic additives, heavy metals, and halogenated organic pollutants. On the other hand, electronic devices contain also various metals, particularly precious and useful metals such as gold, whose recovery and reuse allow for addressing environmental pollution and fostering a circular economy. With this aim, a study introduces magnetic nanorobots featuring yolk–shell mesoporous structures made from Fe_3_O_4_ nanoparticles coated with an Au­(III) ion-imprinted polymer to recover Au in water.^1606^ The imprinting process creates specific cavities that allow the nanorobots to selectively adsorb Au­(III) ions, which when combined with the magnetic motion yields a 99% removal efficiency in 30 minutes, even from the leachates of damaged smartphones. Additionally, the nanorobots successively degrade organic pollutants, like 4-nitrophenol and dyes by exploiting the catalytic properties of the captured Au. A closed-loop system for sustainable electronic waste treatment is proposed that involves four key stages (adsorption, desorption, regeneration, and degradation).

In industrial and wastewater disinfection, high levels of organic pollutants and residual H_2_O_2_ pose environmental risks. An innovative approach highlights the sustainability of transforming pollutants into components for micro/nanorobots to achieve environmentally friendly decomposition of H_2_O_2_.^1607^ This study presents hollow MnO_2_ microrobots that, when introduced into phenol-polluted water, polymerizes the pollutants instead of oxidizing them. The polymeric coating regulates MnO_2_ exposure and H_2_O_2_ decomposition, ensuring controlled motion and preventing excessive heat during the propulsion, and offering a safe and innovative method for pollutant removal and water treatment.

Differently from the previous strategy, another study reveals the possibility of exploiting water pollutants as fuel to power the self-propulsion and degradation performance of nanorobots.^1608^ In particular, Fe_3_O_4_@silica nanoparticles modified with laccase or lipase enzyme show enhanced diffusion when introduced in solutions contaminated by bisphenol A and Congo Red, substrates for laccase, or oily triglyceride triacetin, a substrate for lipase. In this way, the pollutants are almost totally consumed in 40 minutes with 94–99% degradation efficiency. Recently, it has been also demonstrated that bubble-propelled laccase-functionalized MnO_2_ micromotors can convert urea, a major component of urine, which is commonly found in domestic and industrial wastewater, into ammonia in 15 minutes.^1609^ Consequently, this study demonstrates an innovative approach to achieve both pollution removal and sustainable energy production.

In conclusion, the exploration of sustainable design principles in micro/nanorobots for water purification reveals a transformative potential in mitigating environmental challenges. From harnessing the capabilities of bio-inspired microorganisms to repurposing waste materials and pollutants, these approaches align with ecological consciousness and offer practical and scalable solutions. By continually pushing the boundaries of innovation and embracing eco-friendly practices, further research in sustainable micro/nanorobots can revolutionize water purification, balancing technological progress and environmental preservation.

#### 7.2.4 Beyond Water: Micro/Nanorobots for Soil Purification

The active movement of micro/nanorobots enhances mass transfer, improving pollutant contact and boosting degradation efficiency, especially in water samples. However, applying this approach to soil remediation, particularly in the subsurface environment, presents various challenges. Soil contamination, exacerbated by human activities like pesticide use in agriculture, requires innovative solutions. *In situ* chemical oxidation using metal oxide catalysts and oxidizing agents is an efficient method, but the limited transport of purification agents in the soil remains an obstacle.^1610^ Self-propelled micro/nanorobots, capable of autonomous movement, offer a promising solution to improve the dispersion of these agents in soil. For this purpose, a recent study proposes bio-templated tubular microrobots that integrate Fe_3_O_4_ nanoparticles for magnetic guidance, MnO_2_ nanosheets for bubble-propulsion in H_2_O_2_ solutions, and g-C_3_N_4_ nanosheets to enable visualization of the microrobots.^1539^ These microrobots showed a high transport efficiency in complex microenvironments, simulating the soil subsurface and degradation efficiency of 82–99% against a broad spectrum of pollutants, including various water-soluble antibiotics and water-insoluble polycyclic aromatic hydrocarbons, in 15 minutes through Fenton-like reactions based on the production of ^•^OH upon reaction with H_2_O_2_ and sulfate radicals SO_4_^–•^ upon reaction with persulfate.

Although the use of micro/nanorobots for soil purification has shown promise in laboratory settings, challenges remain for real-world applications. For instance, their recovery and reusability become particularly difficult as microrobots penetrate deeper into the soil. To address these limitations, future designs may incorporate biodegradable catalysts, reducing concerns about retrieval and environmental impact. Moreover, the potential use of micro/nanorobots extends beyond soil remediation. They could also be employed for purifying other water/solid interfaces, such as degrading residual pesticides on plants. This can be achieved by spraying an aqueous suspension of catalytic micro/nanorobots onto contaminated surfaces, offering a new avenue for environmental remediation.

### 7.3 Micro/Nanorobots in Analytical Sensing and Biosensing

Micro/nanorobots possess ability to navigate themselves through a liquid sample, thus enhancing their ability to reach, interact, capture, and transport target analytes. As such, self-propelled micromotors open a new dimension in analytical chemistry, with a vast development since the first observation of accelerated motion of Au-Pt nanomotors in the presence of Ag ions back in 2009.^140^ Such a pioneer work reveals the opportunities of micromotors for analytical sensing, as summarized in Figure 18. What makes micromotors particularly attractive is the enhanced motion and great acceleration of the reaction kinetics—especially in catalytic micromotors—which allow for a great reduction of the detection times; most importantly, the amount of sample needed to just a few microliters.^1611^ This is of great relevance in clinical scenarios where, in some cases, the samples are difficult to obtain or the amount is limited, for example in blood from neonates, cerebrospinal fluid, among others.^1612,1613,1614^

A diverse array of micro/nanorobotics, such as microtube rockets, jellyfish-like micromotors, and Janus nanoparticles, has demonstrated remarkable proficiency in the detection of critical biomarkers such as oligonucleotides, DNA, RNA, proteins, circulating tumor cells, viruses, bacteria, pH levels, glucose, and trace elements.^1615^ These miniaturized devices leverage unique properties such as motion-induced enhancement of hybridization efficiency, catalytic propulsion, and magnetic guidance, facilitating expedited and reliable diagnostic processes.

Chemical sensing and biosensing with micromotors can be achieved by three broad categories according to the detection principle: motion-based sensing by monitoring the changes in the micromotor speed by the action of certain analytes, optical sensing either with colorimetric or fluorescence detection, or by electrochemical sensing. The type of micromotor, motion mechanism, and overall design in terms of functionalization of operation are highly dependent on the type of detection principle.

**Figure 18:**
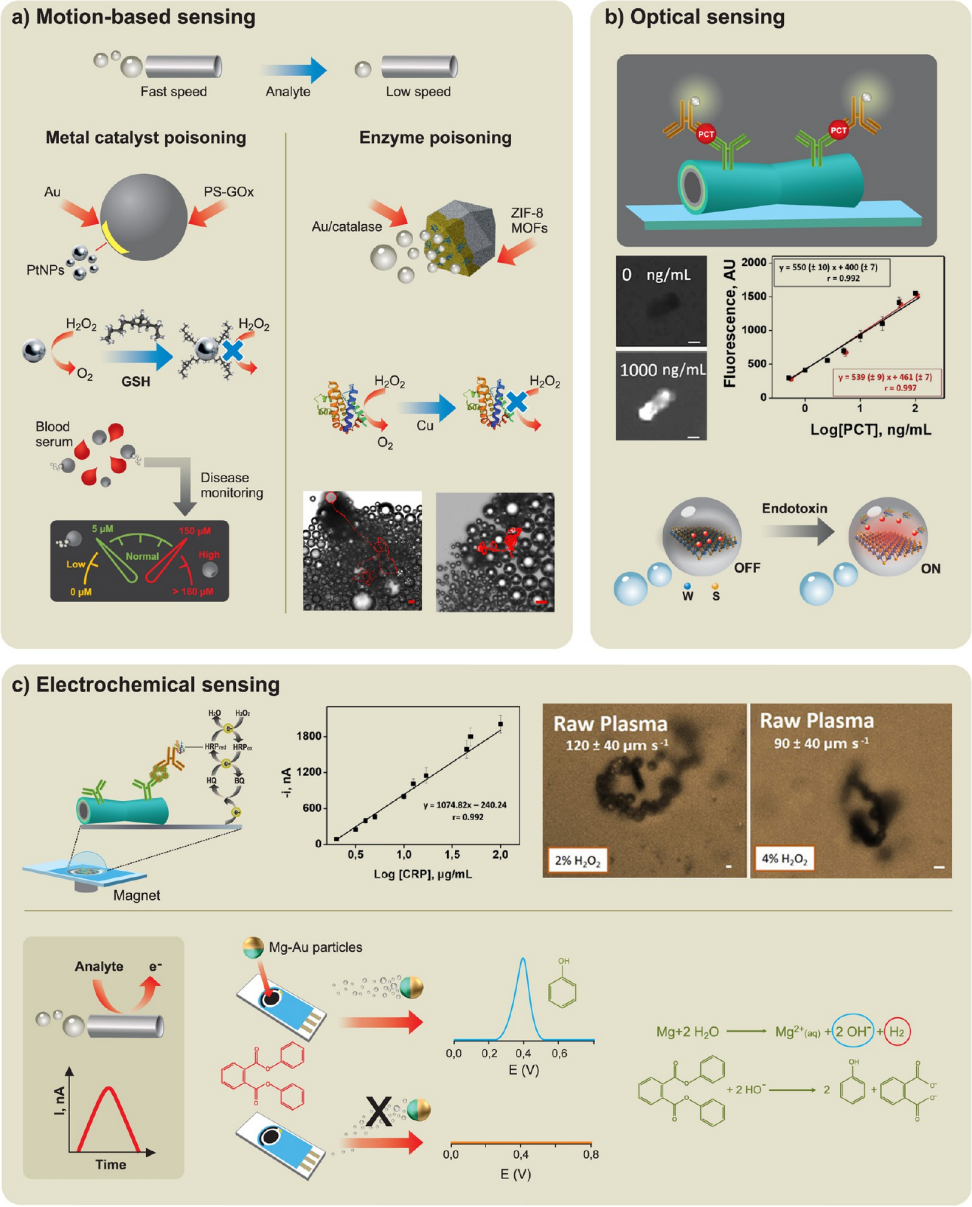
**Overview of micromotors for analytical sensing and biosensing. a)** Motion-based sensing. On top, a schematic of the principle for detection, based on the decrease in the micromotor speed in the presence of the target analyte. As an example of metal catalyst poisoning, polystyrene (PS)-graphene oxide (GOx)-Pt nanoparticles (PtNPs) catalytic micromotors are illustrated. Specific attachment of glutathione (GSH) results in poisoning of the PtNPs, reducing the speed in a concentration dependent manner, allowing for disease monitoring. Reprinted with permission under a Creative Commons CC-BY License from ref 1625, Copyright 2021 American Chemical Society. As an example of enzyme poisoning, zeolitic imidazole (ZIF) MOFs propelled by catalytic decomposition of the hydrogen peroxide by the enzyme catalase are poisoned in the presence of Cu. The time-lapse microscopy images show the micromotors navigation in cerebrospinal fluid samples, where the presence of Cu served as indicator for Alzheimer disease monitoring. Reprinted with permission from ref 1626, Copyright 2024 Royal Society of Chemistry. **b)** Optical sensing. On top, a micromotor based immunoassay using polypyrrole/niquel Pt-based micromotors for procalcitonin (PCT) detection from very low-birth-weight infants. The images illustrate the generation of an immunosandwich, using a FITC-labelled secondary antibody. The microscopy images show the fluorescence in the micromotor surface in the absence (0 ng/ml) and presence (1000 ng/ml) of PCT, with the corresponding calibration plot. Reprinted with permission from ref 1399, Copyright 2020 American Chemical Society. Bottom part shows the use of polycaprolactone/Pt Janus micromotors loaded with WS_2_ and modified with a rhodamine labelled affinity peptide for OFF-ON endotoxins detection. Adapted with permission from ref 1638, Copyright 2020 Elsevier. **c)** Electrochemical sensing with micromotors. On top, on-the-move immunoassay using carbon-based micromotors modified with antibodies for C-reactive protein (CRP) detection. Right part shows the corresponding calibration plots and the time-lapse microscopy images of the micromotor propulsion in raw plasma samples in the absence of surfactant at different H_2_O_2_ concentrations: in the presence (2% H_2_O_2_) and absence (4% H_2_O_2_) of surfactant. Reprinted with permission from ref 1651, Copyright 2020 Elsevier. The bottom part shows Mg-based micromotors for assisted electrochemical detection of nonelectroactive phthalates. Reprinted with permission from ref 1649, Copyright 2016 American Chemical Society.

#### 7.3.1 Motion-Based Sensing

Motion-based sensing approaches rely on simple designs, mostly based on catalytic propulsion. For example, accelerated motion can be achieved by modifying certain detection probes with catalytic particles in the form of microtube rockets. DNA and RNA sensing have been achieved using Ag-tagged detection probes, generating only a sandwich in the presence of the target nucleic acid, increasing thus the motion in H_2_O_2_ solutions, which in turn can be related to the analyte concentration.^139^ These nanomotors can detect DNA with a sensitivity down to 40 fmol by correlating their motion speed with DNA concentration. A similar strategy has been adopted using DNA-modified poly­(3,4-ethylenedioxythiophene) polystyrene sulfonate/Au catalytic micromotors^1616^ or polystyrene/Pt beads for Zika virus detection.^1617^ Jellyfish-like micromotors also showed enhanced sensitivity in DNA detection in protein-rich environments due to their stable and reproducible motion characteristics.^1618^ Liang and co-workers introduced a biodegradable MOF-based nanobot that uses chemically driven buoyancy to “find-and-fetch” circulating tumor cells.^1619^ Functionalized with antibodies against a carcinoembryonic antigen, these nanobots specifically recognize cancer cells within a mixed cell population and autonomously transport and separate them due to a drastic buoyancy change when the nanobots bind. Importantly, these MOF nanorobots are easily degraded, allowing the captured cells to regain their full metabolic potential for further studies. This innovative micro/nanorobotic approach offers significant biomedical opportunities. Like catalytic biosensing, electrokinetic active particles can be exploited for motion-based biosensing.^139^ This strategy involves decorating the more polarizable region of particles with biorecognition elements (e.g., antibodies, aptamers) such that, as biomarkers selectively bind to the recognition elements on particles, the mismatch in electrical polarizability is altered,^1620^ changing the transport characteristics of the particles in a manner that can be observed by microscopy.^1621^

To simplify the overall motion-sensing procedures, deceleration of the micromotor’s motion can be easily achieved by two strategies. As such, PEDOT/Au tubular or Janus micromotors have been modified with catalase, an enzyme that can be poisoned by metal ions or chemical threats in water.^1622,1623^ Toward even more simplification, certain metals have been probed to poison the catalytic Pt layer of template-prepared and halloysite-based nanotubes, avoiding the use of enzymes or additional responsive/sensing units.^1624^ While simple, all the above-mentioned strategies require the use of high-resolution optical microscopes to track and monitor changes in micromotor speed, which prevent practical on-site applications in the analytical domain. While still more advances are needed, a work from 2021 described a 3D-printed device integrating a mobile phone equipped with a low-cost high-resolution optical lens to observe the motion of micromotors.^1625^ In this work, motion-based sensing of glutathione has been illustrated with a good analytical match using both the portable intelligent phone-based device and a high-resolution optical microscope. The principle behind sensing relies on poisoning the Pt catalytic layer of graphene oxide-based micromotors by the thiol groups present in glutathione (Figure 18a­(i)). Another elegant recent strategy is illustrated in Figure 18a­(ii), where catalase-powered ZIF-8 micromotors experience a decrease in the speed in the presence of Cu, which is overexpressed in the cerebrospinal fluid of Alzheimer disease patient samples. The as-developed fast screening method allows for the discrimination of samples at the different states of the disease using 1 μL and in less than 5 minutes.^1626^

#### 7.3.2 Optical Sensing

Optical sensing approaches represent the next logical step in the roadmap of the development of micromotors for analytical sensing. Early configurations illustrated the capture of cells (cancer, bacteria, *etc*.) using lectins or antibody-modified tubular micromotors.^141,1627^ Direct visualizations of the captured target (bacteria, modified tags) demonstrated the utility of micromotors to perform immunoassays in localized environments, another unique potential of micromotors in biosensing applications, even in the small channels and reservoirs within microfluidic devices.^1628,1629^ Recently, micromotors have been shown to serve as active carriers for functionalized microparticles via reversible dielectrophoretic trapping, enabling sandwich immunoassay sensing.^1630^ Yet, efforts were directed to the introduction of fluorescence or colorimetric reporters to perform analytical detection of the target analytes toward practical applications. Regarding fluorescence detection approaches, micromotors were combined with inorganic nanoparticles such as quantum dots. For example, PEDOT/Pt micromotors were modified with CdS quantum dots for ON/OFF mercury detection. The principle behind sensing relies on the attachment and ligand exchange of mercury with the Cd, displacing the emission bandgap and resulting in a fluorescence quenching directly related to the analyte concentration.^1207^ Following a similar principle, graphene quantum dots modified with aminophenyl boronic acid have been encapsulated in polycaprolactone/Pt catalytic micromotors and used for bacteria-related endotoxin detection. In this case, the aminophenyl boronic acid group interacts with sugar residues in the endotoxin molecule, resulting in a fluorescence quenching in a concentration-related manner.^1631^ In addition, the latter method allows the detection of bacterial contamination and can assess food quality, achieving detection in just 5 minutes in microliter sample volumes.^913^ Also, fluorescence polymer-based catalytic micromotor-based immunoassays have been proposed for highly sensitive procalcitonin determination in low volumes of plasma samples (25 μL) for sepsis-suspected low-weight neonates at clinically relevant concentrations (0.5–150 ng.mL^–1^), with an excellent agreement with the gold standard immunoassay method used by hospitals (Figure 18b­(i)).^1399^

The use of fluorescence-labeled DNA,^1632^ aptamers and affinity peptides, in ON/OFF approaches has also been evaluated. Catalytic micromotors with outer carbon nanomaterials (mainly graphene) or other 2D nanomaterials (WS_2_, MoS_2_) have mainly been explored. When using aptamers or affinity peptides, the principle of the assay relies on the probe attachment to the nanomaterial *via* π–π or hydrophobic interactions (OFF state). The higher affinity of the analyte toward the detection probe results in the release from the micromotor surface (ON state) in a concentration-dependent manner, with high selectivity, as the probe is specifically designed for recognition of specific, unique chemical features of the given analyte. This has been demonstrated for ricin^1633^ or proteins related to sepsis processes in neonates, using aptamer-modified graphene micromotors, for PCT^1634^ and interleukin-6,^1635^ allowing even dual assays.^1636^ Affinity peptides have been used in connection with 2D nanomaterials-based tubular and Janus micromotors for bacterial-related toxin detection (Figure 18b­(ii)).^1637,1638^ Molecularly imprinted tubular micromotors are an alternative to the use of sophisticated probes, as the tailored recognition abilities are introduced onto the micromotor surfaces.^1639^ The latter approach is convenient for fluorescence analytes, such as phycocyanin^912^ or venom toxins.^1640^ Regarding intracellular detection, ultrasound propulsion in connection with graphene-modified nanowires and fluorescence-labeled DNA probes is an effective approach for intracellular mRNA detection in cancer cells, opening new avenues to extend the technology using other probes and micromotor designs.^138^ For portable detection, this can be performed in 3D-printed platforms that integrate tailored lasers and high optical resolution lenses, all coupled with smartphones.^1641^ Visual micromotor-based detection approaches also offer a convenient solution, allowing direct naked-eye detection or integration into microplate readers for greatly simplified detection. A cortisol-based micromotor assay using PEDOT micromotors in connection with horseradish peroxidase secondary antibodies as tags and 3,3′,5,5′-tetramethylbenzidine allows for naked detection.^1642^ Phenylenediamine isomers have been detected using MnO_2_-based catalytic micromotors by OH and motion-induced generation of the corresponding-colored polymers.^1643^ In this direction, tubular chitosan/Prussian Blue catalytic micromotors can be used for enzyme encapsulation, as illustrated with acetylthiocholinesterase. The micromotors were used in an inhibition assay format for the detection of neostigmine as a nerve agent. Such analyte inhibits the action of the enzyme, preventing poisoning of the Prussian Blue catalytic layer and oxidizing 3,3′,5,5′-tetramethylbenzidine into blue color.^1644^ To have more biocompatible alternatives, fuel-free schemes have been explored. As such, a magnetic micromotor-based assay using bacteriophage-modified micromotors in connection with Au nanoparticles has been integrated into microplate readers for bacteria detection with exquisite selectivity.^1645^ In addition, and interestingly, photophoretic and light-driven micromotors can be combined with the light source of Raman equipment to facilitate surface-enhanced Raman scattering (SERS) sensing by accumulation and amplification of the signal and even preconcentration of the analyte.^1646,1647^

#### 7.3.3 Electrochemical Sensing

Electrochemical sensing using micromotors is very convenient due to its inherent miniaturization and versatility. A few convenient early micromotor-based approaches explored the use of assisted electrochemical detection.^1648,1649,1650^

Antibody-modified carbon-based micromotors have been used for sepsis protein detection, using horseradish peroxidase-labeled antibodies as reporters for facilitated electrochemical detection (Figure 18c­(i)),^1651^ even within microfluidic channels.^1652^ A fuel-free scheme based on the use of magnetic micromotors has been illustrated for viral RNA (COVID) detection by electrochemical approaches.^1653^ Recently, an Alzheimer’s disease diagnosis approach has been explored using both electrochemical immunoassays for Aβ-42 ^1614^ and their related oligomer AβO-42 aptassays^1654^ using on-board catalytic micromotors in low volumes of clinical samples with high significance such as brain tissue, cerebrospinal fluid, and plasma samples. The excellent agreement with gold standard methods, along with the low detection limits (in the pg/mL range) and low volume of sample required (5 μL), testify to the applicability of the approaches for fast and early diagnosis. Indeed, the high binding capacity of the micromotors in connection with the immunorecognition elements in the external layer and the enhanced mixing associated with the catalytic propulsion in the internal layer, allow for rapid detection, excellent selectivity, and high sensitivity. As an alternative to avoid the use of H_2_O_2_, Figure 18c­(ii) illustrates the use of Mg micromotors exploiting OH ions generated during Mg micromotors’ hydrogen-based propulsion for the detection of nonelectroactive analytes, such as phthalates, after basic hydrolysis to phenol, all steps being carried out on a low-volume sample on the screen-printed electrode, showing the suitability of micromotor integration with electrochemical detection.^1649^ Although the field is evolving and considerable efforts have been made towards electrochemical detection, the development of these approaches has been much slower compared to the development of optical or even motion-based approaches.

### 7.4 Application of Micro/Nanorobots in Different Technologies

The application of micro/nanorobots is not restricted to biomedical, environmental, and sensing technologies. Other promising fields where the functionality of micro/nanorobots has been explored include chemical warfare agent decontamination, agricultural technologies, the food industry, and more. Organophosphorus nerve agents used in chemical warfare are highly dangerous due to their ability to cause neuromuscular paralysis and death by inhibiting acetylcholinesterase. To address this, a pioneering study by Wang’s group introduced hybrid tubular micromotors made of ZrO_2_–graphene oxide/Pt for the selective capture and detoxification of these agents.^1655^ The micromotors use ZrO_2_ for effective binding and graphene oxide for increased surface area, enhancing detoxification. Their rapid movement in contaminated solutions, aided by microbubbles from H_2_O_2_ decomposition, accelerates detoxification. Additionally, these microrobots can be regenerated through pH changes and alkali treatments, making them a promising, cost-effective solution for nerve agent decontamination. Chlorpyrifos is another hazardous organophosphorus nerve agent used as both a pesticide and a biological warfare agent. A recent study explored the use of 2D-microrobots made from layered MnPS_3_ and Fe_3_O_4_ nanochains, obtained *via* electrostatic assembly, to enhance the photodegradation of chlorpyrifos.^1656^ These magnetically driven microrobots, which move through vertical tumbling under a rotating magnetic field, utilize the photocatalytic properties of MnPS_3_ to degrade chlorpyrifos. By implementing a programmed swarming mode, the microrobots increase local fluid convection and improve molecular interaction, resulting in an 8.8% higher photodegradation efficiency compared to static hybrids. This highlights their potential for effective nerve agent removal.

Food safety has become a global concern due to potential diseases and fatalities worldwide, leading to substantial socio-economic consequences. Key foodborne pathogens, such as *Campylobacter*, *Salmonella*, *Listeria* monocytogenes, and *Escherichia coli*, are under intense investigation. Self-propelled microrobots, capable of converting energy into locomotion, have revolutionized sensing technology and show immense potential for applications in food safety.

In recent decades, the determination of mycotoxins in food commodities has become increasingly important due to their potent toxicity, which adversely affects the health of both animals and humans. Nowadays, they are regarded as the most significant chronic dietary risk factor, surpassing even food additives or pesticide residues. A study explored the use of tubular micromotors based on reduced graphene/Pt nanoparticles for the assessment of highly concerning mycotoxins.^1657^ This new method offers a powerful fluorescent aptamer-based ON/OFF detection with high sensitivity and selectivity. The same group also investigated the utilization of self-propelled Janus structure microsensors, employing a robust ON/OFF fluorescence sensing approach to detect *Salmonella enterica* endotoxin, a key indicator of food contamination.^913^

For microrobot applications in the agriculture industry, Pumera’s group developed hydrogel-based microrobots made from chitosan and Fe_3_O_4_ to encapsulate and deliver the insecticide ethyl parathion to target pests like mealworm larvae.^1658^ These microrobots, activated by a rotating magnetic field, were ingested by larvae, where the acidic midgut environment triggered ethyl parathion release, leading to high mortality rates. The microrobots achieved 80% mortality for mealworm larvae within 1 hour and 100% elimination after 2 hours, demonstrating their potential for effective pest control.

Besides all these applications, focusing mostly on the removal or the destruction of harmful agents, microrobots can also be advantageously employed for a complementary set of tasks dedicated to the synthesis of high-added-value compounds. They can act as mobile microreactors having specific catalytic properties including, *e.g.*, for enantioselective synthesis.^1658a^ In this latter case, a core–shell type architecture combines a reactive metal core, which by its spontaneous oxidative dissolution provides electrons. They are partially used for generating H_2_ bubbles, ensuring the propulsion of the microreactor, whereas the other fraction is transferred to the shell, composed of an inherently chiral polymer. The latter acts as a heterogeneous catalyst allowing the enantioselective reduction of a prochiral starting compound with over 90% enantiomeric excess. Autonomous motion provides an efficient self-stirring of the solution, therefore avoiding mass transport problems. The efficiency of this chemistry “on-the-fly” strategy can be further improved by combining the system with an external magnetic field, significantly accelerating the motion of the microrobots due to the generated Lorentz force.^1660^ This leads to an enhancement of the overall reaction rate by more than an order of magnitude.

## 8 Technical-Related Up-Scaling Manufacturing

### 8.1 Toward Technical Scale-Up

#### 8.1.1 Rapid Prototyping

Micro/nanorobots have attracted considerable research interest for their potential applications in biomedicine and environmental remediation. Though they may differ in terms of the base materials as well as their dimensions, above all else, micro/nanorobots must be able to provide sufficient propulsion to perform their programmed tasks such as targeted drug delivery and pollutant removal. There are several different innovative strategies suggested in the literature, an example of which is Janus particles, which have a spherical micro/nanorobot body with an asymmetric coating of catalytic materials on one side to achieve directional propulsion.^788,1087,1665^ In addition, micro/nanorobots featuring asymmetric structures with internal cavities, such as hollow tube structures,^33^ yolk–shell structures,^1239^ and bottle-shaped structures,^1666^ have been proposed for cargo encapsulation and transport. It is clear that the overall structure and composition of micro/nanorobots dictate their functionality; however, this also suggests that the micro/nanorobots must be meticulously produced with rigorous quality control to ensure their viability. Unfortunately, conventional synthesis approaches are either highly time-consuming or have limited scalability, hindering their large-scale production for practically relevant applications. Therefore, innovative and effective methods for the uniform mass production of intricately structured micro/nanorobots remain high in demand.

One example of innovation in scalable production methods is found in the work of Lv *et al.*, who introduced a technique for the synthesis of Janus particles based on the Pickering emulsion strategy, which could be a comparatively more scalable method compared to conventional sputtering processes conducted at the laboratory scale.^1667^ The Pickering emulsion technique utilizes the density difference between wax and water to stabilize nanoparticles. Because the yield of resultant micro/nanorobots can be scaled up linearly by increasing the reaction volume, this technique is a potential candidate for mass production. Fu *et al*. proposed a method for creating shuttlecock-shaped silica-based micro/nanorobot bodies through a wet chemical process, and the yield could be scaled-up to hundreds of grams per batch.^1225^ The subsequent NH_2_ surface treatment and enzyme immobilization steps for providing micro/nanorobot propulsion capabilities, also based on wet chemical processes, can be scaled-up as well. This suggests that these methods could facilitate large-scale production, making them promising approaches for the mass production of micro/nanorobots. Additionally, Dai *et al*. explored the fabrication of matchstick-shaped micro/nanorobots by synthesizing TiO_2_ *via* a sol-gel process and subsequently growing SiO_2_ rods to form asymmetric structures, followed by the uniform attachment of Au nanoparticles.^1668^ Liu *et al*. synthesized magnetic Fe_3_O_4_ spheres as the micro/nanorobot body through a simple hydrothermal process.^1669^ Subsequently, they uniformly attached Au nanoparticles to Fe_3_O_4_ micro/nanorobot bodies using HAuCl_4_ and acetic acid in the liquid phase, and through additional processes, transformed them into Au nanostars with a large surface area. This method is highly valuable for mass production processes as it allows for the rapid attachment of catalysts to micro/nanorobot bodies, significantly shortening the time required for the propulsion system process. Yu *et al*. synthesized Fe_2_O_3_ tubular nanorobots using the hydrothermal method, demonstrating the feasibility of mass production by simply providing additional material.^1670^

In the shape-controlled design of micro/nanorobots, materials, structure, and synthetic methods for each component (*i.e.*, body, catalysts, enzymes, functional layers) are vastly different for each research group. This diversity acts as an immense barrier against rapid prototyping of micro/nanorobots. For instance, the difference between TiO_2_ spheres and Fe_2_O_3_ nanorods in terms of their preparation as micro/nanorobots involves two different shape-controlled synthesis protocols as well as two different methods for catalyst decoration based on the respective surface characteristics of TiO_2_ and Fe_2_O_3_. From a research perspective, identifying the principles for changing the structure or material to provide micro/nanorobots asymmetry as well as for adjusting where catalysts are attached may provide insights for developing a rational design of micro/nanorobots through creative and novel synthesis methods. However, from the standpoint of rapid prototyping, it is impractical to devise and apply a different wet chemical synthesis protocol for every possible combination of materials and shapes.

To accelerate the commercialization of micro/nanorobots for a variety of applications, we need to develop robust methods for rapid prototyping and quick evaluation of the properties and performance of a given micro/nanorobot. Factors to consider for rapid prototyping include the design (structure) of micro/nanorobots, the materials composing the micro/nanorobot body, and the catalysts and enzymes to be attached to the micro/nanorobot body. Among these, the processes for attaching catalysts^1661,1671^ or enzymes^35,1672^ to the micro/nanorobot body are not considered as significant barriers for rapid prototyping, as general methods based on wet chemical synthesis are available regardless of the structure and composition of the micro/nanorobot body (Figure 19a and Figure 19b). However, controlling the shape and material of the micro/nanorobot body, for which no general process method exists, requires new synthesis methods and optimization, thereby significantly increasing the processing time.

**Figure 19:**
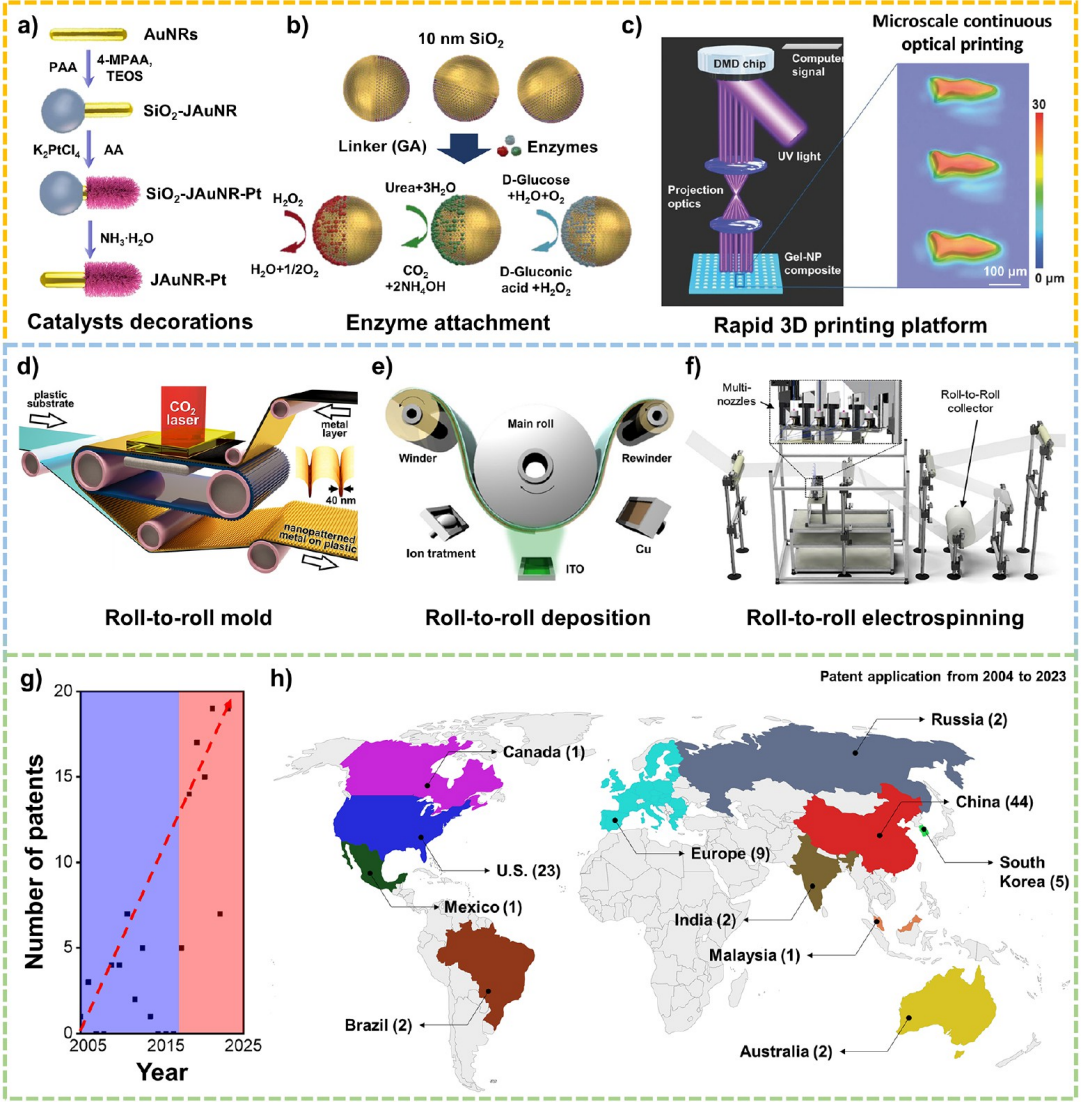
**Introduction to processes capable of swiftly synthesizing various forms of micro/nanorobot bodies for rapid prototyping and methods for attaching enzymes and catalysts to pre-formed micro/nanorobots bodies to equip them with propulsion systems. a)** Catalyst decorations. Reproduced from ref 1661, Copyright 2022 American Chemical Society. **b)** Enzyme attachment. Reproduced from ref 35, Copyright 2015 American Chemical Society. **c)** Rapid 3D printing platform. Introduction to various micro/nanorobots large-scale synthesis processes that enable cost efficiency and quality control for commercialization. Reproduced from ref 1138, Copyright 2015 WILEY-VCH. **d)** Roll-to-roll mold synthesis. Reproduced from ref 1662, Copyright 2016 Springer Nature. **e)** Roll-to-roll deposition system. Reproduced with permission under a Creative Commons CC-BY License from ref 1663, Copyright 2016 Springer Nature. **f)** Roll-to-roll electrospinning system for large-scale micro/nanorobot production. Reproduced from ref 1664, Copyright 2020 American Chemical Society. World map of research area for micro/nanorobots. **g)** Graph of number of patent applications. **h)** Patent world map since 2004.

The simplest method to control the shape and material of micro/nanorobots is through scaffold-assisted synthesis, considering that the goal of rapid prototyping is to quickly check the performance with a small amount of the final synthesized product. Micro/nanorobots with desired shapes and materials can be rapidly synthesized using processes such as layer-by-layer (LbL),^1214^ electrodeposition,^126^ sputtering,^1673^ and atomic layer deposition (ALD),^1674^ on top of prefabricated molds and scaffolds.^1675,1676^ To this end, Wen *et al*. introduced various methods for fabricating anodic aluminum oxide and discussed approaches for creating molds with complex structures.^1569^ Additionally, Wu *et al*. described a method for nano-patterning on desired mold surfaces using nanoimprinting techniques.^1676^ Both techniques are anticipated to significantly aid in the fabrication of molds necessary for micro/nanorobot production and may further be applied to create molds for complex structures (such as flasks, shuttlecocks, *etc*.) by utilizing dual-layer techniques. In this sense, improving the manufacturability of molds into different shapes at the micro- or nanoscale would greatly enhance the potential for rapid prototyping of micro/nanorobots.

Sputtering offers an immediate advantage over other methods since it is very straightforward and viable so long as a suitable sputtering target and mold are available. Other methods, such as LbL assembly and ALD, require specific recipes and optimization, complicating the process. However, for synthesizing molds with high aspect ratios or submicrometer scale micro/nanorobots for which sputtering cannot reach sufficient resolution for conformal coverage, effective synthesis can be accomplished through more sophisticated methods. Zhu *et al*. synthesized fish-shaped microrobots using microscale continuous optical printing, ingeniously incorporating Pt and iron oxide nanoparticles at the tail and head, respectively, to create a directional propulsion system for the microrobot (Figure 19c).^1138^ Ceylan *et al*. utilized a direct laser writing system to fabricate microrobots *via* two-photon polymerization, achieving microscale double-helical complex microswimmer structures.^1101^ This process, capable of synthesizing one microswimmer structure every ten seconds, offers high reproducibility and the ability to print various structures provided there is a 3D design blueprint. Although the absence of higher-resolution laser writing or continuous optical printing systems limits fabrication to microscale robots, these techniques enable rapid verification of whether the intended micro/nanorobot design can achieve propulsion, significantly facilitating rapid prototyping. Ultimately, rapid prototyping will significantly reduce the time and cost for the development of novel designs, drawing the commercialization of micro/nanorobots technology closer to realization.

To summarize, the molds or scaffolds in conjunction with LbL assembly, electroplating, sputtering, ALD, and direct laser writing (3D printing) techniques can be utilized for rapid prototyping in micro/nanorobots. Each technique offers distinct advantages concerning step coverage, processing time, cost-effectiveness, and material versatility. For instance, while ALD is suitable for verifying the operational capability of micro/nanorobots at extremely small sizes, 3D printing is apt for quickly assessing the propulsion feasibility based on the structure of micro/nanorobots.^1674,1677^ Moreover, sputtering is appropriate for swiftly verifying propulsion methods upon changing material types, and LBL is suitable for significantly reducing initial research costs as it does not require specialized equipment.^1678^ These process-specific indicators can facilitate overcoming the most time-consuming aspects of synthesis appropriately, thus enabling the application of rapid prototyping in micro/nanorobot research.^35,1101,1138,1661,1675^

#### 8.1.2 Commercialization of Micro/Nanorobots

The commercialization of micro/nanorobots is challenged by two major factors. The first is the need to undergo multiple synthesis steps to create complex final structures. As the number of process steps increases, so do the requirements for new equipment, material costs, and associated labor costs, leading to an exponential increase in process costs and presenting a significant financial concern for commercialization. In general, the micro/nanorobot design must consider the propulsion direction (through asymmetric catalyst binding on the nanorobots body and imparting structural asymmetry to the nanorobots itself), equip the system for propulsion (using fuel for bubbling, catalysts, or enzymes), and attach functional groups for tasks such as water purification or drug delivery. Consequently, the process inherently involves multiple steps to provide such functionalities.^13,1135,1679^ Therefore, for commercialization, it is essential to consider how much the process steps can be reduced, separate from the prototyping phase, to synthesize the desired shape efficiently.^1680^ The second challenge is the difficulty in producing micro/nanorobots in mass with consistent quality across several synthesis steps.^1681^ Even the slightest fluctuations in experimental conditions and parameters can lead to uncontrolled and undesired shapes, which degrade the propulsion capability, reduce the directionality, and compromise functionality. Attempting to consolidate multiple steps into one to save time and cost could pose a significant risk in terms of quality control. The key considerations for addressing potential issues in micro/nanorobot commercialization include cost efficiency, technical validation and quality control, intellectual property management, and compliance with stability regulations. This section focuses on cost efficiency, technical validation, and quality control.

To enhance cost efficiency, it is essential to establish mass production processes, reduce the number of process steps, lower the manufacturing cost per unit, and ensure a low defect rate. To this end, the sputtering process can employ a high material diversity by simply changing the target, allowing for the synthesis of more complicated geometries. For instance, hollow tube structures could be obtained by sputtering various materials as multilayers on micro/nanopatterned metal molds and then partially etching away the metal mold for a rolling-up synthesis.^1682^ However, for a more elaborate micro/nanorobot design, micro/nanomolds (scaffolds) could be configured in a roll-to-roll manner to incorporate roll-to-roll sputtering or roll-to-roll ALD within the same line. Goswami *et al*. extended laser shock imprinting technology to a roll-to-roll process, introducing a method for roll-to-roll imprinting nanopatterned metal–epoxy nanomolds onto metal substrates (Figure 19d).^1662^ This process, applicable even to very thin metal plates and capable of utilizing various metals, could serve as a method for mass-producing micro/nanopatterned molds affordably. Park *et al*. presented a process system capable of sputtering ITO-Cu-ITO multilayers in a roll-to-roll manner (Figure 19e)^1663^ and Hsieh *et al*. demonstrated the feasibility of synthesizing TiO_2_ *via* ALD in a roll-to-roll process.^1683^ The roll-to-roll process in these deposition techniques plays a crucial role in enhancing the possibility of mass production and significantly reducing process costs. In this case, the mold could even be reused for roll-to-roll synthesis if the etching process is mild enough to remove only the surface layer of the material in contact with the mold. Adjusting the sputtering layer to differentiate the materials inside and outside the hollow tube structure could simultaneously create asymmetry for nanorobot propulsion, reducing process steps and making it a promising technology for mass-producing micro/nanorobot bodies in a cost-efficient manner. Roll-to-roll ALD, although currently limited to composing bodies solely of TiO_2_ or Al_2_O_3_,^1683,1684^ offers excellent step coverage that could overcome the limitations of sputtering in complex molds, potentially enabling the mass production of complex nanorobot bodies. Because mold and deposition processes are used, this approach offers a very low defect rate, making it highly advantageous for quality control.

Another process that can be utilized for the mass production of micro/nanorobot bodies is electrospinning. Electrospinning allows for the mass synthesis of nanofibers with diameters ranging from hundreds of nm to tens of μm by applying a high voltage between a needle containing a melted polymer solution and a metal plate receiving the fibers.^1685,1686^ By mixing a metal precursor into the polymer solution and subsequently heat-treating the formed nanofibers, this process can easily synthesize metal oxide nanofibers, offering a method for mass-producing micro/nanorobot bodies with structures like nanofibers,^1687,1688,1689^ hollow tubes,^1690,1691^ and core-shell.^1692^ Additionally, by adjusting the polymer concentration and voltage level, the same instrument can engage in electrospraying to synthesize sphere-shaped micro/nanorobots,^1693^ offering the advantage of producing micro/nanorobot bodies with various structures. Multi-axial electrospinning techniques can enable the fabrication of multilayered hollow tube^1694^ and fiber structures,^1695^ applying asymmetry to the structure or uniformly binding desired metal catalyst particles to the surface,^1690,1696^ reducing the process steps needed to create a propulsion system on micro/nanorobot bodies. The scalability of electrospinning for mass production involves placing long metal plates capable of applying electric fields and using multiple syringes containing electrospinning solution simultaneously, easily extending it to a large-area nanofiber roll-to-roll synthesis system (Figure 19f).^1664,1697^ The scalability of electrospinning for mass production suggests it could dramatically reduce process costs and is particularly suitable for commercializing micro/nanorobots due to its potential to reduce process steps. Electrospinning is highly sensitive to humidity; hence, conducting the process in an anhydrous room could yield uniform shapes across a wide area in a single process, leading to relatively high uniformity in quality. Another factor affecting quality uniformity is the random orientation of fibers produced through electrospinning. However, using insulating plates to control the electric field could enable the synthesis of aligned nanofibers,^1697^ making it a suitable technology for commercializing micro/nanorobots from a quality control perspective.

The use of a continuous-flow droplet reactor represents one of the methods proposed for attaching propulsion systems to fabricated micro/nanorobot bodies. Highlighted by Niu *et al.*, this approach enables the controlled and uniform growth of Pt-Ni octahedral nanocrystals with specific composition ratios and sizes of Pt and Ni on a carbon substrate.^1698^ With its relatively short process times, capability for uniform catalyst particle growth on surfaces, and potential for scalability in mass production, the continuous-flow droplet reactor may provide a solution for scalable and controllable production of rationally designed micro/nanorobots. Such synthetic control is beneficial for mitigating the risk of uneven catalyst growth due to concentration disparities within the system when scaling up wet chemical processes. This method reduces the occurrence of defective products, thereby presenting an advantageous option for quality control in the production of micro/nanorobots. The process of uniformly attaching enzymes on the micro/nanorobot body is expected to be adaptable and extendable, utilizing various linkers to attach target enzymes.^35,152^ Pre-surface treatment before reactions enables uniform enzyme attachment, leading to a low defect rate even after mass production, thus promising efficiency and quality in enzyme integration on micro/nanorobots.

For commercialization using these proposed processes, it is necessary to establish performance standards for micro/nanorobots and conduct technology validation. Thus, corporations, universities, and research institutions should not only research these processes but also deeply investigate potential issues considering various criteria (toxicity, speed, lifespan, *etc*.) for application in biomedicine and environmental fields, proposing new solutions for suitable applications.

In summary, for the commercialization of micro/nanorobots, methods involving mold-assisted deposition, electrospinning, and the decoration of catalysts or enzymes have been discussed. Mold-assisted deposition, although offering high-quality control, requires expensive deposition systems, making it costly as it only synthesizes the micro/nanorobot body and additional processes are needed for the propulsion scheme. Electrospinning, on the other hand, is cost-effective due to the low price of process equipment and materials, and the possibility to reduce process steps significantly as catalyst binding can be done in a one-pot process. Additionally, if humidity conditions are controlled, it can also exhibit relatively excellent quality control characteristics. Lastly, for creating propulsion systems on micro/nanorobots through catalyst and enzyme decoration, the high potential for mass production and ongoing research into shape control of the final product suggests it as an efficient process in terms of both cost efficiency and quality control.^1662,1663,1664,1698^

#### 8.1.3 Intellectual Property

In the competitive landscape of modern technology, intellectual property rights (IPR) management is vital for protecting research and maintaining a competitive edge, leading to a global push for nanomotor patents. According to the World Intellectual Property Organization (WIPO), a total of 123 filed patents related to the keywords “nanomotors” or “nanorobots” have been published in the last 20 years (2004–2023). In fact, until 2016, there had only been 27 related patents; however, the number of patent applications has increased significantly ever since. Since 2018, tens of patents have been filed annually, reflecting the research progress and the rising potential for practical applications (Figure 19g). These patent applications have been filed worldwide, including 44 in China, 23 in the United States, 9 in Europe, and 5 in Korea, as part of efforts to secure intellectual property rights related to micro/nanorobots (Figure 19h).^1699,1700,1701,1702^ In addition, to protect the intellectual property rights of rapidly advancing micro/nanorobots research internationally, patent applications are not only made through national agencies such as the United States Patent and Trademark Office (USPTO), China National Intellectual Property Administration (CNIPA), Korean Intellectual Property Office (KIPO), and Japan Patent Office (JPO), but also international organizations like the European Patent Office (EPO) and WIPO.

This strategy seeks to protect nanorobots innovations globally, but excessive intellectual property layers in micro/nanorobot fields may hinder research and innovation. The core purpose of intellectual property rights is to acknowledge the efforts and innovations of creators and inventors by protecting their potential market value. This grants exclusive rights to individuals or entities over the results of their research, thereby encouraging further development and innovation. If the ownership of such rights hinders the healthy competition of technological advancements and restricts the free flow of knowledge, then this approach should be avoided by researchers. In the field of micro/nanorobots, characterized by rapid changes and ongoing innovative research, the monopolization of related patents by an individual or corporation could potentially delay or halt many research endeavors, leading to outcomes that are not in the public interest. Considering the significant potential of micro/nanorobot technology in addressing global challenges, such as cancer treatment^1703,1704^ and various environmental issues,^1590,1705^ it is imperative to manage intellectual property rights with the public interest and innovation sustainability in mind. To achieve this, corporations and research institutions must play their part in establishing solid cooperation. Research institutions should engage in lab-scale prototyping and experimental fine-tuning while pursuing key technological breakthroughs, the results of which would lead to filing patents with and initiating technology transfers to partnering corporations. Corporations should leverage these patents and technologies to commercialize these research outcomes into viable products and solutions, realizing the practical potential of the research. The revenues generated from commercial activities should then be re-invested back into research institutions to provide additional momentum for further research and development. This relationship is therefore mutually beneficial, continuously fostering an effective synergy between the two parties. To avert the monopolization of intellectual property rights, it is essential for the micro/nanorobot research community, both domestically and internationally, to cultivate an environment that encourages the sharing of knowledge and technology. Additionally, governments and international bodies should formulate new policies and regulations that promote research and innovation while safeguarding the public interest.

### 8.2 Regulatory Framework

To ensure the practical and reliable use of micro/nanorobots in real-world applications, a robust regulatory framework and multidisciplinary expertise are essential from development to end-user implementation. These frameworks focus on validating material stability, ensuring user safety, and assessing toxicity, which is critical for bridging laboratory research and real-world applications. (Figure 20). First, a comprehensive validation process for the stability, biocompatibility, and lifespan of nanomaterials in micro/nanorobots is crucial. Second, clear regulations on user safety, data privacy, and sustainability are needed to enhance trust and utility. Lastly, toxicity assessments for micro/nanorobots used in applications involving human and environmental exposure are in their early stages of development. Systematic criteria and discussions among experts are essential to holistically evaluate the biological safety of micro/nanorobots, encompassing assessments in blood environments, immune responses, organ toxicity, protein modifications, and immune reactions.

**Figure 20:**
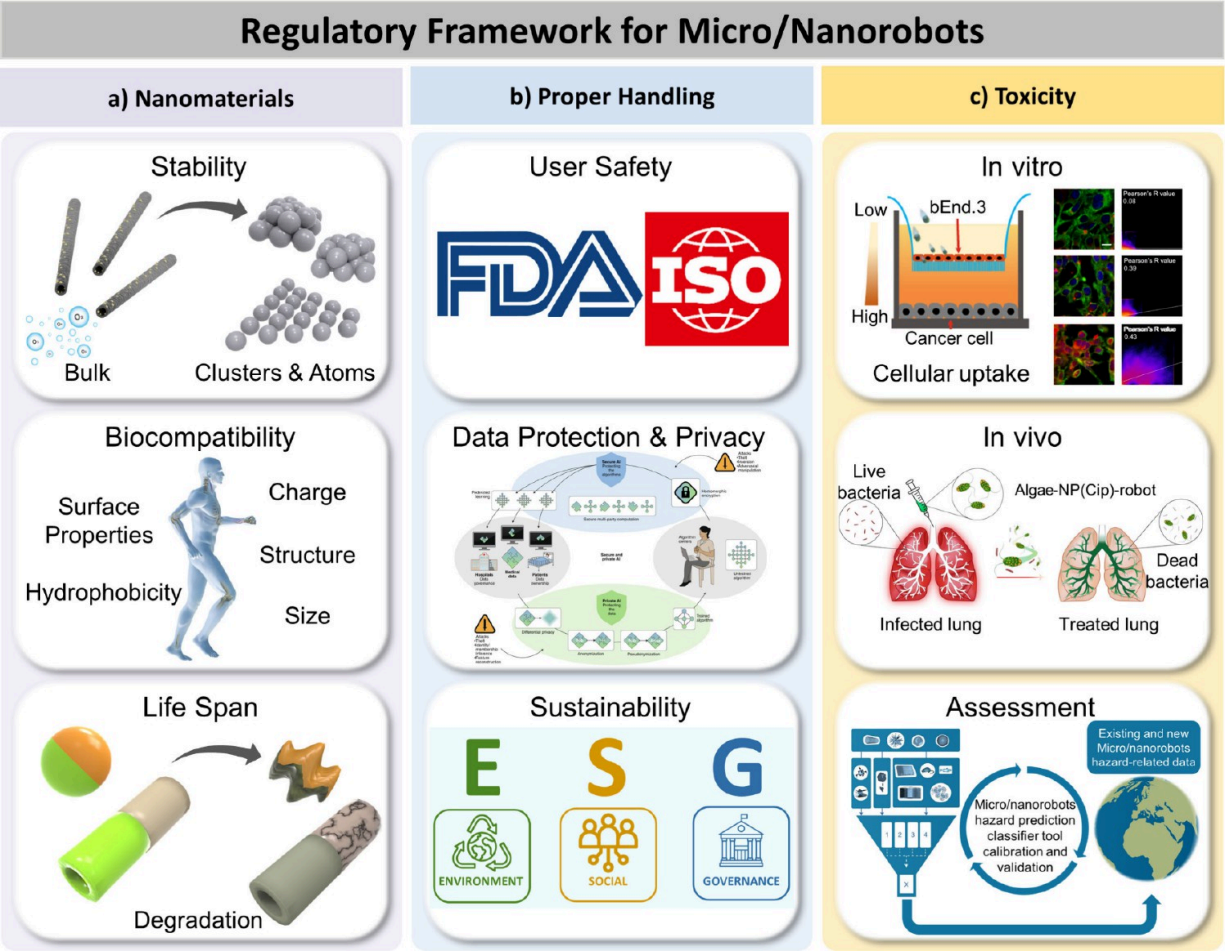
**Key issues to address for the regulatory framework for micro/nanorobots, highlighting the proper handling of nanomaterials and toxicity considerations. a)** In-depth exploration of key perspectives that warrant careful examination concerning nanomaterials predominantly employed in the fabrication of micro/nanorobots. Images under “stability” demonstrate structural alterations in bulk micro/nanorobots in response to specific environmental conditions. Images under “Biocompatibility” illustrate considerations for nanomaterials regarding human compatibility. Images under “Life Span” provide perspectives on issues of degradation when micro/nanorobots are exposed to the human body environment. **b)** Exploring the adequate management of micro/nanorobots as they transition from laboratories to end-users. Images under “User Safety” emphasize the need for institutions to establish regulatory frameworks to ensure user safety in micro/nanorobot management. Images under “Data Protection & Privacy” emphasize the protection of users’ personal information. Adapted with permission from ref 1706, Copyright 2020 Springer Nature. Images under “sustainability” outline a management policy for the market entry of micro/nanorobots from an ESG perspective. **c)** To enhance the universality of micro/nanorobots, establish a rigorous toxicity assessment system focusing on both *in vitro* and *in vivo* aspects. Images under “*In vitro*” reproduced with permission under a Creative Commons CC-BY License from ref 1707, Copyright 2023 Springer Nature. Images under “*In vivo*” adapted with permission from ref 131, Copyright 2022 Springer Nature. Images under “Assessment” adapted with permission from ref 1708, Copyright 2018 Springer Nature.

#### 8.2.1 Nanomaterials Safety

The use of nanomaterials in environmental and biomedical engineering has grown significantly, impacting human healthcare. However, concerns about the stability and toxicity of nanomaterials persist due to their size, which alters physicochemical properties and may trigger new biological reactions. The stability of nanomaterials, which is influenced by size, composition, and surface modifications, can lead to unique interactions within the body due to their nanoscale dimensions (Figure 20a).^1709,1710^

Stability concerns, especially in micro/nanorobots, require careful evaluation, with active communication and collaboration among experts to ensure safe real-world applications. Designing future micro/nanorobots requires biocompatibility not only for the substrate but also for the driving system based on user needs. Factors like surface properties, hydrophobicity, charge, structure, and size^1711,1712^ are crucial for biomedical applications in the bloodstream. Surface interactions can trigger immune responses, so preclinical biocompatibility tests should assess hemolysis, platelet aggregation, clotting time, complement activation, leukocyte proliferation, and macrophage uptake.^1713^

Biological safety is another key consideration when selecting a driving system for micro/nanorobots. Early chemical micro/nanorobots used harmful substances like H_2_O_2_, but enzyme-catalyzed systems powered by physiological fuels like glucose have gained attention. Biological micro/nanorobots, such as those powered by bacteria, neutrophils, or sperm cells, operate naturally to navigate the human body effectively, offering promising avenues for biocompatible application. For example, enzyme-driven nanorobots powered by glucose oxidase have been shown to move across the blood–brain barrier^204^ using physiological glucose levels. These systems ensure biocompatibility and enhance practical applicability, making biocompatibility a crucial factor in driving system selection. Some enzyme-driven micro/nanorobots have been demonstrated to be powered by physiological concentrations of fuel. Additionally, biological micro/nanorobots are designed using motile bacteria,^1714,1715,1716,1717^ neutrophils,^1232^ and sperm cells,^909,1438,1443,1718^ among others, which possess characteristics allowing natural operation within the body. Therefore, considering biocompatibility in the selection of driving systems for micro/nanorobots is crucial.

The limited lifespan of self-propelled micro/nanorobots, often measured in seconds or minutes, poses challenges for sustained operation in real-world applications, necessitating further research into external stimuli and material aging mechanisms. Using external stimuli could extend their operational time, but these methods face limitations in deep body penetration and introduce reliance on external devices. Additionally, the aging of micro/nanorobots, like nanomaterials, is an underexplored issue. Stegemeier *et al*. reported significant differences in the absorption between primitive and aged nanoparticles in plants.^1719^ Most current research on micro/nanorobots has focused on materials that are structurally or chemically stable. Future research must address lifespan, aging, and stability concerns. Collaboration among research groups is essential to overcome these challenges and advance micro/nanorobot technology.^1720^

#### 8.2.2 Proper Handling

Because micro/nanorobots are often custom-made to suit the user’s requirements, clear and universally accepted regulations and limitations must be established for proper handling, user safety, data protection and privacy, and sustainability (Figure 20b).^1721^ Compliance with guidelines from bodies like the U.S. Food and Drug Administration (FDA) and the International Organization for Standardization (ISO), *e.g.*, ISO 10993 for biocompatibility testing, is crucial.^1722^ These standards cover various evaluations, including cytotoxicity and toxicity.^1723^ Recent research highlights the crucial integration of micro/nanorobotics with medical AI,^1724^ especially in medical imaging. Medical imaging is considered crucial among the evaluation criteria for micro/nanorobots.^1352^ AI technology is vital for meeting user demands, ensuring safety, and bridging data between research and clinical use. The use of large image datasets and pre-trained algorithms in oncology and other fields^1706^ benefits from transfer learning, but the scarcity of data and strict patient data regulations pose challenges. To advance this integration, updates to patient data regulations and active collaboration with AI experts are needed.

Integrating ESG (Environmental, Social, Governance) principles in micro/nanorobot development ensures environmentally sustainable practices, social equity, and transparent governance, laying the groundwork for responsible innovation.^1725^ Environmental considerations include using eco-friendly materials and managing potential risks. Socially, technology should promote equity and benefit vulnerable groups while addressing any adverse effects. In terms of governance, adhering to ethical standards and ensuring transparency in research and development are essential. Applying ESG policies ensures that the advancement of micro/nanorobot technology is responsible and sustainable. Establishing regulations for micro/nanorobotic-based on ESG (Environmental, Social, Governance) principles is crucial. While micro/nanorobot technology holds promise across medicine, the environment, and industry, it demands social responsibility and sustainable practices. Environmental considerations include using eco-friendly materials and managing potential risks.^1726,1727^ Socially, technology should promote equity and benefit vulnerable groups while addressing any adverse effects.^1728^ In terms of governance, adhering to ethical standards and ensuring transparency in research and development are essential.^1720^ Applying ESG policies ensures that the advancement of micro/nanorobot technology is responsible and sustainable.

#### 8.2.3 Toxicity

Numerous strategies (Figure 20c) have been developed to address micro/nanorobot toxicity, which is closely tied to biocompatibility and interactions, and requires diverse methods based on tissue location and exposure duration. This includes tests for cell toxicity, sensitization, irritation, systemic and chronic toxicity, and genotoxicity.^1709,1710,1723,1729^ A comprehensive approach should encompass both *in vitro* evaluations at cellular and tissue levels and *in vivo* assessments for applications like drug delivery and tumor therapy.

First, *in vitro* toxicity assessment involves analyses such as MTT assay,^1730^ with most cell toxicity evaluations primarily conducted within test tubes.^1707,1731,1732^ For instance, Chen *et al*. presented a step-wise targeting strategy based on the synergistic effects of chemotaxis and tumor microenvironment reactions, validated by *in vitro* dynamic 3D blood–brain barrier model construction and performance evaluation of nanomotors penetrating the blood-brain barrier.^1707^ Another example is provided by Wan *et al.*, who reported a new chemotherapy mode called “recognition–penetration–reversal–removal” and based on the synergistic effects of nanomotor recognition, vascular penetration, intercellular penetration, specific intracellular distribution (escaping from lysosomes and accumulating in Golgi and nucleus), 3D multicellular tumor spheroid penetration, extracellular matrix degradation, and sustained nitric oxide release performance.^1731^ Investigation of the multidrug resistance reversal mechanism using *in vitro* test tube assays demonstrated the low cytotoxicity of nanorobots.^1731^ This suggests a potential approach to prove the low cytotoxicity of nanorobots, attributed to their low surface electronegativity, and provides insights into future studies on nanorobots.

Next, *in vivo* toxicity assessment is conducted using methods such as animal model testing,^131,1733^ biochemical analysis,^1734,1735^ and imaging analysis.^229,1352^ For example, Zhang *et al*. reported hybrid microrobots coated with antibiotic-containing neutrophil membranes attached to natural microalgae for active antibiotic delivery targeting the lungs *in vivo*.^131^ To assess *in vivo* toxicity, the algae-NP­(Cip)-robot was administered to the lungs, followed by time-tracked examinations. Analysis of cytokines was performed to evaluate inflammatory responses in the hearts, livers, spleens, lungs, and kidneys of mice, with histological sections used to assess inflammation and prove biosafety.^131^ For instance, Zhang *et al.*, demonstrated a microrobotic platform with prolonged *in vivo* operation, swimming behavior inside the body through oral administration to overcome the short propulsion lifespan, and confirming its biosafety *via* comprehensive biochemical analysis.^1734^ For the algae motor capsule platform’s *in vivo* toxicity assessment, comprehensive blood chemistry panels, and hematological evaluations were conducted after administration. Biosafety was confirmed by maintaining all serum biochemical marker levels and blood cell (red blood cell, white blood cell, and platelet) counts at normal levels compared to control mice after capsule administration.^1734^ Such biochemical analysis ensures biosafety of new micro/nanorobot platforms overcoming short propulsion times and presents possibilities for further research advancements.

Despite recent advancements in understanding micro/nanorobot biology, key challenges such as biocompatibility, toxicity, and operational longevity remain unresolved. The Organization for Economic Co-operation and Development (OECD) Nano Working Group noted that while existing chemical testing methods are mostly suitable for nanomaterials, some guidelines need updates.^1708^ Traditional methods are insufficient; validated *in vitro* and *in vivo* tests are needed. AI and high-throughput screening can analyze large datasets to improve safety designs and predict risks, enhancing the reliability of micro/nanorobot safety evaluations.^229,909,1731,1734,1736,1737,1738,1739^

## 9 Burning Issues and Outlook

### 9.1 Fundamental Understanding of Micro/Nanorobots

Micro/nanorobots face fundamental challenges across four key areas: individual propulsion, pairwise interactions, collective behaviors, and robot-environment interactions. At the individual level, understanding propulsion mechanisms is crucial.^1740^ While significant progress has been made, gaps remain in understanding certain mechanisms. For instance, the motion of metallic nanorods driven by MHz ultrasound is not yet fully understood despite theoretical advancements.^59,455,1741^ Micro/nanorobots driven by self-generated gradients present varying levels of clarity. For example, some chemically driven robots, such as the classic Pt-coated microrobots in H_2_O_2_, remain controversial due to incomplete knowledge of the chemical species and concentration profiles driving their motion.^22,1742,1743^

Microrobots rarely locomote individually, especially during complex missions, making it important to understand how they interact with their neighbors. The pairwise interactions are generally better understood both experimentally and theoretically, for externally driven robots than for those driven by chemical gradients. The former often involve dipolar attraction/repulsion and hydrodynamic interactions while the latter require a better understanding of chemical profile evolution and the various ways it leads to pairwise interactions and understand how these chemicals interact with both the surface of the micro/nanorobots and its surrounding boundaries.^1744,1745^

The collective behaviors of micro/nanorobots, such as clustering, schooling, flocking/swarming, predator–prey dynamics, and motion waves, have been extensively discussed in this roadmap. Fundamentally, it is intriguing to explore how these behaviors emerge at the nano- or microscopic level. These questions lie at the heart of the physics of active matter, an emerging frontier attracting materials scientists, chemists, and physicists.^1746,1747,1748^

Finally, how do complex environments influence the speed, direction, and even mode of motion of individual nanorobots and their collective behaviors? For example, the presence of a physical boundary can significantly alter chemical profiles and subsequently change the propulsion of the nanorobots. Despite significant progress, a deeper understanding of the above four key areas—individual propulsion, pairwise interactions, collective behaviors, and environmental interactions—remains essential. This knowledge will not only help elucidate existing phenomena but also enable the prediction and engineering of entirely new functionalities, paving the way for tackling increasingly complex tasks in equally complex environments.

#### 9.1.1 Materials Selection

The selection of materials is crucial for the propulsion and functionality of micro/nanorobots, as it directly impacts their power conversion efficiency, biocompatibility, and application-specific capabilities.^6^ The choice of materials determines the type of propulsion mechanism, such as catalytic, magnetic, or light-driven motion, and influences the ability to integrate additional functionalities like payload loading, and responsiveness to environmental stimuli. Despite significant advancements, several key attributes of biological systems remain absent in synthetic active systems, limiting their ability to fully replicate the complexity and efficiency of natural processes. For instance, biological systems exhibit highly adaptive behavior, self-repair mechanisms, and dynamic responsiveness to diverse environmental stimuli. Additionally, natural systems operate with remarkable energy efficiency and possess the ability to perform complex, multifunctional tasks simultaneously, often leveraging hierarchical structures and cooperative interactions at multiple scales. Incorporating these features into synthetic active systems is essential to achieving true biomimetic functionality, enabling them to autonomously navigate, adapt, and perform tasks in dynamic and unpredictable environments. This integration requires interdisciplinary approaches that merge materials science, biology, and engineering in order to design systems capable of mimicking the intricacies of life.

Another aspect to be considered for material selection is to further scale down the design of micro/nanorobots. Single atom engineering offers an emerging design strategy to tailor the catalytic, magnetic, and optical properties of materials at the atomic scale, enabling precise control over propulsion efficiency, responsiveness, and multifunctionality in micro/nanorobots.^1749^ By leveraging atomically dispersed metals and engineered coordination environments, future nanorobots could achieve enhanced adaptability, energy efficiency, and cooperative behaviors, bridging the gap between synthetic systems and their biological counterparts.^1750^ This approach aligns with the broader concept of nanoarchitectonics,^1751^ a postnanotechnology framework that aims to build functional material systems by architecting atoms, molecules, and nanomaterials as building blocks. Nanoarchitectonics provides a universal methodology to create complex systems, including nanorobots,^1752^ through atomistic, molecular, supramolecular, and biomolecular engineering. Applying nanoarchitectonics to nanorobot design could facilitate unprecedented control over structural hierarchy and functionality, enhancing their potential for biomedical applications through optimized performance, adaptability, and biocompatibility.

#### 9.1.2 Translating toward Clinical Applications

Biocompatibility is the first consideration before the translation of micro/nanorobots into real-world applications. Biocompatible and biodegradable materials are essential for applications in biomedical settings in order to ensure minimal toxicity and effective clearance from the body after accomplishing their intended tasks. These materials should ideally have a well-documented history of safe use in food or pharmaceuticals, verified through scientific research, and recognized as generally regarded as safe by the U.S. Food and Drug Administration.^1753^ Alternatively, if the materials lack such history, extensive toxicological screening through *in vitro* and *in vivo* tests is necessary to confirm they do not produce adverse biological responses.

Another primary challenge in operating nanorobots in biological environments is the rapid formation of a “protein corona” upon exposure to biological fluids like blood. This fouling effect alters nanorobot properties, such as targeting and therapeutic efficacy.^1754^ Researchers have explored how size, shape, and surface properties affect corona composition. While protein opsonization aids immune recognition, it hampers nanorobot efficiency, especially for surface-based reactions. Recent work has demonstrated using super resolution microscopy and proteomics that although protein corona is still formed on enzyme nanomotors, the enzymatic activity slightly reduces and alters the corona while minimally diminishing the speed of nanomotors.^1286^ Future research must focus on integrating antifouling strategies with advanced functionalization techniques to enable robust, efficient nanorobots capable of navigating and performing *in vivo* tasks with precision.

Imaging technologies for micro/nanorobots in biomedical applications have been extensively studied,^1755^ with each modality presenting unique advantages and limitations, such as penetration depth, system complexity, and spatiotemporal resolution.^1383^ Optical imaging, including fluorescence-based tracking, offers high spatiotemporal resolution but suffers from limited tissue penetration.^1756^ Strategies such as functionalizing microrobots with NIR imaging probes and using advanced methods like two-photon fluorescence excitation microscopy address this limitation, allowing for two-/three-dimensional visualization of fluorescent microrobots beneath biological tissues.^1757,1758^ However, despite its potential, two-photon fluorescence excitation microscopy faces challenges like narrow fields of view and invasive requirements.^37,229,1759^ Recent advancements in multiphoton microscopy, such as adaptive optics systems achieving 1.4 mm depth *in vivo*, highlight the promise of integrating NIR-excitable and NIR-emitting imaging probes with noninvasive techniques for precise microrobot tracking and functionality monitoring.^1760,1761^

Overcoming biological barriers, such as the endothelial lining of blood vessels and the blood–brain barrier, is critical for accessing target tissues but remains a significant hurdle.^1352^ Targeting precision is another key challenge. Micro/nanorobots must demonstrate superiority of active targeted delivery capability over inert nanoparticles in order to justify their complex designs and fabrication costs. In addition, controlled *in vitro* assays fail to capture interactions with the extracellular matrix, biodistribution, immune system responses, and long-term effects—all critical for *in vivo* applications.

#### 9.1.3 Environmental Remediation

Unleashing the full potential of micro/nanorobots in water purification requires a strategic fusion of cutting-edge technologies and sustainable design principles. Achieving optimal performance hinges on maximizing contact probability, increasing the number of binding sites for pollutants, and ensuring sufficiently high catalytic activity to eliminate contaminants effectively. Future research should prioritize testing micro/nanorobots in large-scale systems, such as wastewater purification plants, or in open water bodies. These studies would provide critical insights into challenges associated with using fuels in real-world scenarios. Multifunctionality is also vital, showcasing the potential of micro/nanorobots for not only addressing but also sensing water contamination. Beyond water purification, micro/nanorobots hold potential for soil remediation, though significant challenges remain. While self-propelled micro/nanorobots can effectively penetrate soil subsurfaces, their performance and behavior at greater depths require further exploration.^1539^ Factors such as navigation, pollutant interaction, and environmental impact must be thoroughly studied.

In conclusion, an ideal micro/nanorobot for environmental remediation would combine hybrid natural–artificial structures. A biological component could serve as a self-motile scaffold, supporting biocompatible nanostructures that provide navigation and pollutant collection, along with large surface area and catalytic functionalities. Multifunctional materials, potentially integrated with enzymes, would enable pollutant recognition, adsorption, and simultaneous degradation through various mechanisms. Such a design represents a challenging and promising path toward efficient, scalable, and sustainable solutions for environmental challenges.

#### 9.1.4 Multifunctionality

Researchers are urgently pursuing multifunctionality and the ability to operate in complex environments. For example, an ideal drug delivery micro/nanorobot should exhibit accurate autonomous navigation, sensitive diagnostic capabilities, efficient drug delivery and release functions, and the ability to actively decompose and be safely metabolized after completing its task.^1762^ Over years of development, various types of micro/nanorobots have been fabricated, evolving from simple autonomous movement to incorporating a range of advanced functions, such as sensing and detection, multiple driving mechanisms with precise control, and drug delivery and release capabilities. However, the size limitations of micro/nanorobots pose challenges for integrating multiple functions into a small device. A compelling approach to overcoming these challenges was proposed in a comment,^1602^ advocating for streamlined designs that eliminate unnecessary features while maximizing efficiency. This multifunctional design philosophy emphasizes customizing micro/nanorobots’ functions to meet the primary tasks they are designed to perform. Despite progress in the multifunctional design of micro/nanorobots, practical application requirements remain unmet due to the current limitations in micro/nanotechnology. To address these challenges, this paper proposes strategies for achieving multifunctionality in micro/nanorobots by leveraging the multifunctional components of biological systems, developing intelligent responsive materials, and drawing inspiration from swarm intelligence. Another swarming intelligence approach could be the combination of use of various troops where each of them will have one task while maintaining micro/nanorobots as simple as possible (1 troop 1 task).^1763^ These approaches offer promising pathways toward realizing the full potential of multifunctional micro/nanorobots for practical applications in diverse fields, including medicine, environmental remediation, and beyond.

#### 9.1.5 Operation in Extreme Environments

Operation in extreme environments presents a significant challenge for micro/nanorobots, including conditions such as high viscosity, high ionic strength, and rapid liquid flow rates. Compared to chemical-driven micro/nanorobots, those propelled by external fields—such as magnetic or ultrasonic actuation—offer inherent advantages in these challenging environments. Field-driven methods are less sensitive to high ionic environments; however, they still face challenges related to high viscosity and fast-flowing liquids. These issues have been mitigated through structural and shape optimizations, enabling micro/nanorobots to surpass blood flow rates or maintain adherent movement. Conversely, chemical-driven micro/nanorobots are particularly vulnerable in extreme environments, often losing their ability to propel effectively in high or low-ionic-strength conditions. However, enzyme-based nanomotors help penetrating viscous media such as synovial fluids,^1763^ mucus,^1291^ and mucin gels.^1123^ Efforts to improve their performance—such as polymer surface modifications, catalyst optimization, and size reduction—have shown promise.^998,1672^ However, significant progress is still needed to enable chemical-driven micro/nanorobots to function reliably in these environments.

To address these challenges, strategies include optimizing the morphology, structure, and materials of micro/nanorobots to enhance propulsion and resilience in extreme environments, such as high viscosity and ionic strength. A deeper understanding of propulsion mechanisms under these conditions can guide targeted performance improvements. Additionally, exploring novel propulsion methods beyond phoretic effects can increase adaptability and versatility in challenging environments.

#### 9.1.6 Embodied Intelligence

Achieving embodied intelligence enables micro/nanorobots to adapt dynamically to interactions with components, humans, environments, and other robots. Intelligent micro/nanorobots require sensorimotor systems (sensors, actuators, frames, propulsion), a “brain” (circuitry, memory, processors, communication units), and energy systems. These elements facilitate information flow and interaction during operations. Figure 9e illustrates a hypothetical micro/nanorobot design that could achieve embodied intelligence. However, integrating all these components at the micro/nanoscale remains challenging due to gaps between on-board and off-board intelligence and top-down *versus* bottom-up approaches.^1764^

#### 9.1.7 Technology Roadmap

Over the past two decades, significant progress has been made toward bridging these gaps, advancing micro/nanorobot design in five key directions: 1) shifting from rigid to soft, smart materials; 2) evolving from simple to reconfigurable mechanisms; 3) adopting bio-inspired and modular principles; 4) enhancing functionality to be multifunctional and multimodal; and 5) enabling swarm cooperation. While these advancements drive miniaturization and intelligentization, a seamless combination of these strategies is yet to be realized due to persistent technological and conceptual challenges.

To outline a clear vision for the advancement of micro/nanorobots, we propose the 10 most critical questions for the next decade, serving as a foundation for a technology roadmap. These questions are intended to bridge the current understanding of the field with long-term objectives for a practical future of micro/nanorobotics. Achieving these goals will require a dual strategy: advancing fundamental knowledge by addressing the "critical problems" of the micro/nanoworld and driving technical innovation to solve real-world applications.

1) What is the smallest scale of nanorobots?
2) Can we design chemically powered micro/nanorobots that are compatible with their working environments?
3) What is the fundamental principle of collective motion of chemically propelled micro/nanorobots?
4) Can we achieve intelligent micro/nanorobotic swarms with and without AI-assistance?
5) What is the most versatile approach to manipulate micro/nanorobots externally?
6) What is the most suitable strategy to track and monitor micro/nanorobots?
7) Can life-like materials be autonomous and as such, create autonomous micro/nanorobots?
8) Can we fabricate micro/nanorobots that mimic motility, biosensing and shape change characteristics of living microorganisms?
9) Can we enhance medical treatments by swallowing, injecting or inhaling micro/nanorobots?
10) How can we achieve scalable production of micro/nanorobots ensuring precision, quality control, and regulatory compliance?

The field of micro/nanorobots exemplifies the extraordinary potential of interdisciplinary research, uniting nanotechnology, materials science, biology, and engineering to address some of humanity’s most pressing challenges. Advancing the field of micro/nanorobots in the next decades comes with its share of challenges, but we are confident that their transformative potential will far outweigh the obstacles. This technological roadmap review aims to motivate the research community to address critical barriers hindering progress and to explore innovative approaches that can accelerate the development and application of these technologies. We envision a future where experts from diverse disciplines collaborate to unlock the full potential of micro/nanorobots, as such multidisciplinary efforts are crucial for overcoming limitations and achieving groundbreaking advancements in this exciting field.

## Vocabulary

Micro/nanorobots: a broadly encompassing term referring to micro- or nanoscale devices capable of converting energy (chemical, electrical, magnetic, *etc*.) into mechanical motion for controlled locomotion or task execution. This definition includes both systems with moving parts and those relying on nonreciprocal interactions, and applies to swimmers, active colloids, and self-propelled particles across diverse operating environments.
Low Reynolds number: a fluid dynamic regime where viscous forces dominate over inertial forces, typically occurring at microscopic scales (*Re* ≪ 1), resulting in highly laminar, time-reversible flow that significantly constrains locomotion strategies of micro/nanoscale systems.
Self-phoresis: a propulsion mechanism where a particle moves through a fluid by generating its own local gradient in a driving field - such as concentration, electric potential, or temperature - via surface activity, leading to motion without external force.
Collective behavior: emergent, coordinated motion or pattern formation arising from interactions among multiple micro/nanorobots, often mediated by hydrodynamic, chemical, or physical cues, enabling functionalities beyond the capabilities of individual units.
Smart responsive materials: refer to materials capable of making visible and tangible responses to changes in their surrounding environment or external stimuli (such as stress, electricity, magnetism, light, heat, pH, or chemical compounds.
Nanoarchitectonics: A postnanotechnology framework focused on constructing functional material systems by precisely arranging atoms, molecules, and nanomaterials through atomistic, molecular, supramolecular, and biomolecular engineering, enabling complex architectures such as nanorobots with enhanced control over structure and function.
Intelligence: The ability of micro/nanorobots to autonomously accomplish specific tasks in dynamic, unpredictable microscale environments by integrating sensors, actuators, controllers, power sources, and interfaces into a cohesive system. This intelligence allows the robots to perceive and respond to external stimuli, similar to biological systems, enabling them to adapt and function effectively in complex, energy-dissipating, and fluidic conditions.
